# Durability of Protection Against Symptomatic COVID-19 Among Participants of the mRNA-1273 SARS-CoV-2 Vaccine Trial

**DOI:** 10.1001/jamanetworkopen.2022.15984

**Published:** 2022-06-08

**Authors:** Dan-Yu Lin, Lindsey R. Baden, Hana M. El Sahly, Brandon Essink, Kathleen M. Neuzil, Lawrence Corey, Jacqueline Miller

**Affiliations:** 1 Department of Biostatistics, University of North Carolina at Chapel Hill, Chapel Hill; 2 Brigham and Women’s Hospital, Boston, Massachusetts; 3 Department of Medicine, Baylor College of Medicine, Houston, Texas; 4 Meridian Clinical Research, Omaha, Nebraska; 5 Department of Medicine, University of Maryland, Baltimore; 6 Fred Hutchinson Cancer Research Center, Seattle, Washington; 7 Moderna, Cambridge, Massachusetts

## Abstract

This cohort study assesses the durability of protection against symptomatic COVID-19 among participants of the mRNA-1273 SARS-CoV-2 (Moderna) vaccine trial.

## Introduction

Evaluating the durability of protection afforded by COVID-19 vaccines is a public health priority, with the results needed to inform policies around booster vaccinations as well as those around nonpharmaceutical interventions. We considered the mRNA-1273 P301 cohort study, which is an ongoing phase 3, randomized, placebo-controlled trial of 30 415 US adults to evaluate the efficacy and safety of the mRNA-1273 SARS-CoV-2 (Moderna) vaccine.^1,2^ The vaccine efficacy (VE) against symptomatic COVID-19 was estimated at 94.1% at interim analysis and at 93.2% at completion of the blinded phase.^1,2^ Comparison of these 2 estimates would suggest a slight waning of VE. However, this comparison is not sensitive enough to detect the true degree of waning, because the VE estimate was obtained under the assumption that VE is constant during the period of analysis and thus represents a mean of the time-varying vaccine effect over a broad study period, weighted by when the event occurs, rather than the VE at the end of the study period.

## Methods

For this cohort study, we considered the per-protocol population, which included 28 451 participants who tested negative for SARS-CoV-2 at baseline and had received 2 doses of vaccine by the end of the blinded phase. Participants received the first dose between July 27 and October 23, 2020. COVID-19 cases were defined by at least 2 systemic symptoms or at least 1 respiratory sign or symptom and were confirmed by a positive SARS-CoV-2 reverse transcriptase–polymerase chain reaction assay result. The Central Institutional Review Board approved the protocol and the consent forms, and all participants provided written informed consent.^1,2^ The study followed the Strengthening the Reporting of Observational Studies in Epidemiology (STROBE) reporting guideline. The trial protocol is available in Supplement 1.

To obtain precise estimates of protection, we fit a Cox regression model that represents the log hazard ratio for the vaccine effect as a piecewise-linear function of time since vaccination,^3^ with change points placed at 40, 80, and 120 days after dose 1 (at the time points where the slope of the log hazard ratio was expected to change). This formulation enables estimation of the entire curve of VE on the current risk of disease. Because the rate of community transmission varied drastically over time, we use calendar time since study initiation rather than time since participant randomization as the time scale for the analysis,^3,4,5^ such that we compare the disease incidence between vaccinated and unvaccinated persons at the same calendar date. The analysis was performed via the dove2 option of the R package DOVE, version 4.1 (R Foundation for Statistical Computing).^3,4^

## Results

A total of 14 164 patients with 769 cases of COVID-19 were in the placebo group, and 14 287 patients with 56 cases of COVID-19 were in the mRNA-1273 group. The demographic and clinical characteristics of the study participants are described elsewhere.^1,2^ The results of the current analysis are displayed in the Figure. The VE reached 92.6% (95% CI, 80.5%-97.2%) at 40 days after dose 1 and increased gradually to a peak of 94.1% (95% CI, 89.5%-96.7%) at 120 days. The VE started to decrease at approximately 120 days and dropped to 89.6% (95% CI, 41.7%-98.2%) at 200 days. These results show mild waning of VE over time and are more informative about duration of protection than previous estimates.^1,2^ The level of protection was still high even 200 days after dose 1, although there was considerable uncertainty in estimating VE near the end of blinded follow-up.

**Figure.**
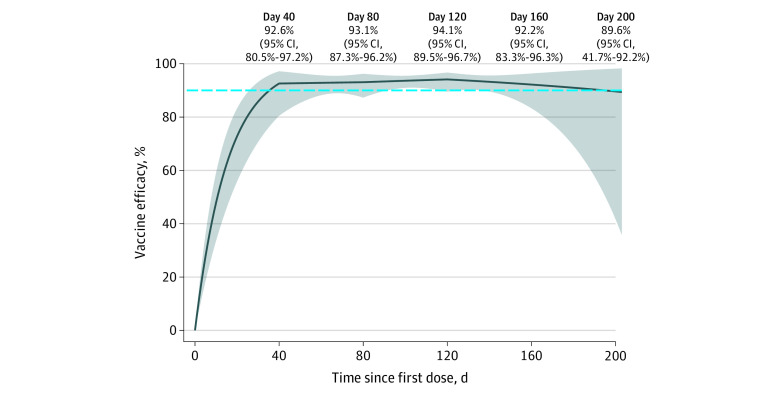
Efficacy of the mRNA-1273 SARS-CoV-2 Vaccine in Reducing the Current Risk of Symptomatic COVID-19 Solid curved line indicates the estimate under a Cox regression model with a piecewise-linear function for the log hazard ratio; shaded area, the corresponding 95% CIs; and dashed line, 90%.

## Discussion

Because of the crossover of placebo recipients to the vaccine arm, the phase 3 trials provide placebo-controlled efficacy data for less than 7 months after dose 1.^3,6^ Indeed, few cases of COVID-19 occurred after 6 months in our study, making it difficult to precisely estimate the degree of waning at the end of the blinded follow-up. Observational studies can provide information about the longer-term benefits of vaccines.

## Notes

Supplement 1.
Trial Protocol

Supplement 2. **Nonauthor Collaborators.** The COVE Study Group
