# Supplementary material for: Durability of Protection Against Symptomatic COVID-19 Among Participants of the mRNA-1273 SARS-CoV-2 Vaccine Trial
Source: JAMA Netw Open. 2022 Jun 8;5(6):e2215984. doi: 10.1001/jamanetworkopen.2022.15984 (PMC9178430; doi:10.1001/jamanetworkopen.2022.15984)
Supplement: Supplement 1. — Trial Protocol [file jamanetwopen-e2215984-s001.pdf]

This supplement contains the following items:

1. Original protocol, final protocol, summary of changes.

2. Original statistical analysis plan, final statistical analysis plan (summary of changes for statistical analysis plan is integrated into document history in final statistical analysis plan)

CLINICAL STUDY PROTOCOL

**Protocol Title:**	A Phase 3, Randomized, Stratified, Observer-Blind, Placebo-Controlled Study to Evaluate the Efficacy, Safety, and Immunogenicity of mRNA-1273 SARS-CoV-2 Vaccine in Adults Aged 18 Years and Older
**Protocol Number:**	mRNA-1273-P301
**Sponsor Name:**	ModernaTX, Inc.
**Legal Registered Address:**	200 Technology Square<br>Cambridge, MA 02139
**Sponsor Contact and Medical Monitor:**	Tal Zaks, MD, PhD, Chief Medical Officer<br>ModernaTX, Inc.<br>200 Technology Square<br>Cambridge, MA 02139<br>Telephone: 1-617-209-5906<br>e-mail: Tal.Zaks@modernatx.com
**Regulatory Agency Identifier Number(s):**	IND: 19745
**Approval Date:**	15 Jun 2020

CONFIDENTIAL

All financial and nonfinancial support for this study will be provided by ModernaTX, Inc. The concepts and information contained in this document or generated during the study are considered proprietary and may not be disclosed in whole or in part without the expressed written consent of ModernaTX, Inc. The study will be conducted according to the *International Council for Harmonisation (ICH) Technical Requirements for Registration of Pharmaceuticals for Human Use, E6(R2) Good Clinical Practice (GCP) Guidance.*

PROTOCOL APPROVAL – SPONSOR SIGNATORY

**Study Title:**	A Phase 3, Randomized, Stratified, Observer-Blind, Placebo-Controlled Study to Evaluate the Efficacy, Safety, and Immunogenicity of mRNA-1273 SARS-CoV-2 Vaccine in Adults Aged 18 Years and Older
**Protocol Number:**	mRNA-1273-P301
**Protocol Version Date:**	Jun

Protocol accepted and approved by:

See esignature and date signed on last page of document.

Tal Zaks, MD, PhD<br>Chief Medical Officer<br>ModernaTX, Inc.<br>200 Technology Square<br>Cambridge, MA 02139<br>Telephone: 1-617-209-5906	Date

DECLARATION OF INVESTIGATOR

I have read and understood all sections of the protocol entitled "A Phase 3, Randomized, Stratified, Observer-Blind, Placebo-Controlled Study to Evaluate the Efficacy, Safety, and Immunogenicity of mRNA-1273 SARS-CoV-2 Vaccine in Adults Aged 18 Years and Older" and the most recent version of the Investigator's Brochure.

I agree to supervise all aspects of the protocol and to conduct the clinical investigation in accordance with the current Protocol, the *International Council for Harmonisation (ICH) Technical Requirements for Registration of Pharmaceuticals for Human Use, E6(R2) Good Clinical Practice (GCP) Guidance,* and all applicable government regulations. I will not make changes to the protocol before consulting with ModernaTX, Inc. or implement protocol changes without IRB/IEC approval except to eliminate an immediate risk to participants.

I agree to administer study treatment only to participants under my personal supervision or the supervision of a sub-investigator. I will not supply study treatment to any person not authorized to receive it. I also agree that persons debarred from conducting or working on clinical studies by any court or regulatory agency will not be allowed to conduct or work on studies for the sponsor or a partnership in which the sponsor is involved. I will immediately disclose it in writing to the sponsor if any person who is involved in the study is debarred, or if any proceeding for debarment is pending, or, to the best of my knowledge, threatened.

I will not disclose confidential information contained in this document including participant information, to anyone other than the recipient study staffs and members of the IRB/IEC. I agree to ensure that this information will not be used for any purpose other than the evaluation or conduct of the clinical investigation without the prior written consent from ModernaTX, Inc. I will not disclose information regarding this clinical investigation or publish results of the investigation without authorization from ModernaTX, Inc.

The signature below provides the necessary assurance that this study will be conducted according to all stipulations of the protocol, including statements regarding confidentiality, and according to local legal and regulatory requirements, US federal regulations, and ICH E6(R2) GCP guidelines.

____________________________________ ____________________

Signature of Principal Investigator Date

____

Printed Name of Principal Investigator

1. PROTOCOL SUMMARY

1.1. Synopsis

**Protocol Number:**	**mRNA-1273-P301**
**Title:**	A Phase 3, Randomized, Stratified, Observer-Blind, Placebo-Controlled Study to Evaluate the Efficacy, Safety, and Immunogenicity of mRNA-1273 SARS-CoV-2 Vaccine in Adults Aged 18 Years and Older
**Study Phase:**	3
**Objectives:**	Primary:<br>• To demonstrate the efficacy of mRNA-1273 to prevent COVID-19.<br>• To evaluate the safety and reactogenicity of 2 injections of mRNA-1273 given 28 days apart.<br>Secondary:<br>• To evaluate the efficacy of mRNA-1273 to prevent severe COVID-19.<br>• To evaluate the efficacy of mRNA-1273 to prevent serologically confirmed SARS-CoV-2 infection or COVID-19 regardless of symptomatology or severity.<br>• To evaluate vaccine efficacy (VE) against a secondary definition of COVID-19.<br>• To evaluate VE to prevent death caused by COVID-19.<br>• To evaluate the efficacy of mRNA-1273 to prevent COVID-19 after the first dose of investigational product (IP).<br>• To evaluate the efficacy of mRNA-1273 to prevent COVID-19 in all study participants, regardless of evidence of prior SARS-CoV-2 infection.<br>• To evaluate the efficacy of mRNA-1273 to prevent asymptomatic SARS-CoV-2 infection.

	Exploratory:<br>• To evaluate the effect of mRNA-1273 on the viral infection kinetics as measured by viral load at SARS-CoV-2 infection diagnosis by RT-PCR and number of days from the estimated date of SARS-CoV-2 infection until undetectable SARS-CoV-2 infection by RT-PCR.<br>• To assess VE to reduce the duration of symptoms of COVID-19.<br>• To assess VE against burden of disease (BOD) due to COVID-19.<br>• To evaluate VE against all-cause mortality.<br>• To evaluate the genetic and/or phenotypic relationships of isolated SARS-CoV-2 strains to the vaccine sequence.<br>• To evaluate immune response markers after dosing with IP as correlates of risk of COVID-19 and as correlates of risk of SARS-CoV-2 infection.<br>• To conduct additional analyses related to furthering the understanding of SARS-CoV-2 infection and COVID-19, including analyses related to the immunology of this or other vaccines, detection of viral infection, and clinical conduct.
**Study Design and Methodology:**	This is a Phase 3, randomized, stratified, observer-blind, placebo-controlled study to evaluate the efficacy, safety, and immunogenicity of mRNA-1273 SARS-CoV-2 vaccine compared to placebo in adults 18 years of age and older who have no known history of SARS-CoV-2 infection but whose locations or circumstances put them at appreciable risk of acquiring COVID-19 and/or SARS-CoV-2 infection. Figure 1 shows the study flow.<br><br>Participants will be randomly assigned to receive injections of either 100 µg of mRNA-1273 vaccine or a placebo control in a 1:1 randomization ratio. Assignment will be stratified by age and health risk. This is a case-driven study and thus final study size will depend on the actual attack rate of COVID-19.<br><br>All participants will be assessed for efficacy and safety endpoints and provide a nasopharyngeal (NP) swab sample and blood sample before the first dose of IP in addition to a series of post-dose blood samples for immunogenicity through 24 months after the second dose of IP. Efficacy assessments will include surveillance for COVID-19 with RT-PCR confirmation of SARS-CoV-2 infection after the first dose of IP. As noted above, this is a case-driven study: if the prespecified criteria for early efficacy are met at the time of either interim analysis or overall efficacy at the primary analysis, a final study report

describing the efficacy and safety of mRNA-1273 will be prepared based on the data available at that time. In the event that success criteria are met either at the time of the interim analyses or when the total number of cases toward the primary endpoint have accrued, participants will continue to be followed in a blinded fashion until Month 25, to enable assessment of long-term safety and durability of VE . If the study concludes early, all participants will be requested to provide a final blood sample at the time of study conclusion.

All participants may have up to 7 scheduled clinic visits, including Screening, Day 1, Day 29, Day 57, Day 209, Day 394, and Day 759. Each participant will receive 2 doses of IP by 0.5 mL intramuscular (IM) injection, the first on Day 1 and the second on Day 29. A nasopharyngeal (NP) swab sample will be collected prior to the first dose of IP, for evaluation by RT-PCR. Participants will be given an electronic diary (eDiary) to report solicited adverse reactions (ARs) for 7 days after each dose of IP and to prompt an unscheduled clinic visit for clinical evaluation and NP swab sample if a participant experiences any symptoms of COVID-19. All participants will receive safety calls 7 days and 14 days after each dose (on Day 8, Day 15, Day 36, and Day 43) that will serve both to monitor AEs possibly related to IP dosing and to monitor for symptoms of COVID-19.

Surveillance for COVID-19 will be performed through weekly contacts with the participant via a combination of telephone calls and completion of an eDiary starting at Day 1 through the end of the study. Participants meeting clinical criteria for COVID-19 will return to the clinic or will be visited at home by medically qualified site staff within 72 hours to collect an NP swab sample for RT-PCR testing for SARS-CoV-2 and other respiratory pathogens, or alternatively, if a clinic or home visit is not possible, will submit a nasal swab or saliva sample for SARS-CoV-2 RT-PCR testing.

Any confirmed COVID-19 occurring in a participant will be captured as an MAAE along with relevant concomitant medications and details about severity, seriousness, and outcome. Study participants will be monitored by medically qualified site personnel for a 28-day period after diagnosis. Each participant diagnosed with COVID-19 will record the presence of symptoms using a paper diary/eDiary for 28 days following the laboratory confirmation of COVID-19. In addition to daily recording of symptoms, assessments will include the collection of weekly samples (NP swab [preferred], nasal swab, or saliva) for 28 days following the diagnosis of SARS-CoV-2 infection. Additionally, a convalescent visit will be scheduled approximately 28 days after diagnosis. At this visit, a blood sample will be collected for serologic assessment of SARS-CoV-2 infection.

At each dosing visit, participants will be instructed (Day 1) or reminded (Day 29) on how to document and report solicited ARs in the eDiary provided. Solicited ARs will be assessed for 7 days after each IP dose and unsolicited AEs will be assessed for 28 days after each IP dose; SAEs, MAAEs, AEs leading to withdrawal, will be assessed throughout the study.

Participants will have scheduled blood sampling (for immunogenicity assessment) at Screening, Day 1, Day 29, Day 57, Day 209, Day 394, and Day 759.

This study will be conducted in compliance with the protocol, Good Clinical Practice (GCP), and all applicable regulatory requirements.

Randomization:

Up to approximately 30,000 participants will be randomly assigned in 1:1 ratio to receive either mRNA-1273 100 μg or placebo. The randomization will be in a blinded manner using a centralized Interactive Response Technology (IRT) at the Day 1 visit, in accordance with pre-generated randomization schedules.

Stratification:

Randomization will be stratified based on age and, if they are < 65 years of age, based on the presence or absence of risk factors for severe illness from COVID-19 based on current CDC recommendation There will be 3 strata for randomization: ≥ 65 years, < 65 years and categorized to be at increased risk (“at risk”) for the complications of COVID-19, and < 65 years “not at risk”. Risk will be defined based on the study participants’ relevant past and current medical history. At least 25% of enrolled participants, but not more than 40%, will be either ≥ 65 years of age or < 65 years of age and “at risk” at Screening.

Participants who are < 65 years old will be categorized as at risk for severe COVID-19 illness if they have at least 1 of the following risk factors at Screening:

- Chronic lung disease (eg, emphysema and chronic bronchitis), idiopathic pulmonary fibrosis and cystic fibrosis) or moderate to severe asthma
- Significant cardiac disease (eg, heart failure, coronary artery disease, congenital heart disease, cardiomyopathies, and pulmonary hypertension)
- Severe obesity (body mass index ≥ 40 kg/m^2)
- Diabetes (Type 1, Type 2 or gestational)
- Liver disease

**Study Population:**	Participants (males and females 18 years of age or older at time of consent), who are adults at risk of SARS-CoV-2 infection who have no known history of SARS-CoV-2 infection, is a subset of the planned target population. Additionally, potential study participants at increased risk of complications from COVID-19 will be included since it is hypothesized that these participants might derive the greatest benefit from a vaccine. Study sites may be selected based on SARS-COV-2 infection risk of the local population. Approximately 30,000 participants will be enrolled.<br>The full lists of inclusion and exclusion criteria are provided in the body of the protocol.
**Efficacy Assessments:**	**Primary Efficacy Assessment:**<br>To be considered as a case of COVID-19 for the evaluation of the Primary Efficacy Endpoint, the following criteria must be met:<br>• The participant must have experienced at least TWO of the following systemic symptoms: Fever (≥ 38ºC), chills, myalgia, headache, sore throat, new olfactory and taste disorder(s), OR<br>• The participant must have experienced at least ONE of the following respiratory signs/symptoms: cough, shortness of breath or difficulty breathing, OR clinical or radiographical evidence of pneumonia; AND<br>• The participant must have at least one NP swab, nasal swab, or saliva sample positive for SARS-CoV-2 by RT-PCR.<br>**Secondary Efficacy Assessments:**<br>To be considered a severe COVID-19, the following criteria must be met: a confirmed COVID-19 as per the Primary Efficacy Endpoint case definition, plus any of the following:<br>• Clinical signs indicative of severe systemic illness, Respiratory Rates ≥ 30 per minute, Heart Rate ≥ 125 beats per minute, $SpO_2 \leq 93\%$ on room air at sea level or $PaO_2/FIO_2 < 300$ mm Hg, OR<br>• Respiratory failure or Acute Respiratory Distress Syndrome (ARDS), (defined as needing high-flow oxygen, non-invasive or mechanical ventilation, or ECMO), evidence of shock (systolic blood pressure < 90 mmHg, diastolic BP < 60 mmHg or requiring vasopressors), OR<br>• Significant acute renal, hepatic or neurologic dysfunction, OR<br>• Admission to an intensive care unit or death.

The secondary case definition of COVID-19 is defined as the following systemic symptoms: fever (temperature >38ºC) or chills, cough, shortness of breath or difficulty breathing, fatigue, muscle aches or body aches, headache, new loss of taste or smell, sore throat, nasal congestion or rhinorrhea, nausea or vomiting or diarrhea AND a positive NP swab, nasal swab, or saliva sample for SARS-CoV-2 by RT-PCR.

Death attributed to COVID-19 is defined as any participant who dies during the study with a cause directly attributed to a complication of COVID-19.

Immunogenicity Assessments:

Immunogenicity assessments will include the following:

- Serum bAb levels against SARS-CoV-2 as measured by enzyme-linked immunosorbent assay (ELISA) specific to the SARS-CoV-2 S protein.
- Serum bAb levels against SARS-CoV-2 as measured by ELISA specific to the SARS-CoV-2 nucleocapsid protein
- Serum nAb titer against SARS-CoV-2 as measured by pseudovirus and/or live virus neutralization assays.
- Seroconversion is defined differently for participants seronegative at Baseline and seropositive at Baseline:
    - Participants seronegative at Baseline: bAb levels against SARS-CoV-2 nucleocapsid either from below the limit of detection (LOD) or lower limit of quantification (LLOQ) at Study Day 1 that increase to above or equal to LOD or LLOQ starting at Study Day 57 or later.
    - Participants seropositive at Baseline: bAb levels against SARS-CoV-2 nucleocapsid above the LOD or LLOQ at study Day 1 that increase by 4-fold or more in participants with pre-existing bAb starting at Study Day 57 or later.

**Safety Assessments:**	Safety assessments will include monitoring and recording of the following for each participant:<br>• Solicited local and systemic ARs that occur during the 7 days following each injection (ie, the day of injection and 6 subsequent days). Solicited ARs will be recorded daily using eDiaries.<br>• Unsolicited AEs observed or reported during the 28 days following each injection (ie, the day of injection and 27 subsequent days).<br>• AEs leading to discontinuation from dosing and/or study participation from Day 1 through Day 759 or withdrawal from the study.<br>• MAAEs from Day 1 through Day 759 or withdrawal from the study.<br>• SAEs from Day 1 through Day 759 or withdrawal from the study.<br>• Abnormal vital sign measurements.<br>• Physical examination findings.
**Investigational Product, Dosage, and Route of Administration:**	The mRNA-1273 IP is an LNP dispersion of an mRNA encoding the prefusion stabilized S protein of SARS-CoV-2 formulated in LNPs composed of 4 lipids (1 proprietary and 3 commercially available): the proprietary ionizable lipid SM-102; cholesterol; 1,2-distearoyl-sn-glycero-3 phosphocholine (DSPC); and 1 monomethoxypolyethyleneglycol-2,3-dimyristylglycerol with polyethylene glycol of average molecular weight 2000 (PEG2000-DMG). The mRNA-1273 is provided as a sterile liquid for injection and is a white to off- white dispersion in appearance, at a concentration of 0.2 mg/mL in 20 mM Tris buffer containing 87 mg/mL sucrose and 10.7 mM sodium acetate at pH 7.5.<br>The placebo is 0.9% sodium chloride (normal saline) injection, which meets the criteria of the United States Pharmacopeia (USP).<br>Investigational product will be administered as an IM injection into the deltoid muscle on a 2-dose injection schedule on Day 1 and Day 29, with at least a 28-day interval between doses. Each injection will have a volume of 0.5 mL and contain mRNA-1273 100 µg, or saline placebo. Preferably, vaccine should be administered into the nondominant arm. The second dose of IP should be administered in the same arm as the first dose.

Unblinded personnel, who will not participate in any other aspect of the study, will perform IP accountability, dose preparation, and IP administration.

Sample Size:

The sample size is driven by the total number of cases to demonstrate VE (mRNA-1273 vs. placebo) to prevent COVID-19. Under the assumption of proportional hazards over time and with 1:1 randomization of mRNA-1273 and placebo, a total of 151 COVID-19 cases will provide 90% power to detect a 60% reduction in hazard rate (60% VE), rejecting the null hypothesis H0: VE ≤ 30%, with 2 IAs at 35% and 70% of the target total number of cases using a 1-sided O'Brien-Fleming boundary for efficacy and a log-rank test statistic with a 1-sided false positive error rate of 0.025. The total number of cases pertains to the Per-Protocol (PP) Set accruing at least 14 days after the second dose. There are 2 planned IAs in this study, which will be performed when approximately 35% and 70% of the target total number of cases have been observed. Up to approximately 30,000 participants will be randomized with the following assumptions:

- The target VE against COVID-19 is 60% (with 95% confidence interval lower bound ruling out 30%, rejecting the null hypothesis H0: VE ≤ 30%).
- A 6-month COVID-19 incidence rate of 0.75% in the placebo arm.
- An annual dropout rate of 2% (loss of evaluable participants).
- Fifteen percent of the participants are seropositive at baseline, are non-compliant with injection assessments, or have major protocol deviations.
- Two IAs at 35% and 70% of total target cases across the 2 treatment groups with O'Brien-Fleming boundaries for efficacy monitoring.
- 3-month uniform accrual.
- Approximately 15% of participants will be excluded from the PP population, and participants are at risk for COVID-19 starting 14 days after the second dose.

Power for Selected Secondary Efficacy Endpoints:

For the secondary objective on VE against virologically confirmed SARS-CoV-2 infection or COVID-19 regardless of symptomology or severity (COV-INF), the study will have ≥ 90% power to demonstrate the VE is above 30% (to reject null hypothesis VE ≤ 30%) at 1-sided alpha of 2.5% if the true VE to prevent

COV-INF is 60%, because every COVID-19 endpoint is necessarily a COV-INF endpoint.

For the secondary objective on VE against severe COVID-19, the power of demonstrating VE based on a total of 20 and 30 events under different scenarios of true VE and VE criteria has been calculated.

Statistical Methods:

Statistical Hypotheses: For the primary efficacy objective, the null hypothesis of this study is that the VE of mRNA-1273 to prevent first occurrence of COVID-19 is ≤ 30% (ie, $H_0^{efficacy}$: VE ≤ 0.3).

The study will be considered to meet the primary efficacy objective if the corresponding CI of VE rules out 30% at either one of the interim analyses or at the primary analysis. In the primary analysis of VE of COVID-19, cases will be counted starting 14 days after the second dose of IP.

Vaccine efficacy is defined as the percent reduction in the hazard of the primary endpoint (mRNA-1273 vs placebo). Equivalently, the null hypothesis is:

- $H_0^{efficacy}$: hazard ratio (HR) ≥ 0.7 (equivalently, proportional hazards VE ≤ 0.3).

A stratified Cox proportional hazard model will be used to assess the magnitude of the treatment group difference (ie, HR) between mRNA-1273 and placebo at a 1-sided 0.025 significance level.

Analysis Populations: Analysis populations for statistical analyses are Randomization Set, Full Analysis Set (FAS), Modified Intent-to-Treat (mITT) Set, PP Set, Immunogenicity Subset, Solicited Safety Set, and Safety Set, as shown in the table below:

**Population**	**Description**
Randomization Set	All participants who are randomized, regardless of the participants' treatment status in the study.
Full Analysis Set (FAS)	All randomized participants who received at least one dose of IP.<br>Participants will be analyzed according to the group to which they were randomized.

Modified Intent-to-Treat (mITT) Set	All participants in the FAS who had no serologic or virologic evidence of prior COVID-19 or SARS-CoV-2 infection defined by bAb against SARS-CoV-2 nucleocapsid below LOD or LLOQ at Day 1 before the first dose of IP.<br>Participants will be analyzed according to the group to which they were randomized.
Per-protocol (PP) Set	All participants in the mITT Set who received planned doses of IP per schedule and have no major protocol deviations.<br>Participants will be analyzed according to the group to which they were randomized.
Immunogenicity Subset	All participants in the FAS who had a valid immunogenicity test result prior to the first dose of IP and at least 1 valid result after the first dose of IP.
Solicited Safety Set	The Solicited Safety Set consists of all randomized participants who received at least one dose of IP and contributed any solicited AR data.<br>The Solicited Safety Set will be used for the analyses of solicited ARs and participants will be included in the treatment group corresponding to the IP that they actually received.
Safety Set	All randomized participants who received at least one dose of IP. The Safety Set will be used for all analyses of safety except for the solicited ARs.<br>Participants will be included in the treatment group corresponding to the IP that they actually received.

Efficacy Analyses: Efficacy analyses will be performed using the FAS, mITT and PP populations, and participants will be included in the treatment group to which they are randomized. The primary analysis population for efficacy will be the PP Set.

The Table below summarizes the analysis approach for primary and secondary efficacy endpoints. Sensitivity analysis methods are described for each endpoint as applicable.

Endpoint	Statistical Analysis Methods
**Primary endpoint:**<br>Vaccine efficacy (VE) of mRNA-1273 to prevent COVID-19	• Primary analysis: VE will be estimated with 1 - HR (mRNA-1273 vs placebo) using a Cox proportional hazard regression model with treatment group as a fixed effect and adjust for stratification factor based on the PP Set, with cases counted starting 14 days after the second dose of IP.<br>• Analysis using the same model based on the mITT Set.<br>• Sensitivity analysis using the same model based on the PP Set, with cases counted starting either immediately after the second dose of IP or immediately after the first dose of IP.<br>• Subgroup analysis of the primary efficacy endpoint will be performed to assess consistency of VE, such as in the age groups ≥ 18 and < 65 years and ≥ 65 years.<br>• Supportive analysis of VE to be estimated with 1 - ratio of incidence rates with 95% confidence interval (CI) using the exact method conditional upon the total number of cases.<br>• Supportive analysis of cumulative incidence VE.
**Secondary endpoints:**<br>• Vaccine efficacy of mRNA-1273 to prevent severe COVID-19<br>• Vaccine efficacy of mRNA-1273 to prevent serologically confirmed SARS-CoV-2 infection or	Similar analysis method as for the primary endpoint analysis.<br>For each of the secondary endpoints:<br>• Primary analysis: VE will be estimated with 1 - HR (mRNA-1273 vs placebo) using a Cox proportional hazard regression model with treatment group as a fixed effect and adjusting for stratification factor based on the PP Set, with cases

COVID-19 regardless of symptomatology or severity.<br>• Vaccine efficacy of mRNA-1273 to prevent COVID-19 using a secondary definition of symptoms<br>• Vaccine efficacy of mRNA-1273 to prevent death caused by COVID-19<br>• Vaccine efficacy of mRNA-1273 to prevent COVID-19 after the first dose of IP<br>• Vaccine efficacy of mRNA-1273 to prevent asymptomatic SARS-CoV-2 infection	counted starting 14 days after the second dose of IP.<br>• Analysis using the same model based on the mITT Set.<br>• Sensitivity analyses with cases counted starting immediately after the second dose of IP, 14 days after the first dose of IP, immediately after the first dose of IP, and immediately after randomization.<br>• Vaccine efficacy and 95% CI based on the incidence of will be estimated with 1 - ratio of incidence rates using the exact method conditional upon the total number of cases.
Vaccine efficacy of mRNA-1273 to prevent all-cause mortality.	Similar analysis method as for the primary endpoint analysis using PP Set, mITT Set, and FAS. Death, regardless of cause, from time of randomization will be included.
Vaccine efficacy of mRNA-1273 to prevent COVID-19 in all study participants, regardless of evidence of prior SARS-CoV-2 infection	The FAS population will be used for this secondary objective, using similar analysis methods as for the primary endpoint analysis.<br><br>• Primary analysis: VE will be estimated with 1 - HR (mRNA-1273 vs placebo) using a Cox proportional hazard regression model with treatment group as a fixed effect and adjusting for stratification factor based on the FAS, with cases

	counted starting 14 days after the second dose of IP.<br>• Sensitivity analyses with cases counted starting immediately after the second dose of IP, 14 days after the first dose of IP, immediately after the first dose of IP, and immediately after randomization.

Safety: Safety and reactogenicity will be assessed by clinical review of all relevant parameters including solicited ARs (local and systemic events), unsolicited AEs, SAEs, MAAEs, AEs leading to discontinuation, abnormal vital signs, and physical examination findings.

All safety analyses will be based on the Safety Set, except summaries of solicited ARs, which will be based on the Solicited Safety Set. All safety analyses will be provided by treatment group and baseline SARS-CoV-2 serostatus, unless otherwise specified.

The number and percentage of participants with any solicited local AR, with any solicited systemic AR, and with any solicited AR during the 7-day follow-up period after each injection will be provided. A 2-sided 95% exact CI using the Clopper-Pearson method will be also provided for the percentage of participants with any solicited AR for each treatment group.

The number and percentage of participants with solicited ARs, unsolicited AEs, SAEs, MAAEs, Grade 3 or higher ARs and AEs, and AEs leading to discontinuation from study vaccine or participation in the study will be summarized. Unsolicited AE will be presented by MedDRA preferred term and system organ class.

For all other safety parameters, descriptive summary statistics will be provided.

Further details will be described in the statistical analysis plan (SAP).

Immunogenicity: The secondary immunogenicity endpoints will be analyzed using the Immunogenicity Subset, by treatment group and by baseline SARS-CoV-2 serostatus, unless otherwise specified.

The SAP will describe the complete set of immunogenicity analyses, including the approach to sample individuals into an immunogenicity subset for characterizing vaccine immunogenicity and assessing immunological correlates of risk and protection.

Data from quantitative immunogenicity assays will be summarized for each treatment group using positive response rates and geometric means with 95% confidence intervals, for each timepoint for which an assessment is performed. Data from qualitative (ie, yielding a positive or negative result) assays will be summarized by tabulating the frequency of positive responses for each assay by group at each timepoint that an assessment is performed. Analyses will focus on the 2 key immunogenicity time points and the change in marker response between them: Day 1 before the first dose of IP and Day 57 (28 days after the second dose of IP). The SAP will describe the complete set of immunogenicity analyses.

Quantitative levels or geometric mean titer (GMT) of specific bAb with corresponding 95% CI at each timepoint and geometric mean fold rise (GMFR) of specific bAb with corresponding 95% CI at each post-baseline timepoint over pre-dose baseline at Day 1 will be provided by study arm. Descriptive summary statistics including median, minimum, and maximum will also be provided.

GMT of specific nAb with corresponding 95% CI at each timepoint and GMFR of specific nAb with corresponding 95% CI at each post-baseline timepoint over pre-dose baseline at Day 1 will be provided by study arm. Descriptive summary statistics including median, minimum, and maximum will also be provided. For summarizations of group variables values, antibody values reported as below the LOD or LLOQ will be replaced by 0.5 × LOD or 0.5 × LLOQ. Values that are greater than the upper limit of quantification (ULOQ) will be converted to the ULOQ.

The number and percentage of participants with GMFR ≥ 2, GMFR ≥ 3, and GMFR ≥ 4 of serum SARS-CoV-2-specific nAb titers and participants with seroconversion from baseline will be provided with 2-sided 95% CI using the Clopper-Pearson method at each post-baseline timepoint. Seroconversion at a participant level is defined as a change of bAb titer against SARS-CoV-2 nucleocapsid antigen from below the LOD or LLOQ to equal to or above LOD or LLOQ (respectively), or a 4-times or higher log-transformed titer ratio in participants with pre-existing bAb titers.

Geometric mean ratio (GMR) of mRNA-1273 arm versus Placebo arm and its 2-sides 95% CI will be calculated at each study timepoint using an analysis of covariance (ANCOVA) model with the treatment group as an exploratory variable, the analysis may adjust for the stratification factor.

1.2. Schema

Figure 1: Study Flow Diagram

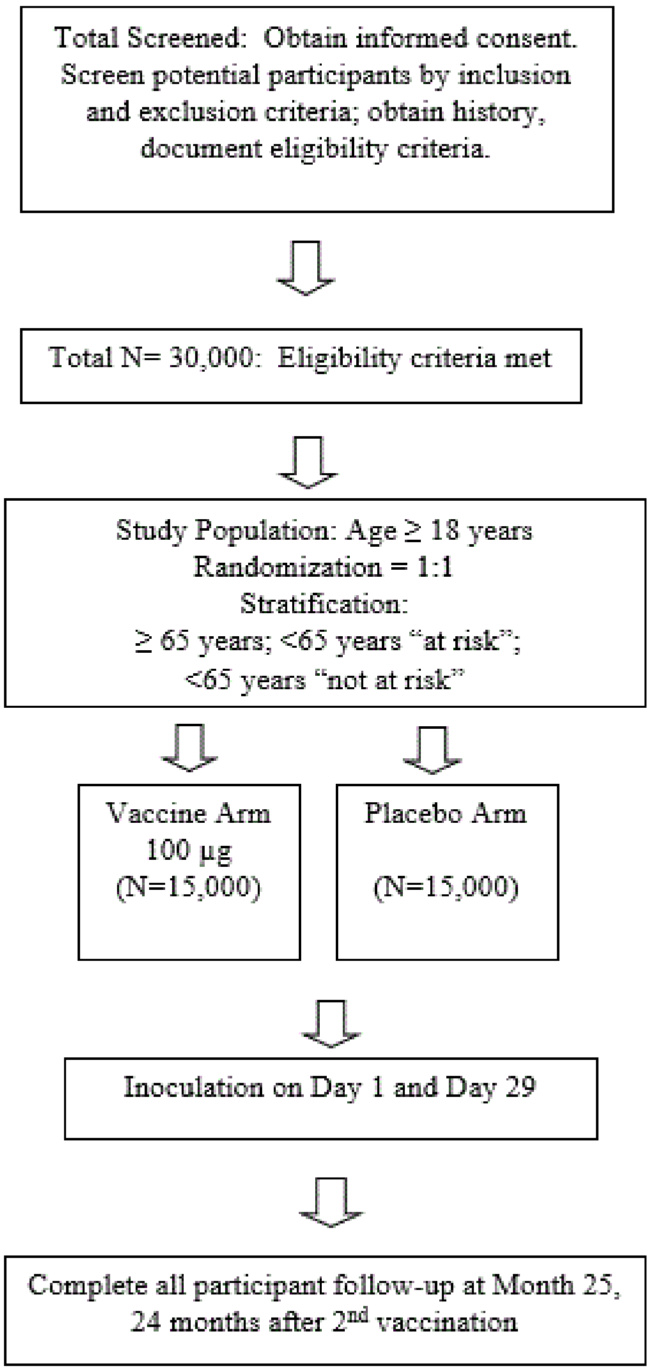

TABLE OF CONTENTS

Clinical study Protocol **1**

Protocol Approval – Sponsor Signatory **2**

Declaration of Investigator **3**

1. Protocol Summary **4**

1.1. Synopsis 4

1.2. Schema 18

Table of Contents **19**

List of Tables **23**

List of Figures **23**

List of Abbreviations **24**

2. Introduction **27**

2.1. Study Rationale 27

2.2. Background and Overview 27

2.2.1. Nonclinical Studies 28

2.2.2. Clinical Studies 28

2.3. Benefit/Risk Assessment 29

2.3.1. Potential Benefits of Study Participation 29

2.3.2. Risks from Study Participation 30

2.3.3. Overall Benefit/Risk Conclusion 31

3. Objectives and endpoints **32**

4. Study Design **36**

4.1. General Design 36

4.2. Scientific Rationale for Study Design 39

4.3. Choice of Dose and Control Product 39

4.4. End of Study Definition 40

5. Study Population **41**

5.1. Inclusion Criteria 41

5.2. Exclusion Criteria 42

5.3. Participant Restrictions 43

5.4. Screen Failures 43

6. Study Treatment **45**

6.1. Investigational Product 45

6.2. Dosing and Management of Investigational Product 45

6.2.1. Method of Randomly Assigning Participants to Treatment Groups 45

6.2.1.1. Stratification 46

6.2.2. Administration of Investigational Product 46

6.2.3. Delivery and Receipt 47
6.2.4. Packaging and Labeling 47
6.2.5. Storage 48
6.2.6. Investigational Product Accountability 48
6.2.7. Handling and Disposal 48
6.2.8. Unblinding 49
6.2.8.1. Planned Unblinding 49
6.2.8.2. Unplanned Unblinding 50
6.3. Study Treatment Compliance 50
6.4. Prior and Concomitant Therapy 51
6.4.1. Prior Medications and Therapies 51
6.4.2. Concomitant Medications and Therapies 51
6.4.3. Concomitant Medications and Vaccines that May Lead to the Elimination of a Participant from Per-Protocol Analyses 52
7. Delaying or Discontinuing Study Treament and Participant Withdrawal from the Study 53
7.1. Criteria for Delay of Study Treatment 53
7.2. Discontinuation of Study Treatment 53
7.3. Participant Withdrawal from the Study 55
7.3.1. Participant Withdrawal 55
7.4. Lost to Follow-up 56
8. Study Assessments and Procedures 57
8.1. Efficacy and Immunogenicity Assessments and Procedures 58
8.1.1. Efficacy Assessments Related to COVID-19 and SARS-CoV-2 Infection 58
8.1.2. Surveillance for COVID-19 Symptoms 59
8.1.3. Follow up/Convalescent Period After Diagnosis with COVID-19 61
8.1.4. Ancillary Supplies for Participant Use 62
8.1.5. Immunogenicity Assessments 62
8.2. Safety Assessments 63
8.2.1. Safety Phone Calls 63
8.2.2. Use of Electronic Diaries or Paper Diaries 64
8.2.3. Demographics/Medical History 65
8.2.4. Physical Examination 66
8.2.5. Vital Sign Measurements 66
8.2.6. Blood Sampling Volumes 67
8.3. Safety Definitions and Procedures 67
8.3.1. Adverse Event 67
8.3.2. Medically Attended Adverse Events 68
8.3.3. Serious Adverse Events 68

8.3.4. Solicited Adverse Reactions 69
8.3.5. Recording and Follow-up of Pregnancy 71
8.3.6. Recording and Follow-up of an AE and/or SAE 72
8.3.7. Time Period and Frequency for Collecting AE and SAE Information 72
8.3.8. Assessment of Intensity 72
8.3.9. Assessment of Causality 73
8.3.10. Reporting Adverse Events 73
8.3.11. Regulatory Reporting Requirements for SAEs 74
8.4. Monitoring Committees 75
8.4.1. Protocol Safety Review Team 75
8.4.2. Data and Safety Monitoring Board 75
8.4.3. Adjudication Committee 76
8.5. Management of Overdose 76
8.6. Pharmacokinetics 77
8.7. Pharmacodynamics 77
8.8. Exploratory Assessments and Biomarkers 77
8.9. Medical Resource Utilization and Health Economics 77
9. Statistical Considerations 78
9.1. Blinding and Responsibility for Analyses 78
9.2. Statistical Hypotheses 78
9.3. Sample Size Determination 79
9.3.1. Power for Selected Secondary Efficacy Endpoints 81
9.4. Analysis Populations 82
9.5. Statistical Analyses 83
9.5.1. Efficacy Analyses 84
9.5.1.1. Efficacy Analysis on Primary Endpoint 86
9.5.1.2. Efficacy Analysis on Secondary Endpoint 87
9.5.2. Safety Analyses 88
9.5.2.1. Adverse Events 88
9.5.2.2. Baseline Descriptive Statistics 89
9.5.3. Immunogenicity Analyses 89
9.5.4. Exploratory Analyses 91
9.5.5. Subgroup Analyses 91
9.6. Interim Analyses 92
10. References 94
11. Supporting Documentation and Operational Considerations 97
11.1. APPENDIX 1: Schedules of Events 98
11.2. APPENDIX 2: Study Governance Considerations 109

11.2.1. Regulatory and Ethical Considerations 109
11.2.2. Study Monitoring 109
11.2.3. Audits and Inspections 111
11.2.4. Financial Disclosure 111
11.2.5. Recruitment Procedures 111
11.2.6. Informed Consent Process 112
11.2.7. Protocol Amendments 113
11.2.8. Protocol Deviations 113
11.2.9. Data Protection 113
11.2.10. Sample Retention and Future Biomedical Research 114
11.2.11. Data Quality Assurance and Quality Control 115
11.2.12. Data Collection and Management 116
11.2.13. Source Documents 116
11.2.14. Retention of Records 117
11.2.15. Study and Site Closure 117
11.2.16. Publication Policy 118
11.3. APPENDIX 3: Contraceptive Guidance 119
11.4. APPENDIX 4 - Statistical Appendices 121
11.4.1. Estimands and Estimand Specifications 121
11.4.2. Statistical Methods and Sensitivity Analyses 124

LIST OF TABLES

Table 1: Objectives and Endpoints 32
Table 2: Summary of Treatment Groups 45
Table 3: Maximum Planned Blood Sampling Volumes per Participant by Visit67
Table 4: Solicited Adverse Reactions and Grades 70
Table 5: Conditions and Sample Size to Demonstrate Vaccine Efficacy 80
Table 6: Power of Demonstrating Vaccine Efficacy Against Severe COVID-1982
Table 7: Populations for Analyses 82
Table 8: Statistical Analysis Methods of Efficacy Endpoints....................85
Table 9: Analysis Strategy for Safety Parameters....................88
Table 10: Immunogenicity Endpoints and Statistical Methods 90
Table 11: Burden of Disease Score....................91
Table 12: Interim Boundaries Using O'Brien-Fleming Spending function, Calculation Based on the PP Set for the Primary Efficacy Endpoint .92
Table 13: Schedule of Events (Vaccination Phase, Day 1 – Day 57)....................99
Table 14: Schedule of Events (Surveillance Phase, Day 64 – Day 394) 102
Table 15: Schedule of Events (Surveillance Phase, Day 401 – Day 759) 105
Table 16: Schedule of Events (28-Day Follow-up After Confirmation of COVID-19) 107
Table 17: Intercurrent Event Types....................121
Table 18: Primary Objective and Estimands with Rationale for Strategies to Address Intercurrent Events for Per Protocol Analysis 122
Table 19: Summary of Statistical Methods and Sensitivity Analyses 124

LIST OF FIGURES

Figure 1: Study Flow Diagram....................18
Figure 2: Event/Case-Driven Study with 24 Months of Planned Follow-Up 37
Figure 3: Surveillance for COVID-19 Symptoms and the Corresponding Clinical Data Pathways....................60
Figure 4: Boundary Crossing Probabilities by Effect Size....................80

LIST OF ABBREVIATIONS

The following abbreviations and terms are used in this study protocol.

Abbreviation	Definition
AC	Adjudication Committee
AE	adverse event
AR	adverse reaction
ARDS	acute respiratory distress syndrome
bAb	binding antibody
BOD	burden of disease
BUN	blood urea nitrogen
CDC	US Centers for Disease Control and Prevention
CFR	Code of Federal Regulations
CI	confidence interval
CMV	cytomegalovirus
CONSORT	Consolidated Standards of Reporting Trials
CoV	coronavirus
COV-INF	virologically confirmed SARS-CoV-2 infection regardless of symptomology or severity
CRO	contract research organization
CSR	clinical study report
DHHS	Department of Health and Human Services
DMID	Division of Microbiology and Infectious Diseases
DSMB	Data and Safety Monitoring Board
DSPC	1,2-distearoyl-sn-glycero-3-phosphocholine
eCRF	electronic case report form
eDiary	electronic diary
ELISA	enzyme-linked immunoabsorbent assay
FAS	full analysis set
FDA	Food and Drug Administration
FSH	follicle-stimulating hormone
GCP	Good Clinical Practice
GMFR	geometric mean fold-rise
GMP	Good Manufacturing Practice
GMT	geometric mean titer
HCP	healthcare practitioner

Abbreviation	Definition
hMPV	human metapneumovirus
HR	hazard ratio
HRT	hormonal replacement therapy
IA	interim analysis
IB	investigator's brochure
ICF	informed consent form
ICH	International Council for Harmonisation
IM	intramuscular
IP	investigational product
IRB	institutional review board
IRT	interactive response technology
LB	lower boundary
LLOQ	lower limit of quantification
LNP	lipid nanoparticle
LOD	limit of detection
MAAE	medically attended adverse event
MedDRA	Medical Dictionary for Regulatory Activities
MERS-CoV	Middle East Respiratory Syndrome coronavirus
mRNA	messenger RNA
nAb	neutralizing antibody
NIAID	National Institute of Allergy and Infectious Diseases
NP	nasopharyngeal
PCR	polymerase chain reaction
PEG2000-DMG	1-monomethoxypolyethyleneglycol-2,3-dimyristylglycerol with polyethylene glycol of average molecular weight 2000
PIV3	parainfluenza virus type 3
PP	per-protocol
RT-PCR	reverse transcriptase polymerase chain reaction
S	spike
S-2P	spike protein with 2 proline residues introduced for stability in a prefusion conformation
SAE	serious adverse event
SAP	statistical analysis plan
SARS-CoV	Severe Acute Respiratory Syndrome coronavirus

Abbreviation	Definition
SM-102	heptadecan-9-yl 8-((2-hydroxyethyl) (6-oxo-6-(undecyloxy) hexyl) amino) octanoate
SoE	Schedule of Events
TEAE	treatment-emergent adverse event
ULOQ	upper limit of quantification
USP	United States Pharmacopeia
VE	vaccine efficacy
WHO	World Health Organization

2. INTRODUCTION

2.1. Study Rationale

Coronaviruses (CoVs) are a large family of viruses that cause illness ranging from the common cold to more severe diseases, such as Middle East Respiratory Syndrome (MERS-CoV) and Severe Acute Respiratory Syndrome (SARS-CoV).

An outbreak of the CoV disease (COVID-19) caused by SARS-CoV-2 began in Wuhan, Hubei Province, China in December 2019 and has spread throughout China and to over 216 other countries and territories, including the United States (WHO 2020). On 11 March 2020, the World Health Organization (WHO) officially declared COVID-19 a pandemic. As of 28 May 2020, the WHO reported more than 5,593,631 confirmed cases and 353,334 deaths globally and the US Centers for Disease Control and Prevention (CDC) reported 1,698,523 confirmed and probable cases of COVID-19, with 100,446 deaths in the United States (CDC 2020a). The CDC have reported that the highest risk of disease burden is in older adults (≥ 65 years old) and people of any age who have serious underlying medical conditions, such as chronic lung disease or moderate to severe asthma; serious heart conditions; severe obesity; diabetes; chronic kidney disease requiring dialysis; liver disease; and those who are immunocompromised (CDC 2020b).

There is currently no vaccine against SARS-CoV-2. Global efforts to evaluate novel antivirals and therapeutic strategies to treat severe SARS-CoV-2 infections have intensified, but no proven therapeutic currently exists. Therefore, there is an urgent public health need for rapid development of novel interventions to prevent the spread of this disease. The primary goal of this Phase 3 study is to evaluate the vaccine efficacy (VE) of mRNA-1273 to prevent COVID-19, compared to placebo.

2.2. Background and Overview

ModernaTX, Inc. (the Sponsor) has developed a rapid-response, proprietary vaccine platform based on a messenger RNA (mRNA) delivery system. The platform is based on the principle and observations that cells in vivo can take up mRNA, translate it, and then express protein viral antigen(s) on the cell surface. The delivered mRNA does not enter the cellular nucleus or interact with the genome, is nonreplicating, and is expressed transiently. mRNA vaccines have been used to induce immune responses against infectious pathogens such as cytomegalovirus (CMV) (NCT03382405), human metapneumovirus (hMPV) and parainfluenza virus type 3 (PIV3) (NCT03392389), Zika virus (NCT03325075), and influenza virus (NCT03076385 and NCT03345043).

The Sponsor is using its mRNA-based platform to develop a novel lipid nanoparticle (LNP)-encapsulated mRNA-based vaccine against SARS-CoV-2 (mRNA-1273). mRNA-1273

encodes for the full-length spike (S) protein of SARS-CoV-2, modified to introduce 2 proline residues to stabilize the S protein (S2P) in a prefusion conformation. The CoV S protein mediates attachment and entry of the virus into host cells (by fusion), making it a primary target for neutralizing antibodies that prevent infection (Johnson et al 2016; Wang et al 2015; Wang et al 2018; Chen et al 2017; Corti et al 2015; Yu et al 2015; Kim et al 2019; Widjaja et al 2019). It has been confirmed that the stabilized SARS-CoV-2 S2P antigen presents in the correct prefusion conformation (Wrapp et al 2020).

The development of the mRNA-1273 vaccine is being accelerated to address the current SARS-CoV-2 outbreak as a result of the uniquely rapid and scalable manufacturing process for mRNA-1273. The primary goal of this Phase 3 study is to evaluate the VE of mRNA-1273 to prevent COVID-19, compared to placebo.

2.2.1. Nonclinical Studies

Nonclinical studies have demonstrated that CoV S proteins are immunogenic and S protein-based vaccines, including those based on mRNA delivery platforms, are protective in animals. Prior clinical studies of vaccines targeting related CoVs and other viruses have demonstrated that mRNA-based vaccines are safe and immunogenic. It is therefore anticipated that mRNA-1273 will generate robust immune responses to the SARS-CoV-2 S protein and will be well tolerated. In addition, mRNA-1273 has shown preliminary evidence of protection against SARS-CoV-2 in a murine model of infection (data on file).

In support of development of mRNA-1273 for prophylaxis against SARS-CoV-2 infection, nonclinical immunogenicity, biodistribution, and safety studies have been completed with similar mRNA-based vaccines formulated in LNPs containing SM-102 (heptadecan-9-yl 8 ((2 hydroxyethyl)(6 oxo 6-(undecyloxy)hexyl)amino)octanoate), the novel proprietary lipid used in the mRNA-1273 LNP formulation.

A detailed review of nonclinical experience with mRNA-1273 vaccine is provided in the investigator's brochure (IB).

2.2.2. Clinical Studies

The mRNA-1273 vaccine is currently being evaluated for safety and immunogenicity in a dose ranging Phase 1 study (NCT04283461) sponsored and conducted by the Division of Microbiology and Infectious Diseases (DMID) of the National Institute of Allergy and Infectious Diseases (NIAID). The Phase 1 DMID study is an open-label dose ranging study of mRNA-1273 in healthy adult male and non-pregnant female participants in 3 age groups: age 18 to 55 years, inclusive (45 participants); age 56 to 70 years, inclusive (30 participants); and ≥ 71 years (30 participants). Participants in each cohort are randomly assigned to 1 of 3 dose levels of mRNA-1273: 25 µg,

100 µg, and 250 µg. Each participant will receive an IM injection (0.5 mL) of mRNA-1273 on Days 1 and 29 in the deltoid muscle and will be followed for 13 months after the second injection.

As of 14 May 2020, 15 participants in each of the 3 dose levels of the 18 to 55-year age cohort had received at least 1 dose of mRNA-1273. Recruitment of participants in the 2 older-age cohorts is ongoing. There have been no SAEs reported and no triggering of study pause rules. A review of preliminary solicited local and systemic ARs in participants in the 18 to 55-year age cohort after the second injection showed 3 participants in the 100 µg dose group who reported severe local ARs (grade 3 erythema and induration) and 3 participants in the 250 µg dose group who reported severe systemic ARs (fever, fatigue, feverishness, myalgia, and nausea). These adverse reactions resolved within 24 hours and were not assessed as serious.

Additionally, a dose-finding Phase 2a study (mRNA-1273-P201) conducted by the Sponsor under IND 19745 will expand the safety and immunogenicity database by testing 2 two dose levels (50 µg and 100 µg) in 400 adults.

As of January 6, 2020, approximately 365 participants were dosed with either an SM-102-containing lipid vaccine or placebo (doses ranging from 10 to 300 µg) across 3 Phase 1 studies for CMV, HMPV/PIV3, or Zika vaccines. There were no related serious adverse events (SAEs), and the vaccines appeared to be generally well tolerated. The results of 2 Phase 1 dose-ranging studies of an earlier formulation of the Sponsor's mRNA vaccine against H10N8 and H7N9 pandemic influenza was recently published. Both vaccines were immunogenic and well tolerated at doses up to 100 µg (NCT03076385 and NCT03345043; Feldman et al 2019). A detailed review of clinical experience with LNPs containing SM-102 (mRNA vaccines and placebo) is provided in the IB.

2.3. Benefit/Risk Assessment

2.3.1. Potential Benefits of Study Participation

The target study population for this study is adults with no known history of SARS-CoV-2 infection but whose locations or circumstances put them at high risk of COVID-19. The following benefits may accrue to participants:

- The mRNA-1273 vaccine may be an effective vaccine against COVID-19.
- A baseline (Day 1) evaluation for SARS-CoV-2 infection and ongoing surveillance for COVID-19 throughout the study.
- Contributing to the development of a vaccine against COVID-19, a current pandemic disease.

2.3.2. Risks from Study Participation

Immediate systemic allergic reactions (eg, anaphylaxis) can occur following any vaccination. These reactions are very rare and are estimated to occur once per 450,000 vaccinations for vaccines that do not contain allergens such as gelatin or egg protein (Zent et al 2002). As a precaution, all participants will remain under observation at the study site for at least 30 minutes after vaccination.

Vasovagal syncope (fainting) can occur before or after any vaccination, is usually triggered by the pain or anxiety caused by the injection and is not related to the substance injected. Therefore, it is important that standard precautions and procedures be followed to avoid injury from fainting.

Intramuscular injection with other mRNA vaccines manufactured by the Sponsor containing the SM-102 lipid formulation commonly results in a transient and self-limiting local inflammatory reaction. This typically includes pain, erythema (redness), or swelling (hardness) at the injection site, which are mostly mild to moderate in severity and usually occur within 24 hours of vaccination. More severe, but self-limited, local reactions, erythema and induration, have been observed at dose of mRNA-1273 exceeding the dose proposed in this study.

Most systemic adverse events observed after vaccination do not exceed mild-to-moderate severity. The most commonly reported systemic adverse reactions (ARs) are anticipated to be fever, fatigue, chills, headache, myalgias and arthralgias. More severe reactions, including erythema, induration, fever, headache and nausea, were reported after receiving doses of mRNA-1273 that were greater than the dose proposed for use in this study. In all cases, the reactions resolved spontaneously.

Laboratory abnormalities (including increases in liver functional tests and serum lipase levels) following vaccination were observed in clinical studies with similar mRNA-based vaccines. These abnormalities were without clinical symptoms or signs and returned toward baseline (Day 1) values over time. The clinical significance of these observations is unknown. Further details are provided in the current IB.

There is a theoretical risk that active vaccination to prevent the novel viral infection caused by SARS-CoV-2 may cause a paradoxical increase in the risk of disease. This possibility is based on the rare phenomenon of vaccine-associated disease enhancement which was first seen in the 1960s with 2 vaccines made in the same way (formalin-inactivated whole virus) and designed to protect children against infection with RSV (Chin et al 1969) or measles (Fulginiti et al 1967). Disease enhancement has also been proposed as a possible explanation for cases of more serious disease associated with dengue vaccination (Thomas and Yoon 2019; WHO 2018). It is not known if mRNA-1273 will increase the risk of enhanced disease.

To monitor the risk of enhanced disease in this study, an independent Data and Safety Monitoring Board (DSMB) will review unblinded cases of COVID-19 to assess for inefficacy and also for

numerical imbalance in cases of both COVID-19 and severe COVID-19 with the purpose of providing a non-binding recommendation to the Sponsor (Section 8.4.2).

2.3.3. Overall Benefit/Risk Conclusion

All participants will be included based on their increased risk of SARS-CoV-2 infection. Accordingly, all will benefit from baseline and ongoing evaluations for SARS-CoV-2 infection.

Since this is a placebo-controlled study (Section 4), half the participants will have the potential to receive mRNA-1273 vaccine, the efficacy of which is unknown at present. Vaccination with mRNA-1273 may not prevent COVID-19 in all vaccinees.

Participants who receive placebo as part of this study may have an opportunity cost of not being treated with another investigational vaccine against COVID-19.

The placebo for this study is a saline solution, without any LNP. Thus, participants receiving saline may be at lower risk of adverse events (AEs) related to injection than participants receiving mRNA-1273.

Safety findings will be monitored and periodically reviewed by the DSMB to evaluate the safety and treatment status of all participants. The DSMB will review and assess the safety data as described in Section 8.4.2.

Considering the lack of approved vaccines for COVID-19, the participants' risk of COVID-19 outside the study, and the nonclinical and clinical data to date, the Sponsor considers the potential benefits of participation to exceed the risks.

3. OBJECTIVES AND ENDPOINTS

Table 1: Objectives and Endpoints

Objectives and Endpoints	
**Primary Objective**	**Primary Endpoints**
**Efficacy Objective (Primary):**<br>To demonstrate the efficacy of mRNA-1273 to prevent COVID-19.	**Efficacy Endpoints (Primary):** Vaccine efficacy of mRNA-1273 to prevent the first occurrence of COVID-19 starting 14 days after the second dose of investigational product (IP), where COVID-19 is defined as symptomatic disease based on the following criteria:<br>• The participant must have experienced at least TWO of the following systemic symptoms: Fever (≥ 38ºC), chills, myalgia, headache, sore throat, new olfactory and taste disorder(s), OR<br>• The participant must have experienced at least ONE of the following respiratory signs/symptoms: cough, shortness of breath or difficulty breathing, OR clinical or radiographical evidence of pneumonia; AND<br>• The participant must have at least one NP swab, nasal swab, or saliva sample positive for SARS-CoV-2 by RT-PCR.
**Safety Objective (Primary):**<br>To evaluate the safety and reactogenicity of 2 injections of the mRNA-1273 vaccine given 28 days apart.	**Safety Endpoint (Primary):**<br>• Solicited local and systemic ARs through 7 days after each dose of IP.<br>• Unsolicited AEs through 29 days after each dose of IP.<br>• Medically attended adverse events (MAAEs) or AEs leading to withdrawal through the entire study period.<br>• SAEs throughout the entire study period.<br>• Pregnancies and perinatal outcomes.

**Efficacy Objectives (Secondary)**	**Efficacy Endpoints (Secondary)**
To evaluate the efficacy of mRNA-1273 to prevent severe COVID-19.	• Vaccine efficacy of mRNA-1273 to prevent severe COVID19, defined as first occurrence of COVID-19 starting 14 days after the second dose of IP, (as per the primary endpoint) AND any of the following:<br>o Clinical signs indicative of severe systemic illness, Respiratory Rates ≥ 30 per minute, Heart Rate ≥ 125 beats per minute, $SpO_2 \leq 93\%$ on room air at sea level or $PaO_2/FIO_2 < 300$ mm Hg, OR<br>o Respiratory failure or Acute Respiratory Distress Syndrome (ARDS), (defined as needing high-flow oxygen, non-invasive or mechanical ventilation, or ECMO), evidence of shock (systolic blood pressure < 90 mmHg, diastolic BP < 60 mmHg or requiring vasopressors), OR<br>o Significant acute renal, hepatic or neurologic dysfunction, OR<br>o Admission to an intensive care unit or death.
To evaluate the efficacy of mRNA-1273 to prevent serologically confirmed SARS-CoV-2 infection or COVID-19 regardless of symptomatology or severity.	Vaccine efficacy of mRNA-1273 to prevent the first occurrence of either COVID-19 or SARS-CoV-2 infection starting 14 days after the second IP dose.<br>This endpoint is a combination of COVID-19, defined as for the primary endpoint, and asymptomatic SARS-CoV-2 infection, assessed by seroconversion and with a negative nasopharyngeal (NP) swab sample for SARS-CoV-2 at Day 1 (Section 8.1.1).
To evaluate VE against a secondary definition of COVID-19.	Vaccine efficacy of mRNA-1273 to prevent the secondary case definition of COVID-19 starting 14 days after the second IP dose.<br>The secondary case definition of COVID-19 is defined as the following systemic symptoms: fever (temperature ≥ 38ºC), or chills, cough, shortness of breath or difficulty breathing, fatigue, muscle aches or body aches, headache, new loss of taste or smell, sore throat, nasal congestion or rhinorrhea, nausea or vomiting, or diarrhea AND a positive NP swab, nasal swab, or saliva sample for SARS-CoV-2 by RT-PCR.

To evaluate VE to prevent death caused by COVID-19.	Vaccine efficacy of mRNA-1273 to prevent death due to a cause directly attributed to a complication of COVID-19, starting 14 days after the second IP dose.
To evaluate the efficacy of mRNA-1273 to prevent COVID-19 after the first dose of IP.	Vaccine efficacy of mRNA-1273 to prevent the first occurrence of COVID-19 starting 14 days after the first dose of IP.
To evaluate the efficacy of mRNA-1273 to prevent COVID-19 in all study participants, regardless of evidence of prior SARS-CoV-2 infection.	Vaccine efficacy of mRNA-1273 to prevent the first occurrence of COVID-19 starting 14 days after the second dose of IP regardless of evidence of prior SARS-CoV-2 infection determined by serologic titer against SARS-CoV-2 nucleocapsid (FAS analysis population, see Section 9.4).
To evaluate the efficacy of mRNA-1273 to prevent asymptomatic SARS-CoV-2 infection.	Vaccine efficacy to prevent the first occurrence of SARS-CoV-2 infection in the absence of symptoms defining COVID-19 starting 14 days after the second IP dose. SARS-CoV-2 infection assessed by seroconversion and with a negative NP swab sample for SARS-CoV-2 at Day 1 (Section 8.1.1).
**Immunogenicity Objective (Secondary):**	**Immunogenicity Endpoints (Secondary):**
To evaluate the immunogenicity of 2 doses of mRNA-1273 given 28 days apart.	• Geometric mean titer (GMT) of SARS-CoV-2-specific neutralizing antibody (nAb) on Day 1, Day 29, Day 57, Day 209, Day 394, and Day 759.<br>• Geometric mean fold rise (GMFR) of SARS-CoV-2-specific nAb relative to Day 1 on Day 29, Day 57, Day 209, Day 394, and Day 759.<br>• Quantified levels or GMT of S protein-specific binding antibody (bAb) on Day 1, Day 29, Day 57, Day 209, Day 394, and Day 759.<br>• GMFR of S protein-specific bAb relative to Day 1 on Day 29, Day 57, Day 209, Day 394, and Day 759.
**Exploratory Objectives**	
To evaluate the effect of mRNA-1273 on the viral infection kinetics as measured by viral load at SARS-CoV-2 infection diagnosis by RT-PCR and number of days from the estimated date of SARS-CoV-2 infection until undetectable SARS-CoV-2 infection by RT-PCR.	
To assess VE to reduce the duration of symptoms of COVID-19.	
To assess VE against burden of disease (BOD) due to COVID-19.	

To evaluate VE against all-cause mortality.
To evaluate the genetic and/or phenotypic relationships of isolated SARS-CoV-2 strains to the vaccine sequence.
To evaluate immune response markers after dosing with IP as correlates of risk of COVID-19 and as correlates of risk of SARS-CoV-2 infection.
To conduct additional analyses related to furthering the understanding of SARS-CoV-2 infection and COVID-19, including analyses related to the immunology of this or other vaccines, detection of viral infection, and clinical conduct.

Abbreviations: AE = adverse event; AR = adverse reaction; bAb = binding antibody; GMFR = geometric mean fold rise; GMT = geometric mean titer; ICU = intensive care unit; MAAE = medically attended adverse event; nAb = neutralizing antibody; NP = nasopharyngeal; SAE = serious adverse event; VE = vaccine efficacy.

4. STUDY DESIGN

4.1. General Design

This is a Phase 3, randomized, stratified, observer-blind, placebo-controlled study to evaluate the efficacy, safety, and immunogenicity of mRNA-1273 SARS-CoV-2 vaccine compared to placebo in adults 18 years of age and older who have no known history of SARS-CoV-2 infection but whose locations or circumstances put them at appreciable risk of acquiring COVID-19 and/or SARS-CoV-2 infection. Figure 1 shows the study flow and Appendix 1 (Section 11.1) displays the planned Schedules of Events (SoEs).

Up to approximately 30,000 participants will be randomly assigned to receive doses of either 100 µg of mRNA-1273 vaccine or a placebo control in a 1:1 randomization ratio. Assignment will be stratified by age and health risk (Section 6.2.1.1). This is a case-driven study and thus final study size will depend on the actual attack rate of COVID-19.

All participants will be assessed for efficacy and safety endpoints and provide a nasopharyngeal (NP) swab sample and blood sample before the first dose of IP in addition to a series of post-dose blood samples for immunogenicity through 24 months after the second dose of IP. Efficacy assessments will include surveillance for COVID-19 with RT-PCR confirmation of SARS-CoV-2 infection after the first dose of IP. As noted above, this is a case-driven study: if the prespecified criteria for early efficacy are met at the time of either interim analysis (IA) or overall efficacy at the primary analysis, a final study report describing the efficacy and safety of mRNA-1273 will be prepared based on the data available at that time. In the event that success criteria are met either at the time of the interim analyses or when the total number of cases toward the primary endpoint have accrued, participants will continue to be followed in a blinded fashion until Month 25, to enable assessment of long-term safety and durability of VE (Figure 2). If the study concludes early, all participants will be requested to provide a final blood sample at the time of study conclusion.

Figure 2: Event/Case-Driven Study with 24 Months of Planned Follow-Up

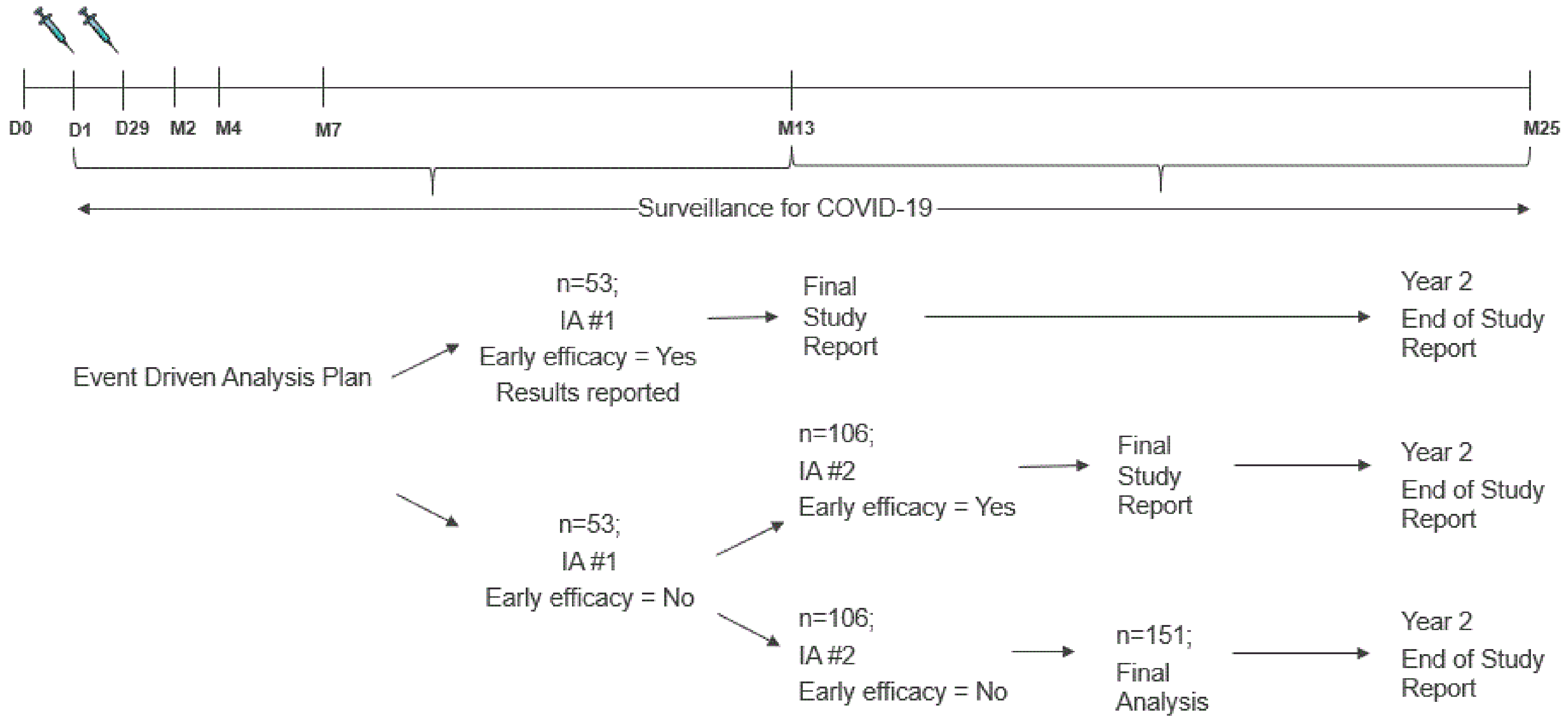

FA=final analysis; IA=interim analysis; n= number of cases

All participants may have up to 7 scheduled clinic visits, including Screening, Day 1, Day 29, Day 57, Day 209, Day 394 and Day 759 (Section 11.1). Each participant will receive 2 doses of IP by 0.5 mL intramuscular (IM) injection, the first on Day 1 and the second on Day 29. An NP swab sample will be collected prior to the first dose of IP, for evaluation by RT-PCR. To preserve observer blinding, only delegated unblinded study personnel responsible for study vaccine preparation, administration and/or accountability will have knowledge of study treatment assignment (Section 6.2.8.1).

Participants will be given an electronic diary (eDiary) to report solicited ARs for 7 days after each dose of IP and to prompt an unscheduled clinic visit for clinical evaluation and NP swab sample if a participant experiences any symptoms of COVID-19. Participants will use the eDiary to report solicited ARs for 7 days after each dose of IP and weekly eDiary prompts to elicit an unscheduled illness visit if the participant is experiencing COVID-19 symptoms. All participants will receive safety calls 7 days and 14 days after each dose (on Day 8, Day 15, Day 36, and Day 43) that will serve both to monitor AEs possibly related to IP dosing and to monitor for symptoms of COVID-19.

Safety telephone calls and eDiary safety prompts will be performed in conjunction with surveillance for COVID-19 according to the SoEs (Section 11.1) and are intended to capture SAEs, MAAEs, AEs leading to withdrawal, concomitant medications associated with these events, and receipt of non-study vaccinations (Section 8.2.1). Participants will also be asked to report if they have been exposed to anyone diagnosed with COVID-19. If an eDiary prompt results in identification of a relevant safety event, a follow-up safety call will be triggered.

Surveillance for COVID-19 will be performed through weekly contacts with the participant via a combination of telephone calls and completion of an eDiary starting at Day 1 through the end of the study (Section 11.1). Participants meeting clinical criteria for COVID-19 will return to the clinic or will be visited at home by medically qualified site staff within 72 hours to collect an NP swab sample for RT-PCR testing for SARS-CoV-2 and other respiratory pathogens, or alternatively, if a clinic or home visit is not possible, will submit a nasal swab or saliva sample for SARS-CoV-2 RT-PCR testing (Section 8.1.1).

Any confirmed COVID-19 occurring in a participant will be captured as an MAAE along with relevant concomitant medications and details about severity, seriousness, and outcome. Study participants will be monitored by medically qualified site personnel for a 28-day period after diagnosis. Each participant diagnosed with COVID-19 will record the presence of symptoms using a paper diary/eDiary for 28 days following the laboratory confirmation of COVID-19 (Table 16). In addition to daily recording of symptoms, assessments will include the collection of weekly samples (NP swab [preferred], nasal swab, or saliva) for 28 days following the diagnosis of SARS-

CoV-2 infection. Additionally, a convalescent visit will be scheduled approximately 28 days after diagnosis. At this visit, a blood sample will be collected for serologic assessment of SARS-CoV-2 infection.

At each dosing visit, participants will be instructed (Day 1) or reminded (Day 29) on how to document and report solicited ARs in the eDiary provided. Solicited ARs will be assessed for 7 days after each IP dose and unsolicited AEs will be assessed for 28 days after each IP dose; SAEs, MAAEs, AEs leading to withdrawal, will be assessed throughout the study.

Participants will have scheduled blood sampling (for immunogenicity assessment) at Screening, Day 1, Day 29, Day 57, Day 209, Day 394, and Day 759.

Participants may experience AEs that necessitate an unscheduled visit. There may also be situations in which the Investigator asks a participant to report for an unscheduled visit following the report of an AE. Additional examinations may be conducted at these visits as necessary to ensure the safety and well-being of participants during the study. Electronic case report forms (eCRFs) should be completed for each unscheduled visit.

This study will be conducted in compliance with the protocol, Good Clinical Practice (GCP), and all applicable regulatory requirements.

4.2. Scientific Rationale for Study Design

The mRNA-1273 vaccine is being developed to prevent COVID-19, the disease resulting from SARS-CoV-2 infection. The study is designed to directly evaluate the clinical efficacy and safety of mRNA-1273 to prevent COVID-19 for up to 2 years after the second dose of mRNA-1273.

The immunogenicity endpoints and detection of SARS-CoV-2 infection are secondary objectives.

This design and focus are dependent on the current COVID-19 pandemic, requiring identification of participant candidates at high risk of SARS-CoV-2 infection. The Sponsor may adjust the size of the study or duration of follow-up based on the blinded review of the total number of cases of COVID-19 accrued during the study, in addition to estimated percentages of study participants with serologic evidence of SARS-CoV-2 infection at baseline. If achieving 151 cases of COVID-19 is manifestly unattainable based on plausible expansions of sample size or increased follow-up, an analysis of blinded data may be performed and change of the study design such as changing lower bound or the endpoint may be proposed.

4.3. Choice of Dose and Control Product

The dose selected for this study (100 µg of mRNA-1273) is based on assessment of available safety and immunogenicity data from Phase 1 studies of mRNA-1647 (NCT03382405), the DMID study 20-0003 entitled "Phase I, Open-Label, Dose-Ranging Study of the Safety and Immunogenicity of

2019-nCoV Vaccine (mRNA-1273) in Healthy Adults" (NCT04283461) and the mRNA-1273-P201 study (Section 2.2.2). The schedule (2 doses administered 28 days apart) is being used in these studies.

There is no licensed SARS-CoV-2 vaccine currently available to serve as a reference control; accordingly, 0.9% sodium chloride (normal saline) injection (USP) will be used as a placebo control.

4.4. End of Study Definition

Participants are considered to have completed the study if they complete the final visit at Day 759 (Month 25), 24 months following the last dose of IP.

The study duration will be approximately 26 months for each participant. This includes a screening period of up to 1 month and a study period of 25 months that includes the first dose of IP on Day 1 and the second dose on Day 29. The participant's final scheduled visit will be on Day 759 (Month 25), 24 months after the second dose of IP on Day 29 (Month 1).

The end of study will be the final participant's final scheduled visit at Day 759 (Month 25).

5. STUDY POPULATION

The study population, adults at risk of SARS-CoV-2 infection who have no known history of SARS-CoV-2 infection, is a subset of the planned target population. Additionally, potential study participants at increased risk of complications from COVID-19 will be included since it is hypothesized that these participants might derive the greatest benefit from a vaccine. Study sites may be selected on the basis of SARS-COV-2 infection risk of the local population. Approximately 30,000 participants will be enrolled.

There will be no prospective approval of protocol deviations to recruitment and enrollment criteria (also known as protocol waivers or exemptions).

5.1. Inclusion Criteria

Participants are eligible to be included in the study only if all the following criteria apply:

1. Adults, ≥ 18 years of age at time of consent, who are at high risk of SARS-CoV-2 infection, defined as adults whose locations or circumstances put them at appreciable risk of exposure to SARS-CoV-2 and COVID-19.
2. Understands and agrees to comply with the study procedures and provides written informed consent.
3. Able to comply with study procedures based on the assessment of the Investigator.
4. Female participants of nonchildbearing potential may be enrolled in the study. Nonchildbearing potential is defined as surgically sterile (history of bilateral tubal ligation, bilateral oophorectomy, hysterectomy) or postmenopausal (defined as amenorrhea for ≥ 12 consecutive months prior to Screening without an alternative medical cause). A follicle-stimulating hormone (FSH) level may be measured at the discretion of the investigator to confirm postmenopausal status.
5. Female participants of childbearing potential may be enrolled in the study if the participant fulfills all the following criteria:
   - Has a negative pregnancy test at Screening and on the day of the first dose (Day 1).
   - Has practiced adequate contraception or has abstained from all activities that could result in pregnancy for at least 28 days prior to the first dose (Day 1).
   - Has agreed to continue adequate contraception through 3 months following the second dose (Day 29).
   - Is not currently breastfeeding.

Adequate female contraception is defined as consistent and correct use of a Food and Drug Administration (FDA) approved contraceptive method in accordance with the product label. For example:

- Barrier method (such as condoms, diaphragm, or cervical cap) used in conjunction with spermicide
- Intrauterine device
- Prescription hormonal contraceptive taken or administered via oral (pill), transdermal (patch), subdermal, or IM route
- Sterilization of a female participant's monogamous male partner prior to entry into the study

Note: periodic abstinence (eg, calendar, ovulation, symptothermal, post-ovulation methods) and withdrawal are not acceptable methods of contraception.

6. Male participants engaging in activity that could result in pregnancy of sexual partners must agree to practice adequate contraception and refrain from sperm donation from the time of the first dose and through 3 months after the second dose.
   Adequate contraception for male participants is defined as:

   - Monogamous relationship with a female partner using an intrauterine device or hormonal contraception (described above)
   - Use of barrier methods and spermicide
   - History of surgical sterilization

   Male participants with partners who have become pregnant prior to Screening are eligible to participate in the study.

7. Healthy adults or adults with pre-existing medical conditions who are in stable condition. A stable medical condition is defined as disease not requiring significant change in therapy or hospitalization for worsening disease during the 3 months before enrollment.

5.2. Exclusion Criteria

Participants are excluded from the study if any of the following criteria apply:

1. Is acutely ill or febrile 72 hours prior to or at Screening. Fever is defined as a body temperature ≥ 38.0°C/100.4°F. Participants meeting this criterion may be rescheduled within the relevant window periods. Afebrile participants with minor illnesses can be enrolled at the discretion of the investigator.

2. Is pregnant or breastfeeding.
3. Known history of SARS-CoV-2 infection.
4. Prior administration of an investigational coronavirus (SARS-CoV, MERS-CoV) vaccine or current/planned simultaneous participation in another interventional study to prevent or treat COVID-19.
5. Demonstrated inability to comply with the study procedures.
6. An immediate family member or household member of this study's personnel.
7. History of anaphylaxis, urticaria, or other significant adverse reaction requiring medical intervention after receipt of a vaccine.
8. Bleeding disorder considered a contraindication to intramuscular injection or phlebotomy.
9. Has received or plans to receive a vaccine within 28 days prior to the first dose (Day 1) or plans to receive a non-study vaccine within 28 days prior to or after any dose of IP (except for seasonal influenza vaccine, see Section 6.4.1).
10. Has participated in an interventional clinical study within 28 days prior to the day of enrollment.
11. Current or previous diagnosis of immunocompromising condition, immune-mediated disease, or other immunosuppressive condition.
12. Has received systemic immunosuppressants or immune-modifying drugs for >14 days in total within 6 months prior to Screening (for corticosteroids ≥ 20 mg/day of prednisone equivalent). Topical tacrolimus is allowed if not used within 14 days prior to Screening.
13. Has received systemic immunoglobulins or blood products within 3 months prior to the day of screening.
14. Has donated ≥ 450 mL of blood products within 28 days prior to Screening.

5.3. Participant Restrictions

Participants must not eat or drink anything hot or cold within 10 minutes before oral temperature is taken.

5.4. Screen Failures

Screen failures are defined as participants who consent to participate in the clinical study but are not subsequently randomly assigned to treatment. A minimum set of screen failure information is required to ensure transparent reporting of screen failures to meet the Consolidated Standards of

Reporting Trials (CONSORT) publishing requirements and to respond to queries from regulatory authorities. Minimum information includes date of informed consent, demography, screen failure details, eligibility criteria, and information on any SAE that may have occurred from the time informed consent was obtained to the time of withdrawal.

Participants meeting the exclusion criterion #1, acutely ill or febrile prior to or at the Screening Visit (exclusion criterion #1, Section 5.2), may be rescheduled within the relevant window periods and will retain their initially assigned participant number.

6. STUDY TREATMENT

6.1. Investigational Product

The term "investigational product" refers to both mRNA-1273 vaccine and placebo administered in this study.

The mRNA-1273 IP is an LNP dispersion of an mRNA encoding the prefusion stabilized S protein of SARS-CoV-2 formulated in LNPs composed of 4 lipids (1 proprietary and 3 commercially available): the proprietary ionizable lipid SM-102; cholesterol; 1,2-distearoyl-sn-glycero-3-phosphocholine (DSPC); and 1-monomethoxypolyethyleneglycol-2,3-dimyristylglycerol with polyethylene glycol of average molecular weight 2000 (PEG2000-DMG). The mRNA-1273 is provided as a sterile liquid for injection and is a white to off white dispersion in appearance, at a concentration of 0.2 mg/mL in 20 mM Tris buffer containing 87 mg/mL sucrose and 10.7 mM sodium acetate at pH 7.5.

The placebo is 0.9% sodium chloride (normal saline) injection, which meets the criteria of the United States Pharmacopeia (USP).

6.2. Dosing and Management of Investigational Product

6.2.1. Method of Randomly Assigning Participants to Treatment Groups

Up to approximately 30,000 participants will be randomly assigned in 1:1 ratio to receive either mRNA-1273 100 µg or placebo. The randomization will be in a blinded manner using a centralized Interactive Response Technology (IRT) at the Day 1 visit, in accordance with pre-generated randomization schedules. Only the Unblinded personnel (Section 6.2.8.1) will have controlled access to which arm the participant is randomly assigned.

Dose group assignment in is summarized in Table 2.

Table 2: Summary of Treatment Groups

Treatment Groups	Investigational Product	Age (years)	Estimated Total Participants
mRNA-1273	mRNA-1273 100 µg	≥ 18	15,000
Placebo	Placebo	≥ 18	15,000
**Total**			**30,000**

6.2.1.1. Stratification

Randomization will be stratified based on age and, if they are < 65 years of age, based on the presence or absence of risk factors for severe illness from COVID-19 based on current CDC recommendation (CDC 2020b). There will be 3 strata for randomization: ≥ 65 years, < 65 years and categorized to be at increased risk (“at risk”) for the complications of COVID-19, and < 65 years “not at risk.” Risk will be defined based on the study participants’ relevant past and current medical history. At least 25% of enrolled participants, but not to exceed 40%, will be either ≥ 65 years of age or < 65 years of age and at risk at Screening.

Participants who are < 65 years old will be categorized as at risk for severe COVID-19 illness if they have at least 1 of the following risk factors at Screening:

- Chronic lung disease (eg, emphysema and chronic bronchitis), idiopathic pulmonary fibrosis and cystic fibrosis) or moderate to severe asthma
- Significant cardiac disease (eg, heart failure, coronary artery disease, congenital heart disease, cardiomyopathies, and pulmonary hypertension)
- Severe obesity (body mass index ≥ 40 kg/m^2)
- Diabetes (Type 1, Type 2 or gestational)
- Liver disease

Thus, there will be 3 strata for randomization: ≥ 65 years, < 65 years at risk, and < 65 years not at risk.

6.2.2. Administration of Investigational Product

Investigational product will be administered as an IM injection into the deltoid muscle on a 2-dose injection schedule on Day 1 and Day 29, with at least a 28-day interval between doses. Each injection will have a volume of 0.5 mL and contain mRNA-1273 100 µg or saline placebo. Preferably, vaccine should be administered into the nondominant arm. The second dose of IP should be administered in the same arm as the first dose.

The IP will be prepared for injection as a single 0.5 mL dose for each participant based on the randomization assignment, as detailed in the Pharmacy Manual. Unblinded personnel who will not participate in any other aspect of the study, will perform IP accountability, dose preparation, and IP administration. The investigator will designate unblinded medically qualified personnel (not involved in assessments of study endpoints) to administer the IP according to the procedures stipulated in this study protocol and Pharmacy Manual. Study-specific training will be provided.

At each visit when IP is administered, participants will be monitored for a minimum of 30 minutes after administration. Assessments will include vital sign measurements and monitoring for local or systemic reactions (Table 13).

Eligibility for a subsequent dose of IP is determined by following the criteria outlined in Section 7.

The study site will be appropriately staffed with individuals with basic CPR training/certification. Either on site resuscitation equipment and personnel or appropriate protocols for the rapid transport of subject to a resuscitation area/facility are required.

6.2.3. Delivery and Receipt

The Sponsor or designee is responsible for the following:

- Supplying the IP
- Confirming the appropriate labeling of mRNA-1273 IP, so that it complies with the legal requirements of the US

The investigator is responsible for acknowledging the receipt of the IP by a designated staff member at the site, including the following:

- Confirming that the IP was received in good condition
- Confirmation that the temperature during shipment from the Sponsor to the investigator's designated storage location was appropriate
- Confirming whether the Sponsor has authorized the IP for use
- Ensuring the appropriate dose level of mRNA-1273 is properly prepared using aseptic technique

Further description of the IP and instructions for the receipt, storage, preparation, administration, accountability, and destruction of the IP are described in the mRNA-1273-P201 Pharmacy Manual.

6.2.4. Packaging and Labeling

The Sponsor will provide the investigator and study site with adequate quantities of mRNA-1273. The sterile vaccine product is packaged in a 10R glass vial with a 5.0-mL fill volume. mRNA-1273 vaccine will have all required labeling per regulations and will be supplied to the pharmacy in an unblinded manner. Each vial will be individually labeled for future participant identification purposes.

mRNA-1273 will be packaged and labeled in accordance with the standard operating procedures (SOPs) of the Sponsor or of its designee, Code of Federal Regulations Title 21 (CFR), Good

Manufacturing Practice (GMP) guidelines, International Council for Harmonisation (ICH) GCP guidelines, guidelines for Quality System Regulations, and applicable regulations.

The Sponsor or Sponsor's designee will supply the 0.9% sodium chloride injection for use as both a placebo and a diluent to mRNA-1273. The 0.9% sodium chloride bears a commercial label and does not contain study-specific identification.

6.2.5. Storage

mRNA-1273 vaccine must be stored at 2°C to 8°C in a secure area with limited access (unblinded personnel only) and protected from moisture and light until it is prepared for administration (Section 6.2.2). The refrigerator should have automated temperature recording and a 24-hour alert system in place that allows for rapid response in case of refrigerator malfunction. There must be an available backup refrigerator. The refrigerators must be connected to a backup generator. In addition, vaccine accountability study staff (eg, the unblinded personnel) are required to keep a temperature log to establish a record of compliance with these storage conditions. The site is responsible for reporting any mRNA-1273 vaccine that was not temperature controlled during shipment or during storage to the unblinded site monitor. Such mRNA-1273 will be retained for inspection by the unblinded monitor and disposed of according to approved methods.

The 0.9% sodium chloride injection (USP) should be stored at 20ºC to 25ºC (68ºF to 77ºF) in a restricted access area.

6.2.6. Investigational Product Accountability

It is the investigator's responsibility that the unblinded personnel maintain accurate records in an IP accountability log of receipt of all IP, inventory at the site, dispensing of mRNA-1273 and placebo, IP injections, and return to the Sponsor or alternative disposition of used/unused products.

An unblinded site monitor will review the inventory and accountability log during site visits and at the completion of the study. Additional details are found in the mRNA-1273-P301 Pharmacy Manual.

6.2.7. Handling and Disposal

An unblinded site monitor will reconcile the IP during the conduct and at the end of the study for compliance. Once fully reconciled at the site at the end of the study, the IP can be destroyed at the investigational site or at a Sponsor-selected third party, as appropriate.

Investigational product may be destroyed at the study site only if permitted by local regulations and authorized by the Sponsor. A Certificate of Destruction must be completed and sent to the Sponsor or designee.

6.2.8. Unblinding

6.2.8.1. Planned Unblinding

This is an observer-blind study. The investigator, study staff, study participants, site monitors, and Sponsor personnel (or its designees) will be blinded to the IP administered until study end, with the following exceptions:

- Unblinded personnel (of limited number) will be assigned to vaccine accountability procedures and will prepare IP for all participants. These personnel will have no study functions other than study vaccine management, documentation, accountability, preparation, and administration. They will not be involved in participant evaluations and will not reveal the identity of IP to either the participant or the blinded study site personnel involved in the conduct of the study unless this information is necessary in the case of an emergency.
- Unblinded medically qualified study site personnel will administer the IP. They will not be involved in assessments of any study endpoints.
- Unblinded site monitors, not involved in other aspects of monitoring, will be assigned as the IP accountability monitors. They will have responsibilities to ensure that sites are following all proper IP accountability, preparation, and administration procedures.
- An unblinded statistical and programming team will perform the pre-planned IAs (Section 9.6).
- An independent DSMB will review the interim data to safeguard the interests of clinical study participants and to help ensure the integrity of the study. The DSMB will review unblinded statistical outputs and IA results, provided by the independent unblinded statistician, and make recommendations to the Sponsor (Section 8.4.2).
- If prespecified criteria for early efficacy are met by an IA or if the primary efficacy analysis is completed based on accrual of prespecified COVID-19 cases, pre-identified Sponsor and CRO team members responsible for the analysis and reporting will be unblinded to treatment assignments in order to prepare a final study report. In order to maintain an observer-blind design, investigators, site staff, participants, and Sponsor and CRO staff with oversight of study conduct will remain blinded to treatment allocation for the study duration. All study participants will be followed for efficacy and safety endpoints through the remainder of planned study period and results will be summarized in an end of study report (Sections 4.1 and 9.1).

The dosing assignment will be concealed by having the unblinded personnel prepare the IP in a secure location that is not accessible or visible to other study staff. An opaque sleeve over the syringe used for injection will maintain the blind at the time of injection, as the doses containing mRNA-1273 will look different than placebo. Only delegated unblinded site staff will conduct the injection procedure. Once the injection is completed, only the blinded study staff will perform further assessments and interact with the participants. Access to the randomization code will be strictly controlled at the pharmacy.

6.2.8.2. Unplanned Unblinding

A participant or participants may be unblinded in the event of an SAE or other severe event, or if there is a medical emergency requiring the identity of the product to be known to properly treat a participant. If a participant becomes seriously ill or pregnant during the study, the study investigator may request that the blind will be broken if knowledge of the administered vaccine will affect that participant's dosing options. In this situation or in the event of a medical emergency requiring identification of the IP administered to an individual participant, the investigator will make every attempt to contact the Sponsor medical lead to explain the need for opening the code within 24 hours of opening the code. The investigator will be responsible for documenting the time, date, reason for the code break, and the names of the personnel involved.

In addition to the aforementioned situations where the blind may be broken, the data will also be unblinded to a statistical team at specified time points for IAs as outlined in Section 9.6.

6.3. Study Treatment Compliance

All doses of IP will be administered at the study site under direct observation of unblinded medically qualified study personnel and appropriately recorded (date and time) in the eCRF. Unblinded personnel will confirm that the participant has received the entire dose of vaccine. If a participant does not receive vaccine or does not receive all of the planned doses, the reason for the missed dose will be recorded. Data will be reconciled with site accountability records to assess compliance.

Participants who miss the second dose of IP due to noncompliance with the visit schedule and not due to a safety pause will still be required to follow the original visit and testing schedule as described in the protocol. Unless consent is withdrawn, a participant who withdraws or is withheld from receiving the second dose of study vaccine will remain in the study and complete all efficacy, safety and immunogenicity assessments required through the participant's scheduled end of study.

The study site is responsible for ensuring that participants comply with the study windows allowed. If a participant misses a visit, every effort should be made to contact the participant and complete a visit within the defined visit window (SoE Tables, Section 11.1). If a participant does not

complete a visit within the time window, that visit will be classified as a missed visit and the participant will continue with subsequent scheduled study visits. All safety requirements of the missed visit will be captured and included in the subsequent visit (eg, clinical laboratory testing, eDiary review for reactogenicity, immunologic testing, as applicable).

6.4. Prior and Concomitant Therapy

6.4.1. Prior Medications and Therapies

Information about prior medications (including any prescription or over-the-counter medications, vaccines, or blood products) taken by the participant within the 28 days before providing informed consent (or as designated in the inclusion/exclusion requirements) will be recorded in the participant's eCRF.

6.4.2. Concomitant Medications and Therapies

At each study visit, study site staff must question the participant regarding any medications taken and vaccinations received by the participant and record the following information in the eCRF:

- All non-study vaccinations administered within the period starting 28 days before the first dose of IP.
- Seasonal influenza vaccine administered for the current influenza season (typically October through April in the Northern Hemisphere).
- All concomitant medications and non-study vaccinations taken through 28 days after each dose of IP. Antipyretics and analgesics taken prophylactically (ie, taken in the absence of any symptoms in anticipation of an injection reaction) will be recorded as such.
- Any concomitant medications used to prevent or treat COVID-19.
- Any concomitant medications relevant to or for the treatment of an SAE or a MAAE.
- Participant will be asked in the eDiary if they have taken any antipyretic or analgesic to treat or prevent fever or pain within 7 days after each IP injection, including the day of dosing. Reported antipyretic or analgesic medications should be recorded in the source document by the site staff during the post-injection study visits or via other participant interactions (eg, phone calls).

6.4.3. Concomitant Medications and Vaccines that May Lead to the Elimination of a Participant from Per-Protocol Analyses

The use of the following concomitant medications and/or vaccines will not require withdrawal of the participant from the study but may determine a participant's eligibility to receive a second dose or evaluability in the per-protocol analysis (Analysis Sets are described in Section 9.4):

- Any investigational or nonregistered product (drug or vaccine) other than the study vaccine used during the study period.
- A licensed vaccine (with the exception of seasonal influenza vaccine) administered during the period from 28 days before through 28 days after each dose of IP, except for any licensed influenza vaccine that was administered more than 14 days before or after any dose of IP.
- Immunoglobulins and/or any blood products administered during the study period (except for treatment of COVID-19).
- Medications that suppress the immune system (except for treatment of COVID-19).

If a participant takes a prohibited drug therapy, the investigator and the contract research organization's (CRO's) medical monitor will make a joint decision about continuing or withholding further dosing from the participant based on the time the medication was administered, the drug's pharmacology and pharmacokinetics, and whether use of the medication will compromise the participant's safety or the interpretation of data. It is the investigator's responsibility to ensure that details regarding the concomitant medications are adequately recorded in the eCRF.

All medication and interventions necessary for the appropriate care for the study participant, particularly to treat COVID-19, should be administered and appropriately documented along with the adverse event.

7. DELAYING OR DISCONTINUING STUDY TREAMENT AND PARTICIPANT WITHDRAWAL FROM THE STUDY

7.1. Criteria for Delay of Study Treatment

Body temperature must be measured at the Day 1 and Day 29 visits prior to any study treatment administration. The following events constitute criteria for delay of study treatment, and if either of these events occur at the time scheduled for dosing, the participant may be injected at a later date within the time window specified in Table 13, or the participant may be discontinued from dosing at the discretion of the investigator (Section 7.2):

- Acute moderate or severe infection with or without fever at the time of dosing
- Fever, defined as body temperature ≥ 38.0°C (100.4°F) at the time of dosing

Participants with a minor illness without fever, as assessed by the investigator, can be administered IP. Participants with a fever of 38.0°C (100.4°F) or higher will be contacted within the time window acceptable for participation and reevaluated for eligibility. If the Investigator determines that the participant's health on the day of administration temporarily precludes dosing with IP, the visit should be rescheduled within the allowed interval for that visit.

7.2. Discontinuation of Study Treatment

Every reasonable attempt will be made to follow up with participants for safety throughout the entire study period, even if further dosing is discontinued or the participant misses one or more visits. Unless consent is withdrawn, a participant who withdraws or is withheld from receiving the second dose of study vaccine will remain in the study and complete all scheduled visits and assessments. (Section 11.1).

The investigator, in consultation with the Sponsor's medical monitor, may withhold a participant from further dosing if the participant experiences any of the following:

- Becomes pregnant (Section 8.3.5)
- Develops, during the course of the study, symptoms or conditions listed in the exclusion criteria
- Experiences an AE (other than reactogenicity) after dosing that is considered by the investigator to be related to IP (Section 8.3.4) and is of Grade 3 (severe) or greater intensity (Section 8.3.8)
- Experiences an AE or SAE that, in the judgment of the investigator, requires study IP withdrawal due to its nature, severity, or required treatment, regardless of the causal relationship to vaccine

- Experiences a clinically abnormal vital sign measurement or finding on physical examination, or general condition that, in the judgment of the investigator, requires IP withdrawal

The reason(s) for discontinuation from further dosing will be recorded in the eCRF.

Prior to receiving a second dose of IP, participants will be reassessed to ensure that they continue to meet eligibility requirements as outlined below.

The following events in a participant constitute absolute contraindications to any further dosing of the IP to that participant. If any of these events occur during the study, the participant must not receive additional doses of vaccine but will be encouraged to continue study participation for safety through 24 months following last dose.

- Diagnosed COVID-19. If COVID-19 is suspected, further administration of IP must be withheld until COVID-19 test results are available.
- Anaphylaxis or systemic hypersensitivity reaction following the administration of vaccine.
- Any SAE judged by investigator or Sponsor to be related to study vaccine.
- Any clinically significant medical condition that, in the opinion of the investigator, poses an additional risk to the participant if he/she continues to participate in the study.

The following events constitute contraindications to administration of study vaccine at certain points in time, and if any of these events occur at the time scheduled for dosing, the participant may be injected at a later date, within the time window specified in the Schedule of Events (Table 13, Table 14, Table 15, Table 16), or the participant may be withdrawn from dosing at the discretion of the investigator (Section 7.3):

- Acute moderate or severe infection with or without fever at the time of dosing
- Fever, defined as body temperature ≥ 38.0°C (100.4°F) at the time of dosing

Participants with a minor illness without fever, as assessed by the investigator, can be administered IP. Participants with a fever of 38.0°C (100.4°F) or higher will be contacted within the time window acceptable for participation and reevaluated for eligibility.

7.3. Participant Withdrawal from the Study

7.3.1. Participant Withdrawal

Participants who withdraw from the study are withdrawn will not be replaced. A “withdrawal” from the study refers to a situation wherein a participant does not return for the final visit planned in the protocol. The statistical management of participant withdrawals is discussed in Section 9.

Participants can withdraw consent and withdraw from the study at any time, for any reason, without prejudice to further treatment the participant may need to receive. The investigator will request that the study participant complete all study procedures pending at the time of withdrawal.

If participant desires to withdraw from the study because of an AE, the investigator will try to obtain agreement to follow up with the participant until the event is considered resolved or stable and will then complete the end of study electronic case report form (eCRF).

Information related to the withdrawal will be documented in the eCRF. The Investigator will document whether the decision to withdraw a participant from the study was made by the participant or by the Investigator, as well as which of the following possible reasons was responsible for withdrawal:

- AE (specify)
- SAE (specify)
- Death
- Lost to follow-up
- Physician decision (specify)
- Pregnancy
- Protocol deviation
- Study terminated by Sponsor
- Withdrawal of consent by participant (specify)
- Other (specify)

Participants who are withdrawn from the study because of AEs (including SAEs) must be clearly distinguished from participants who are withdrawn for other reasons. Investigators will follow up with participants who are withdrawn from the study as result of an SAE or AE until resolution of the event.

A participant withdrawing from the study may request destruction of any samples taken and not tested, and the investigator must document this in the site study records.

If the participant withdraws consent for disclosure of future information, the Sponsor may retain and continue to use any data collected before such a withdrawal of consent (see Section 11.2.10).

The Sponsor will continue to retain and use all research results that have already been collected for the study evaluation, unless the participant has requested destruction of these samples. All biological samples that have already been collected may be retained and analyzed at a later date (or as permitted by local regulations).

7.4. Lost to Follow-up

Participants will be considered lost to follow-up (LTFU) if they repeatedly fail to return for scheduled visits without stating an intention to withdraw consent and they cannot be contacted by the study site. The following actions must be taken if a participant fails to return to the clinic for a required study visit:

- The site must attempt to contact the participant and reschedule the missed visit as soon as possible, counsel the participant on the importance of maintaining the assigned visit schedule and ascertain whether the participant wishes to and/or should continue in the study.
- Before a participant is deemed lost to follow up, the investigator or designee must make every effort to regain contact with the participant (where possible, 3 telephone calls and, if necessary, a certified letter to the participant's last known mailing address or local equivalent methods). These contact attempts (eg, dates of telephone calls and registered letters) should be documented in the participant's medical record. A participant should not be considered LTFU until these efforts have been made.
- Should the participant continue to be unreachable, he/she will be considered to have withdrawn from the study.

8. STUDY ASSESSMENTS AND PROCEDURES

Before performing any study procedures, all potential participants will sign an informed consent form (ICF) (as detailed in Section 11.2.6). Participants will undergo study procedures at the time points specified in the SoEs (Section 11.1).

A participant also can be seen for an unscheduled visit at any time during the study. An unscheduled visit may be prompted by reactogenicity issues, illness visit criteria for COVID-19, or new or ongoing AEs. The site also has the discretion to make reminder telephone calls or send text messages to inform the participant about visits, review paper or eDiary requirements, or follow up on ongoing or outstanding issues.

In accordance with "FDA Guidance on Conduct of Clinical Trials of Medical Products during the COVID-19 Pandemic" (DHHS 2020), investigators may convert study site visits to telemedicine visits with the approval of the Sponsor. Such action should be taken to protect the safety and well-being of study participants and study site staff or to comply with state or municipal mandates.

General considerations for study assessments and procedures include the following:

- Protocol waivers or exemptions are not allowed. The study procedures and their timing must be followed as presented in Section 11.1. Adherence to the study design requirements is essential and required for study conduct.
- Immediate safety concerns should be discussed with the Sponsor immediately upon occurrence or awareness to determine if the participant should continue study treatment or participation in the study.
- All screening evaluations must be completed and reviewed to confirm that potential participants meet all eligibility criteria. The Investigator will maintain a screening log to record details of all participants screened and to confirm eligibility or record reasons for screening failure, as applicable.
- Procedures conducted as part of the participant's routine clinical management (eg, blood count) and obtained before signing of the ICF may be utilized for screening or baseline purposes provided the procedures met the protocol-specified criteria and were performed within the time frame defined in the SoE.

8.1. Efficacy and Immunogenicity Assessments and Procedures

8.1.1. Efficacy Assessments Related to COVID-19 and SARS-CoV-2 Infection

Each study participant will have an NP swab sample collected for SARS-CoV-2 testing by RT-PCR on Day 1, prior to receiving a dose of the IP as specified in SoE (Table 13).

COVID-19:

To be considered as a case of COVID-19 for the evaluation of the Primary Efficacy Endpoint, the following case definition must be met:

- The participant must have experienced at least TWO of the following systemic symptoms: Fever (≥ 38ºC), chills, myalgia, headache, sore throat, new olfactory and taste disorder(s), OR
- The participant must have experienced at least ONE of the following respiratory signs/symptoms: cough, shortness of breath or difficulty breathing, OR clinical or radiographical evidence of pneumonia; AND
- The participant must have at least one NP swab, nasal swab, or saliva sample positive for SARS-CoV-2 by RT-PCR.

Severe COVID-19:

To be considered severe COVID-19, the following criteria must be met:

- Confirmed COVID-19 as per the Primary Efficacy Endpoint case definition, plus any of the following:
  - Clinical signs indicative of severe systemic illness, Respiratory Rates ≥ 30 per minute, Heart Rate ≥ 125 beats per minute, SpO_2 ≤ 93% on room air at sea level or PaO_2/FIO_2 < 300 mm Hg, OR
  - Respiratory failure or Acute Respiratory Distress Syndrome (ARDS), (defined as needing high-flow oxygen, non-invasive or mechanical ventilation, or ECMO), evidence of shock (systolic blood pressure < 90 mmHg, diastolic BP < 60 mmHg or requiring vasopressors), OR
  - Significant acute renal, hepatic or neurologic dysfunction, OR
  - Admission to an intensive care unit or death.

The secondary case definition of COVID-19 is defined as the following systemic symptoms: fever (temperature >38ºC), or chills, cough, shortness of breath or difficulty breathing, fatigue, muscle aches, or body aches, headache, new loss of taste or smell, sore throat, nasal congestion or

rhinorrhea, nausea, or vomiting or diarrhea AND a positive NP swab, or nasal swab, or saliva sample for SARS-CoV-2 by RT-PCR.

Death attributed to COVID-19 is defined as any participant who dies during the study with a cause directly attributed to a complication of COVID-19.

SARS-CoV-2 Infection:

- SARS-CoV-2 infection is defined by seroconversion measured by bAb against SARS-CoV-2 nucleocapsid. Seroconversion is defined differently for participants seronegative at Baseline and seropositive at Baseline:
    - Participants seronegative at Baseline: bAb levels against SARS-CoV-2 nucleocapsid either from below the limit of detection (LOD) or lower limit of quantification (LLOQ) at Study Day 1 that increase to above or equal to LOD or LLOQ starting at Study Day 57 or later.
    - Participants seropositive at Baseline: bAb levels against SARS-CoV-2 nucleocapsid above the LOD or LLOQ at study Day 1 that increase by 4-fold or more in participants with pre-existing bAb starting at Study Day 57 or later.

8.1.2. Surveillance for COVID-19 Symptoms

Surveillance for COVID-19 symptoms will be conducted via weekly telephone calls or eDiary prompts as specified in Section 8.2.2; Figure 3 starting after enrollment and throughout the study. If there is no response to an eDiary prompt for 2 days, the site staff will contact the study participant by phone.

According to the CDC as of 10 June 2020 (CDC 2020c), patients with COVID-19 have reported a wide range of symptoms ranging from mild symptoms to severe illness. Throughout the study, to surveil for COVID-19, the following prespecified symptoms that meet the criteria for suspicion of COVID-19 will be elicited weekly from the participant:

- Fever (temperature ≥ 38ºC) or chills
- Cough
- Shortness of breath or difficulty breathing
- Fatigue
- Muscle or body aches
- Headache
- New loss of taste or smell

- Sore throat
- Congestion or runny nose
- Nausea or vomiting
- Diarrhea

Figure 3: Surveillance for COVID-19 Symptoms and the Corresponding Clinical Data Pathways

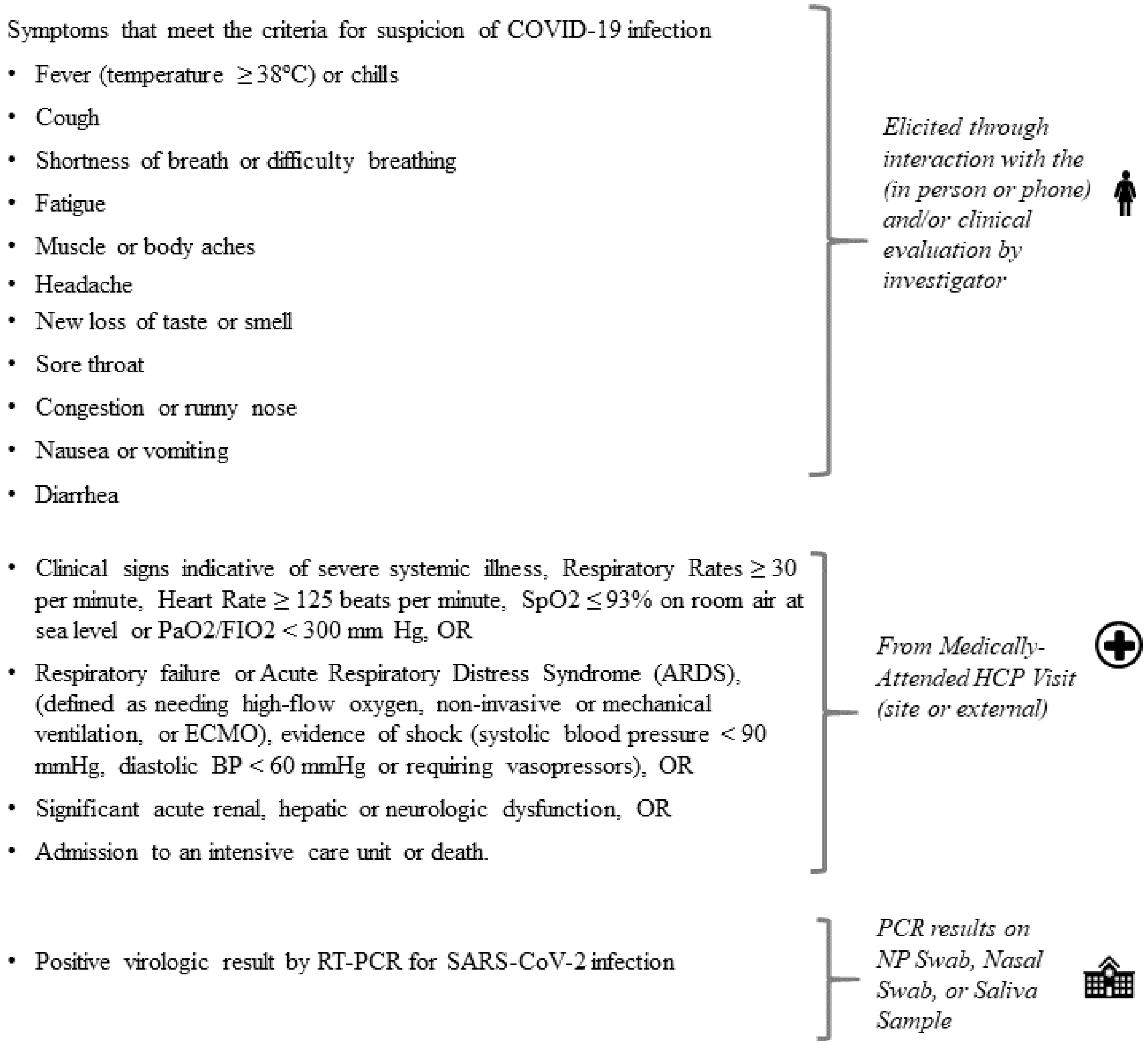

During the course of the study, participants meeting prespecified criteria of suspicion for COVID-19 will be asked to return within 72 hours or as soon as possible to the study site or medically qualified staff from the site will conduct a home visit as soon as possible to collect an NP swab sample (for RT-PCR), collect a blood sample for serologic analysis of SARS-CoV-2

infection, and to evaluate for COVID-19. The NP swab sample will also be tested for the presence of other respiratory infections.

In addition, the study site may collect an additional NP sample for SARS-CoV-2 testing to be able to render appropriate medical care for the study participant as determined by local standards of care. If neither a study site visit or home visit is possible, participants will be sent a nasal swab or saliva kit via courier or other Sponsor-approved method. Participant will deliver a nasal swab or saliva sample to the study site by courier or other Sponsor-approved method, and the sample will be tested by RT-PCR for SARS-CoV-2. If participants are confirmed to have SARS-CoV-2 infection, the investigator will notify the participant, and if applicable, the participant's primary care physician, of the diagnosis. The participant will also be instructed on infection prevention measures.

If scheduled, a study site illness visit may include assessments such as medical history, physical examination, blood sampling for clinical laboratory testing, and NP swab sampling for viral PCR (including multiplex PCR for respiratory viruses including SARS-CoV-2) to evaluate the severity of the clinical case. Radiologic imaging studies may be conducted. Blood samples will be collected for potential future serologic assessment of SARS-CoV-2 infection.

Cases are defined as participants meeting clinical criteria based both on symptoms for COVID-19 and on RT-PCR detection of SARS-CoV-2 from samples collected within 72 hours of the study participant reporting symptoms meeting the definition of COVID-19. Participants who are hospitalized for COVID-19 without the opportunity for a clinic or home visit or submission of nasal swab or saliva sample will also be considered cases, assuming that the symptomology criteria for COVID-19 are met and a respiratory sample is positive for SARS-CoV-2 by PCR at a CLIA certified lab. Investigators are encouraged to try to obtain a respiratory sample during the course of hospitalization for submission to the study central laboratory, if feasible. The investigator should determine if the criteria for severe COVID-19 has been met.

Evidence of severe COVID-19 is defined as in Section 8.1.1.

All clinical findings will be recorded in the eCRF.

8.1.3. Follow up/Convalescent Period After Diagnosis with COVID-19

Any confirmed COVID-19 occurring in a participant will be captured as an MAAE along with relevant concomitant medications and details about severity, seriousness, and outcome. Study participants will be monitored by medically qualified site personnel for a 28-day period after diagnosis. Each participant diagnosed with COVID-19 will record the presence of symptoms using a paper diary/eDiary for 28 days following the laboratory confirmation of COVID-19 (Table 16). Options for home visit or self-collection of specimens for RT-PCR will be provided to participants

to enhance collection of complete data. In addition to daily recording of symptoms, assessments will include the collection of weekly samples (NP swab [preferred], nasal swab, or saliva sample) for 28 days following the diagnosis of COVID-19. Additionally, a convalescent visit will be scheduled approximately 28 days after diagnosis. At this visit, a blood sample will be collected for serologic assessment of SARS-CoV-2 infection (Table 16). The investigator should determine if the criteria for severe COVID-19 has been met. If the participant is hospitalized, medically qualified site personnel will try to obtain medical records and SARS-CoV-2 diagnostic results. If the participant is later discharged from the hospital during the 28-day period following diagnosis of COVID-19, the study site personnel will arrange for a resumption of the schedule for weekly sampling and evaluation.

8.1.4. Ancillary Supplies for Participant Use

Study sites will distribute Sponsor-provided thermometers and rulers for use by participants in assessing body temperature and injection site reactions for recording solicited ARs in eDiaries (Section 8.2.2).

8.1.5. Immunogenicity Assessments

Blood samples for immunogenicity assessments will be collected at the time points indicated in the SoEs (Section 11.1). On Day 1 and Day 29, blood samples for immunogenicity assessment will be collected before administration of study treatment. The following analytes will be measured:

- Serum bAb levels against SARS-CoV-2 as measured by enzyme-linked immunosorbent assay (ELISA) specific to the SARS-CoV-2 S protein
- Serum bAb levels against SARS-CoV-2 as measured by ELISA specific to the SARS-CoV-2 nucleocapsid protein
- Serum nAb titer against SARS-CoV-2 as measured by pseudovirus and/or live virus neutralization assays

Serum will be tested using the ELISA specific to the SARS-CoV-2 nucleocapsid to determine the serologic status of study participants at baseline and assess for seroconversion during the course of the study. Serum from a subset of subjects will be tested in the other assays. The selection of the subset and timepoints to be tested will be described in the statistical analysis plan. Sample aliquots will be designed to ensure that backup samples are available and that adequate vial volumes may allow for further testing. The actual time and date of each sample collected will be recorded in the eCRF, and unique sample identification will be used to maintain the blind at the laboratory at all times and to allow for automated sample tracking and storage. Handling and

preparation of the samples for analysis, as well as shipping and storage requirements, will be provided in a separate study manual.

The ELISA and measurement of nAb titers will be performed in a laboratory designated by the Sponsor.

8.2. Safety Assessments

Safety assessments will include monitoring and recording of the following for each participant:

- Solicited local and systemic ARs (Section 8.3.4) that occur during the 7 days following each injection (ie, the day of dosing and 6 subsequent days). Solicited ARs will be recorded daily using eDiaries (Section 8.2.2).
- Unsolicited AEs observed or reported during the 28 days following each dose of IP (i.e, the day of dosing and 27 subsequent days). Unsolicited AEs are AEs that are not included in the protocol-defined solicited ARs (Section 8.3.4).
- AEs leading to discontinuation from dosing and/or study participation from Day 1 through Day 759 or withdrawal from the study.
- MAAEs from Day 1 through Day 759 or withdrawal from the study.
- SAEs from Day 1 through Day 759 or withdrawal from the study.
- Abnormal vital sign measurements.
- Physical examination findings.

8.2.1. Safety Phone Calls

A safety phone call is a telephone call made to the participant by medically qualified study staff. Medically qualified staff are those appropriately delegated individuals who are permitted to elicit verbal medical history from participants based on local regulations and local licensing requirements.

This call will follow a script, which will facilitate the collection of relevant safety information. The participant will be interviewed according to the script about occurrence of AEs, MAAEs, SAEs, or AEs leading to study withdrawal and concomitant medications associated with those events, as well as about occurrence of any non-study vaccinations. Occurrence of AEs will only be collected by safety phone call during the Vaccination Phase (Table 13).

The timing of the safety phone calls and the relevant safety information collected is provided in the SoEs (Section 11.1).

All safety information described by the participant must be documented in source documents and not documented on the script used for the safety telephone contact. All AEs, MAAEs, SAEs, and AEs leading to study withdrawal must be recorded in the eCRF as specified in Section 8.3.6.

8.2.2. Use of Electronic Diaries or Paper Diaries

At the time of consent, the participants must confirm they will be willing to complete a paper diary/eDiary (for 28-day COVID-19 symptoms) and an eDiary (for 7-day reactogenicity and to capture occurrence of AEs, MAAEs, SAEs and COVID-19 symptoms) for using either an application downloaded to their smartphone or using a device that is provided at the time of enrollment. This study will utilize the Medidata Patient Cloud Application as the eDiary for both collection of 7-day reactogenicity and weekly eDiary prompts to elicit an unscheduled illness visit if the participant is experiencing COVID-19 symptoms. This application allows for real-time data collection on a 21 CFR Part 11 compliant system directly from participants.

Before enrollment on Day 1, the participant will be instructed to download the eDiary application on their personal mobile device or will be provided an eDiary device to record solicited ARs (Section 8.3.4).

At each dosing visit, participants will record data into the eDiary starting approximately 30 minutes after dosing under supervision of the study site staff to ensure successful entry of assessments. The 30-minute assessment is an opportunity for site staff to train the participant. The site staff will perform any retraining as necessary. Study participants will continue to record data in an eDiary after they leave the study site, preferably in the evening and at the same time each day, on the day of dosing and for 6 days following dosing.

The following local ARs will be solicited by the eDiary: pain at injection site, erythema (redness) at injection site, swelling/induration (hardness) at injection site, and localized axillary swelling or tenderness ipsilateral to the injection arm.

The following systemic ARs will be solicited by the eDiary: headache, fatigue, myalgia (muscle aches all over the body), arthralgia (aching in several joints), nausea/vomiting, body temperature (potentially fever), and chills.

Solicited local and systemic reactogenicity ARs, as defined in Section 8.3.4, that occur on the day of each IP injection and during the 7 days after IP injection (ie, the day of dosing and 6 subsequent days). Any solicited AR that is ongoing beyond Day 7 will be reported in an eDiary until resolution. Adverse reactions recorded in diaries beyond Day 7 should be reviewed by study site staff either during the next scheduled phone call or at the next study site visit (Table 13).

In addition, between safety calls, a questionnaire will be triggered in the eDiary application, to capture the occurrence of AEs, MAAEs, SAEs, or AEs leading to study withdrawal.

Participants will record, in eDiary, the following data:

- Changes in health since the last time completing the questionnaire or has been in contact with the study clinic.
- Known exposure to someone with known SARS-CoV-2 infection or COVID-19.
- Capture of any experience of symptoms of COVID-19.
- Any MAAEs or SAEs.

If eDiary prompts result in identification of relevant safety events according to the study period or symptoms of COVID-19, a follow-up safety call will be triggered.

Participants will also be provided with a paper diary/eDiary at the time of enrollment on Day 1 to complete at the onset of COVID-19 symptoms. Participants will use this diary to record the daily entries of their symptoms during the 28 day follow-up after symptoms arise and confirmation of COVID-19 during the study (Table 16), continuing until resolution of all COVID-19 symptoms. Participants will confirm if they are experiencing symptoms or not and include daily assessments of their symptoms that are included in the COVID-19 case definition.

At each dosing visit, participants will be instructed (Day 1) or reminded (Day 29) on thermometer usage to measure body temperature, ruler usage to measure injection site erythema and swelling/induration (hardness), and self-assessment for localized axillary swelling or tenderness on the same side as the injection arm.

Daily oral body temperature measurement should be performed at approximately the same time each day using the thermometer provided by the study site.

8.2.3. Demographics/Medical History

Demographic information relating to the participant's sex, age, and race will be recorded at Screening on the appropriate eCRF page.

Additionally, information regarding participant occupational circumstances (eg, essential worker status) will be collected at Screening.

Medical history of each participant will be collected and recorded on the appropriate eCRF page. Significant findings that were present prior to the signature of the informed consent must be included in the Medical History eCRF page.

Study participants will also be asked to report history of receipt of seasonal influenza vaccine during the current influenza season (typically October through April in the Northern Hemisphere) as a concomitant medication.

8.2.4. Physical Examination

A full physical examination, including vital signs, height, and weight, will be performed at Screening and on Day 1, and symptom-directed physical examinations at other scheduled time points as indicated in the SoEs (Table 13, Table 14, Table 15, Table 16). The full examination will include assessment of skin, head, ears, eyes, nose, throat, neck, thyroid, lungs, heart, cardiovascular, abdomen, lymph nodes, and musculoskeletal system/extremities. Symptom-directed physical examinations may be performed at other timepoints at the discretion of the investigator.

On each dosing day before injection, the arm receiving the injection should be examined and the associated lymph nodes should be evaluated.

Body mass index will be calculated at the Screening Visit (Day 0) only.

Any clinically significant finding identified during a study visit after the first dose should be reported as a MAAE.

Significant new findings that begin or worsen after informed consent must be recorded on the AE eCRF page.

8.2.5. Vital Sign Measurements

Vital signs will be measured at the time points indicated in the SoEs (Table 13, Table 14, Table 15, Table 16). The participant will be seated for at least 5 minutes before all measurements are taken. On Day 1 and Day 29, vital sign measurements will be collected once before IP injection and at least 30 minutes after IP injection (before participants are discharged from the study site). When applicable, vital sign measurements should be performed before blood collection. When procedures overlap and are scheduled to occur at the same time point, the order of procedures should be vital sign measurements and then the blood collection.

Febrile participants at Day 1 and Day 29 visits (fever is defined as a body temperature ≥ 38.0°C/100.4°F) may be rescheduled within the relevant window periods. Criteria for delay of study treatment are provided in Section 7.1. Afebrile participants with minor illnesses may be injected at the discretion of the investigator.

If any of the vital sign measurements meet the toxicity grading criteria for clinical abnormalities of Grade 3 or greater, the abnormal value and Grade will be documented on the AE page of the eCRF (unless there is another known cause of the abnormality that would result in an AE classification). The investigator will continue to monitor the participant with additional assessments until the vital sign value has reached the reference range, returns to the vital sign value at baseline, is considered stable, or until the investigator determines that follow up is no longer medically necessary.

8.2.6. Blood Sampling Volumes

The maximum planned volumes of blood sampled per participant are 50 mL for 1 day, 100 mL for 28 days, and 300 mL for the complete study (Table 3).

Table 3: Maximum Planned Blood Sampling Volumes per Participant by Visit

Study Visit Day	D1	D29	D57	D209	D394	D759	Total
Immunogenicity blood samples	50 mL	50 mL	50 mL	50 mL	50 mL	50 mL	300 mL

Abbreviation: D = Day.

8.3. Safety Definitions and Procedures

8.3.1. Adverse Event

An AE is defined as any untoward medical occurrence associated with the use of a drug in humans, whether or not considered drug related.

A treatment-emergent AE (TEAE) is defined as any event not present before exposure to IP or any event already present that worsens in intensity or frequency after exposure.

Events Meeting the Adverse Event Definition

- Exacerbation of a chronic or intermittent pre-existing condition including either an increase in frequency and/or intensity of the condition.
- New conditions detected or diagnosed after the first dose of IP even though they may have been present before the start of the study.

Events NOT Meeting the Adverse Event Definition

- Medical or surgical procedure (eg, endoscopy, appendectomy): the condition that leads to the procedure should be the AE.
- Situations in which an untoward medical occurrence did not occur (social and/or convenience admission to a hospital).

An AR is any AE for which there is a reasonable possibility that the IP caused the AE (Section 8.3.4). For the purposes of investigational new drug safety reporting, “reasonable possibility” means that there is evidence to suggest a causal relationship between the IP and the AE.

An unsolicited AE is any AE reported by the participant that is not specified as a solicited AR in the protocol; is specified as a solicited AR in the protocol; or is specified as a solicited AR in the

protocol, but starts outside the protocol-defined period for reporting solicited ARs (ie, for the 7 days after each dose of IP).

8.3.2. Medically Attended Adverse Events

An MAAE is an AE that leads to an unscheduled visit to a healthcare practitioner (HCP). This would include visits to a study site for unscheduled assessments (eg, rash assessment, abnormal laboratory follow-up, SARS-CoV-2 infection [Section 8.1.1]) and visits to HCPs external to the study site (eg, urgent care, primary care physician). Investigators will review unsolicited AEs for the occurrence of any MAAEs. All MAAEs must be fully reported on the MAAE page of the eCRF.

8.3.3. Serious Adverse Events

An AE (including an AR) is considered an SAE if, in the view of either the investigator or Sponsor, it results in any of the following outcomes:

- **Death**
  A death that occurs during the study or that comes to the attention of the investigator during the protocol-defined follow-up period must be reported to the Sponsor, whether or not it is considered related to IP.
- **Is life-threatening**
  An AE is considered life-threatening if, in the view of either the investigator or the Sponsor, its occurrence places the participant at immediate risk of death. It does not include an AE that, had it occurred in a more severe form, might have caused death.
- **Inpatient hospitalization or prolongation of existing hospitalization**
  In general, inpatient hospitalization indicates the participant was admitted to the hospital or emergency ward for at least one overnight stay for observation and/or treatment that would not have been appropriate in the physician's office or outpatient setting. The hospital or emergency ward admission should be considered an SAE regardless of whether opinions differ as to the necessity of the admission. Complications that occur during inpatient hospitalization will be recorded as an AE; however, if a complication/AE prolongs hospitalization or otherwise fulfills SAE criteria, the complication/AE will be recorded as a separate SAE.
- **Persistent or significant incapacity or substantial disruption of the ability to conduct normal life functions**
  This definition is not intended to include experiences of relatively minor medical significance such as uncomplicated headache, nausea/vomiting, diarrhea, influenza,

and accidental trauma (eg, sprained ankle) which may interfere with or prevent everyday life functions but do not constitute a substantial disruption.

- **Congenital anomaly or birth defect**
- **Medically important event**
  Medical judgment should be exercised in deciding whether SAE reporting is appropriate in other situations, such as important medical events that may not be immediately life-threatening or result in death or hospitalization but may jeopardize the participant or require medical or surgical intervention to prevent one of the other outcomes listed in the above definition. These events should usually be considered serious. Examples of such medical events include allergic bronchospasm requiring intensive treatment in an emergency room or at home, blood dyscrasias or convulsions that do not result in inpatient hospitalization, or the development of drug dependency or drug abuse.

8.3.4. Solicited Adverse Reactions

The term "reactogenicity" refers to the occurrence and intensity of selected signs and symptoms (ARs) occurring after IP injection. The eDiary will solicit daily participant reporting of ARs using a structured checklist (Section 8.2.2). Participants will record such occurrences in an eDiary on the day of each IP injection and for the 6 days after the day of dosing.

The investigator will review, confirm, and Grade reactogenicity according to the grading scales presented in Table 4 modified from the Toxicity Grading Scale for Healthy Adult and Adolescent Volunteers Enrolled in Preventative Vaccine Clinical Trials (DHHS 2007).

If a solicited local or systemic AR continues beyond 7 days after dosing, the participant will be prompted daily to capture solicited local or systemic AR in the eDiary until resolution. Adverse reactions recorded in eDiaries beyond Day 7 should be reviewed either via phone call or at the following study visit. All solicited ARs (local and systemic) will be considered causally related to dosing.

Table 4: Solicited Adverse Reactions and Grades

Reaction	Grade 0	Grade 1	Grade 2	Grade 3	Grade 4*
Injection site pain	None	Does not interfere with activity	Repeated use of over-the-counter pain reliever > 24 hours or interferes with activity	Any use of prescription pain reliever or prevents daily activity	Requires emergency room visit or hospitalization
Injection site erythema (redness)	< 25 mm/ < 2.5 cm	25 - 50 mm/ 2.5 - 5 cm	51 - 100 mm/ 5.1 - 10 cm	> 100 mm/ > 10 cm	Necrosis or exfoliative dermatitis
Injection site swelling/induration (hardness)	< 25 mm/ < 2.5 cm	25 - 50 mm/ 2.5 - 5 cm	51 - 100 mm/ 5.1 - 10 cm	> 100 mm/ > 10 cm	Necrosis
Axillary (underarm) swelling or tenderness ipsilateral to the side of injection*	None	No interference with activity	Repeated use of over-the-counter (non-narcotic) pain reliever > 24 hours or some interference with activity	Any use of prescription (narcotic) pain reliever or prevents daily activity	Emergency room visit or hospitalization
Headache	None	No interference with activity	Repeated use of over-the-counter pain reliever > 24 hours or some interference with activity	Significant; any use of prescription pain reliever or prevents daily activity	Requires emergency room visit or hospitalization
Fatigue	None	No interference with activity	Some interference with activity	Significant; prevents daily activity	Requires emergency room visit or hospitalization
Myalgia (muscle aches all over body)	None	No interference with activity	Some interference with activity	Significant; prevents daily activity	Requires emergency room visit or hospitalization
Arthralgia (joint aches in several joints)	None	No interference with activity	Some interference with activity	Significant; prevents daily activity	Requires emergency room visit or hospitalization
Nausea/vomiting	None	No interference with activity or 1-2 episodes/ 24 hours	Some interference with activity or > 2 episodes/24 hours	Prevents daily activity, requires outpatient intravenous hydration	Requires emergency room visit or hospitalization for hypotensive shock
Chills	None	No interference with activity	Some interference with activity not requiring medical intervention	Prevents daily activity and requires medical intervention	Requires emergency room visit or hospitalization
Fever (oral)	< 38.0°C < 100.4°F	38.0 – 38.4°C 100.4 – 101.1°F	38.5 – 38.9°C 101.2 – 102.0°F	39.0 – 40.0°C 102.1 – 104.0°F	> 40.0°C > 104.0°F

Any solicited AR that meets any of the following criteria must be entered into the participant's source document and must also be recorded as an AE in the participant's Adverse Event eCRF:

- Solicited local or systemic AR that results in a visit to an HCP (MAAE)
- Solicited local or systemic AR leading to the participant withdrawing from the study or the participant being withdrawn from the study by the investigator (AE leading to withdrawal)
- Solicited local or systemic AR lasting beyond 7 days post injection
- Solicited local or systemic AR that leads to participant withdrawal from IP
- Solicited local or systemic AR that otherwise meets the definition of an SAE

8.3.5. Recording and Follow-up of Pregnancy

Female participants who have a positive pregnancy test at Screening should not be enrolled; participants who have a positive pregnancy test any time during the study should receive no further dosing with IP but should be asked to remain in the study and be followed-up for safety. Pregnancy testing is scheduled to occur at Screening, Day 1, and Day 29 (Table 13).

Details of all pregnancies in female participants will be collected after the start of study treatment and until Day 759.

- If a pregnancy is reported, the investigator should inform the Sponsor within 24 hours of learning of the pregnancy and should follow the procedures outlined in this section.
- Abnormal pregnancy outcomes (eg, spontaneous abortion, fetal death, stillbirth, congenital anomalies, ectopic pregnancy) are considered SAEs.

Pregnancies occurring in participants after enrollment must be reported to Sponsor or designee within 24 hours of the site learning of its occurrence. If the participant agrees to submit this information, the pregnancy must be followed to determine the outcome, including spontaneous or voluntary termination, details of the birth, and the presence or absence of any birth defects, congenital abnormalities, or maternal and/or newborn complications. This follow-up should occur even if intended duration of the safety follow-up for the study has ended. Pregnancy report forms will be distributed to the study site to be used for this purpose. The investigator must immediately (within 24 hours of awareness) report to the Sponsor any pregnancy resulting in an abnormal outcome according to the procedures described for SAEs.

8.3.6. Recording and Follow-up of an AE and/or SAE

The investigator is responsible for ensuring that all AEs and SAEs are recorded in the eCRF and reported to the Sponsor.

Solicited ARs will be collected from Day 1 through 7 days after each dose. Other (unsolicited) AEs will be collected from Day 1 through 28 days after each dose up to Day 57.

Both MAAEs and SAEs will be collected from Day 1 throughout entire study duration (Day 759 for all participants), as specified in the SoEs (Table 13, Table 14, Table 15, Table 16). Any AEs occurring before receipt of IP will be analyzed separately from TEAEs.

At every study site visit or telephone contact, participants will be asked a standard question to elicit any medically related changes, including surveillance for COVID-19 symptoms, in their well-being according to the scripts provided. Participants will also be asked if they have been hospitalized, had any accidents, used any new medications, changed concomitant medication regimens (both prescription and over-the-counter medications), or had any non-study vaccinations.

In addition to participant observations, physical examination findings, or other documents relevant to participant safety classified as an AE will be documented on the AE page of the eCRF.

After the initial AE/SAE report, the Investigator is required to proactively follow each participant at subsequent visits/contacts. All AEs and SAEs will be treated as medically appropriate and followed until resolution, stabilization, the event is otherwise explained, or the participant is LTFU (as defined in Section 7.4).

8.3.7. Time Period and Frequency for Collecting AE and SAE Information

All SAEs will be recorded and reported to the Sponsor or designee immediately and under no circumstance should this exceed 24 hours, as indicated in Section 8.3.10. The Investigator will submit any updated SAE data to the Sponsor within 24 hours of it being available.

Investigators are not obligated to actively seek AEs or SAEs after conclusion of the study participation (Day 759). However, if an investigator learns of any SAE, including a death, at any time after a participant has been discharged from the study and considers the event to be reasonably related to the study IP or study participation, the investigator must promptly notify the Sponsor.

8.3.8. Assessment of Intensity

An event is defined as “serious” when it meets at least one of the predefined outcomes as described in the definition of an SAE (Section 8.3.3), NOT when it is rated as severe.

The severity (or intensity) of an AR or AE refers to the extent to which it affects the participant’s daily activities. The Toxicity Grading Scale for Healthy Adult and Adolescent Volunteers Enrolled

in Preventative Vaccine Clinical Trials (DHHS 2007) will be used to categorize local and systemic reactogenicity events (solicited ARs), clinical laboratory test results, and vital sign measurements observed during this study. Specific criteria for local and systemic reactogenicity events are presented in Section 8.3.4.

The determination of severity for all unsolicited AEs should be made by the Investigator based upon medical judgment and the definitions of severity as follows:

- Mild: These events do not interfere with the participant's daily activities.
- Moderate: These events cause some interference with the participant's daily activities and require limited or no medical intervention.
- Severe: These events prevent the participant's daily activity and require intensive therapeutic intervention.

Changes in the severity of an AE should be documented in the participant's source documentation to allow an assessment of the duration of the event at each level of intensity to be performed. An AE characterized as intermittent requires documentation of onset and duration of each episode. An AE that fluctuates in severity during the course of the event is reported once in the eCRF at the highest severity observed.

8.3.9. Assessment of Causality

The investigator's assessment of an AE's relationship to IP is part of the documentation process but is not a factor in determining what is or is not reported in the study.

The investigator will assess causality (ie, whether there is a reasonable possibility that the IP caused the event) for all AEs and SAEs. The relationship will be characterized using the following classification:

Not related: There is not a reasonable possibility of a relationship to the IP. Participant did not receive the IP OR temporal sequence of the AE onset relative to administration of the IP is not reasonable OR the AE is more likely explained by another cause than the IP.

Related: There is a reasonable possibility of a relationship to the IP. There is evidence of exposure to the IP. The temporal sequence of the AE onset relative to the administration of the IP is reasonable. The AE is more likely explained by the IP than by another cause.

8.3.10. Reporting Adverse Events

The investigator is responsible for reporting all AEs that are observed or reported during the study, regardless of their relationship to IP or their clinical significance. If there is any doubt as to whether a clinical observation is an AE, the event should be reported.

All unsolicited AEs reported or observed during the study will be recorded on the AE page of the eCRF. Information to be collected includes cohort, type of event, time of onset, investigator-specified assessment of severity and relationship to IP, time of resolution of the event, seriousness, as well as any required treatment or evaluations, and outcome. The unsolicited AEs resulting from concurrent illnesses, reactions to concurrent illnesses, reactions to concurrent medications, or progression of disease states must also be reported. All AEs will be followed until they are resolved or stable or judged by the investigator to be not clinically significant. The Medical Dictionary for Regulatory Activities (MedDRA) will be used to code all unsolicited AEs.

Any medical condition that is present at the time that the participant is screened but does not deteriorate should not be reported as an unsolicited AE. However, if it deteriorates at any time during the study, it should be recorded as an unsolicited AE.

Any AE considered serious by the investigator or that meets SAE criteria (Section 8.3.3) must be reported to the Sponsor immediately (within 24 hours of becoming aware of the SAE). The investigator will assess whether there is a reasonable possibility that the IP caused the SAE. The Sponsor will be responsible for notifying the relevant regulatory authorities of any SAE as outlined in the 21 US CFR Parts 312 and 320. The investigator is responsible for notifying the institutional review board (IRB) directly.

If the eCRF is unavailable at the time of the SAE, the following contact information is to be used for SAE reporting:

- SAE Mailbox: Safety_Moderna@iqvia.com
- SAE Hotline (USA and Canada): +1-866-599-1341
- SAE Fax line (USA and Canada): +1-866-599-1342

8.3.11. Regulatory Reporting Requirements for SAEs

Prompt notification by the Investigator to the Sponsor of a SAE is essential so that legal obligations and ethical responsibilities towards the safety of participants and the safety of a study intervention under clinical investigation are met.

The Sponsor has a legal responsibility to notify both the local regulatory authority and other regulatory agencies about the safety of a study intervention under clinical investigation. The Sponsor will comply with country-specific regulatory requirements relating to safety reporting to the regulatory authority, IRBs, and investigators.

Investigator safety reports must be prepared for suspected unexpected serious adverse reactions (SUSAR) according to local regulatory requirements and Sponsor policy and forwarded to investigators as necessary.

An Investigator who receives an Investigator safety report describing a SAE or other specific safety information (eg, summary or listing of SAEs) from the Sponsor will review and then file it along with the IB and will notify the IRB/IEC, if appropriate according to local requirements.

8.4. Monitoring Committees

8.4.1. Protocol Safety Review Team

A Protocol Safety Review Team (PSRT) will be formed to review interim and cumulative blinded safety data on a regular basis with a remit to escalate concerns to the DSMB.

8.4.2. Data and Safety Monitoring Board

An independent DSMB will periodically review blinded and unblinded data, including both safety and cases of COVID-19 at scheduled data review meetings and at 2 planned interim analyses.

- In addition to blinded and unblinded review of safety data, at each data review meeting, the DSMB will review the numbers and rate of COVID-19 cases, including rate of severe COVID disease with prespecified thresholds for imbalance in the treatment groups which would trigger halting rules.
- At the interim analyses, the DSMB will review the interim analyses results and make recommendations to the Sponsor in terms of study results reporting and unblinding based on the boundaries of early efficacy as described in Section 9.6 of the protocol.
- The DSMB will monitor the study for non-efficacy at the IA. The boundary for non-efficacy is non-binding and is based on the nominal confidence interval (CI) of VE to prevent stopping the study early before the vaccine effect ramps up. The boundary for non-efficacy is based on the nominal CI of VE of mRNA-1273 to prevent COVID-19 (the primary objective). If the CI falls below 0%, ie, upper bound < 0%, the boundary for non-efficacy is considered met.
- The DSMB will also monitor the study for vaccine harm based on both COVID-19 and severe disease at data review meetings, the frequency of which will be described in the DSMB charter. Disease will be monitored after the first 15 cases of disease have accrued and will be monitored continuously until the first IA. Severe disease will be continuously monitored after the first 7 cases of severe disease have accrued and will be monitored continuously until the primary analysis time. The monitoring boundary for disease will ensure that the overall 1-sided type I error rate for disease is at most 0.05. Separately, the monitoring boundary for severe disease will ensure that the overall 1-sided type I error rate is at most 0.05. The monitoring boundary for disease will be based on the anticipated number of disease cases at the time of the first IA. The monitoring boundary for severe

disease will be based on the anticipated number of severe disease cases at the time of the primary analysis. Details will be provided in the SAP and DSMB Charter.

- The boundaries are considered guidelines, ie, a recommendation to modify the study would not be based solely on statistical rules, as many other factors (ie, totality of the data from the study including additional efficacy, safety and immunogenicity endpoints as well as data external to the study) may be part of the decision process. In the case a recommendation to continue the trial, regardless of crossing the boundaries for efficacy or inefficacy, the reason for disregarding the boundary must be documented in the meeting minutes and communicated to the Sponsor.

The DSMB will review analyses for early efficacy and non-efficacy. Guiding, non-binding principles for non-efficacy will be provided in the DSMB charter.

After each data review meeting or IA, the DSMB will make a recommendation to the Sponsor to take one of the following courses of action:

- Stop further enrollment due to meeting criteria for early efficacy or due to a safety concern.
- Pause enrollment and consider a change in study design.
- Continue enrollment.

The Sponsor may also request that the DSMB conduct ad hoc reviews of safety events from this study or other data, including new nonclinical or clinical information related to mRNA-1273 external to this study. The DSMB will review all available study data to adjudicate such events in accordance with the DSMB charter.

The DSMB composition, its remit, and frequency of data review will be further defined in the DSMB charter.

8.4.3. Adjudication Committee

An Adjudication Committee (AC) will be assembled for the purpose of reviewing potential cases to determine if the criteria for the primary and secondary endpoints have been met. The AC will remain blinded to treatment assignment. The AC composition, its remit, and frequency of data review will be further defined in a Charter.

8.5. Management of Overdose

As the study treatment is to be administered by a healthcare professional, it is unlikely that an overdose will occur. Dose deviations will be tracked as protocol deviations (Section 11.2.8).

8.6. Pharmacokinetics

Pharmacokinetic parameters are not evaluated in this study.

8.7. Pharmacodynamics

Pharmacodynamic parameters are not evaluated in this study.

8.8. Exploratory Assessments and Biomarkers

Exploratory assessments may include assessment of serologic markers of disease severity, immune response to SARS-CoV-2, RT-PCR of NP swab, or nasal swab, or saliva samples, and genetic sequences of SARS-CoV-2 strains isolated from participants' samples.

8.9. Medical Resource Utilization and Health Economics

Medical resource utilization and health economics parameters are not evaluated in this study.

9. STATISTICAL CONSIDERATIONS

This section summarizes the planned statistical analysis strategy and procedures for the study. The details of statistical analysis will be provided in the statistical analysis plan (SAP), which will be finalized before the clinical database lock for the study and treatment unblinding. If, after the study has begun, but prior to any unblinding, changes are made to primary and/or key secondary objectives/hypotheses, or the statistical methods related to those hypotheses, then the protocol will be amended (consistent with ICH Guideline E9). Changes to other secondary or exploratory analyses made after the protocol has been finalized, along with an explanation as to when and why they occurred, will be listed in the SAP or Clinical Study Report (CSR) for the study. Ad hoc exploratory analyses, if any, will be clearly identified in the CSR.

9.1. Blinding and Responsibility for Analyses

Blinding during the study will be conducted as described in Section 6.2.8. The Sponsor Biostatistics department or designee will generate the randomized allocation schedule(s) for study treatment assignment. Randomization will be implemented via an IRT.

Planned interim and primary analyses are described in Section 9.6. Participant-level unblinding will be restricted to an independent unblinded statistician and, as needed, a statistical programmer performing the IAs, who will have no other responsibilities associated with the study.

In addition to the routine study monitoring outlined in this protocol, an external DSMB will review interim data to safeguard the interests of clinical study participants and to enhancing the integrity of the study. The DSMB will review treatment-level results of the IAs, provided by the independent unblinded statistician. Limited additional Sponsor personnel may be unblinded to the treatment-level results of the IAs, if required, in order to act on the recommendations of the DSMB. The extent to which individuals are unblinded with respect to results of IAs will be documented. Depending on the recommendation of the DSMB, the Sponsor may prepare a regulatory submission after an IA. In this case, pre-identified Sponsor members including the analysis and reporting team will be unblinded to treatment assignments and remain unblinded for the remainder of the study. Participants and investigators will remain blinded.

9.2. Statistical Hypotheses

For the primary efficacy objective, the null hypothesis of this study is that the VE of mRNA-1273 to prevent first occurrence of COVID-19 is ≤ 30% (ie, $H_0^{efficacy}$: VE ≤ 0.3).

The study will be considered to meet the primary efficacy objective if the corresponding CI of VE rules out 30% at either one of the interim analyses or at the primary analysis.

Vaccine efficacy is defined as the percent reduction in the hazard of the primary endpoint (mRNA-1273 vs. placebo). Equivalently, the null hypothesis is:

$H_0^{efficacy}$: hazard ratio (HR) $\geq$ 0.7 (equivalently, proportional hazards VE $\leq$ 0.3).

A stratified Cox proportional hazard model will be used to assess the magnitude of the treatment group difference (ie, HR) between mRNA-1273 and placebo at a 1-sided 0.025 significance level.

The primary analysis population for efficacy will be the Per-Protocol (PP) Set, defined in Section 9.4. In the primary analysis of efficacy, cases will be counted starting 14 days after the second dose of IP.

9.3. Sample Size Determination

The sample size is driven by the total number of cases to demonstrate VE (mRNA-1273 vs. placebo) to prevent COVID-19. Under the assumption of proportional hazards over time and with 1:1 randomization of mRNA-1273 and placebo, a total of 151 COVID-19 cases will provide 90% power to detect a 60% reduction in hazard rate (60% VE), rejecting the null hypothesis H_0: VE $\leq$ 30%, with 2 IAs at 35% and 70% of the target total number of cases using a 1-sided O'Brien-Fleming boundary for efficacy and a log-rank test statistic with a 1-sided false positive error rate of 0.025. The total number of cases pertains to the PP Set accruing at least 14 days after the second dose. There are 2 planned IAs in this study, which will be performed when approximately 35% and 70% of the target total number of cases have been observed. Up to approximately 30,000 participants will be randomized with the following assumptions:

- The target VE against COVID-19 is 60% (with 95% CI lower bound ruling out 30%, rejecting the null hypothesis H_0: VE $\leq$ 30%)
- A 6-month COVID-19 incidence rate of 0.75% in the placebo arm
- An annual dropout rate of 2% (loss of evaluable participants)
- Fifteen percent of the participants are seropositive at baseline, are non-compliant with injection assessments, or have major protocol deviations
- Two IAs at 35% and 70% of total target cases across the 2 treatment groups with O'Brien-Fleming boundaries for efficacy monitoring
- 3-month uniform accrual
- Approximately 15% of participants will be excluded from the PP population, and participants are at risk for COVID-19 starting 14 days after the second dose

Table 5 provides sample size with 90% power to demonstrate VE on COVID-19.

Table 5: Conditions and Sample Size to Demonstrate Vaccine Efficacy

Target VE	Lower Bound	Randomization Ratio	Total # of Cases	6-Month Incidence Rate		Total Sample Size*
				Placebo	mRNA-1273	
60%	30%	1:1	151	0.75%	0.30%	30,000

*Sample size to account for 15% participants to be excluded from the PP Set (eg, seropositive at baseline, have not received planned IP)

The sample size is calculated using R package gsDesign (Anderson 2007)

Under these above assumptions including 6-month incidence rate of 0.75% on placebo, with 30,000 subjects, it will take approximately 5, 8, and 10 months from study start (first subject first dose), respectively, to accrue 35% (approximately 53), 70% (approximately 106) and 100% (151) of the target number of cases in the PP Set.

Figure 4 shows the power of the primary efficacy endpoint under true VE at the 2 planned IAs and the primary efficacy analysis assuming a total of 151 events.

Figure 4: Boundary Crossing Probabilities by Effect Size

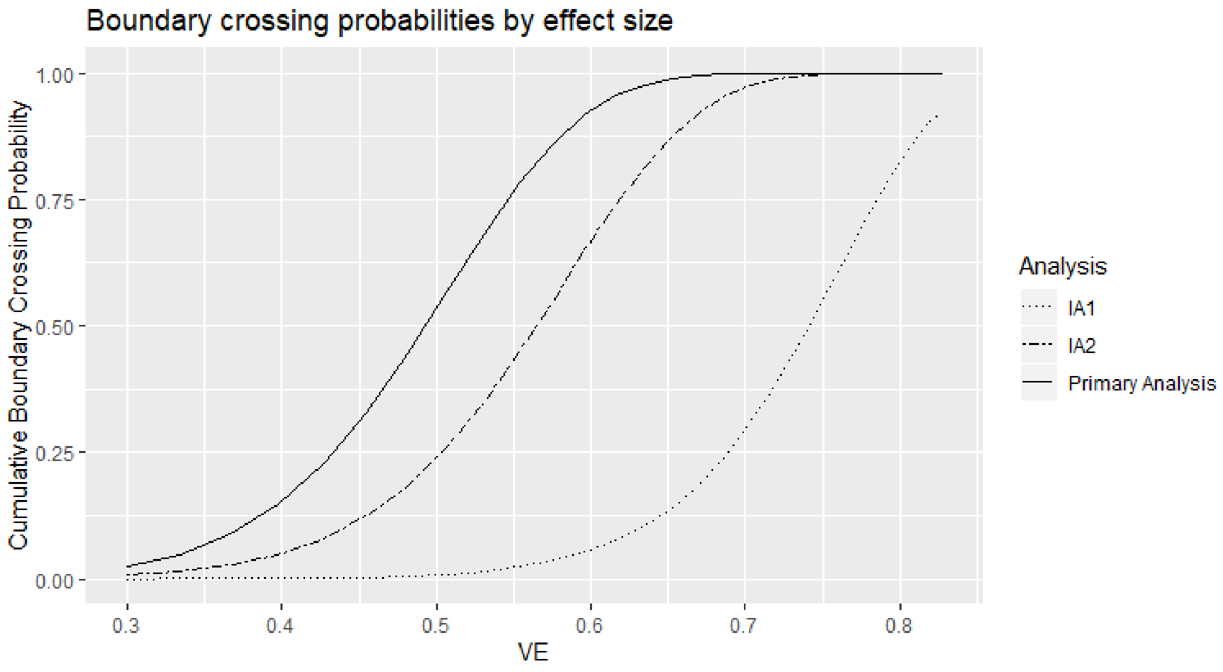

The Sponsor may adjust the size of the study or duration of follow-up based on the blinded review of the total number of cases of COVID-19 accrued during the study, in addition to estimated percentages of study participants with serologic evidence of SARS-CoV-2 infection at baseline.

9.3.1. Power for Selected Secondary Efficacy Endpoints

For the secondary objective on VE against virologically confirmed SARS-CoV-2 infection or COVID-19 regardless of symptomology or severity (COV-INF), the study will have ≥ 90% power to demonstrate the VE is above 30% (to reject null hypothesis VE ≤ 30%) at 1-sided alpha of 2.5% if the true VE to prevent COV-INF is 60% because every COVID-19 endpoint is necessarily a COV-INF endpoint.

For the secondary objective on VE against severe COVID-19, Table 6 provides power of demonstrating VE based on a total of 20 and 30 events under different scenarios of true VE and VE criteria.

Table 6: Power of Demonstrating Vaccine Efficacy Against Severe COVID-19

True VE	Total No. of Severe COVID-19 Cases	Severe COVID-19 (Secondary Objective)	
		To reject null hypothesis	Power
60%	30	VE≤ 0%	70.9%
70%	20	VE≤ 10%	60.3%
70%	20	VE≤ 20%	47.5%
70%	20	VE≤ 0%	> 90%
60%	20	VE≤ 10%	85.3%
60%	20	VE≤ 20%	76.6%
80%	20	VE≤ 0%	> 90%
50%	20	VE≤ 10%	> 90%
50%	20	VE≤ 20%	> 90%
60%	60	VE≤ 0%	> 90%
70%	30	VE≤ 10%	88.1%
70%	30	VE≤ 20%	76.6%
70%	30	VE≤ 0%	> 90%
60%	30	VE≤ 10%	> 90%
60%	30	VE≤ 20%	> 90%
80%	30	VE≤ 0%	> 90%
50%	30	VE≤ 10%	> 90%
50%	30	VE≤ 20%	> 90%

9.4. Analysis Populations

Analysis populations for statistical analyses are Randomization Set, Full Analysis Set (FAS), modified intent-to-treat (mITT) set, PP Set, Immunogenicity Subset, Solicited Safety Set, and Safety Set, as shown in Table 7.

Table 7: Populations for Analyses

Population	Description

Randomization Set	All participants who are randomized, regardless of the participants' treatment status in the study.
Full Analysis Set (FAS)	All randomized participants who received at least one dose of IP. Participants will be analyzed according to the group to which they were randomized.
Modified Intent-to-Treat (mITT) Set	All participants in the FAS who had no serologic or virologic evidence of prior COVID-19 or SARS-CoV-2 infection defined by bAb against SARS-CoV-2 nucleocapsid below LOD or LLOQ at Day 1 before the first dose of IP. Participants will be analyzed according to the group to which they were randomized.
Per-protocol (PP) Set	All participants in the mITT Set who received planned doses of IP per schedule and have no major protocol deviations. Participants will be analyzed according to the group to which they were randomized.
Immunogenicity Subset	All participants in the FAS who had a valid immunogenicity test result prior to the first dose of IP and at least 1 valid result after the first dose of IP.
Solicited Safety Set	The Solicited Safety Set consists of all randomized participants who received at least one dose of IP and contributed any solicited AR data. The Solicited Safety Set will be used for the analyses of solicited ARs and participants will be included in the treatment group corresponding to the IP that they actually received.
Safety Set	All randomized participants who received at least one dose of IP. The Safety Set will be used for all analyses of safety except for the solicited ARs. Participants will be included in the treatment group corresponding to the IP that they actually received.

9.5. Statistical Analyses

This section provides a summary of the planned statistical analyses of the primary and secondary endpoints.

The overall Type I error rate for the primary endpoint at the IAs and the primary analysis is strictly controlled at 2.5% (1-sided) based on the Lan-DeMets O'Brien-Fleming approximation spending function (see Section 9.6 for details). The primary efficacy results that will be considered statistically significant after consideration of the strategy for controlling the Type I error as described in Section 9.6. Statistical significance of the primary efficacy endpoint can be achieved at either one of the interim analyses or at the primary analysis. A sequential/hierarchical testing procedure will be used to control type 1 error rate over the primary efficacy endpoint and the

secondary efficacy endpoints. Secondary efficacy endpoints will only be tested when the primary efficacy endpoint achieves statistical significance. Multiplicity adjustments among the secondary efficacy endpoints may be performed for secondary efficacy endpoints, in that case, will be specified in the SAP.

No formal multiple comparison adjustments will be employed for multiple safety endpoints or multiple efficacy endpoints. Nominal p-values and confidence intervals may be computed for other efficacy analyses without controlling for multiplicity as a measure of VE.

9.5.1. Efficacy Analyses

Efficacy analyses will be performed using the FAS, mITT and PP populations, and participants will be included in the treatment group to which they are randomized. The primary analysis population will be the PP Set.

Table 8 summarizes the analysis approach for primary and secondary efficacy endpoints. Sensitivity analysis methods are described for each endpoint as applicable.

Table 8: Statistical Analysis Methods of Efficacy Endpoints

Endpoint	Statistical Analysis Methods
**Primary endpoint:**<br>Vaccine Efficacy (VE) of mRNA-1273 to prevent COVID-19	• Primary analysis: VE will be estimated with 1 - HR (mRNA-1273 vs placebo) using a Cox proportional hazard regression model with treatment group as a fixed effect and adjust for stratification factor based on the PP Set, with cases counted starting 14 days after the second dose of IP.<br>• Analysis using the same model based on the mITT Set.<br>• Sensitivity analysis using the same model based on the PP Set, with cases counted starting either immediately after the second dose of IP or immediately after the first dose of IP<br>• Subgroup analysis of the primary efficacy endpoint will be performed to assess consistency of VE, such as in the age groups ≥ 18 and < 65 years and ≥ 65 years<br>• Supportive analysis of VE to be estimated with 1 - ratio of incidence rates with 95% CI using the exact method conditional upon the total number of cases<br>• Supportive analysis of cumulative incidence VE
**Secondary endpoints:**<br>• Vaccine efficacy of mRNA-1273 to prevent severe COVID-19<br>• Vaccine efficacy of mRNA-1273 to prevent serologically confirmed SARS-CoV-2 infection or COVID-19 regardless of symptomatology or severity<br>• Vaccine efficacy of mRNA-1273 to prevent COVID-19 using a secondary definition of symptoms<br>• Vaccine efficacy of mRNA-1273 to prevent death due to COVID-19<br>• Vaccine efficacy of mRNA-1273 to prevent COVID-19 after the first dose of IP<br>• Vaccine efficacy of mRNA-1273 to prevent asymptomatic SARS-CoV-2 infection	Similar analysis method as for the primary endpoint analysis.<br>For each of the secondary endpoints:<br>• Primary analysis: VE will be estimated with 1 - HR (mRNA-1273 vs placebo) using a Cox proportional hazard regression model with treatment group as a fixed effect and adjusting for stratification factor based on the PP Set, with cases counted starting 14 days after the second dose of IP.<br>• Analysis using the same model based on the mITT Set.<br>• Sensitivity analyses with cases counted starting immediately after the second dose of IP, 14 days after the first dose of IP, immediately after the first dose of IP, and immediately after randomization.<br>• Vaccine efficacy and 95% CI based on the incidence of will be estimated with 1 - ratio of incidence rates using the exact method conditional upon the total number of cases.
• Vaccine efficacy of mRNA-1273 to prevent all-cause mortality.	Similar analysis method as for the primary endpoint analysis using PP Set, mITT Set, and FAS. Death, regardless of cause, from time of randomization will be included.
• Vaccine efficacy of mRNA-1273 to prevent COVID-19 in all study participants, regardless of evidence of prior SARS-CoV-2 infection	The FAS population will be used for this secondary objective, using similar analysis methods as for the primary endpoint analysis.<br>• Primary analysis: VE will be estimated with 1 - HR (mRNA-1273 vs placebo) using a Cox proportional hazard regression model with treatment group as a fixed effect and adjusting for stratification factor based on the FAS, with cases counted starting 14 days after the second dose of IP.<br>• Sensitivity analyses with cases counted starting immediately after the second dose of IP, 14 days after the first dose of IP, immediately after the first dose of IP, and immediately after randomization.

9.5.1.1. Efficacy Analysis on Primary Endpoint

To assess the primary efficacy endpoint of VE of mRNA-1273 in preventing the first occurrence of COVID-19 from 14 days after second dose of IP, Cox proportional hazards regression will be used to estimate proportional hazards VE (PH VE), measured by one minus the HR (mRNA-1273 vs. placebo), with a 2-sided score-based 95% CI and 2-sided *p*-value for testing H_0: VE ≤ 30%.

Vaccine efficacy is defined as the percent reduction in the hazard of the primary endpoint (mRNA-1273 vs placebo). The VE will be estimated using one minus the HR (mRNA-1273 vs placebo) estimand. A stratified Cox proportional hazard model will be used to assess the magnitude of the treatment group difference (ie, HR) between mRNA-1273 and placebo. The HR and its 95% CI from the stratified Cox model with Efron's method of tie handling and with treatment group as covariate will be reported. The same stratification factors used for randomization will be applied to the stratified Cox model.

For the primary efficacy endpoints, participants without documented COVID-19 will be censored at the last study assessment date. Potential intercurrent events may include: 1) withdrawal from the study or death unrelated to COVID-19; 2) early COVID-19 up to 14 days after second study dose; 3) missing dose of IP; and 4) seropositive at baseline.

In the estimand of the primary analysis on the primary endpoint, a treatment policy strategy will be used to address the intercurrent events of 1) withdrawal from the study or death unrelated to COVID-19, where the time to COVID-19 will be censored at the date of withdrawal from the study or death; 2) early COVID-19, where the time to COVID-19 will be censored at the time of early infection. Principal stratum strategy will be used to address the other 2 types of intercurrent events in the primary analysis based on the PP Set. The details of intercurrent event description and estimand strategies are presented in Section 11.4.1.

For the primary efficacy analysis, cases will be counted starting 14 days after the second vaccination. Sensitivity analyses with cases counted immediately after the second vaccination, and after randomization will also be carried out.

Analyses of the primary endpoint will be also performed based on the mITT Set using the same methods described above.

For the primary efficacy analysis, cases will be counted starting 14 days after the second dose of IP. Sensitivity analyses with cases counted starting immediately after the second dose of IP and starting immediately after randomization will also be carried out.

Subgroup analysis of the primary efficacy endpoint will be performed in selected subgroups, such as age groups ≥18 and <65 year and ≥65 years to assess consistency of VE as described in Section 9.5.5.

As a supportive analysis, VE will also be estimated by one minus the infection rate ratio, where the number of cases (ie, participants with first occurrence of COVID-19) will be used and the CI will be computed using the exact method conditional upon the total number of cases. Cumulative incidence VE, one minus the ratio of cumulative incidences (mRNA-1273 vs placebo) of COVID-19, may also be assessed, the cumulative incidence for each arm will be estimated using a covariate adjustment method based on Zeng (2004) that makes use of baseline characteristics.

9.5.1.2. Efficacy Analysis on Secondary Endpoint

For each of the below secondary objectives:

- Vaccine efficacy to prevent severe COVID-19
- Vaccine efficacy to prevent serologically confirmed SARS-CoV-2 infection or COVID-19 regardless of symptomology or severity
- Vaccine efficacy to prevent COVID-19 using a broad definition of symptoms
- Vaccine efficacy to prevent death caused by COVID-19
- Vaccine efficacy to prevent asymptomatic SARS-CoV-2 infection

For each of the above secondary objectives, the same Cox proportional hazard model described above for the primary objective will be applied using the PP Set, with cases counted starting 14 days after the second dose of IP. Sensitivity analyses with cases counted starting after the second dose of IP, 14 days after the first dose of IP, after the first dose of IP, and after randomization will also be performed.

The same model will be applied using the mITT population with cases counted starting 14 days after the second dose of IP.

Vaccine efficacy will be estimated with 1- ratio of incidence rates with the 95% CI using the exact method conditional upon the total number of cases.

<u>Vaccine efficacy to prevent COVID-19 after the first dose of IP</u>

The same Cox proportional hazard model described above for the primary objective will be applied using the PP Set, with cases counted starting 14 days after the first dose of IP.

<u>Vaccine efficacy to prevent all-cause mortality</u>

The same Cox proportional hazard model described above for the primary objective will be applied using the PP Set, the mITT Set, and the FAS. Death, regardless of cause, from randomization will be included.

Vaccine efficacy to prevent COVID-19 regardless of prior SARS-CoV-2 infection

The FAS will be used for analysis to evaluate VE to prevent COVID-19 regardless of prior SARS-CoV-2 infection. The same methods described above for the primary objective will be applied with cases counted starting 14 days after the second dose of IP. Sensitivity analyses with cases counted starting after the second dose of IP, 14 days after the first dose of IP, the first dose of IP, and randomization will also be performed.

Vaccine efficacy will be estimated with 1- ratio of incidence rates with the 95% CI using the exact method conditional upon the total number of cases.

9.5.2. Safety Analyses

All safety analyses will be based on the Safety Set, except summaries of solicited ARs, which will be based on the Solicited Safety Set. All safety analyses will be provided by treatment group and baseline SARS-CoV-2 serostatus, unless otherwise specified.

9.5.2.1. Adverse Events

Safety and reactogenicity will be assessed by clinical review of all relevant parameters.

The number and percentage of participants with any solicited local AR, with any solicited systemic AR, and with any solicited AR during the 7-day follow-up period after each dose will be provided. A 2-sided 95% exact CI using the Clopper-Pearson method will be also provided for the percentage of participants with any solicited AR for each treatment group.

Number and percentage of participants with unsolicited AEs, SAEs, MAAEs, Grade 3 or higher ARs and AEs, and AEs leading to discontinuation from IP or withdrawal from the study will be summarized. Unsolicited AE will be presented by MedDRA preferred term and system organ class.

Number of events of solicited ARs, unsolicited AEs/SAEs, and MAAEs will be reported in summarization tables accordingly.

For all other safety parameters, descriptive summary statistics will be provided, and Table 9 summarizes analysis strategy for safety parameters.

Table 9: Analysis Strategy for Safety Parameters

Safety Endpoint	Number and Percentage of Participants, Number of Events	95% CI

Any Solicited AR (overall and by local, systemic)	X	X
Any Unsolicited AE	X	
Any SAE	X	
Any Unsolicited MAAE	X	
Any Unsolicited Treatment-Related AE	X	
Any Treatment-Related SAE	X	
Discontinuation due to AE	X	
Any Grade 3 and above AE	X	
Any Treatment-Related Grade 3 and above AE	X	

Notes: 95% CI using the Clopper-Pearson method, X = results will be provided. Unsolicited AEs will be summarized by SOC and PT coded by MedDRA.

Further details will be described in the SAP.

9.5.2.2. Baseline Descriptive Statistics

Demographic variables and baseline characteristics will be summarized by treatment group by descriptive statistics (mean, standard deviation for continuous variable, and number and percentage for categorical variables).

9.5.3. Immunogenicity Analyses

The secondary immunogenicity endpoints will be analyzed using the Immunogenicity Subset, by treatment group and by baseline SARS-CoV-2 serostatus, unless otherwise specified.

The SAP will describe the complete set of immunogenicity analyses, including the approach to sample individuals into an immunogenicity subset for characterizing mRNA-1273 immunogenicity and assessing immunological correlates of risk and protection.

Data from quantitative immunogenicity assays will be summarized for each treatment group using positive response rates and geometric means with 95% confidence intervals, for each timepoint for which an assessment is performed. Data from qualitative (ie, yielding a positive or negative result) assays will be summarized by tabulating the frequency of positive responses for each assay by group at each timepoint that an assessment is performed. Analyses will focus on the 2 key immunogenicity time points and the change in marker response between them: Day 1 before the first dose of IP and Day 57 (28 days after the second dose of IP). The SAP will describe the complete set of immunogenicity analyses.

Quantitative levels or GMT of specific bAb with corresponding 95% CI at each timepoint and GMFR of specific bAb with corresponding 95% CI at each post-baseline timepoint over pre-dose

baseline at Day 1 will be provided by study arm. Descriptive summary statistics including median, minimum, and maximum will also be provided.

GMT of specific nAb with corresponding 95% CI at each timepoint and GMFR of specific nAb with corresponding 95% CI at each post-baseline timepoint over pre-dose baseline at Day 1 will be provided by study arm. Descriptive summary statistics including median, minimum, and maximum will also be provided. For summarizations of group values, antibody values reported as below the LOD or LLOQ will be replaced by 0.5 × LOD or 0.5 × LLOQ. Values that are greater than the ULOQ will be converted to the ULOQ.

The number and percentage of participants with GMFR ≥ 2, GMFR ≥ 3, and GMFR ≥ 4 of serum SARS-CoV-2-specific nAb titers and participants with seroconversion from baseline will be provided with 2-sided 95% CI using the Clopper-Pearson method at each post-baseline timepoint.

Seroconversion at a participant level is defined as a change of bAb titer against SARS-CoV-2 nucleocapsid antigen from below the LOD or LLOQ to equal to or above LOD or LLOQ (respectively), or a 4-times or higher log-transformed titer ratio in participants with pre-existing bAb titers.

Geometric mean ratio (GMR) of mRNA-1273 arm versus Placebo arm and its 2-sides 95% CI will be calculated at each study timepoint using an analysis of covariance (ANCOVA) model with the treatment group as an exploratory variable, the analysis may adjust for the stratification factor (Table 10).

Table 10: Immunogenicity Endpoints and Statistical Methods

Endpoint	Statistical Analysis Methods
GMT	Descriptive statistics
GMFR	• GMFR - descriptive statistics<br>• Binomial endpoints of GMFR ≥2, 3, and 4, and seroconversion – the Clopper-Pearson method
GMR	• Descriptive statistics<br>• model

Further details will be described in the SAP.

9.5.4. Exploratory Analyses

The endpoint of viral infection kinetics will be assessed by determining the number of days until testing of either NP swab, nasal swab, or saliva sample become negative after COVID-19 is established.

The endpoint of duration of symptoms will be assessed by determining the total number of days that a study participant with COVID-19 remains symptomatic through daily assessments after diagnosis.

This endpoint of BOD is defined based on the post SARS-CoV-2 infection follow-up. A BOD score will be used to reflect the severity of symptoms with maximum score at COVID-19 death (Table 11).

Table 11: Burden of Disease Score

Patient State	BOD Score
Uninfected/Asymptomatic infection	0
Symptomatic without hospitalization	1
Hospitalization	2
Death	3

All other analyses of exploratory endpoints will be described in the SAP before database lock.

9.5.5. Subgroup Analyses

To determine whether the VE is consistent across various subgroups, the VE and its 95% CI may be estimated using the similar model within each category of the following classification variables.

- Age groups: ≥ 18, < 65, and ≥ 65
- Age and health risk for severe disease: ≥ 18, < 65 and not at risk, ≥ 18 and < 65 at risk, and ≥ 65
- Sex (female, male)
- Race (white, non-white)
- At risk for severe COVID-19 illness (Section 6.2.1.1)

9.6. Interim Analyses

There are 2 planned IAs at 35% and 70% of total target cases across the 2 treatment groups. The primary objective of the IAs is for early detection of reliable evidence that VE is above 30%. The Lan-DeMets O'Brien-Fleming approximation spending function is used for calculating efficacy bounds and to preserve the (1-sided) 0.025 false positive error rate over the IAs and the primary analysis (when the target number of cases have been observed), relative to the hypothesis:

$H_0^{efficacy}$: HR ≥ 0.7 (equivalently, proportional hazards VE ≤ 0.3).

There is no intention to stop the study early if the efficacy has been demonstrated at any of the IAs. If efficacy is demonstrated at an IA, the subsequent IA or primary analysis will be considered supportive in nature. The DSMB will review the IA results and make recommendations to the Sponsor in terms of study results reporting and unblinding based on the boundaries of early efficacy as described in this section, safety data and data external to this study. In addition to possible early efficacy at IAs, the DSMB will monitor for non-efficacy and vaccine harm; the guiding principles (non-binding) is provided in Section 8.4.2, and the details will be provided in the SAP.

Table 12 summarizes the timing, number of cases and decision guidance at each IA and primary analysis.

The first IA will occur when approximately 35% of the total cases have been observed (across both treatment groups). The study will be considered positive at the first IA if the p-value for rejecting HR ≥ 0.7 is less than 0.0002 based on the Lan-DeMets O'Brien-Fleming approximation spending function. This corresponds to an observed HR of approximately 0.259, or an observed VE approximately 0.741.

The second IA will occur when approximately 70% of the total cases have been observed. The study will be considered positive (VE has been demonstrate) if the p-value for rejecting HR ≥ 0.7 is less than 0.0073 based on the Lan-DeMets O'Brien-Fleming approximation spending function. This corresponds to an observed HR of approximately 0.435, or an observed VE of approximately 0.565.

The primary analysis will be performed when approximately 151 cases have been observed in the study. The study will be considered positive at the primary analysis if the 1-sided p-value for rejecting HR ≥ 0.7 is less than 0.0227. This corresponds to an observed HR of approximately 0.505 or observed VE of approximately 0.495.

Table 12: Interim Boundaries Using O’Brien-Fleming Spending function, Calculation Based on the PP Set for the Primary Efficacy Endpoint

Information fraction (% of total #cases)	Number of cases	Nominal Alpha	Efficacy Boundary Rejecting H0 (HR ≥ 0.7, VE LB> 30%)	Cum Prob (crossing efficacy boundary if the true VE = 60%)
**IA1 35%**	53	0.0002	VE ≥ 0.741<br>(HR ≤ 0.259)	4.6%
**IA2 70%**	106	0.0073	VE ≥ 0.565<br>(HR ≤ 0.435)	61.5%
**Primary analysis 100%**	151	0.0227	VE ≥ 0.495<br>(HR ≤ 0.505)	90.0%

Abbreviations: HR = hazard ratio; IA: interim analysis; LB = lower boundary; PP = per-protocol; VE = vaccine efficacy.

10. REFERENCES

Anderson K. gsDesign, version 3.1.1. 2020. Available from The Comprehensive R Archive Network at https://cran.r-project.org/web/packages/gsDesign/.

Centers for Disease Control and Prevention (CDC). 2020a. Coronavirus Disease 2019 (COVID-19). Retrieved from: https://www.cdc.gov/coronavirus/2019-ncov/cases-updates/cases-in-us.html. (accessed 2020 May 29).

Centers for Disease Control and Prevention (CDC). 2020b. Coronavirus Disease 2019 (COVID-19). Retrieved from: https://www.cdc.gov/coronavirus/2019-ncov/need-extra-precautions/people-at-higher-risk.html (accessed 2020 May 29).

Centers for Disease Control and Prevention (CDC). 2020c. Coronavirus Disease 2019 (COVID-19). Retrieved from: https://www.cdc.gov/coronavirus/2019-ncov/symptoms-testing/symptoms.html (accessed 2020 Jun 11).

Chen Y, Lu S, Jia H, Deng Y, Zhou J, Huang B, et al. A novel neutralizing monoclonal antibody targeting the N-terminal domain of the MERS-CoV spike protein. Emerg Microbes Infect. 2017 May 24;6(5):e37.

Chin J, Magoffin RL, Shearer LA, Schieble JH, Lennette EH. Field evaluation of a respiratory syncytial virus vaccine and a trivalent parainfluenza virus vaccine in a pediatric population. Am J Epidemiol 1969;89:449–63

ClinicalTrials.gov [Internet]. Bethesda (MD): National Library of Medicine (US). - Identifier NCT03076385, Safety, tolerability, and immunogenicity of VAL-506440 in healthy adult subjects. Retrieved from: https://clinicaltrials.gov/ct2/keydates/NCT03076385. (accessed 2020 Jun 01).

ClinicalTrials.gov [Internet]. Bethesda (MD): National Library of Medicine (US). - Identifier NCT03325075, Safety, tolerability, and immunogenicity of VAL-181388 in healthy subjects. Retrieved from: https://clinicaltrials.gov/ct2/keydates/NCT03325075. (accessed 2020 Jun 01).

ClinicalTrials.gov [Internet]. Bethesda (MD): National Library of Medicine (US). - Identifier NCT03345043, Safety, tolerability, and immunogenicity of VAL-339851 in healthy adult subjects. Retrieved from: https://clinicaltrials.gov/ct2/keydates/NCT03345043. (accessed 2020 Jun 01).

ClinicalTrials.gov [Internet]. Bethesda (MD): National Library of Medicine (US). - Identifier NCT03382405, Safety, reactogenicity, and immunogenicity of cytomegalovirus vaccines mRNA-1647 and mRNA-1443 in healthy adults. Retrieved from: https://clinicaltrials.gov/ct2/keydates/NCT03382405. (accessed 2020 Jun 01).

ClinicalTrials.gov [Internet]. Bethesda (MD): National Library of Medicine (US). - Identifier NCT03392389, Safety, reactogenicity, and immunogenicity of mRNA-1653 in healthy adults. Retrieved from: https://clinicaltrials.gov/ct2/keydates/NCT03392389. (accessed 2020 Jun 01).

ClinicalTrials.gov [Internet]. Bethesda (MD): National Library of Medicine (US). - Identifier NCT04283461, Safety and immunogenicity study of SARS-CoV-2 vaccine (mRNA-1273) to treat novel coronavirus; 2020 Feb 22. Retrieved from: https://clinicaltrials.gov/ct2/keydates/NCT04283461. (accessed 2020 Jun 01).

Corti D, Zhao J, Pedotti M, Simonelli L, Agnihothram S, Fett C, et al. Prophylactic and postexposure efficacy of a potent human monoclonal antibody against MERS coronavirus. Proc Natl Acad Sci U S A. 2015 Aug 18;112(33):10473-8.

Department of Health and Human Services (DHHS), Food and Drug Administration, Center for Biologics Evaluation and Research (US). Guidance Document. FDA guidance on conduct of clinical trials of medical products during COVID-19 public health emergency. March 2020. Updated on April 16, 2020. [cited 2020 Apr 17]. Available from: https://www.fda.gov/media/136238/download.

Department of Health and Human Services (DHHS), Food and Drug Administration, Center for Biologics Evaluation and Research (US). Guidance for industry: Toxicity grading scale for healthy adult and adolescent volunteers enrolled in preventative vaccine clinical trials. September 2007 [cited 2019 Apr 10] [10 screens]. Available from:

https://www.fda.gov/downloads/BiologicsBloodVaccines/GuidanceComplianceRegulatoryInformation/Guidances/Vaccines/ucm091977.pdf.

Feldman RA, Fuhr R, Smolenov I, Mick Ribeiro A, Panther L, Watson M, et al. mRNA vaccines against H10N8 and H7N9 influenza viruses of pandemic potential are immunogenic and well tolerated in healthy adults in phase 1 randomized clinical trials. Vaccine. 2019 May 31;37(25):3326-3334.

Fulginiti VA, Eller JJ, Downie AW, Kempe CH. Altered reactivity to measles virus. Atypical measles in children previously immunized with inactivated measles virus vaccines. JAMA. 1967 202:1075-80.

Johnson RF, Bagci U, Keith L, Tang X, Mollura DJ, Zeitlin L, et al. 3B11-N, a monoclonal antibody against MERS-CoV, reduces lung pathology in rhesus monkeys following intratracheal inoculation of MERS-CoV Jordan-n3/2012. Virology. 2016 Mar;490:49-58.

Kim Y, Lee H, Park K, Park S, Lim JH, So MK, et al. Selection and characterization of monoclonal antibodies targeting middle east respiratory syndrome coronavirus through a human synthetic fab phage display library panning. Antibodies (Basel). 2019 Jul 31;8(3).

Thomas SJ and Yoon IK. A review of Dengvaxia®: development to deployment. Hum Vaccin Immunother. 2019;15(10), 2295-2314.

Wang L, Shi W, Joyce MG, Modjarrad K, Zhang Y, Leung K, et al. Evaluation of candidate vaccine approaches for MERS-CoV. Nat Commun. 2015 Jul 28;6:7712.

Wang L, Shi W, Chappell JD, Joyce MG, Zhang Y, Kanekiyo M, et al. Importance of neutralizing monoclonal antibodies targeting multiple antigenic sites on the middle east respiratory syndrome coronavirus spike glycoprotein to avoid neutralization escape. J Virol. 2018 Apr 27;92(10).

Widjaja I, Wang C, van Haperen R, Gutiérrez-Álvarez J, van Dieren B, Okba NMA, et al. Towards a solution to MERS: protective human monoclonal antibodies targeting different domains and functions of the MERS-coronavirus spike glycoprotein. Emerg Microbes Infect. 2019;8(1):516-30.

World Health Organization (WHO). Dengue vaccine: WHO position paper, September 2018 – recommendations. Vaccine. 2019 Aug;37(35):4848-4849

World Health Organization (WHO). 2020 (Data reported as of 2020, May 28). Coronavirus disease 2019 (COVID-19) Situation Report – 129. Retrieved from: https://www.who.int/docs/default-source/coronaviruse/situation-reports/20200528-covid-19-sitrep-129.pdf?sfvrsn=5b154880_2. (accessed 2020 May 29).

Wrapp D, Wang N, Corbett KS, Goldsmith JA, Hseih CL, Abiona O, et al. Cryo-EM structure of the 2019-nCoV spike in the prefusion conformation. Science. 2020;367:1260-3.

Yu X, Zhang S, Jiang L, Cui Y, Li D, Wang D, et al. Structural basis for the neutralization of MERS-CoV by a human monoclonal antibody MERS-27. Sci Rep. 2015 Aug 18;5:13133.

Zeng D. 2004. Estimating marginal survival function by adjusting for dependent censoring using many covariates. The Annals of Statistics, 32(4), 1533-1555.

11. SUPPORTING DOCUMENTATION AND OPERATIONAL CONSIDERATIONS

11.1. APPENDIX 1: Schedules of Events

The SoEs are presented in Table 13, Table 14, Table 15, and Table 16.

If a participant cannot attend a study site visit (scheduled or unscheduled) with the exception of Screening, Day 1, and Day 29 visits, a home visit is acceptable if performed by appropriately delegated study site staff or a home healthcare service provided by the Sponsor. If neither a participant visit to the study site nor a home visit to the participant is possible (with the exception of Screening, Day 1, and Day 29 visits), a safety phone call should be performed that includes the assessments scheduled for the biweekly safety phone calls (Table 13).

Table 13: Schedule of Events (Vaccination Phase, Day 1 – Day 57)

Visit Number	0	1				2				3
Type of Visit	C	C	SC	SC	SC	C	SC	SC	SC	C
Month/Weekly Timepoint		M0				M1				M2
**Study Visit Day**	D0[1] (Screening)	D1 (Baseline)	7 days after D1 D8	14 days after D1 D15	21 days after D1 D22	D29 [9]	7 days after D29 D36	14 days after D29 D43	21 days after D29 D50	D57 [9]
**Window Allowance (Days)**	-28		+3	+3	+3	-3/+7	+3	+3	+3	-3/+7
**Days Since Most Recent Vaccination**	-	0	7	14	21	28/0	7	14	21	28
ICF, demographics, concomitant medications, medical history	X									
Confirm participant meets inclusion and exclusion criteria	X	X								
Physical examination[2]	X	X				X				X
Pregnancy testing[3]	X	X				X				
Randomization		X								
**Dosing**										
Study injection (including 30-minute post-dosing observation period)		X				X				
**Efficacy Assessment**										
Surveillance for COVID-19/Unscheduled Visit[4]		X	X	X	X	X	X	X	X	X
Nasopharyngeal swab		X								
**Immunogenicity Assessment**										
Blood for serologic analysis[5]		X				X				X
**Safety Assessments**										
eDiary activation for recording solicited adverse reactions (7 days)[6]		X				X				
Review of eDiary			X				X			
Follow-up Safety[7]			X	X	X		X	X		
Recording of Unsolicited AEs		X	X	X	X	X	X	X	X	X
Recording of MAAEs, AE leading to withdrawal and concomitant medications relevant to or for the treatment of the MAAE[8]		X	X	X	X	X	X	X	X	X

Visit Number	0	1				2				3
**Type of Visit**	C	C	SC	SC	SC	C	SC	SC	SC	C
**Month/Weekly Timepoint**		M0				M1				M2
**Study Visit Day**	D0[1] (Screening)	D1 (Baseline)	7 days after D1 D8	14 days after D1 D15	21 days after D1 D22	D29 [9]	7 days after D29 D36	14 days after D29 D43	21 days after D29 D50	D57 [9]
**Window Allowance (Days)**	-28		+3	+3	+3	-3/+7	+3	+3	+3	-3/+7
**Days Since Most Recent Vaccination**	-	0	7	14	21	28/0	7	14	21	28
Recording of SAEs and concomitant medications relevant to or for the treatment of the SAE[8]	X	X	X	X	X	X	X	X	X	X
Recording of concomitant medications and non-study vaccinations[8]		X	X	X	X	X	X	X	X	X

Abbreviations: AE = adverse event; C = clinic visit; D = day; eDiary= electronic diary; ICF = informed consent form; M = month; MAAE = medically attended AE; SAE = serious adverse event; SC = safety (phone) call.

Note: In accordance with FDA Guidance on Conduct of Clinical Trials of Medical Products during the COVID-19 Pandemic (FDA March 2020), investigators may convert study site visits to telemedicine visits with the approval of the Sponsor.

1. Day 0 and Day 1 may be combined the same day. Additionally, the Day 0 visit may be performed over multiple visits if within the 28-day screening window.
2. Physical examination: a full physical examination, including vital signs, height, and weight, will be performed at Screening and Day 1. Body mass index will be calculated at the Screening Visit (Day 0) only. Symptom-directed physical examinations will be performed on Day 29 and Day 57. On each dosing day before injection, the arm receiving the injection should be examined and the associated lymph nodes should be evaluated. Any clinically significant finding identified during a study visit should be reported as a MAAE. Vital signs are to be collected pre- and post-dosing on days of injection (Day 1 and Day 29). When applicable, vital sign measurements should be performed before blood collection. Participants who are febrile (body temperature ≥ 38.0°C/100.4°F) before dosing on Day 1 or Day 29 must be rescheduled within the relevant window period to receive the injection. Afebrile participants with minor illnesses can be enrolled at the discretion of the investigator.
3. Pregnancy test at Screening and Day 1 and before the second vaccination will be a point-of-care urine test. At the discretion of the investigator a pregnancy test either via blood or point-of-care urine test can be performed. Follicle-stimulating hormone level may be measured to confirm menopausal status at the discretion of the investigator.
4. Participants meeting prespecified criteria of suspicion for COVID-19 will be asked to return within 72 hours or as soon as possible to the study site for an unscheduled visit, to include an NP swab sample (for RT-PCR testing) and other clinical evaluations. If a study site visit is not possible, a home visit may be arranged to collect the NP sample and conduct clinical evaluations. If a home visit is not possible, the participant will be asked to submit a nasal swab or saliva sample to the study site by a Sponsor-approved method. At this visit, NP swab and blood samples will be collected to evaluate for the presence of SARS-CoV-2 infection. NP swab samples will also be tested for the presence of other respiratory pathogens. In addition, the study site may collect an

additional NP sample for SARS-CoV-2 testing to be able to render appropriate medical care for the study participant as determined by local standards of care. Additionally, clinical information will be carefully collected to evaluate the severity of the clinical case.

5. Sample must be collected prior to dosing on days of injection (Day 1 and Day 29).
6. The participant will record entries in the eDiary approximately 30 minutes after dosing while at the study site, with instruction provided by study staff. Study participants will continue to record in the eDiary each day after they leave the study site, preferably in the evening, on the day of dosing and for 6 days following. Any solicited AR that is ongoing beyond Day 7 will be reported until resolution. Adverse reactions recorded in eDiaries beyond Day 7 should be reviewed either via phone call or at the following study visit. Participants will be given thermometers to record their temperatures and rulers to measure any injection site reactions.
7. Trained study personnel will call all participants to collect information relating to any unsolicited AEs through Day 57 (including any signs and symptoms of COVID-19), MAAEs, AEs leading to withdrawal, SAEs, information on concomitant medications associated with those events, and any non-study vaccinations.
8. All concomitant medications and non-study vaccinations will be recorded through 28 days after each injection; all concomitant medications relevant to or for the treatment of an SAE or MAAE will be recorded from Screening through the final visit (Day 759).
9. All scheduled study visits should be completed within the respective visit windows. If the participant is not able to come on site for a study site visit as a result of the COVID-19 pandemic (self-quarantine or disruption of study site activities following business continuity plans and/or local government mandates for “stay at home” or “shelter in place”), a safety call to the participant should be made in place of the study site visit. The safety call should encompass all scheduled visit assessments that can be completed remotely, such as assessment for AEs and concomitant medications (eg, as defined in scheduled biweekly safety phone calls). Home visits will be permitted for all non-dosing visits except for Screening if a participant cannot come to the study site as a result of the COVID19 pandemic.

Table 14: Schedule of Events (Surveillance Phase, Day 64 – Day 394)

Visit Number				4						5												6
**Type of Visit**	eD	SC	eD	C	eD	SC	eD	SC	eD	C	eD	SC	eD	SC	eD	SC	eD	SC	eD	SC	eD	C
**Month (M)/Weekly (W) Timepoint**	W	M3	W	M4	W	M5	W	M6	W	M7	W	M8	W	M9	W	M10	W	M11	W	M12	W	M13
**Study Visit Day**	Weekly D64 – D78[1]	D85[1]	Weekly D92 – D113[1]	D119 [1,2] 90 days after D29	Weekly D126 – D140[1]	D149[1]	Weekly D154-D168[1]	D179[1]	Weekly D182-D203[1]	D209[1] 180 days after D29	Weekly D216-D230[1]	D239[1]	Weekly D246-D260[1]	D269[1]	Weekly D276-D290[1]	D299[1]	Weekly D306-D320[1]	D329[1]	Weekly D336-D350[1]	D359[1]	Weekly D366 - D387[1]	D394[1] 365 days after D29
**Window Allowance (Days)**	±2	±3	±2	±14	±2	±3	±2	±3	±2	±14	±2	±3	±2	±3	±2	±3	±2	±3	±2	±3	±2	±14
**Days Since Most Recent Vaccination**	-	56	-	90	-	120	-	150	-	180	-	210	-	240	-	270	-	300	-	330	-	365
Physical examination[2]				X						X												X
**Efficacy Assessment**																						
eDiary activation for surveillance for COVID-19/Unscheduled Visit	X																					
Surveillance for COVID-19/Unscheduled Visit[3]	X	X	X	X	X	X	X	X	X	X	X	X	X	X	X	X	X	X	X	X	X	X
**Immunogenicity Assessment**																						
Blood for serologic analysis										X												X
**Safety Assessments**																						
eDiary activation for safety follow-up	X																					
Follow-up Safety[4]	X	X	X	X	X	X	X	X	X	X	X	X	X	X	X	X	X	X	X	X	X	X
Recording of MAAEs, AE leading to withdrawal and concomitant medications relevant to or for the treatment of the MAAE[5]	X	X	X	X	X	X	X	X	X	X	X	X	X	X	X	X	X	X	X	X	X	X

Visit Number				4						5												6
**Type of Visit**	eD	SC	eD	C	eD	SC	eD	SC	eD	C	eD	SC	eD	SC	eD	SC	eD	SC	eD	SC	eD	C
**Month (M)/Weekly (W) Timepoint**	W	M3	W	M4	W	M5	W	M6	W	M7	W	M8	W	M9	W	M10	W	M11	W	M12	W	M13
**Study Visit Day**	Weekly D64 – D78[1]	D85[1]	Weekly D92 – D113[1]	D119 [1,2] 90 days after D29	Weekly D126 – D140[1]	D149[1]	Weekly D154-D168[1]	D179[1]	Weekly D182-D203[1]	D209[1] 180 days after D29	Weekly D216-D230[1]	D239[1]	Weekly D246-D260[1]	D269[1]	Weekly D276-D290[1]	D299[1]	Weekly D306-D320[1]	D329[1]	Weekly D336-D350[1]	D359[1]	Weekly D366 - D387[1]	D394[1] 365 days after D29
**Window Allowance (Days)**	±2	±3	±2	±14	±2	±3	±2	±3	±2	±14	±2	±3	±2	±3	±2	±3	±2	±3	±2	±3	±2	±14
**Days Since Most Recent Vaccination**	-	56	-	90	-	120	-	150	-	180	-	210	-	240	-	270	-	300	-	330	-	365
Recording of SAEs and concomitant medications relevant to or for the treatment of the SAE[5]	X	X	X	X	X	X	X	X	X	X	X	X	X	X	X	X	X	X	X	X	X	X
Recording of concomitant medications and non-study vaccinations[5]	X	X	X	X	X	X	X	X	X	X	X	X	X	X	X	X	X	X	X	X	X	X

Abbreviations: AE = adverse event; C = clinic visit; D = day; eD = electronic diary; ICF = informed consent form; M = month; MAAE = medically attended AE; SAE = serious adverse event; SC = safety (phone) call; W = week.

Note: In accordance with FDA Guidance on Conduct of Clinical Trials of Medical Products during the COVID-19 Pandemic (FDA March 2020), investigators may convert study site visits to telemedicine visits with the approval of the Sponsor.

1. All scheduled study visits should be completed within the respective visit windows. If the participant is not able to come on site for a study site visit as a result of the COVID-19 pandemic (self-quarantine or disruption of study site activities following business continuity plans and/or local government mandates for “stay at home” or “shelter in place”), a safety phone call to the participant should be made in place of the study site visit. The safety call should encompass all scheduled visit assessments that can be completed remotely, such as assessment for AEs and concomitant medications (eg, as defined in scheduled safety phone calls). Home visits will be permitted for all non-dosing visits except for Screening if a participant cannot come to the study site as a result of the COVID-19 pandemic. Home visits must be permitted by the site IRB and the participant via informed consent and have prior approval from the Sponsor (or its designee).
2. Symptom-directed physical examinations may be performed at the discretion of the investigator.
3. Participants meeting prespecified criteria of suspicion for COVID-19 will be asked to return within 72 hours or as soon as possible to the study site for an unscheduled visit, to include an NP swab sample (for RT-PCR testing) and other clinical evaluations. If a study site visit is not possible, a home visit may

be arranged to collect the NP sample and conduct clinical evaluations. If a home visit is not possible, the participant will be asked to submit a nasal swab or saliva sample to the study site by a Sponsor-approved method. At this visit, NP swab and blood samples will be collected to evaluate for the presence of SARS-CoV-2 infection. NP swab samples will also be tested for the presence of other respiratory pathogens. In addition, the study site may collect an additional NP sample for SARS-CoV-2 testing to be able to render appropriate medical care for the study participant as determined by local standards of care. Additionally, clinical information will be carefully collected to evaluate the severity of the clinical case.

4. Trained study personnel will call all participants to collect information relating to any sign/symptoms of COVID-19, MAAEs, AEs leading to withdrawal, SAEs, information on concomitant medications associated with those events, and any non-study vaccinations.
5. All concomitant medications relevant to or for the treatment of an SAE or MAAE will be recorded from Screening through the final visit (Day 759).

Table 15: Schedule of Events (Surveillance Phase, Day 401 – Day 759)

Visit Number																						7
**Type of Visit**	eD	SC	eD	SC	eD	SC	eD	SC	eD	SC	eD	SC	eD	SC	eD	SC	eD	SC	eD	SC	eD	C
**Month (M)/Weekly (W) Timepoint**	W	M14	W	M15	W	M16	W	M17	W	M18	W	M20	W	M21	W	M22	W	M23	W	M24	W	M25
**Study Visit Day**	Weekly D401 – D415[1]	D424[1]	Weekly D431– D445[1]	D454[1]	Weekly D461 – D475[1]	D484[1]	Weekly D491 – D505[1]	D514[1]	Weekly D521-D535[1]	D544[1]	Weekly D551-D593[1]	D604[1]	Weekly D611-D625[1]	D634[1]	Weekly D641-D655[1]	D664[1]	Weekly D671-D685[1]	D694[1]	Weekly D701-D715[1]	D724[1]	Weekly D731 – D752[1]	D759[1] 365 days after Year 1
**Window Allowance (Days)**	±2	±3	±2	±3	±2	±3	±2	±3	±2	±3	±2	±3	±2	±3	±2	±3	±2	±3	±2	±3	±2	±14
**Days Since Most Recent Vaccination**	-		-		-		-		-		-		-		-		-		-		-	730
Physical examination[2]																						X
**Efficacy Assessment**																						
Surveillance for COVID-19/Unscheduled Visit[3]	X	X	X	X	X	X	X	X	X	X	X	X	X	X	X	X	X	X	X	X	X	X
**Immunogenicity Assessment**																						
Blood for serologic analysis																						X
**Safety Assessments**																						
Follow-up Safety[4]	X	X	X	X	X	X	X	X	X	X	X	X	X	X	X	X	X	X	X	X	X	X
Recording of MAAEs, AE leading to withdrawal and concomitant medications relevant to or for the treatment of the MAAE[5]	X	X	X	X	X	X	X	X	X	X	X	X	X	X	X	X	X	X	X	X	X	X
Recording of SAEs and concomitant medications relevant to or for the treatment of the SAE[5]	X	X	X	X	X	X	X	X	X	X	X	X	X	X	X	X	X	X	X	X	X	X
Recording of concomitant medications and non-study vaccinations[5]	X	X	X	X	X	X	X	X	X	X	X	X	X	X	X	X	X	X	X	X	X	X

Abbreviations: AE = adverse event; C = clinic visit; D = day; eD = electronic diary; ICF = informed consent form; M = month; MAAE = medically attended AE; SAE = serious adverse event; SC = safety (phone) call.

Note: In accordance with FDA Guidance on Conduct of Clinical Trials of Medical Products during the COVID-19 Pandemic (FDA March 2020), investigators may convert study site visits to telemedicine visits with the approval of the Sponsor.

1. All scheduled study visits should be completed within the respective visit windows. If the participant is not able to come on site for a study site visit as a result of the COVID-19 pandemic (self-quarantine or disruption of study site activities following business continuity plans and/or local government mandates for “stay at home” or “shelter in place”), a safety phone call to the participant should be made in place of the study site visit. The safety phone call should encompass all scheduled visit assessments that can be completed remotely, such as assessment for AEs and concomitant medications (eg, as defined in scheduled safety phone calls). Home visits will be permitted for all non-dosing visits except for Screening if a participant cannot come to the study site as a result of the COVID-19 pandemic. Home visits must be permitted by the site IRB and the participant via informed consent and have prior approval from the Sponsor (or its designee).
2. Symptom-directed physical examinations may be performed at the discretion of the investigator.
3. Participants meeting prespecified criteria of suspicion for COVID-19 will be asked to return within 72 hours or as soon as possible to the study site for an unscheduled visit, to include an NP swab sample (for RT-PCR testing) and other clinical evaluations. If a study site visit is not possible, a home visit may be arranged to collect the NP sample and conduct clinical evaluations. If a home visit is not possible, the participant will be asked to submit a saliva sample to the study site by a sponsor-approved method. At this visit, NP swab and blood samples will be collected to evaluate for the presence of SARS-CoV-2 infection. NP swabs will also be tested for the presence of other respiratory pathogens. In addition, the study site may collect an additional NP sample for SARS-CoV-2 testing to be able to render appropriate medical care for the study participant as determined by local standards of care. Additionally, clinical information will be carefully collected to evaluate the severity of the clinical case.
4. Trained study personnel will call all participants to collect information relating to any AEs, MAAEs, AEs leading to withdrawal, SAEs, information on concomitant medications associated with those events, and any non-study vaccinations.
5. All concomitant medications relevant to or for the treatment of an SAE or MAAE will be recorded from Screening through the final visit (Day 759).

Table 16: Schedule of Events (28-Day Follow-up After Confirmation of COVID-19)

Unscheduled Visit	1		2		3		4		5
Type of Visit	C		H/C		H/C		H/C		C
Daily Timepoint	D1	D2- D6	D7	D8-D13	D14	D15-D20	D21	D22-D27	D28
**Window Allowance (Days)**	-	±1	±1	±1	±1	±1	±1	±1	+7
**Safety Assessments**									
Physical examination[1]	X								X
Follow-up Safety[2]	X		X		X		X		X
Recording of MAAEs, AE leading to withdrawal and concomitant medications relevant to or for the treatment of the MAAE[3]	X		X		X		X		X
Recording of SAEs and concomitant medications relevant to or for the treatment of the SAE[3]	X		X		X		X		X
Recording of concomitant medications and non-study vaccinations[3]	X		X		X		X		X
**Efficacy Assessments**									
Paper Diary/eDiary Entry[4]	X	X	X	X	X	X	X	X	X
Respiratory Illness Sample[5]	X[6]		X		X		X		X
Blood sample for serologic test for SARS-CoV-2 infection	X								X

Abbreviations: C = clinic visit; D = day; H = home visit; MAAE = medically attended AE; SAE = serious adverse event

Note: In accordance with FDA Guidance on Conduct of Clinical Trials of Medical Products during the COVID-19 Pandemic (FDA March 2020), investigators may convert study site visits to telemedicine visits with the approval of the Sponsor.

1. Physical examination: a full physical examination, including vital signs, height, and weight will be performed initial visit to confirm the diagnosis (denoted as D1 in this table) and the Convalescent Visit (28 days after diagnosis [D28 in this table]).
2. Trained study personnel will call all participants to collect information relating to any AEs, MAAEs, AEs leading to withdrawal, SAEs, information on concomitant medications associated with those events, and any non-study vaccinations. All safety events will be followed until resolution.
3. All concomitant medications relevant to or for the treatment of an SAE or MAAE will be recorded from Screening through the final visit (Day 759).
4. Participants will make daily entries in the paper diary/eDiary, where they will confirm whether they are experiencing symptoms and assess their daily symptoms from onset of COVID-19 symptoms for 28 days following the laboratory confirmation of COVID-19.
5. An NP swab (preferred), nasal swab, or saliva sample will be collected at this visit. The visits on 7, 14, and 21 days after the diagnosis can be performed either at participant's home or at the clinic. The initial visit to confirm the diagnosis (D1 in this table) and the Convalescent Visit (28 days after diagnosis [D28 in this table]) will be a clinic visit.

6. Participants meeting pre-specified criteria of suspicion for COVID-19 will be asked to return within 72 hours or as soon as possible to the study site or medically qualified staff from the site will conduct a home visit as soon as possible to collect an NP swab sample (for RT-PCR), collect a blood sample for serologic assessment of SARS-CoV-2 infection, and to evaluate for COVID-19. NP swab sample will also be tested for the presence of other respiratory infections. In addition, the study site may collect an additional NP sample for SARS-CoV-2 testing to be able to render appropriate medical care for the study participant as determined by local standards of care. Additionally, clinical information will be carefully collected to evaluate the severity of the clinical case.

11.2. APPENDIX 2: Study Governance Considerations

11.2.1. Regulatory and Ethical Considerations

This study will be conducted in accordance with the protocol and with the following:

- Consensus ethical principles derived from international guidelines including the Declaration of Helsinki and Council for International Organizations of Medical Sciences (CIOMS) International Ethical Guidelines.
- Applicable ICH GCP Guidelines.
- Applicable laws and regulatory requirements.
- The protocol, protocol amendments, ICF, Investigator Brochure, and other relevant documents (eg, advertisements) must be submitted to an IRB/IEC by the Investigator and reviewed and approved by the IRB/IEC before the study is initiated.
- Any amendments to the protocol will require IRB/IEC approval before implementation of changes made to the study design, except for changes necessary to eliminate an immediate hazard to study participants.
- The Investigator will be responsible for the following:

– Providing written summaries of the status of the study to the IRB/IEC annually or more frequently in accordance with the requirements, policies, and procedures established by the IRB/IEC

– Notifying the IRB/IEC of SAEs or other significant safety findings as required by IRB/IEC procedures

– Providing oversight of the conduct of the study at the site and adherence to requirements of 21 CFR, ICH guidelines, the IRB/IEC, European regulation 536/2014 for clinical studies (if applicable), and all other applicable local regulations.

11.2.2. Study Monitoring

Before an investigational site can enter a participant into the study, a representative of Moderna or its representatives will visit the investigational study site to:

- Determine the adequacy of the facilities.
- Discuss with the Investigator(s) and other personnel their responsibilities with regard to protocol adherence, and the responsibilities of Moderna or its representatives. This will

be documented in a Clinical Study Agreement between Moderna, designated CRO, and the Investigator.

According to ICH GCP guideline, the Sponsor of the study is responsible for ensuring the proper conduct of the study with regard to protocol adherence and validity of data recorded on the eCRFs. The study monitor's duties are to aid the Investigator and Moderna in the maintenance of complete, accurate, legible, well-organized, and easily retrievable data. The study monitor will advise the Investigator of the regulatory necessity for study-related monitoring, audits, IRB/IEC review, and inspection by providing direct access to the source data/documents. In addition, the study monitor will explain to and interpret for the Investigator all regulations applicable to the clinical evaluation of an IP as documented in ICH guidelines.

It is the study monitor's responsibility to inspect the eCRFs and source documentation throughout the study to protect the rights of the participants; to verify adherence to the protocol; to verify completeness, accuracy, and consistency of the data; and to confirm adherence of study conduct to any local regulations. Details will be outlined in the Clinical Monitoring Plan. During the study, a monitor from Moderna or a representative will have regular contacts with the investigational site, for the following:

- Provide information and support to the Investigator(s).
- Confirm that facilities remain acceptable.
- Confirm that the investigational team is adhering to the protocol, that the data are being accurately recorded in the eCRFs, and that IP accountability checks are being performed.
- Perform source data verification. This includes a comparison of the data in the eCRFs with the participant's medical records at the hospital or practice, and other records relevant to the study. This will require direct access to all original records for each participant (eg, clinical charts or electronic medical record system).
- Record and report any protocol deviations not previously sent.
- Confirm AEs and SAEs have been properly documented on eCRFs and confirm any SAEs have been forwarded to the SAE Hotline, and those SAEs that met criteria for reporting have been forwarded to the IRB/IEC.

The monitor will be available between visits if the Investigator(s) or other staff needs information or advice.

In accordance with FDA Guidance on Conduct of Clinical Trials of Medical Products during the COVID-19 Pandemic (FDA March 2020), investigators may convert study site visits to telemedicine visits with the approval of the Sponsor (Section 8 for Procedures).

The DSMB will also have responsibility for safety monitoring (Section 8.4.1).

11.2.3. Audits and Inspections

Moderna, their designee(s), the IRB/IEC, or regulatory authorities will be allowed to conduct site visits to the investigational facilities for the purpose of monitoring or inspecting any aspect of the study. The Investigator agrees to allow Moderna, their designee(s), the IRB/IEC, or regulatory authorities to inspect the IP storage area, IP stocks, IP records, participant charts and study source documents, and other records relative to study conduct.

Authorized representatives of Moderna, a regulatory authority, and any IRB/IEC may visit the site to perform audits or inspections, including source data verification. The purpose of a Sponsor audit or inspection is to systematically and independently examine all study-related activities and documents to determine whether these activities were conducted, and data were recorded, analyzed, and accurately reported according to the protocol, International Council for Harmonisation Good Clinical Practice (ICH GCP [E6R2]), and any applicable regulatory requirements. The Investigator should contact Moderna immediately if contacted by a regulatory agency about an inspection.

The Principal Investigator must obtain IRB approval for the investigation. Initial IRB approval, and all materials approved by the IRB for this study including the participant consent form and recruitment materials must be maintained by the Investigator and made available for inspection.

11.2.4. Financial Disclosure

The Investigator is required to provide financial disclosure information to allow the Sponsor to submit the complete and accurate certification or disclosure statements required under 21 CFR 54. In addition, the Investigator must provide the Sponsor with a commitment to promptly update this information if any relevant changes occur during the course of the investigation and for 1 year following the completion of the study.

The Sponsor, the CRO, and the study site are not financially responsible for further testing or treatment of any medical condition that may be detected during the screening process. In addition, in the absence of specific arrangements, the Sponsor, the CRO, and the study site are not financially responsible for further treatment of the disease under study.

11.2.5. Recruitment Procedures

Advertisements to be used for the recruitment of study participants, and any other written information regarding this study to be provided to the participant should be submitted to the Sponsor for approval. All documents must be approved by the IRB.

11.2.6. Informed Consent Process

The informed consent document(s) must meet the requirements of 21 CFR 50, local regulations, ICH guidelines, Health Insurance Portability and Accountability Act (HIPAA) requirements, where applicable, and the IRB/IEC or study center. All consent documents will be approved by the appropriate IRB/IEC. The actual ICF used at each center may differ, depending on local regulations and IEC / IRB requirements. However, all versions must contain the standard information found in the sample ICF provided by the Sponsor. Any change to the content of the ICF must be approved by the Sponsor and the IEC / IRB prior to the form being used.

If new information becomes available that may be relevant to the participant's willingness to continue participation in the study, this will be communicated to him / her in a timely manner. Such information will be provided via a revised ICF or an addendum to the original ICF.

The Investigator or his/her representative will explain the nature of the study to the participant and answer all questions regarding the study.

The Investigator is responsible for ensuring that the participant fully understands the nature and purpose of the study. Information should be given in both oral and written form whenever possible.

No participant should be obliged to participate in the study. The participant must be informed that participation is voluntary. Participants, their relatives, guardians, or (if applicable) legal representatives must be given ample opportunity to inquire about details of the study. The information must make clear that refusal to participate in the study or withdrawal from the study at any stage is without any prejudice to the participant's subsequent care.

The participant must be allowed sufficient time to decide whether they wish to participate. The participant must be made aware of and give consent to direct access to his/her source medical records by study monitors, auditors, the IRB/IEC, and regulatory authorities. The participant should be informed that such access will not violate participant confidentiality or any applicable regulations. The participant should also be informed that he/she is authorizing such access by signing the ICF.

A copy of the ICF(s) must be provided to the participant.

A participant who is rescreened is not required to sign another ICF if the rescreening occurs within 28 days from the previous ICF signature date.

The ICF will also explain that excess serum from immunogenicity testing may be used for future research, which may be performed at the discretion of the Sponsor to further characterize the immune response to SARS-CoV-2, additional assay development, and the immune response across CoV.

11.2.7. Protocol Amendments

No change or amendment to this protocol may be made by the investigator or the Sponsor after the protocol has been agreed to and signed by all parties unless such change(s) or amendment(s) has (have) been agreed upon by the investigator or the Sponsor. Any change agreed upon will be recorded in writing, and the written amendment will be signed by the Investigator and the Sponsor. Institutional review board approval is required prior to the implementation of an amendment, unless overriding safety reasons warrant immediate action, in which case the IRB(s)/IEC(s) will be promptly notified.

Any modifications to the protocol or the ICF, which may impact the conduct of the study, potential benefit of the study, or may affect participant safety, including changes of study objectives, study design, participant population, sample sizes, study procedures, or significant administrative aspects will require a formal amendment to the protocol. Such amendment will be released by the Sponsor, agreed by the Investigator(s), and approved by the relevant IRB(s)/IEC(s) prior to implementation. A signed and dated statement that the protocol, any subsequent relevant amended documents and the ICF have been approved by relevant IRB(s)/IEC(s) must be provided to the Sponsor before the study is initiated.

Administrative changes of the protocol are minor corrections and/or clarifications that have no effect on the way the study is to be conducted. These administrative changes will be released by the Sponsor, agreed by the Investigator(s), and notified to the IRB(s)/IEC(s).

11.2.8. Protocol Deviations

The noncompliance may be either on the part of the participant, the Investigator, or the study site staff. As a result of deviations, corrective actions are to be developed by the site and implemented promptly.

It is the responsibility of the site Investigator to use continuous vigilance to identify and report deviations to the Sponsor or its designee. All deviations must be addressed in study source documents, reported to study monitor. Protocol deviations must be sent to the reviewing IRB/IEC per their policies. The site Investigator is responsible for knowing and adhering to the reviewing IRB/IEC requirements.

11.2.9. Data Protection

Participants will be assigned a unique identifier by the Sponsor. Any participant records or datasets that are transferred to the Sponsor will contain the identifier only; participant names or any information which would make the participant identifiable will not be transferred.

The participant must be informed that his/her personal study-related data will be used by the Sponsor in accordance with local data protection law. The level of disclosure must also be explained to the participant.

The participant must be informed that his/her medical records may be examined by Clinical Quality Assurance auditors or other authorized personnel appointed by the Sponsor, by appropriate IRB/IEC members, and by inspectors from regulatory authorities.

Individual participant medical information obtained as a result of this study is considered confidential, and disclosure to third parties is prohibited. Information will be accessible to authorized parties or personnel only. Medical information may be given to the participant's physician or to other appropriate medical personnel responsible for the participant's well-being. Each participant will be asked to complete a form allowing the Investigator to notify the participant's primary health care provider of his/her participation in this study.

All laboratory specimens, evaluation forms, reports, and other records will be identified in a manner designed to maintain participant confidentiality. All records will be kept in a secure storage area with limited access. Clinical information will not be released without the written permission of the participant, except as necessary for monitoring and auditing by the Sponsor, its designee, relevant regulatory authority, or the IRB.

The Investigator and all employees and coworkers involved with this study may not disclose or use for any purpose other than performance of the study, any data, record, or other unpublished, confidential information disclosed to those individuals for the purpose of the study. Prior written agreement from the Sponsor or its designee must be obtained for the disclosure of any confidential information to other parties.

11.2.10. Sample Retention and Future Biomedical Research

The retention period of laboratory samples will be 15 years, or as permitted by local regulations, to address further scientific questions related to mRNA-1273 or anti-respiratory virus immune response. In addition, identifiable samples can be destroyed at any time at the request of the participant. During the study, or during the retention period, in addition to the analysis outlined in the study endpoints, exploratory analysis may be conducted, using other antibody-based methodologies, on any remaining blood or serum samples, including participants who provide samples for screening, but are not subsequently enrolled. These analyses would extend the search for other potentially relevant biomarkers to investigate the effect of mRNA-1273, as well as to determine how changes in the markers may relate to exposure and clinical outcomes. A decision to perform such exploratory research may arise from new scientific findings related to the drug class or disease, as well as reagent and assay availability.

11.2.11. Data Quality Assurance and Quality Control

Data collection is the responsibility of the clinical study staff at the site under the supervision of the site Investigator. The Investigator is responsible for ensuring the accuracy, completeness, legibility, and timeliness of the data reported.

- All participant data relating to the study will be recorded electronic CRF (eCRF) unless transmitted to the Sponsor or designee electronically (eg, laboratory data). The Investigator is responsible for verifying that data entries are accurate and correct by physically or electronically signing the eCRF.
- The Investigator must maintain accurate documentation (source data) that supports the information entered in the eCRF.
- The Investigator must permit study-related monitoring, audits, IRB/IEC review, and regulatory agency inspections and provide direct access to source data documents.
- Monitoring details describing strategy (eg, risk-based initiatives in operations and quality such as Risk Management and Mitigation Strategies and Analytical Risk-Based Monitoring), methods, responsibilities and requirements, including handling of noncompliance issues and monitoring techniques (central, remote, or on-site monitoring) are provided in the study Monitoring Plan, Centralized Monitoring Plan, and Risk Management Plan.
- The Sponsor or designee is responsible for the data management of this study including quality checking of the data.
- The Sponsor assumes accountability for actions delegated to other individuals (eg, Contract Research Organizations).
- Study monitors will perform ongoing source data verification to confirm that data entered into the CRF by authorized site personnel are accurate, complete, and verifiable from source documents; that the safety and rights of participants are being protected; and that the study is being conducted in accordance with the currently approved protocol and any other study agreements, ICH GCP, and all applicable regulatory requirements.
- Records and documents, including signed ICFs, pertaining to the conduct of this study must be retained by the Investigator for 15 years after study completion unless local regulations or institutional policies require a longer retention period. No records may be destroyed during the retention period without the written approval of the

Sponsor. No records may be transferred to another location or party without written notification to the Sponsor.

Quality assurance (QA) includes all the planned and systematic actions that are established to ensure that the clinical study is performed, and the data are generated, documented (recorded), and reported according to ICH GCP and local/regional regulatory standards.

A QA representative from the Sponsor or qualified designee, who is independent of and separated from routine monitoring, may periodically arrange inspections/audits of the clinical study by reviewing the data obtained and procedural aspects. These inspections may include on-site inspections/audits and source data checks. Direct access to source documents is required for the purpose of these periodic inspections/audits.

11.2.12. Data Collection and Management

This study will be conducted in compliance with ICH CGP guidelines. This study will also be conducted in accordance with the most recent version of the Declaration of Helsinki.

This study will use electronic data collection (to collect data directly from the investigational site using eCRFs). The Investigator is responsible for ensuring that all sections of each eCRF are completed promptly and correctly and that entries can be verified against any source data.

Study monitors will perform source document verification to identify inconsistencies between the eCRFs and source documents. Discrepancies will be resolved in accordance with the principles of GCP. Detailed study monitoring procedures are provided in the Clinical Monitoring Plan.

AEs will be coded with the most current available version of MedDRA. Concomitant medications will be coded using WHO – Drug Reference List.

11.2.13. Source Documents

Source documents are original documents or certified copies, and include, but are not limited to, diaries, medical and hospital records, screening logs, informed consent / assent forms, telephone contact logs, and worksheets. Source documents provide evidence for the existence of the participant and substantiate the integrity of the data collected. Source documents are filed at the Investigator's site.

Data reported on the CRF or entered in the eCRF that are transcribed from source documents must be consistent with the source documents or the discrepancies must be explained. The Investigator may need to request previous medical records or transfer records, depending on the study. Also, current medical records must be available.

Moderna or its designee requires that the Investigator prepare and maintain adequate and accurate records for each participant treated with the IP. Source documents such as any hospital, clinic, or

office charts and the signed ICFs are to be included in the Investigator's files with the participant's study records.

11.2.14. Retention of Records

The Principal Investigator must maintain all documentation relating to the study for a period of at least 2 years after the last marketing application approval or, if not approved, 2 years following the discontinuance of the test article for investigation. If this requirement differs from any local regulations, the local regulations will take precedence unless the local retention policy is less than 2 years.

If it becomes necessary for Moderna or the regulatory authority to review any documentation relating to the study, the Investigator must permit access to such records. No records will be destroyed without the written consent of the Sponsor, if applicable. It is the responsibility of the Sponsor to inform the Investigator when these documents no longer need to be retained.

11.2.15. Study and Site Closure

If the study is prematurely terminated or suspended, the Sponsor shall promptly inform the investigators, the IECs/IRBs, the regulatory authorities, and any CRO(s) used in the study of the reason for termination or suspension, as specified by the applicable regulatory requirements. The Investigator shall promptly inform the participant and should assure appropriate participant therapy and/or follow-up.

The Sponsor or designee reserves the right to close the study site or terminate the study at any time for any reason at the sole discretion of the Sponsor.

The Investigator may initiate study-site closure at any time, provided there is reasonable cause and sufficient notice is given in advance of the intended termination.

Reasons for the early closure of a study site by the Sponsor or Investigator may include but are not limited to:

- Continuation of the study represents a significant medical risk to participants.
- Failure of the Investigator to comply with the protocol, the requirements of the IRB/IEC or local health authorities, the Sponsor's procedures, or GCP guidelines.
- Inadequate recruitment of participants by the Investigator.
- Discontinuation of further mRNA-1273 development.

Study sites will be closed upon study completion. A study site is considered closed when all required documents and study supplies have been collected and a study-site closure visit has been performed.

11.2.16. Publication Policy

The results of this study may be published or presented at scientific meetings. If this is foreseen, the Investigator agrees to submit all manuscripts or abstracts to the Sponsor before submission. This allows the Sponsor to protect proprietary information and to provide comments.

The Sponsor will comply with the requirements for publication of study results. In accordance with standard editorial and ethical practice, the Sponsor will generally support publication of multicenter studies only in their entirety and not as individual site data. In this case, a coordinating investigator will be designated by mutual agreement.

Authorship will be determined by mutual agreement and in line with International Committee of Medical Journal Editors authorship requirements.

The clinical study plan and the results of the study will be published on www.ClinicalTrials.gov in accordance with 21 CFR 50.25(c). The results of and data from this study belong to Moderna.

11.3. APPENDIX 3: Contraceptive Guidance

Woman of Childbearing Potential (WOCBP)

Females of childbearing potential are those who are considered fertile following menarche and until becoming postmenopausal unless permanently sterile (see below). If fertility is unclear (eg, amenorrhea in adolescents or athletes) and a menstrual cycle cannot be confirmed before first dose of study treatment, additional evaluation should be considered.

Women in the following categories are not considered WOCBP:

1. Premenarchal
2. Premenopausal, surgically sterile female with 1 of the following:
   a. Documented complete hysterectomy
   b. Documented bilateral salpingectomy
   c. Documented bilateral oophorectomy

For individuals with permanent infertility due to an alternate medical cause other than the above, (eg, mullerian agenesis, androgen insensitivity), investigator discretion should be applied in determining study entry.

Note: Documentation can come from the site personnel's: review of the participant's medical records, medical examination, or medical history interview.

3. Postmenopausal female

- A postmenopausal state is defined as no menses for 12 months without an alternative medical cause. The following age-specific requirements apply:
  - Women <50 years of age would be considered postmenopausal if they have been amenorrheic for 12 months or more following cessation of exogenous hormonal treatments and if they have luteinizing hormone and FSH levels in the postmenopausal range for the institution.
  - Women ≥ 50 years of age would be considered postmenopausal if they have been amenorrheic for 12 months or more following cessation of all exogenous hormonal treatments, had radiation-induced menopause with last menses >1 year ago, had chemotherapy-induced menopause with last menses >1 year ago.
- A high FSH level in the postmenopausal range may be used to confirm a postmenopausal state in women not using hormonal contraception or hormonal replacement therapy (HRT).

- Females on HRT and whose menopausal status is in doubt will be required to use one of the non-estrogen hormonal highly effective contraception methods if they wish to continue their HRT during the study. Otherwise, they must discontinue HRT to allow confirmation of postmenopausal status before study enrollment.

11.4. APPENDIX 4 - Statistical Appendices

11.4.1. Estimands and Estimand Specifications

Table 17: Intercurrent Event Types

Label	Intercurrent Event Type	Comment
IcEv1 (early discontinuation or death without confirmation of cases, ie, unrelated death)	Early discontinuation from study preventing from confirmation of COVID-19, ie, unrelated death or withdrawal consent prior to documented confirmed COVID-19	Participants who withdrawal consent or die due to unrelated to COVID-19 without confirmation of being a case will all be included in statistical analysis.
IcEv2 (early infection)	Infection starting up to 14 days after the second dose of IP	Participants who experience an early infection up to 14 days after the second dose of IP will all be included in statistical analysis.
IcEv3 (missing dose of IP)	Not receiving the second dose of IP per protocol schedule	Participants who miss a dose of IP will be excluded from the PP, but in the FAS and the mITT.
IcEv4 (seropositive at baseline)	Participants with seropositive status at baseline	Participants who were seropositive at baseline will be excluded from the PP and the mITT but included in the FAS.

Abbreviation: IcEv: intercurrent event, PP: per protocol, FAS: full analysis set, mITT: modified intent-to-treat.

Table 18: Primary Objective and Estimands with Rationale for Strategies to Address Intercurrent Events for Per Protocol Analysis

**Objective: To demonstrate the efficacy of mRNA-1273 to prevent COVID-19**	
**Estimand Description**	Vaccine efficacy will be measured using 1 – HR (mRNA-1273/Placebo) of COVID-19 from 14 days after second dose of IP in adults. A treatment policy strategy will be used for early discontinuation (eg, withdrawal consent, deaths unrelated to COVID-19) or early infection. A principal stratum strategy is used to exclude participants missing a dose of IP or being seropositive at baseline.
**Target Population**	Adults aged 18 years and older in circumstances at a high risk of SARS-CoV-2 infection but without medical conditions that pose additional risk of developing severe disease.<br>The population excludes those previously infected or vaccinated for SARS-CoV-2 or with a medical condition, on treatment that poses additional risks (including those requiring immunosuppressants or immune-modifying drugs), or pre-seropositive.
**Variable/Endpoint**	Time to infection, censoring at early discontinuation, early infection, or last assessment for an event not being observed, whichever comes earlier.
**Treatment Condition(s)**	**Test:** mRNA-1273<br>**Reference:** Placebo
**Estimand Label**	**Estimand 1**
**Population-Level Summary**	Vaccine efficacy defined as 1 - HR of mRNA-1273/Placebo
**Intercurrent Event Strategy**	
**IcEv1 (Early discontinuation):**	Treatment policy
**IcEv2 (early infection):**	Treatment policy
**IcEv3 (Missed dose of IP):**	Principal stratum

**Objective: To demonstrate the efficacy of mRNA-1273 to prevent COVID-19**			
**IcEv4 (Seropositive at baseline):**	Principal stratum	N/A	N/A
**Rationale for Strategy(s)**	Treatment policy: early discontinuation censored at time of discontinuation; early case will be censored at the time at confirmation<br>Principal stratum: Participants who were seropositive at baseline or missed dose of IP are excluded from this Estimand		

11.4.2. Statistical Methods and Sensitivity Analyses

Table 19: Summary of Statistical Methods and Sensitivity Analyses

Estimand Label	Estimand Description	Main Estimation			Sensitivity Analysis
		**Analysis Set**	**Imputation/Data/Censoring Rules**	**Analysis Model/Method**	
Estimand 1	See in Table 18	PP	Participants who did not develop COVID-19, ongoing or complete the study will be censored at the last assessment.<br>Participants who discontinue early or die without COVID-19 will be censored at the date of discontinuation or death respectively.<br>Participants who is a confirmed case prior to 14 days after the second dose of IP will be censored on the date of confirmation.	The (1 - HR) with 95% CI will be estimated using Cox proportional hazard regression analysis with treatment arm as a fixed effect stratified by randomization stratification factors. And the null hypothesis will be tested using Wald Chi-square method.	As described in Section 9.5.1 with cases counted starting after the second dose of IP

Signature Page for VV-CLIN-000828 v2.0

Approval	Conor Knightly<br>Clinical<br>16-Jun-2020 16:52:59 GMT+0000

Approval	Charbel Haber<br>Regulatory<br>16-Jun-2020 16:56:35 GMT+0000

Approval	Tal Zaks<br>Clinical<br>16-Jun-2020 17:12:48 GMT+0000

Approval	Brett Leav<br>Clinical<br>16-Jun-2020 17:25:43 GMT+0000

Signature Page for VV-CLIN-000828 v2.0

CLINICAL STUDY PROTOCOL

**Protocol Title:**	A Phase 3, Randomized, Stratified, Observer-Blind, Placebo-Controlled Study to Evaluate the Efficacy, Safety, and Immunogenicity of mRNA-1273 SARS-CoV-2 Vaccine in Adults Aged 18 Years and Older
**Protocol Number:**	mRNA-1273-P301
**Sponsor Name:**	ModernaTX, Inc.
**Legal Registered Address:**	200 Technology Square<br>Cambridge, MA 02139
**Sponsor Contact and Medical Monitor:**	Allison August, MD<br>VP, Clinical Development<br>ModernaTX, Inc.<br>200 Technology Square, Cambridge, MA 02139<br>Telephone: 1-617-678-8937<br>e-mail: Allison.August@modernatx.com
**Regulatory Agency Identifier Number(s):**	IND: 19745
**Amendment Number:**	8
**Date of Amendment 8:**	23 Mar 2021
**Date of Amendment 7:**	10 Feb 2021
**Date of Amendment 6:**	23 Dec 2020
**Date of Amendment 5:**	11 Nov 2020
**Date of Amendment 4:**	30 Sep 2020
**Date of Amendment 3:**	20 Aug 2020
**Date of Amendment 2:**	31 Jul 2020
**Date of Amendment 1:**	26 Jun 2020
**Date of Original Protocol:**	15 Jun 2020

CONFIDENTIAL

All financial and nonfinancial support for this study will be provided by ModernaTX, Inc. The concepts and information contained in this document or generated during the study are considered proprietary and may not be disclosed in whole or in part without the expressed written consent of ModernaTX, Inc. The study will be conducted according to the *International Council for Harmonisation (ICH) Technical Requirements for Registration of Pharmaceuticals for Human Use, E6(R2) Good Clinical Practice (GCP) Guidance.*

Protocol Approval – Sponsor Signatory

Study Title: A Phase 3, Randomized, Stratified, Observer-Blind, Placebo-Controlled Study to Evaluate the Efficacy, Safety, and Immunogenicity of mRNA-1273 SARS-CoV-2 Vaccine in Adults Aged 18 Years and Older

Protocol Number: mRNA-1273-P301

Protocol Version Date: 23 Mar 2021

Protocol accepted and approved by:

See signature and date signed on last page of document

Allison August, MD
VP, Clinical Development
ModernaTX, Inc.
200 Technology Square
Cambridge, MA 02139
Telephone: 1-617-678-8937

Date

DECLARATION OF INVESTIGATOR

I have read and understood all sections of the protocol entitled "A Phase 3, Randomized, Stratified, Observer-Blind, Placebo-Controlled Study to Evaluate the Efficacy, Safety, and Immunogenicity of mRNA-1273 SARS-CoV-2 Vaccine in Adults Aged 18 Years and Older" and the most recent version of the Investigator's Brochure.

I agree to supervise all aspects of the protocol and to conduct the clinical investigation in accordance with the current Protocol, the *International Council for Harmonisation (ICH) Technical Requirements for Registration of Pharmaceuticals for Human Use, E6(R2) Good Clinical Practice (GCP) Guidance,* and all applicable government regulations. I will not make changes to the protocol before consulting with ModernaTX, Inc. or implement protocol changes without IRB/IEC approval except to eliminate an immediate risk to participants.

I agree to administer study treatment only to participants under my personal supervision or the supervision of a sub-investigator. I will not supply study treatment to any person not authorized to receive it. I also agree that persons debarred from conducting or working on clinical studies by any court or regulatory agency will not be allowed to conduct or work on studies for the Sponsor or a partnership in which the Sponsor is involved. I will immediately disclose it in writing to the Sponsor if any person who is involved in the study is debarred, or if any proceeding for debarment is pending, or, to the best of my knowledge, threatened.

I will not disclose confidential information contained in this document including participant information, to anyone other than the recipient study staffs and members of the IRB/IEC. I agree to ensure that this information will not be used for any purpose other than the evaluation or conduct of the clinical investigation without the prior written consent from ModernaTX, Inc. I will not disclose information regarding this clinical investigation or publish results of the investigation without authorization from ModernaTX, Inc.

The signature below provides the necessary assurance that this study will be conducted according to all stipulations of the protocol, including statements regarding confidentiality, and according to local legal and regulatory requirements, US federal regulations, and ICH E6(R2) GCP guidelines.

______________________________ ______________

Signature of Principal Investigator Date

____

Printed Name of Principal Investigator

Protocol Amendment Summary of Changes

DOCUMENT HISTORY	
**Document**	**Date**
Amendment 8	23 Mar 2021
Amendment 7	10 Feb 2021
Amendment 6	23 Dec 2020
Amendment 5	11 Nov 2020
Amendment 4	30 Sep 2020
Amendment 3	20 Aug 2020
Amendment 2	31 Jul 2020
Amendment 1	26 Jun 2020
Original Protocol	15 Jun 2020

Amendment 8, 23 Mar 2021

Main Rationale for the Amendment:

The purpose of this amendment is to update language around unblinding and open-label dosing in the context of the majority of participants already having completed their blinded follow-up. In addition, the amendment updates the reporting of serious COVID-19 cases.

The summary of changes table provided here describes the major changes made in Amendment 8 relative to Amendment 7, including the sections modified and the corresponding rationales. As applicable, the synopsis of Amendment 8 has been modified to correspond to changes in the body of the protocol.

Summary of Major Changes from Protocol Amendment 7 to Protocol Amendment 8:

Section # and Name	Description of Change	Brief Rationale
Section 4.1.2 (Part B, the Open-label Observational Phase)	States that, due to ethical and scientific concerns, all participants will be unblinded once investigational product is no longer available. In addition, the text indicates that if participants want to receive emergency use authorization (EUA) vaccine outside of the study, participants will need to be withdrawn from the study.<br><br>The amendment also provides directions for unblinding after Biologics License Application (BLA) database lock.	At the time of this amendment, more than 95% of the enrolled participants have completed blinded follow-up, either through being unblinded or study discontinuation. Therefore, there is no scientific value for participants to remain blinded.<br><br>In addition, non-study EUA vaccines are widely available, so ethically, participants should be unblinded to ensure they can make an informed choice if they wish to receive EUA COVID-19 vaccine outside of the study.

Section # and Name	Description of Change	Brief Rationale
Section 8.3.7 (Time Period and Frequency for collecting Adverse Events and Serious Adverse Event Information) and Section 8.3.10 (Reporting Adverse Events)	Reporting of only serious COVID-19 cases within 24 hours to sponsor or it's designee.	Non-serious COVID-19 cases are an efficacy endpoint, so reporting to Pharmacovigilance is no longer warranted.

IRB and Regulatory Authority Approval

A copy of this amended protocol will be sent to the institutional review board (IRB) and regulatory authority.

The changes described in this amended protocol require IRB approval prior to implementation. In addition, if the changes herein affect the informed consent, sites are required to update and submit a revised informed consent for approval that incorporates the changes described in this amended protocol.

1. PROTOCOL SUMMARY

1.1. Synopsis

**Protocol Number:**	**mRNA-1273-P301**
**Title:**	A Phase 3, Randomized, Stratified, Observer-Blind, Placebo-Controlled Study to Evaluate the Efficacy, Safety, and Immunogenicity of mRNA-1273 SARS-CoV-2 Vaccine in Adults Aged 18 Years and Older.
**Study Phase:**	3
**Objectives:**	Primary:

- To demonstrate the efficacy of mRNA-1273 to prevent COVID-19.
- To evaluate the safety and reactogenicity of 2 injections of mRNA-1273 given 28 days apart.

Secondary:

- To evaluate the efficacy of mRNA-1273 to prevent severe COVID-19.
- To evaluate the efficacy of mRNA-1273 to prevent serologically confirmed SARS-CoV-2 infection or COVID-19 regardless of symptomatology or severity.
- To evaluate vaccine efficacy (VE) against a secondary definition of COVID-19.
- To evaluate VE to prevent death caused by COVID-19.
- To evaluate the efficacy of mRNA-1273 to prevent COVID-19 after the first dose of investigational product (IP).
- To evaluate the efficacy of mRNA-1273 to prevent COVID-19 in all study participants, regardless of evidence of prior SARS-CoV-2 infection.
- To evaluate the efficacy of mRNA-1273 to prevent asymptomatic SARS-CoV-2 infection.

	Exploratory:<br>• To evaluate the effect of mRNA-1273 on the viral infection kinetics as measured by viral load at SARS-CoV-2 infection diagnosis by RT-PCR and number of days from the estimated date of SARS-CoV-2 infection until undetectable SARS-CoV-2 infection by RT-PCR.<br>• To assess VE to reduce the duration of symptoms of COVID-19.<br>• To evaluate VE against all-cause mortality.<br>• To assess VE against burden of disease (BOD) due to COVID-19.<br>• To evaluate the genetic and/or phenotypic relationships of isolated SARS-CoV-2 strains to the vaccine sequence.<br>• To evaluate immune response markers after dosing with IP as correlates of risk of COVID-19 and as correlates of risk of SARS-CoV-2 infection.<br>• To conduct additional analyses related to furthering the understanding of SARS-CoV-2 infection and COVID-19, including analyses related to the immunology of this or other vaccines, detection of viral infection, and clinical conduct.
**Study Design and Methodology:**	This is a two-part Phase 3 study: Part A and Part B (Figure 1). Participants in Part A, the Blinded Phase of this study are blinded to their treatment assignment. Given that the primary efficacy endpoint for mRNA-1273 against COVID-19 was met per the protocol-defined interim analysis (IA), Part B, the Open-Label Observational Phase of this study is designed to offer participants who received placebo in Part A of this study, an option to request Open-label mRNA-1273 while investigational vaccine is still available. With regards to eligibility in Part B, the CDC-EUA guidance specifications will supersede eligibility criteria in the protocol.<br>**Part A, the Blinded Phase**:<br>The Blinded Phase of this study (Figure 1) is a randomized, stratified, observer-blind, placebo-controlled evaluation of the efficacy, safety, and immunogenicity of mRNA-1273 SARS-CoV-2 vaccine compared to placebo in adults 18 years of age and older who have no known history of SARS-CoV-2 infection but whose locations or circumstances put them at appreciable risk of acquiring COVID-19 and/or SARS-CoV-2 infection.

Participants will be randomly assigned to receive injections of either 100 µg of mRNA-1273 vaccine or a placebo control in a 1:1 randomization ratio. Assignment will be stratified by age and health risk. This is a case-driven study and thus final sample size of the study will depend on the actual attack rate of COVID-19.

All participants will be assessed for efficacy and safety endpoints and provide a nasopharyngeal (NP) swab sample and blood sample before the first and second dose of IP, in addition to a series of post-dose blood samples for immunogenicity through 24 months after the second dose of IP. Efficacy assessments will include surveillance for COVID-19 with RT-PCR confirmation of SARS-CoV-2 infection after the first and second dose of IP.

As noted above, this is a case-driven study: if the prespecified criteria for early efficacy are met at the time of either IA or overall efficacy at the primary analysis, a final study report describing the efficacy and safety of mRNA-1273 will be prepared based on the data available at that time. In the event that success criteria are met either at the time of the IA or when the total number of cases toward the primary endpoint have accrued, participants will continue to be followed in a blinded fashion until Month 25 to enable assessment of long-term safety and durability of VE.

If the study concludes early, all participants will be requested to provide a final blood sample at the time of study conclusion.

Each participant will receive 2 doses of IP by 0.5 mL intramuscular (IM) injection, the first on Day 1 and the second on Day 29. An NP swab sample will be collected prior to the first and second dose of IP, for evaluation by RT-PCR. Participants will be given an electronic diary (eDiary) to report solicited adverse reactions (ARs) for 7 days after each dose of IP (Part A, Blinded Phase only) and to prompt an unscheduled clinic visit for clinical evaluation and NP swab sample if a participant experiences any symptoms of COVID-19. All participants will receive safety calls on Day 8, Day 15, Day 22, Day 36, and Day 43 that will serve both to monitor for unsolicited adverse events (AEs) and to monitor for symptoms of COVID-19.

Surveillance for COVID-19 will be performed through weekly contacts with the participant via a combination of telephone calls and completion of an eDiary starting at Day 1 through the end of the study.

Participants with symptoms of COVID-19 lasting at least 48 hours (except for fever and/or respiratory symptoms) will return to the

clinic or will be visited at home by medically qualified site staff within 72 hours (an "Illness Visit") to collect an NP swab sample for RT-PCR testing for SARS-CoV-2 and other respiratory pathogens, or alternatively, if a clinic or home visit is not possible, will submit a saliva (or nasal swab) sample for SARS-CoV-2 RT-PCR testing. Any confirmed COVID-19 occurring in a participant will be captured as a medically attended adverse event (MAAE) along with relevant concomitant medications and details about severity, seriousness, and outcome. All confirmed serious COVID-19 cases will be reported to the Sponsor or designee within 24 hours.

Starting with the Illness Visit, study participants will be monitored by the study investigator (or appropriately delegated study staff) for a 14-day period after diagnosis or until symptoms resolve, whichever is later. Each participant diagnosed with COVID-19 will monitor their body temperature, oxygen saturation, and symptoms following the diagnosis of COVID-19. In addition to daily follow-up of symptoms, assessments will include the collection of saliva samples during the 28-day period following the diagnosis of SARS-CoV-2 infection. The investigator will determine if medical attention is required due to worsening of COVID-19. Finally, a convalescent visit will be scheduled approximately 28 days after the initial Illness Visit. At this visit, a saliva (or nasal swab) sample will be collected, and a blood sample will be drawn for immunologic assessment of SARS-CoV-2 infection.

The 28-day period following the Illness Visit is referred to as the Convalescent Period. If during the Convalescent Period the participant has a positive result for SARS-CoV-2 from the Illness Visit, the participant will continue the Convalescent Period. If the participant has a negative result for SARS-CoV-2 from the Illness Visit, the participant will exit the Convalescent Period, including discontinuation of daily telemedicine visits and collection of saliva (or nasal swab) samples, and will return to their respective study schedule.

At each dosing visit, participants will be instructed (Day 1) or reminded (Day 29) on how to document and report solicited ARs in the eDiary provided. Solicited ARs will be assessed for 7 days after each IP dose (Part A, the Blinded Phase only) and unsolicited AEs will be assessed for 28 days after each IP dose (Part A, the Blinded Phase only); serious adverse events (SAEs), MAAEs, AEs leading to withdrawal, will be assessed throughout the study.

Part B, the Open-Label Observational Phase:

Part B, the Open-Label Observational Phase of the study is prompted by the authorization of a COVID-19 vaccine under EUA. Transitioning the study to Part B permits all ongoing study participants to be informed of the availability and eligibility criteria of any COVID-19 vaccine made available under an EUA and the option to offer all ongoing study participants who request unblinding, an opportunity to schedule a Participant Decision clinic visit to know their original treatment assignment (placebo vs. mRNA-1273 vaccine).

Part B, the Open-Label Observational Phase of the study, also provides the opportunity for eligible study participants who previously received placebo to actively request to receive 2 doses of mRNA-1273 vaccine (as long as investigational vaccine is available). All study participants will receive a Notification Letter and will be asked to schedule a Participant Decision clinic visit. Principal Investigators should consider current local public health guidance for administration of COVID-19 vaccines under EUA when determining the scheduling priority of participants.

All participants will proceed to Part B, the Open-Label Observational Phase of the study, starting with a Participant Decision clinic visit (Figure 2).

At the Participant Decision clinic visit, all participants will:

- Be encouraged to remain in the ongoing study,
- Be given the option to be unblinded as to their original group assignment (placebo vs. mRNA-1273 vaccine),
- Be counselled about the importance of continuing other public health measures to limit the spread of disease including social distancing, wearing a mask, and hand-washing,
- Sign a revised informed consent form,
- Provide a NP swab for RT-PCR for SARS-CoV-2 and a blood sample for serology prior to unblinding.

The following participants will continue with the original study Schedule of Events (SoEs) presented in Table 16, Table 17, Table 18, and Table 19 (Figure 2):

- participants who request to not be unblinded,
- participants who request to be unblinded and received mRNA-1273, and
- participants who request to be unblinded and received placebo and choose to remain on placebo.

Participants who are unblinded, received placebo, are eligible, and request to receive mRNA-1273 (Figure 2) will have the following clinic visits as shown in the Supplemental SoE (Table 21):

- Open-label Dose 1 (OL-D1): To occur at the Participant Decision clinic visit or at a scheduled subsequent visit.
- Open-label Dose 2 (OL-D29): To occur 28 days after Dose 1 on OL-D1.
- Open-label Clinic Visit (OL-D57): To occur 1-month after Dose 2 on OL-D29.
- in addition to continuing to comply with the Original Study Schedule of Events (Table 16, Table 17, Table 18 and Table 19, as applicable).

Participants who are unblinded, received ONLY 1 dose of mRNA-1273, are eligible, and request to receive mRNA-1273 (Figure 2) will have the following clinic visits as shown in the Modified Supplemental Schedule of Events (Table 22):

- Their second dose on OL-D1: To occur at the Participant Decision clinic visit or at a scheduled subsequent visit
- And return on OL-D29: To occur 1-month after Dose 2 on OL-D1.
- in addition to continuing to comply with the Original Study Schedule of Events (Table 16, Table 17, Table 18, and Table 19, as applicable).

All participants remain on their original SoE to complete 24 months of follow-up after Dose 2; study participants who enter the Supplemental/Modified Supplemental SoE as described above do so in addition to their original SoE. Accordingly, all study participants will complete the full study follow-up to 24 months after the second dose of their original inoculation (mRNA-1273 or placebo).

	At a point when the mRNA-1273 vaccine is no longer available for study use, any participant who schedules a Participant Decision Visit after this time-point will be unblinded (with no option to stay blinded), but will not be offered to receive mRNA-1273 study vaccine. These participants will have all of the same procedures performed outlined in the SoE table, with the exception of being offered to receive mRNA-1273 vaccine..<br><br>After the Biologics License Application (BLA) database lock, any remaining participants who have not been unblinded will be unblinded by the Moderna study team and will receive their unblinding treatment assignment (placebo vs. mRNA vaccine) via certified letter from the site..<br><br>This study will be conducted in compliance with the protocol, Good Clinical Practice (GCP), and all applicable regulatory requirements.
**Randomization:**	In Part A, the Blinded Phase of the study, approximately 30,000 participants will be randomly assigned in 1:1 ratio to receive either mRNA-1273 100 µg or placebo. The randomization will be in a blinded manner using a centralized Interactive Response Technology, in accordance with pre-generated randomization schedules.
**Stratification:**	Randomization in Part A, the Blinded Phase of the study, will be stratified based on age and, if they are < 65 years of age, based on the presence or absence of risk factors for severe illness from COVID-19 based on CDC recommendation as of March 2020. There will be 3 strata for randomization: ≥ 65 years, < 65 years and categorized to be at increased risk (“at risk”) for the complications of COVID-19, and < 65 years “not at risk”. Risk will be defined based on the study participants’ relevant past and current medical history. At least 25% of enrolled participants, up to 50%, will be either ≥ 65 years of age or < 65 years of age and “at risk” at Screening.<br><br>Participants who are < 65 years old will be categorized as at risk for severe COVID-19 illness if they have at least 1 of the following risk factors at Screening:<br><br>• Chronic lung disease (eg, emphysema and chronic bronchitis, idiopathic pulmonary fibrosis, and cystic fibrosis) or moderate to severe asthma

- Significant cardiac disease (eg, heart failure, coronary artery disease, congenital heart disease, cardiomyopathies, and pulmonary hypertension)
- Severe obesity (body mass index ≥ 40 kg/m^2)
- Diabetes (Type 1, Type 2 or gestational)
- Liver disease
- Human Immunodeficiency Virus (HIV) infection

Study Population:

Participants (males and females 18 years of age or older at time of consent), who are at risk of SARS-CoV-2 infection with no known history of SARS-CoV-2 infection, are a subset of the planned target population. Additionally, potential study participants at increased risk of complications from COVID-19 will be included, since it is hypothesized that these participants might derive the greatest benefit from a vaccine. Participants ≥ 65 years of age will be eligible for enrollment with or without underlying medical conditions further increasing their risk of severe COVID-19.

Study sites may be selected based on SARS-COV-2 infection risk of the local population. Approximately 30,000 participants will be enrolled.

The full lists of inclusion and exclusion criteria are provided in the body of the protocol.

Efficacy Assessments:

Primary Efficacy Assessment:

To be considered as a case of COVID-19 for the evaluation of the Primary Efficacy Endpoint, the following criteria must be met:

- The participant must have experienced at least TWO of the following systemic symptoms: Fever (≥ 38ºC), chills, myalgia, headache, sore throat, new olfactory and taste disorder(s), OR
- The participant must have experienced at least ONE of the following respiratory signs/symptoms: cough, shortness of breath or difficulty breathing, OR clinical or radiographical evidence of pneumonia; AND
- The participant must have at least one NP swab, nasal swab, or saliva sample (or respiratory sample, if hospitalized) positive for SARS-CoV-2 by RT-PCR.

Secondary Efficacy Assessments:

To be considered a severe COVID-19, the following criteria must be met: a confirmed COVID-19 as per the Primary Efficacy Endpoint case definition, plus any of the following:

- Clinical signs indicative of severe systemic illness, Respiratory Rate $\geq$ 30 per minute, Heart Rate $\geq$ 125 beats per minute, $SpO_2 \leq 93\%$ on room air at sea level or $PaO_2/FIO_2 < 300$ mm Hg, OR
- Respiratory failure or Acute Respiratory Distress Syndrome (ARDS), (defined as needing high-flow oxygen, non-invasive or mechanical ventilation, or ECMO), evidence of shock (systolic blood pressure < 90 mmHg, diastolic BP < 60 mmHg or requiring vasopressors), OR
- Significant acute renal, hepatic or neurologic dysfunction, OR
- Admission to an intensive care unit or death.

The secondary case definition of COVID-19 is defined as the following systemic symptoms: fever (temperature $\geq$ 38ºC) or chills, cough, shortness of breath or difficulty breathing, fatigue, muscle aches or body aches, headache, new loss of taste or smell, sore throat, nasal congestion or rhinorrhea, nausea or vomiting or diarrhea AND a positive NP swab, nasal swab, or saliva sample (or respiratory sample, if hospitalized) for SARS-CoV-2 by RT-PCR.

Death attributed to COVID-19 is defined as any participant who dies during the study with a cause directly attributed to a complication of COVID-19.

Asymptomatic SARS-CoV-2 infection is determined by seroconversion due to infection assessed by binding antibody (bAb) levels against SARS-CoV-2 as measured by a ligand-binding assay specific to the SARS-CoV-2 nucleocapsid protein and a negative NP swab sample for SARS-CoV-2 at Day 1.

Immunogenicity Assessments:

Immunogenicity assessments will include the following:

- Serum bAb levels against SARS-CoV-2 as measured by ligand-binding assay specific to the SARS-CoV-2 S protein.
- Serum bAb levels against SARS-CoV-2 as measured by ligand-binding assay specific to the SARS-CoV-2 nucleocapsid protein.
- Serum nAb titer against SARS-CoV-2 as measured by pseudovirus and/or live virus neutralization assays.

**Safety Assessments:**	Safety assessments will include monitoring and recording of the following for each participant:<br>• Solicited local and systemic ARs that occur during the 7 days following each injection (ie, the day of injection and 6 subsequent days). Solicited ARs will be recorded daily using eDiaries. (Part A, Blinded Phase Only)<br>• Unsolicited AEs observed or reported during the 28 days following each injection (ie, the day of injection and 27 subsequent days). (Part A, Blinded Phase Only)<br>• AEs leading to discontinuation from dosing and/or study participation from Day 1 through Day 759 or withdrawal from the study.<br>• MAAEs from Day 1 through Day 759 or withdrawal from the study.<br>• SAEs from Day 1 through Day 759 or withdrawal from the study.<br>• Abnormal vital sign measurements.<br>• Physical examination findings.<br>• Pregnancy and accompanying outcomes.<br>• Concomitant medications and non-study vaccinations.
**Investigational Product, Dosage, and Route of Administration:**	The mRNA-1273 IP is an LNP dispersion of an mRNA encoding the prefusion stabilized S protein of SARS-CoV-2 formulated in LNPs composed of 4 lipids (1 proprietary and 3 commercially available): the proprietary ionizable lipid SM-102; cholesterol; 1,2-distearoyl-sn-glycero-3 phosphocholine (DSPC); and 1 monomethoxypolyethyleneglycol-2,3-dimyristylglycerol with polyethylene glycol of average molecular weight 2000 (PEG2000-DMG). The mRNA-1273 vaccine is provided as a sterile liquid for injection and is a white to off- white dispersion in appearance, at a concentration of 0.2 mg/mL in 20 mM Tris buffer containing 87 mg/mL sucrose and 10.7 mM sodium acetate at pH 7.5.<br><br>The placebo is 0.9% sodium chloride (normal saline) injection, which meets the criteria of the United States Pharmacopeia (USP).<br><br>Investigational product will be administered as an IM injection into the deltoid muscle on a 2-dose injection schedule on Day 1 and Day 29.<br><br>Each injection will have a volume of 0.5 mL and contain mRNA-1273 100 µg, or saline placebo. Preferably, vaccine should be

administered into the nondominant arm. The second dose of IP should be administered in the same arm as the first dose.

Unblinded personnel, who will not participate in any other aspect of the study during Part A, the Blinded Phase, will perform IP accountability, dose preparation, and IP administration. Study site personnel who were blinded during the Blinded Phase, will be unblinded at the participant level at the Participant Decision clinic visit.

Sample Size:

The sample size is driven by the total number of cases to demonstrate VE (mRNA-1273 vs. placebo) to prevent COVID-19. Under the assumption of proportional hazards over time and with 1:1 randomization of mRNA-1273 and placebo, a total of 151 COVID-19 cases will provide 90% power to detect a 60% reduction in hazard rate (60% VE), rejecting the null hypothesis H0: VE ≤ 30%, with 2 IAs at 35% and 70% of the target total number of cases using a 1-sided O'Brien-Fleming boundary for efficacy and a log-rank test statistic with a 1-sided false positive error rate of 0.025. The total number of cases pertains to the Per-protocol (PP) Set accruing at least 14 days after the second dose. There are 2 planned IAs in this study, which will be performed when approximately 35% and 70% of the target total number of cases have been observed. Approximately 30,000 participants will be randomized with the following assumptions:

- The target VE against COVID-19 is 60% (with 95% confidence interval (CI) lower bound ruling out 30%, rejecting the null hypothesis H0: VE ≤ 30%).
- A 6-month COVID-19 incidence rate of 0.75% in the placebo arm.
- An annual dropout rate of 2% (loss of evaluable participants).
- Two IAs at 35% and 70% of total target cases across the 2 treatment groups with O'Brien-Fleming boundaries for efficacy monitoring.
- 3-month uniform accrual.
- Approximately 15% of participants will be excluded from the PP population, and participants are at risk for COVID-19 starting 14 days after the second dose.

Power for Selected Secondary Efficacy Endpoints:

For the secondary objective on VE against virologically confirmed SARS-CoV-2 infection or COVID-19 regardless of symptomology or

severity (COV-INF), the study will have ≥ 90% power to demonstrate the VE is above 30% (to reject null hypothesis VE ≤ 30%) at 1-sided alpha of 2.5% if the true VE to prevent COV-INF is 60%, because every COVID-19 endpoint is necessarily a COV-INF endpoint.

For the secondary objective on VE against severe COVID-19, the power of demonstrating VE based on a total of 20 and 30 events under different scenarios of true VE and VE criteria has been calculated.

Statistical Methods:

Statistical Hypotheses: For the primary efficacy objective, the null hypothesis of this study is that the VE of mRNA-1273 to prevent first occurrence of COVID-19 is ≤ 30% (ie, $H_0^{efficacy}$: VE ≤ 0.3).

The study will be considered to meet the primary efficacy objective if the corresponding CI of VE rules out 30% at either one of the IA or at the primary analysis. In the primary analysis of VE of COVID-19, cases will be counted starting 14 days after the second dose of IP.

Vaccine efficacy is defined as the percent reduction in the hazard of the primary endpoint (mRNA-1273 vs placebo). Equivalently, the null hypothesis is:

- $H_0^{efficacy}$: hazard ratio (HR) ≥ 0.7 (equivalently, proportional hazards VE ≤ 0.3).

A stratified Cox proportional hazard model will be used to assess the magnitude of the treatment group difference (ie, HR) between mRNA-1273 and placebo at a 1-sided 0.025 significance level.

Analysis Populations: Analysis populations for statistical analyses are Randomization Set, Full Analysis Set (FAS), Modified Intent-to-Treat (mITT) Set, PP Set, Immunogenicity Subset, Solicited Safety Set, and Safety Set, as shown below:

- Randomization Set: All participants who are randomized, regardless of the participants' treatment status in the study.
- FAS: All randomized participants who received at least one dose of IP. Participants will be analyzed according to the group to which they were randomized.

- mITT Set: All participants in the FAS who had no immunologic or virologic evidence of prior COVID-19 (ie, negative NP swab test at Day 1 and/or bAb against SARS-CoV-2 nucleocapsid below limit of detection or lower limit of quantification [LLOQ]) at Day 1 before the first dose of IP. Participants will be analyzed according to the group to which they were randomized.
- Immunogenicity Subset: All participants in the FAS who were sampled into subset for characterizing mRNA-1273 immunogenicity and had a valid immunogenicity test result prior to the first dose of IP and at least 1 valid result after the first dose of IP. Participants in the subset who had major protocol deviations that impact critical or key immunogenicity or study data may be excluded from the Immunogenicity Subset. The details of the Immunogenicity Subset will be documented prior to analysis of immunogenicity data.
- Solicited Safety Set: The Solicited Safety Set consists of all randomized participants who received at least one dose of IP and contributed any solicited AR data. The Solicited Safety Set will be used for the analyses of solicited ARs and participants will be included in the treatment group corresponding to the IP that they actually received.
- Safety SetAll randomized participants who received at least one dose of IP. The Safety Set will be used for all analyses of safety except for the solicited ARs. Participants will be included in the treatment group corresponding to the IP that they actually received.

Efficacy Analyses: Efficacy analyses will be performed using the FAS, mITT and PP populations, and participants will be included in the treatment group to which they are randomized. The primary analysis population for efficacy will be the PP Set.

The table below summarizes the analysis approach for primary and secondary efficacy endpoints. Sensitivity analysis methods are described for each endpoint as applicable.

Endpoint	Statistical Analysis Methods
**Primary endpoint:**<br>VE of mRNA-1273 to prevent COVID-19	• Primary analysis: VE will be estimated with 1 - HR (mRNA-1273 vs placebo) using a Cox proportional hazard regression model with treatment group as a fixed effect and adjust for

	stratification factor based on the PP Set, with cases counted starting 14 days after the second dose of IP.<br>• Analysis using the same model based on the mITT Set.<br>• Sensitivity analysis using the same model based on the PP Set, with cases counted starting either immediately after the second dose of IP or immediately after the first dose of IP.<br>• Subgroup analysis of the primary efficacy endpoint will be performed to assess consistency of VE, such as in the age groups ≥ 18 and < 65 years and ≥ 65 years.<br>• Supportive analysis of VE to be estimated with 1 - ratio of incidence rates with 95% CI using the exact method conditional upon the total number of cases.<br>• Supportive analysis of cumulative incidence VE.

**Secondary endpoints:**	
• Vaccine efficacy of mRNA-1273 to prevent severe COVID-19<br>• Vaccine efficacy of mRNA-1273 to prevent serologically confirmed SARS-CoV-2 infection or COVID-19 regardless of symptomatology or severity.<br>• Vaccine efficacy of mRNA-1273 to prevent COVID-19 using a secondary definition of symptoms<br>• Vaccine efficacy of mRNA-1273 to prevent death caused by COVID-19<br>• Vaccine efficacy of mRNA-1273 to prevent COVID-19 after the first dose of IP<br>• Vaccine efficacy of mRNA-1273 to prevent asymptomatic SARS-CoV-2 infection	Similar analysis method as for the primary endpoint analysis.<br>For each of the secondary endpoints:<br>• Primary analysis: VE will be estimated with 1 - HR (mRNA-1273 vs placebo) using a Cox proportional hazard regression model with treatment group as a fixed effect and adjusting for stratification factor based on the PP Set, with cases counted starting 14 days after the second dose of IP.<br>• Analysis using the same model based on the mITT Set.<br>• Sensitivity analyses with cases counted starting immediately after the second dose of IP, 14 days after the first dose of IP, immediately after the first dose of IP, and immediately after randomization.<br>• Vaccine efficacy and 95% CI based on the case incidence will be estimated with 1 - ratio of incidence rates using the exact method conditional upon the total number of cases.

Vaccine efficacy of mRNA-1273 to prevent COVID-19 in all study participants, regardless of evidence of prior SARS-CoV-2 infection	The FAS population will be used for this secondary objective, using similar analysis methods as for the primary endpoint analysis.<br>• Primary analysis: VE will be estimated with 1 - HR (mRNA-1273 vs placebo) using a Cox proportional hazard regression model with treatment group as a fixed effect and adjusting for stratification factor based on the FAS, with cases counted starting 14 days after the second dose of IP.<br>• Sensitivity analyses with cases counted starting immediately after the second dose of IP, 14 days after the first dose of IP, immediately after the first dose of IP, and immediately after randomization.

**Long-term endpoints:**<br>Cases after the second injection of IP for participants in the mRNA-1273 Cohort and the Placebo Cohort, or after the second injection of mRNA-1273 for participants in the Placebo-mRNA-1273 Cohort<br>• COVID-19<br>• Severe COVID-19<br>• Either COVID-19 or SARS-CoV-2 infection<br>• Secondary definition of COVID-19<br>• Death caused by COVID-19<br>• COVID-19 regardless of evidence of prior SARS-CoV-2 infection determined by serologic titer against SARS-CoV-2 nucleocapsid<br>Cases after the first injection of IP for participants in the mRNA-1273 Cohort and the Placebo Cohort, or after the first injection of mRNA-1273 for participants in the Placebo-mRNA-1273 Cohort<br>• COVID-19	• Long-term efficacy will be evaluated after the primary analysis by including data collected in Open-label Phase.<br>• Long-term efficacy data will be summarized descriptively by treatment cohort without cohort comparison.<br>• In the primary approach, cases will be counted starting 14 days after the second dose of IP for participants in treatment cohorts of mRNA-1273 and Placebo or starting 14 days after the second dose of mRNA-1273 for participants in the Placebo-mRNA-1273 Cohort.<br>• Sensitivity analyses with cases starting from the second dose, 14 days after the first dose, or first dose of IP for participants in the cohorts of mRNA-1273 and placebo, or mRNA-1273 for participants in the Placebo-mRNA-1273 Cohort will be provided.<br>• Incidences of cases assessed by numbers, rates, and 2-sided 95% CI based on the exact method adjusting for person-time will be summarized by treatment cohort.<br>• The Kaplan-Meier analysis will be used to estimate cumulative incidences of time to first cases by treatment cohort.<br>• Long-term efficacy analysis will be performed using FAS for the case of COVID-19 regardless of prior SARS-

	CoV-2 infection, and long-term efficacy analysis of other cases will be conducted using the PP Set and mITT Set.<br>• Efficacy analyses based on data collected in the Open-Label Observational Phase only will be provided in the SAP.

Safety: Safety and reactogenicity will be assessed by clinical review of all relevant parameters including solicited ARs (local and systemic events), unsolicited AEs, SAEs, MAAEs, AEs leading to discontinuation, abnormal vital signs, and physical examination findings.

All safety analyses will be based on the Safety Set, except summaries of solicited ARs, which will be based on the Solicited Safety Set. All safety analyses will be provided by treatment group, and by treatment cohort as applicable, unless otherwise specified.

The number and percentage of participants with any solicited local AR, with any solicited systemic AR, and with any solicited AR during the 7-day follow-up period after each injection will be provided (Part A, the Blinded Phase only). A 2-sided 95% exact CI using the Clopper-Pearson method will be also provided for the percentage of participants with any solicited AR for each treatment group.

The number and percentage of participants with solicited ARs, unsolicited AEs, SAEs, MAAEs, Grade 3 or higher ARs and AEs, and AEs leading to discontinuation from study vaccine or participation in the study will be summarized. Unsolicited AE will be presented by MedDRA preferred term and system organ class.

For all other safety parameters, descriptive summary statistics will be provided.

Further details will be described in the statistical analysis plan (SAP).

Immunogenicity: The secondary immunogenicity endpoints will be analyzed using the Immunogenicity Subset by treatment group, by treatment cohort as applicable, and by baseline SARS-CoV-2 serostatus, unless otherwise specified.

The SAP will describe the complete set of immunogenicity analyses, including the approach to sample individuals into an Immunogenicity Subset for characterizing vaccine immunogenicity and assessing immunological correlates of risk and protection.

Data from quantitative immunogenicity assays will be summarized for each treatment group using positive response rates and geometric means with 95% CIs, for each timepoint for which an assessment is performed. Data from qualitative (ie, yielding a positive or negative result) assays will be summarized by tabulating the frequency of positive responses for each assay by group at each timepoint that an assessment is performed. Analyses will focus on the 2 key immunogenicity time points and the change in marker response between them: Day 1 before the first dose of IP and Day 57 (28 days after the second dose of IP). The SAP will describe the complete set of immunogenicity analyses.

Quantitative levels or geometric mean titer (GMT) of specific bAb with corresponding 95% CI at each timepoint and geometric mean fold rise (GMFR) of specific bAb with corresponding 95% CI at each post-baseline timepoint over pre-dose baseline at Day 1 will be provided by study arm. Descriptive summary statistics including median, minimum, and maximum will also be provided.

GMT of specific nAb with corresponding 95% CI at each timepoint and GMFR of specific nAb with corresponding 95% CI at each post-baseline timepoint over pre-dose baseline at Day 1 will be provided by study arm. Descriptive summary statistics including median, minimum, and maximum will also be provided. For summarizations of group variables values, antibody values reported as below the LLOQ will be replaced by 0.5 × LLOQ. Values that are greater than the upper limit of quantification (ULOQ) will be converted to the ULOQ.

The number and percentage of participants with a fold rise ≥ 2, ≥ 3, and ≥ 4 of serum SARS-CoV-2-specific nAb titers and participants with seroconversion due to vaccination from baseline will be provided with 2-sided 95% CI using the Clopper-Pearson method at each post-baseline timepoint. Seroconversion due to vaccination at a participant level may be defined as a change from below the LLOQ to equal or above LLOQ, or at least a 4-fold rise in terms of neutralizing antibody or vaccine antigen-specific bAb in participants with pre-existing bAb or nAb. The definition of seroconversion will be finalized and documented in the SAP prior to analyzing the immunogenicity data.

The GMT of specific nAb for each group and the geometric mean ratio (GMR) of mRNA-1273 versus placebo with corresponding 2-sided 95% CI will be estimated at each study timepoint using an analysis of covariance (ANCOVA) model with the treatment group and baseline values, if applicable, as explanatory variables, the analysis may adjust for the stratification factor.

1.2. Schema

Figure 1: Study Flow Diagram: Part A, the Blinded Phase followed by Part B, the Open-Label Observational Phase

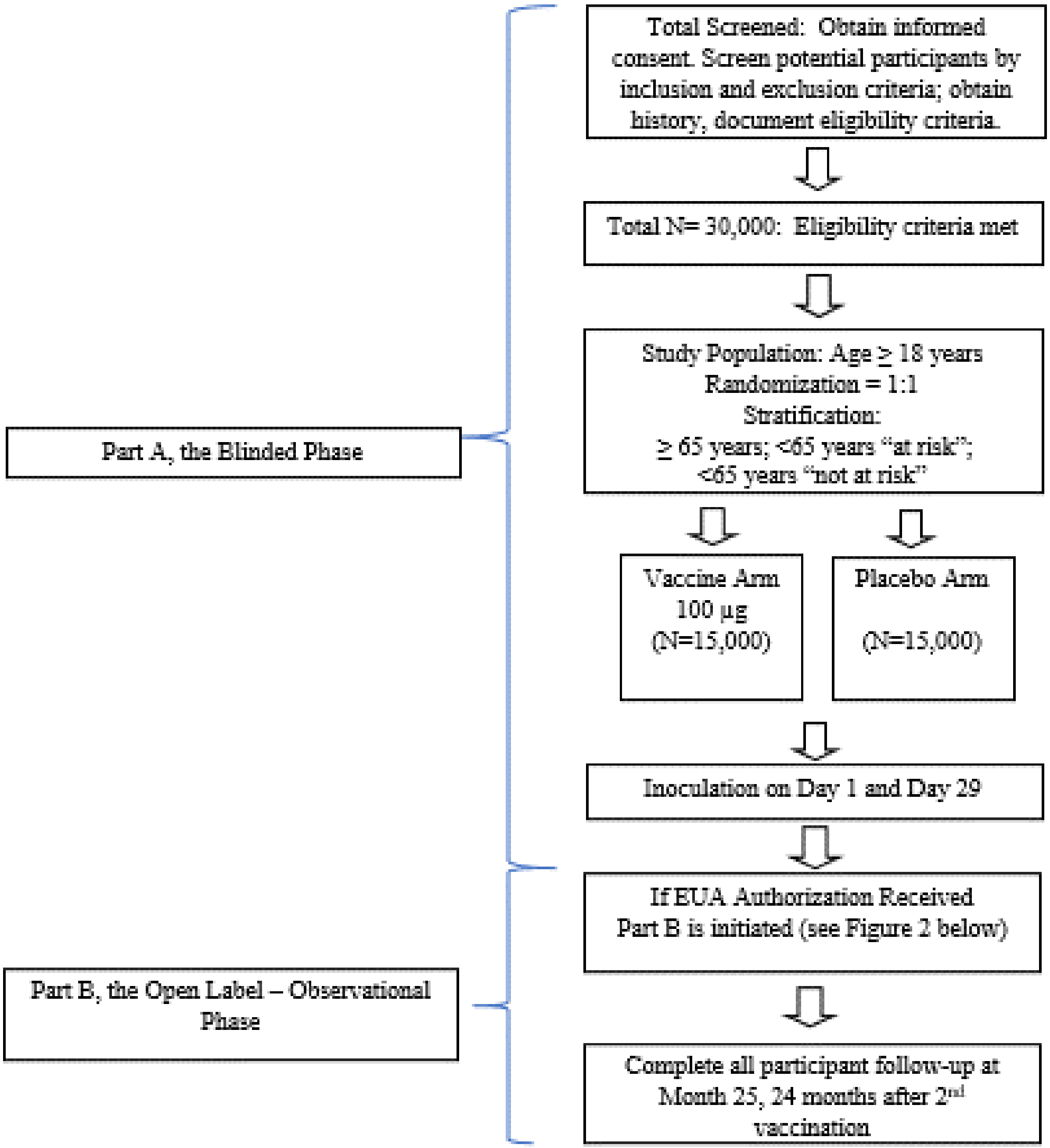

Figure 2: Part B, the Open-Label Observational Phase

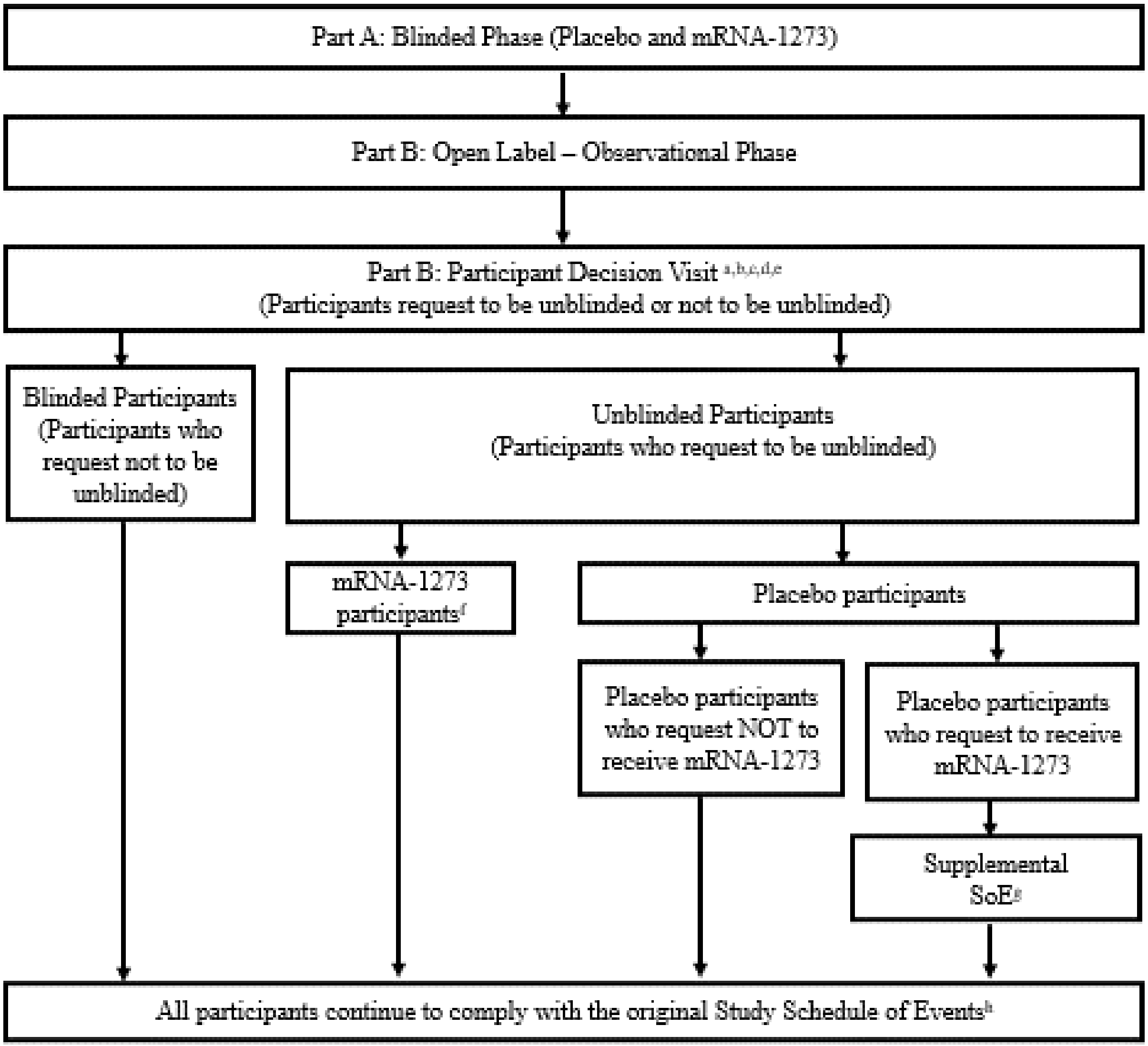

[a] All participants are encouraged to remain in the study.
[b] All participants are given the option to be unblinded to treatment received in Part A: Blinded Phase.
[c] All participants are counselled about the importance of continuing other public health measures to limit the spread of disease including physical-social distancing, wearing a mask, and hand-washing.
[d] All participants sign a revised ICF.
[e] All participants consent to provide a nasopharyngeal swab and a blood sample for immunologic analysis.
[f] mRNA-1273 recipients who only received one dose of the mRNA-1273 vaccine will receive the second dose of mRNA-1273 vaccine. Additional details provided in the Supplemental Schedule of Events (Table 22).
[g] Placebo recipients who request to receive mRNA-1273 and meet eligibility, will comply with a Supplemental Schedule of Events (Table 21) in addition to the Original Study Schedule of Events.
[h] Original Study Schedule of Events (Table 16, Table 17, Table 18, and Table 19).

TABLE OF CONTENTS

Clinical Study Protocol 1
Declaration of Investigator 3
Protocol Amendment Summary of Changes 4
IRB and Regulatory Authority Approval 6
1. Protocol Summary 7
1.1. Synopsis 7
1.2. Schema 27
Table of Contents 29
List of Tables 33
List of Figures 34
List of Abbreviations 35
2. Introduction 38
2.1. Study Rationale 38
2.2. Background and Overview 38
2.2.1. Nonclinical Studies 39
2.2.2. Clinical Studies 39
2.3. Benefit/Risk Assessment 41
2.3.1. Potential Benefits of Study Participation 41
2.3.2. Risks from Study Participation 41
2.3.3. Overall Benefit/Risk Conclusion 43
3. Objectives and Endpoints 44
4. Study Design 48
4.1. General Design 48
4.1.1. Part A, the Blinded Phase 48
4.1.2. Part B, the Open-label Observational Phase 50
4.2. Scientific Rationale for Study Design 54
4.3. Choice of Dose and Control Product 55
4.4. End of Study Definition 55
5. Study Population 56
5.1. Inclusion and Exclusion Criteria (Part A: Blinded Phase and Part B: Open-Label Phase) 56
5.1.1. Inclusion Criteria 56
5.1.2. Exclusion Criteria 57

5.2. Participant Restrictions 58
5.3. Screen Failures (Part A: Blinded Phase Only) 58
6. Study Treatment 60
6.1. Investigational Product 60
6.2. Dosing and Management of Investigational Product 60
6.2.1. Method of Randomly Assigning Participants to Treatment Groups (Part A, Blinded Phase Only) 60
6.2.2. Administration of Investigational Product 61
6.2.3. Delivery and Receipt 62
6.2.4. Packaging and Labeling 62
6.2.5. Storage 63
6.2.6. Investigational Product Accountability 63
6.2.7. Handling and Disposal 63
6.2.8. Unblinding 64
6.3. Study Treatment Compliance 66
6.4. Prior and Concomitant Therapy 67
6.4.1. Prior Medications and Therapies 67
6.4.2. Concomitant Medications and Therapies 67
6.4.3. Concomitant Medications and Vaccines that May Lead to the Elimination of a Participant from Per-protocol Analyses 68
7. Delaying or Discontinuing Study Treatment and Participant Withdrawal from the Study 69
7.1. Criteria for Delay of Study Treatment 69
7.2. Discontinuation of Study Treatment 69
7.3. Participant Withdrawal from the Study 71
7.3.1. Participant Withdrawal 71
7.4. Lost to Follow-up 72
8. Study Assessments and Procedures 73
8.1. Efficacy and Immunogenicity Assessments and Procedures 74
8.1.1. Efficacy Assessments Related to COVID-19 and SARS-CoV-2 Infection 74
8.1.2. Surveillance for COVID-19 Symptoms 75
8.1.3. Convalescent Period Starting with the Illness Visit 78
8.1.4. Ancillary Supplies for Participant Use 80
8.1.5. Immunogenicity Assessments 80

8.2. Safety Assessments 81
8.2.1. Safety Phone Calls 82
8.2.2. Use of Electronic Diaries 82
8.2.3. Demographics/Medical History 84
8.2.4. Physical Examination 85
8.2.5. Vital Sign Measurements 85
8.2.6. Blood Sampling Volumes 86
8.3. Safety Definitions and Procedures 86
8.3.1. Adverse Event 86
8.3.2. Medically Attended Adverse Events 87
8.3.3. Serious Adverse Events 88
8.3.4. Solicited Adverse Reactions (Part A, Blinded Phase Only) 89
8.3.5. Recording and Follow-up of Pregnancy 92
8.3.6. Recording and Follow-up of an AE and/or SAE 93
8.3.7. Time Period and Frequency for Collecting AE and SAE Information 93
8.3.8. Assessment of Intensity 94
8.3.9. Assessment of Causality 94
8.3.10. Reporting Adverse Events 95
8.3.11. Regulatory Reporting Requirements for SAEs 96
8.4. Monitoring Committees 96
8.4.1. Protocol Safety Review Team 96
8.4.2. Data and Safety Monitoring Board 96
8.4.3. Adjudication Committee 97
8.5. Management of Overdose 98
8.6. Pharmacokinetics 98
8.7. Pharmacodynamics 98
8.8. Exploratory Assessments and Biomarkers 98
8.9. Medical Resource Utilization and Health Economics 98
9. Statistical Considerations 99
9.1. Blinding and Responsibility for Analyses 99
9.2. Statistical Hypotheses 100
9.3. Sample Size Determination 100
9.3.1. Power for Selected Secondary Efficacy Endpoints 102

9.4. Analysis Populations 103
9.5. Statistical Analyses 104
9.5.1. Efficacy Analyses 104
9.5.2. Safety Analyses 110
9.5.3. Immunogenicity Analyses 111
9.5.4. Exploratory Analyses 112
9.5.5. Subgroup Analyses 113
9.6. Interim Analyses 113
10. References 115
11. Supporting Documentation and Operational Considerations 118
11.1. APPENDIX 1: Schedules of Events 119
11.2. APPENDIX 2: Study Governance Considerations 137
11.2.1. Regulatory and Ethical Considerations 137
11.2.2. Study Monitoring 137
11.2.3. Audits and Inspections 139
11.2.4. Financial Disclosure 139
11.2.5. Recruitment Procedures 139
11.2.6. Informed Consent Process 139
11.2.7. Protocol Amendments 141
11.2.8. Protocol Deviations 141
11.2.9. Data Protection 141
11.2.10. Sample Retention and Future Biomedical Research 142
11.2.11. Data Quality Assurance and Quality Control 143
11.2.12. Data Collection and Management 144
11.2.13. Source Documents 144
11.2.14. Retention of Records 145
11.2.15. Study and Site Closure 145
11.2.16. Publication Policy 146
11.3. APPENDIX 3: Contraceptive Guidance 147
11.4. APPENDIX 4: Statistical Appendices 148
11.4.1. Estimands and Estimand Specifications 148
11.4.2. Statistical Methods and Sensitivity Analyses 150
11.5. APPENDIX 5: Protocol Amendment History 151

LIST OF TABLES

Table 1: Objectives and Endpoints 44
Table 2: Summary of Treatment Groups 60
Table 3: Grading of COVID-19 Symptoms 79
Table 4: Maximum Planned Blood Sampling Volumes per Participant by Visit 86
Table 5: Solicited Adverse Reactions and Grades 90
Table 6: Conditions and Sample Size to Demonstrate Vaccine Efficacy 101
Table 7: Power of Demonstrating Vaccine Efficacy Against Severe COVID-19 102
Table 8: Populations for Analyses 103
Table 9: Statistical Analysis Methods of Efficacy Endpoints....104
Table 10: Treatment Cohorts for the Long-term Efficacy Analyses....108
Table 11: Long-term Efficacy Endpoints....108
Table 12: Analysis Strategy for Safety Parameters....110
Table 13: Immunogenicity Endpoints and Statistical Methods 112
Table 14: Burden of Disease Score....113
Table 15: Interim Boundaries Using O'Brien-Fleming Spending Function, Calculation Based on the PP Set for the Primary Efficacy Endpoint....114
Table 16: Schedule of Events (Vaccination Phase, Day 1 – Day 57) 120
Table 17: Schedule of Events (Surveillance Phase, Day 64 – Day 394) 123
Table 18: Schedule of Events (Surveillance Phase, Day 401 – Day 759) 126
Table 19: Schedule of Events (Convalescent Period, Starting with the Illness Visit) 128
Table 20: Part B: Participant Decision Clinic Visit 130
Table 21: Supplemental Schedule of Events: Open-Label Observational Phase - Placebo Participants who request to receive mRNA-1273....131
Table 22: Modified Supplemental Schedule of Events: Open-Label Observational Phase – mRNA-1273 Participants who ONLY received 1 dose of mRNA-1273....134
Table 23: Intercurrent Event Types....148
Table 24: Primary Objective and Estimands with Rationale for Strategies to Address Intercurrent Events for Per-Protocol Analysis....149
Table 25: Summary of Statistical Methods and Sensitivity Analyses 150

LIST OF FIGURES

Figure 1: Study Flow Diagram: Part A, the Blinded Phase followed by Part B, the Open-Label Observational Phase....................27
Figure 2: Part B, the Open-Label Observational Phase....................28
Figure 3: Event/Case-Driven Study with 24 Months of Planned Follow-Up....................53
Figure 4: Surveillance for COVID-19 Symptoms and the Corresponding Clinical Data Pathways....................77
Figure 5: Boundary Crossing Probabilities by Effect Size....................101

LIST OF ABBREVIATIONS

The following abbreviations and terms are used in this study protocol.

Abbreviation	Definition
AC	Adjudication Committee
AE	adverse event
AR	adverse reaction
ARDS	acute respiratory distress syndrome
bAb	binding antibody
BARDA	Biomedical Advance Research and Development Authority
BOD	burden of disease
CDC	US Centers for Disease Control and Prevention
CFR	Code of Federal Regulations
CI	confidence interval
CLIA	Clinical Laboratory Improvement Amendments
CMV	cytomegalovirus
CONSORT	Consolidated Standards of Reporting Trials
CoV	coronavirus
COV-INF	virologically confirmed SARS-CoV-2 infection regardless of symptomology or severity
CRO	contract research organization
CSR	clinical study report
DHHS	Department of Health and Human Services
DMID	Division of Microbiology and Infectious Diseases
DSMB	Data and Safety Monitoring Board
DSPC	1,2-distearoyl-sn-glycero-3-phosphocholine
eCRF	electronic case report form
eDiary	electronic diary
EUA	Emergency Use Authorization
ART	antiretroviral therapy
FAS	full analysis set
FDA	Food and Drug Administration
FSH	follicle-stimulating hormone
GCP	Good Clinical Practice
GMFR	geometric mean fold rise
GMP	Good Manufacturing Practice
GMT	geometric mean titer

Abbreviation	Definition
HCP	healthcare practitioner
HIV	Human Immunodeficiency Virus
hMPV	human metapneumovirus
HR	hazard ratio
HRT	hormonal replacement therapy
IA	interim analysis
IB	investigator's brochure
ICF	informed consent form
ICH	International Council for Harmonisation
IM	intramuscular
IP	investigational product
IRB	institutional review board
IRT	interactive response technology
LB	lower boundary
LLOQ	lower limit of quantification
LNP	lipid nanoparticle
LOD	limit of detection
LTFU	lost to follow-up
MAAE	medically attended adverse event
MedDRA	Medical Dictionary for Regulatory Activities
MERS-CoV	Middle East Respiratory Syndrome coronavirus
mRNA	messenger RNA
nAb	neutralizing antibody
NIAID	National Institute of Allergy and Infectious Diseases
NP	nasopharyngeal
PCR	polymerase chain reaction
PEG2000-DMG	1-monomethoxypolyethyleneglycol-2,3-dimyristylglycerol with polyethylene glycol of average molecular weight 2000
PIV3	parainfluenza virus type 3
PP	per-protocol
RT-PCR	reverse transcriptase polymerase chain reaction
S	spike
S-2P	spike protein with 2 proline residues introduced for stability in a prefusion conformation
SAE	serious adverse event

Abbreviation	Definition
SAP	statistical analysis plan
SARS-CoV	Severe Acute Respiratory Syndrome coronavirus
SM-102	heptadecan-9-yl 8-((2-hydroxyethyl) (6-oxo-6-(undecyloxy) hexyl) amino) octanoate
SoE	Schedule of Events
TEAE	treatment-emergent adverse event
ULOQ	upper limit of quantification
USP	United States Pharmacopeia
VE	vaccine efficacy
WHO	World Health Organization

2. INTRODUCTION

2.1. Study Rationale

Coronaviruses (CoVs) are a large family of viruses that cause illness ranging from the common cold to more severe diseases, such as Middle East Respiratory Syndrome (MERS-CoV) and Severe Acute Respiratory Syndrome (SARS-CoV).

An outbreak of the CoV disease (COVID-19) caused by SARS-CoV-2 began in Wuhan, Hubei Province, China in December 2019 and has spread throughout China and to over 216 other countries and territories, including the United States (WHO 2020). On 11 March 2020, the World Health Organization (WHO) officially declared COVID-19 a pandemic. As of 28 May 2020, the WHO reported more than 5,593,631 confirmed cases and 353,334 deaths globally and the US Centers for Disease Control and Prevention (CDC) reported 1,698,523 confirmed and probable cases of COVID-19, with 100,446 deaths in the United States (CDC 2020a). The CDC have reported that the highest risk of disease burden is in older adults (≥ 65 years old) and people of any age who have serious underlying medical conditions, such as chronic lung disease or moderate to severe asthma; serious heart conditions; severe obesity; diabetes; chronic kidney disease requiring dialysis; liver disease; and those who are immunocompromised (CDC 2020b).

There is currently no vaccine against SARS-CoV-2. Global efforts to evaluate novel antivirals and therapeutic strategies to treat severe SARS-CoV-2 infections have intensified, but no proven therapeutic currently exists. Therefore, there is an urgent public health need for rapid development of novel interventions to prevent the spread of this disease. The primary goal of this Phase 3 study is to evaluate the vaccine efficacy (VE) of mRNA-1273 to prevent COVID-19, compared to placebo.

2.2. Background and Overview

ModernaTX, Inc. (the Sponsor) has developed a rapid-response, proprietary vaccine platform based on a messenger RNA (mRNA) delivery system. The platform is based on the principle and observations that cells in vivo can take up mRNA, translate it, and then express protein viral antigen(s) on the cell surface. The delivered mRNA does not enter the cellular nucleus or interact with the genome, is nonreplicating, and is expressed transiently. mRNA vaccines have been used to induce immune responses against infectious pathogens such as cytomegalovirus (CMV) (NCT03382405), human metapneumovirus (hMPV) and parainfluenza virus type 3 (PIV3) (NCT03392389), Zika virus (NCT03325075), and influenza virus (NCT03076385 and NCT03345043).

The Sponsor is using its mRNA-based platform to develop a novel lipid nanoparticle (LNP)-encapsulated mRNA-based vaccine against SARS-CoV-2 (mRNA-1273). mRNA-1273 encodes for the full-length spike (S) protein of SARS-CoV-2, modified to introduce 2 proline

residues to stabilize the S protein (S2P) in a prefusion conformation. The CoV S protein mediates attachment and entry of the virus into host cells (by fusion), making it a primary target for neutralizing antibodies that prevent infection (Johnson et al 2016; Wang et al 2015; Wang et al 2018; Chen et al 2017; Corti et al 2015; Yu et al 2015; Kim et al 2019; Widjaja et al 2019). It has been confirmed that the stabilized SARS-CoV-2 S2P antigen presents in the correct prefusion conformation (Wrapp et al 2020).

The development of the mRNA-1273 vaccine is being accelerated to address the current SARS-CoV-2 outbreak as a result of the uniquely rapid and scalable manufacturing process for mRNA-1273. The primary goal of this Phase 3 study is to evaluate the VE of mRNA-1273 to prevent COVID-19, compared to placebo.

2.2.1. Nonclinical Studies

Nonclinical studies have demonstrated that CoV S proteins are immunogenic and S protein-based vaccines, including those based on mRNA delivery platforms, are protective in animals. Prior clinical studies of vaccines targeting related CoVs and other viruses have demonstrated that mRNA-based vaccines are safe and immunogenic. It is therefore anticipated that mRNA-1273 will generate robust immune responses to the SARS-CoV-2 S protein and will be well tolerated. In addition, mRNA-1273 has shown preliminary evidence of protection against SARS-CoV-2 in a murine model of infection (data on file).

In support of development of mRNA-1273 for prophylaxis against SARS-CoV-2 infection, nonclinical immunogenicity, biodistribution, and safety studies have been completed with similar mRNA-based vaccines formulated in LNPs containing SM-102 (heptadecan-9-yl 8 ((2 hydroxyethyl)(6 oxo 6-(undecyloxy)hexyl)amino)octanoate), the novel proprietary lipid used in the mRNA-1273 LNP formulation.

A detailed review of nonclinical experience with mRNA-1273 vaccine is provided in the Investigator’s Brochure (IB).

2.2.2. Clinical Studies

The mRNA-1273 vaccine is currently being evaluated for safety and immunogenicity in a dose-ranging Phase 1 study (NCT04283461) sponsored and conducted by the Division of Microbiology and Infectious Diseases (DMID) of the National Institute of Allergy and Infectious Diseases (NIAID). The Phase 1 DMID study is an open-label dose-ranging study of mRNA-1273 in healthy adult male and non-pregnant female participants in 3 age groups: age 18 to 55 years, inclusive (45 participants); age 56 to 70 years, inclusive (30 participants); and ≥ 71 years (30 participants). Each participant will receive an IM injection (0.5 mL) of mRNA-1273 on Days 1 and 29 in the deltoid muscle and will be followed for 13 months after the second injection.

As of 26 October 2020, 120 participants were enrolled. All participants received the first injection of mRNA-1273, and 116 participants received the second injection. Three mRNA-1273 dose levels (25, 100, and 50 µg) administered 28 days apart have been assessed in participants 18 to 55 years, 56 to 70 years, and ≥ 71 years of age. The mRNA-1273 250-µg dose was not evaluated in participants 56 to 70 years and ≥ 71 years of age due to reactogenicity observed in 4 participants in the 250 µg (18 to 55 years) dose cohort. Across all age groups, solicited adverse reactions (ARs) were predominantly mild or moderate in severity and most commonly included fatigue, chills, headache, myalgia, and pain at the injection site. The incidence and severity of solicited ARs were generally dose dependent and increases in incidence and severity were generally observed after the second injection. The incidence of systemic reactions increased after the second injection, particularly at the highest (250 µg) dose. Four (27%) participants in the 250 µg dose cohort reported at least 1 severe solicited AR after the second injection, including feverishness, fatigue, fever, headache, myalgia, nausea, and erythema/redness. No serious adverse events (SAEs) have been reported through Day 119, and no pause rules were triggered during the study.

Additionally, a dose-finding Phase 2a study (mRNA-1273-P201) conducted by the Sponsor under IND 19745 will expand the safety and immunogenicity database by testing 2 two dose levels (50 µg and 100 µg) in 400 adults. As of 05 November 2020, 200 (100%) participants each in the mRNA-1273 50 µg group, mRNA-1273 100 µg group, and placebo group received the first dose, and 195 (97.5%) participants in the mRNA-1273 50 µg group, 198 participants (99.0%) in the mRNA-1273 100 µg group, and 194 (97.0%) participants in the placebo group received the second injection (mRNA-1273-P201). In this study, 2 mRNA-1273 dose levels (50 and 100 µg) or placebo administered 28 days apart were assessed in participants ≥ 18 to < 55 years and ≥ 55 years of age. No protocol-specified pause rules were met during the study. In addition, the study initiated with a sentinel group of 50 participants ≥ 55 years of age; after review of the safety data by the independent Safety Monitoring Committee, the cohort was expanded to include the remaining 250 participants ≥ 55 years of age.

In the Phase 3 study (mRNA-1273-P301), as of 25 October 2020, a total of 30,350 participants have been dosed in the Blinded Phase of the Study (15,184 received mRNA-1273 and 15,165 received placebo). The protocol-defined interim analysis (IA) was conducted in November 2020, which indicated that the primary efficacy endpoint in Study mRNA-1273-P301 was met: mRNA-1273 prevented COVID-19 starting 14 days after the second dose of the mRNA-1273 vaccine, based on a total of 95 adjudicated cases accrued (5 cases in the mRNA-1273 group and 90 cases in the placebo group). The VE was 94.5% (95% CI: 86.5%, 97.8%; 1-sided p-value < 0.0001), rejecting the null hypothesis of VE ≤ 30% and achieving the prespecified efficacy boundary based on the 1-sided nominal alpha of 0.0047 using the Lan-DeMets O'Brien-Fleming spending function. The mRNA-1273 vaccine was effective in preventing severe COVID-19, with 11 cases in the placebo group and 0 cases in the mRNA-1273 group. In addition, the vaccine was

efficacious in preventing COVID-19 regardless of prior SARS-CoV-2 infection for cases starting 14 days after the second injection (VE of 93.5% based on hazard ratio [HR]).

In this study, the safety and reactogenicity of mRNA-1273 100 µg compared with placebo administered 28 days apart were assessed in participants 18 years of age and older at increased risk for acquiring COVID-19 based on occupation or location and living circumstances. Reactogenicity (solicited local and/or systemic ARs) was observed in the majority of participants in the mRNA-1273 group and generally increased after the second injection. The rates of local and systemic ARs were higher in the mRNA-1273 group than in the placebo group after each injection. The majority of solicited ARs in the mRNA-1273 group were grade 1 to grade 2 in severity and generally resolved within 3 days or less. The incidence of unsolicited treatment-emergent adverse events (TEAEs), severe TEAEs, and medically attended adverse events (MAAEs) during the 28 days after injection was also generally similar in participants who received mRNA-1273 and those who received placebo.

Deaths and SAEs were generally reported at a similar incidence in the mRNA 1273 and placebo groups. There was no evidence of enhanced disease, as fewer cases of severe COVID-19 and COVID-19 were observed in participants who received mRNA-1273 than in those who received placebo.

2.3. Benefit/Risk Assessment

2.3.1. Potential Benefits of Study Participation

The target study population for this study is adults with no known history of SARS-CoV-2 infection but whose locations or circumstances put them at high risk of COVID-19. The following benefits may accrue to participants:

- The mRNA-1273 vaccine may be an effective vaccine against COVID-19.
- A baseline (Day 1) evaluation for SARS-CoV-2 infection and ongoing surveillance for COVID-19 throughout the study.
- Contributing to the development of a vaccine against COVID-19, a current pandemic disease.

2.3.2. Risks from Study Participation

Immediate systemic allergic reactions (eg, anaphylaxis) can occur following any vaccination. These reactions are very rare and are estimated to occur once per 450,000 vaccinations for vaccines that do not contain allergens such as gelatin or egg protein (Zent et al 2002). As a precaution, all participants will remain under observation at the study site for at least 30 minutes after vaccination.

Vasovagal syncope (fainting) can occur before or after any vaccination, is usually triggered by the pain or anxiety caused by the injection and is not related to the substance injected. Therefore, it is important that standard precautions and procedures be followed to avoid injury from fainting.

Intramuscular injection with other mRNA vaccines manufactured by the Sponsor containing the SM-102 lipid formulation commonly results in a transient and self-limiting local inflammatory reaction. This typically includes pain, erythema (redness), or swelling (hardness) at the injection site, which are mostly mild-to-moderate in severity and usually occur within 24 hours of vaccination. More severe, but self-limited, local reactions, erythema and induration, have been observed at dose of mRNA-1273 exceeding the dose proposed in this study.

Most systemic adverse events (AEs) observed after vaccination do not exceed mild-to-moderate severity. The most commonly reported systemic ARs are anticipated to be fever, fatigue, chills, headache, myalgias and arthralgias. More severe reactions, including erythema, induration, fever, headache and nausea, were reported after receiving doses of mRNA-1273 that were greater than the dose proposed for use in this study. In all cases, the reactions resolved spontaneously.

Laboratory abnormalities (including increases in liver functional tests and serum lipase levels) following vaccination were observed in clinical studies with similar mRNA-based vaccines. These abnormalities were without clinical symptoms or signs and returned toward baseline (Day 1) values over time. The clinical significance of these observations is unknown. Further details are provided in the current IB.

There is a theoretical risk that active vaccination to prevent the novel viral infection caused by SARS-CoV-2 may cause a paradoxical increase in the risk of disease. This possibility is based on the rare phenomenon of vaccine-associated disease enhancement which was first seen in the 1960s with 2 vaccines made in the same way (formalin-inactivated whole virus) and designed to protect children against infection with RSV (Chin et al 1969) or measles (Fulginiti et al 1967). Disease enhancement has also been proposed as a possible explanation for cases of more serious disease associated with dengue vaccination (Thomas and Yoon 2019; WHO 2018). It is not known if mRNA-1273 will increase the risk of enhanced disease.

To monitor the risk of enhanced disease in this study, an independent Data and Safety Monitoring Board (DSMB) will review unblinded cases of COVID-19 to assess for inefficacy and also for numerical imbalance in cases of both COVID-19 and severe COVID-19 with the purpose of providing a non-binding recommendation to the Sponsor (Section 8.4.2).

2.3.3. Overall Benefit/Risk Conclusion

All participants will be included based on their increased risk of SARS-CoV-2 infection. Accordingly, all will benefit from baseline and ongoing evaluations for SARS-CoV-2 infection.

Since this is a placebo-controlled study (Section 4), half the participants will have the potential to receive mRNA-1273 vaccine, the efficacy of which is unknown at present. Vaccination with mRNA-1273 may not prevent COVID-19 in all vaccines.

Participants who receive placebo as part of this study may have an opportunity cost of not being treated with another investigational vaccine against COVID-19.

The placebo for this study is a saline solution, without any LNP. Thus, participants receiving saline may be at lower risk of AEs related to injection than participants receiving mRNA-1273.

Safety findings will be monitored and periodically reviewed by the DSMB to evaluate the safety and treatment status of all participants. The DSMB will review and assess the safety data as described in Section 8.4.2.

Considering the lack of approved vaccines for COVID-19, the participants' risk of COVID-19 outside the study, and the nonclinical and clinical data to date, the Sponsor considers the potential benefits of participation to exceed the risks.

Based on the interim results from the pivotal Phase 3 study, mRNA-1273 prevents COVID-19 and severe COVID-19. The demonstrated clinical benefit of mRNA-1273 is supported by evidence of a robust immune response both in terms of binding antibodies (bAbs) and neutralizing antibodies (nAbs) as well as the induction of CD4+ T-cells with a Th-1 dominant phenotype. Based on administration of mRNA-1273 to approximately 15,693 adults across all 3 clinical studies to date, there have been no emergent safety concerns and the AE profile is manifested largely by mild to moderate reactogenicity lasting 2 to 3 days.

3. OBJECTIVES AND ENDPOINTS

Table 1: Objectives and Endpoints

Objectives and Endpoints	
**Primary Objective**	**Primary Endpoints**
**Efficacy Objective (Primary):**<br>To demonstrate the efficacy of mRNA-1273 to prevent COVID-19.	**Efficacy Endpoints (Primary):** Vaccine efficacy of mRNA-1273 to prevent the first occurrence of COVID-19 starting 14 days after the second dose of investigational product (IP), where COVID-19 is defined as symptomatic disease based on the following criteria:<br>• The participant must have experienced at least TWO of the following systemic symptoms: Fever (≥ 38ºC), chills, myalgia, headache, sore throat, new olfactory and taste disorder(s), OR<br>• The participant must have experienced at least ONE of the following respiratory signs/symptoms: cough, shortness of breath or difficulty breathing, OR clinical or radiographical evidence of pneumonia; AND<br>• The participant must have at least one NP swab, nasal swab, or saliva sample (or respiratory sample, if hospitalized) positive for SARS-CoV-2 by RT-PCR
**Safety Objective (Primary):**<br>To evaluate the safety and reactogenicity of 2 injections of the mRNA-1273 vaccine given 28 days apart.	**Safety Endpoint (Primary):**<br>• Solicited local and systemic ARs through 7 days after each dose of IP (Part A: Blinded Phase only).<br>• Unsolicited AEs through 28 days after each dose of IP (Part A: Blinded Phase only).<br>• MAAEs or AEs leading to withdrawal through the entire study period.<br>• SAEs throughout the entire study period.

**Objectives and Endpoints**	
**Efficacy Objectives (Secondary)**	**Efficacy Endpoints (Secondary)**
To evaluate the efficacy of mRNA-1273 to prevent severe COVID-19	Vaccine efficacy of mRNA-1273 to prevent severe COVID-19, defined as first occurrence of COVID-19 starting 14 days after the second dose of IP, (as per the primary endpoint) AND any of the following:<br>• Clinical signs indicative of severe systemic illness, Respiratory Rate $\geq$ 30 per minute, Heart Rate $\geq$ 125 beats per minute, $SpO_2 \leq 93\%$ on room air at sea level or $PaO_2/FIO_2 < 300$ mm Hg, OR<br>• Respiratory failure or Acute Respiratory Distress Syndrome (ARDS), (defined as needing high-flow oxygen, non-invasive or mechanical ventilation, or ECMO), evidence of shock (systolic blood pressure < 90 mmHg, diastolic BP < 60 mmHg or requiring vasopressors), OR<br>• Significant acute renal, hepatic or neurologic dysfunction, OR<br>• Admission to an intensive care unit or death
To evaluate the efficacy of mRNA-1273 to prevent serologically confirmed SARS-CoV-2 infection or COVID-19 regardless of symptomatology or severity.	Vaccine efficacy of mRNA-1273 to prevent the first occurrence of either COVID-19 or SARS-CoV-2 infection starting 14 days after the second IP dose.<br>This endpoint is a combination of COVID-19, defined as for the primary endpoint, and asymptomatic SARS-CoV-2 infection, determined by seroconversion assessed by bAb levels against SARS-CoV-2 as measured by a ligand-binding assay specific to the SARS-CoV-2 nucleocapsid protein and with a negative nasopharyngeal (NP) swab sample for SARS-CoV-2 at Day 1 (Section 8.1.1).

Objectives and Endpoints	
To evaluate VE against a secondary definition of COVID-19.	Vaccine efficacy of mRNA-1273 to prevent the secondary case definition of COVID-19 starting 14 days after the second IP dose.<br>The secondary case definition of COVID-19 is defined as the following systemic symptoms: fever (temperature ≥ 38ºC), or chills, cough, shortness of breath or difficulty breathing, fatigue, muscle aches or body aches, headache, new loss of taste or smell, sore throat, nasal congestion or rhinorrhea, nausea or vomiting, or diarrhea AND a positive NP swab, nasal swab, or saliva sample (or respiratory sample, if hospitalized) for SARS-CoV-2 by RT-PCR
To evaluate VE to prevent death caused by COVID-19.	Vaccine efficacy of mRNA-1273 to prevent death due to a cause directly attributed to a complication of COVID-19, starting 14 days after the second IP dose.
To evaluate the efficacy of mRNA-1273 to prevent COVID-19 after the first dose of IP.	Vaccine efficacy of mRNA-1273 to prevent the first occurrence of COVID-19 starting 14 days after the first dose of IP.
To evaluate the efficacy of mRNA-1273 to prevent COVID-19 in all study participants, regardless of evidence of prior SARS-CoV-2 infection.	Vaccine efficacy of mRNA-1273 to prevent the first occurrence of COVID-19 starting 14 days after the second dose of IP regardless of evidence of prior SARS-CoV-2 infection determined by serologic titer against SARS-CoV-2 nucleocapsid (FAS analysis population, see Section 9.4).
To evaluate the efficacy of mRNA-1273 to prevent asymptomatic SARS-CoV-2 infection.	Vaccine efficacy to prevent the first occurrence of SARS-CoV-2 infection in the absence of symptoms defining COVID-19 starting 14 days after the second IP dose.<br>SARS-CoV-2 infection determined by seroconversion assessed by bAb levels against SARS-CoV-2 as measured by a ligand-binding assay specific to the SARS-CoV-2 nucleocapsid protein and with a negative NP swab sample for SARS-CoV-2 at Day 1 (Section 8.1.1).

Objectives and Endpoints	
**Immunogenicity Objective (Secondary):**	**Immunogenicity Endpoints (Secondary):**
To evaluate the immunogenicity of 2 doses of mRNA-1273 given 28 days apart.	• Geometric mean titer (GMT) of SARS-CoV-2 -specific neutralizing antibody (nAb) on Day 1, Day 29, Day 57, Day 209, Day 394, and Day 759.<br>• Geometric mean fold rise (GMFR) of SARS-CoV-2-specific nAb relative to Day 1 on Day 29, Day 57, Day 209, Day 394, and Day 759.<br>• Quantified levels or GMT of S protein-specific binding antibody (bAb) on Day 1, Day 29, Day 57, Day 209, Day 394, and Day 759.<br>• GMFR of S protein -specific bAb relative to Day 1 on Day 29, Day 57, Day 209, Day 394, and Day 759.
**Exploratory Objectives**	
To evaluate the effect of mRNA-1273 on the viral infection kinetics as measured by viral load at SARS-CoV-2 infection diagnosis by RT-PCR and number of days from the estimated date of SARS-CoV-2 infection until undetectable SARS-CoV-2 infection by RT-PCR.	
To assess VE to reduce the duration of symptoms of COVID-19.	
To evaluate VE against all-cause mortality.	
To assess VE against burden of disease (BOD) due to COVID-19.	
To evaluate the genetic and/or phenotypic relationships of isolated SARS-CoV-2 strains to the vaccine sequence.	
To evaluate immune response markers after dosing with IP as correlates of risk of COVID-19 and as correlates of risk of SARS-CoV-2 infection.	
To conduct additional analyses related to furthering the understanding of SARS-CoV-2 infection and COVID-19, including analyses related to the immunology of this or other vaccines, detection of viral infection, and clinical conduct.	

Abbreviations: AE = adverse event; AR = adverse reaction; bAb = binding antibody; FAS = full analysis set; GMFR = geometric mean fold rise; GMT = geometric mean titer; ICU = intensive care unit; MAAE = medically attended adverse event; nAb = neutralizing antibody; NP = nasopharyngeal; SAE = serious adverse event; VE = vaccine efficacy.

4. STUDY DESIGN

4.1. General Design

This is a two-part Phase 3 study: Part A and Part B. Participants in Part A, the Blinded Phase of this study are blinded to their treatment assignment. Given that the primary efficacy endpoint for mRNA-1273 against COVID-19 was met per the protocol-defined IA, Part B, the Open-Label Observational Phase of this study is designed to offer eligible participants who received placebo in Part A of this study, an option to request open-label mRNA-1273 (as long as investigational vaccine is available) (Figure 1).

All participants may have up to 8 scheduled clinic visits, including Screening, Day 1, Day 29, Day 57, Day 209, Day 394, Day 759, and the Participant Decision Visit as specified in Schedule of Events (SoEs) (Table 16, Table 17, Table 18, Table 19, and Table 20). Participants eligible for Part B OL dosing may have up to 3 additional scheduled clinic visits as specified in SoEs (Table 21 and Table 22).

This study will be conducted in compliance with the protocol, Good Clinical Practice (GCP), and all applicable regulatory requirements.

4.1.1. Part A, the Blinded Phase

The Blinded Phase of this study is a randomized, stratified, observer-blind, placebo-controlled evaluation of the efficacy, safety, and immunogenicity of mRNA-1273 SARS-CoV-2 vaccine compared to placebo in adults 18 years of age and older who have no known history of SARS-CoV-2 infection but whose locations or circumstances put them at appreciable risk of acquiring COVID-19 and/or SARS-CoV-2 infection. Figure 1 shows the study flow and Appendix 1 (Table 16, Table 17, Table 18, and Table 19) displays the planned Schedules of Events.

Approximately 30,000 participants will be randomly assigned to receive doses of either 100 µg of mRNA-1273 vaccine or a placebo control in a 1:1 randomization ratio. Assignment will be stratified by age and health risk (Section 6.2.1.1). This is a case-driven study and thus final sample size of the study will depend on the actual attack rate of COVID-19.

All participants will be assessed for efficacy and safety endpoints and provide a NP swab sample and blood sample before the first and second dose of IP, in addition to a series of post-dose blood samples for immunogenicity through 24 months after the second dose of IP. Efficacy assessments will include surveillance for COVID-19 with RT-PCR confirmation of SARS-CoV-2 infection after the first and second dose of IP. As noted above, this is a case-driven study: if the prespecified criteria for early efficacy are met at the time of either IA or overall efficacy at the primary analysis, a final study report describing the efficacy and safety of mRNA-1273 will be prepared based on

the data available at that time. In the event that success criteria are met either at the time of the IA or when the total number of cases toward the primary endpoint have accrued, participants will continue to be followed in a blinded fashion until Month 25 to enable assessment of long-term safety and durability of VE (Figure 3). If the study concludes early, all participants will be requested to provide a final blood sample at the time of study conclusion.

Each participant will receive 2 doses of IP by 0.5 mL intramuscular (IM) injection, the first on Day 1 and the second on Day 29. An NP swab sample will be collected prior to the first and second dose of IP, for evaluation by RT-PCR. To preserve observer blinding, only delegated unblinded study personnel responsible for study vaccine preparation, administration and/or accountability will have knowledge of study treatment assignment (Section 6.2.8.1).

Participants will be given an electronic diary (eDiary) to report solicited ARs for 7 days after each dose of IP (Part A, Blinded Phase only) and to prompt an unscheduled clinic visit for clinical evaluation and NP swab sample if a participant experiences any symptoms of COVID-19. Participants will use the eDiary to report solicited ARs for 7 days after each dose of IP (Part A, Blinded Phase only) and weekly eDiary prompts (every 7 days) to elicit an unscheduled Illness Visit if the participant is experiencing COVID-19 symptoms. All participants will receive safety calls on Day 8, Day 15, Day 22, Day 36, and Day 43 that will serve both to monitor for unsolicited AEs and to monitor for symptoms of COVID-19.

Safety telephone calls and eDiary safety prompts will be performed in conjunction with surveillance for COVID-19 according to the SoEs (Table 16, Table 17, Table 18, and Table 19) and are intended to capture SAEs, MAAEs, AEs leading to withdrawal, concomitant medications associated with these events, receipt of non-study vaccinations, and pregnancy (Section 8.2.1). If an eDiary prompt results in identification of a relevant safety event, a follow-up safety call will be triggered.

Surveillance for COVID-19 will be performed through weekly contacts with the participant via a combination of telephone calls and completion of an eDiary starting at Day 1 through the end of the study (Section 8.1.2). Participants with symptoms of COVID-19 lasting at least 48 hours (except for fever and/or respiratory symptoms) will return to the clinic or will be visited at home by medically qualified site staff within 72 hours to collect an NP swab sample for RT-PCR testing for SARS-CoV-2 and other respiratory pathogens, or alternatively, if a clinic or home visit is not possible, will submit a saliva (or nasal swab) sample for SARS-CoV-2 RT-PCR testing (Section 8.1.1).

All study participants who experience COVID-19 symptoms and subsequently present for an Illness Visit (in-clinic or at home) will be given an instruction card listing symptoms and severity grading system along with a thermometer, an oxygen saturation monitor, and saliva collection tubes. The list of symptoms is presented in Section 8.1.2 and the severity scoring system is

presented in Section 8.1.3. Study participants will be contacted by the investigator (or appropriately delegated study staff) daily with telemedicine visits through Day 14 or until symptoms have resolved, whichever is later. During the telemedicine visit (preferably done in the evening), the participant will be asked to verbally report the severity of each symptom, their highest body temperature and lowest oxygen saturation for that day, and the investigator will determine if medical attention is required due to worsening of COVID-19 symptoms (Table 19). Study participants will collect their own saliva (or nasal swab) sample on 3, 5, 7, 9, 14, and 21 days after the initial Illness Visit meeting criteria for COVID-19 (defined as the date of onset of symptoms and positive virologic test). Finally, a convalescent visit will be scheduled approximately 28 days after the initial Illness Visit. At this visit, a saliva (or nasal swab) sample will be collected and a blood sample will be drawn for immunologic assessment of SARS-CoV-2 infection.

At each dosing visit, participants will be instructed (Day 1) or reminded (Day 29) on how to document and report solicited ARs in the eDiary provided. Solicited ARs will be assessed for 7 days after each IP dose (Part A, Blinded Phase only) and unsolicited AEs will be assessed for 28 days after each IP dose (Part A, Blinded Phase only); SAEs, MAAEs, AEs leading to withdrawal, will be assessed throughout the study.

Participants may experience AEs that necessitate an unscheduled visit. There may also be situations in which the investigator asks a participant to report for an unscheduled visit following the report of an AE. Additional examinations may be conducted at these visits as necessary to ensure the safety and well-being of participants during the study. Electronic case report forms (eCRFs) should be completed for each unscheduled visit.

4.1.2. Part B, the Open-label Observational Phase

The Part B, the Open-Label Observational Phase of the study is prompted by the authorization of a COVID-19 vaccine under EUA. Transitioning the study to Part B permits all ongoing study participants to be informed of the availability and eligibility criteria of any COVID-19 vaccine made available under an EUA and the option to offer all ongoing study participants who request unblinding, an opportunity to schedule a Participant Decision clinic visit to know their original treatment assignment (placebo vs. mRNA-1273 vaccine).

Part B, the Open-Label Observational Phase of the study, also provides the opportunity for eligible study participants who previously received placebo, to actively request to receive 2 doses of mRNA-1273 vaccine (as long as investigational vaccine is available). All study participants will receive a Notification Letter and will be asked to schedule a Participant Decision clinic visit. Principal Investigators should consider current local public health guidance for administration of COVID-19 vaccines under EUA when determining the scheduling priority of participants.

With regards to eligibility in Part B, the CDC-EUA guidance specifications will supersede eligibility criteria in the protocol.

All participants will proceed to Part B, the Open-Label Observational Phase of the study, starting with a Participant Decision clinic visit (Figure 2).

At the Participant Decision clinic visit, all participants will:

- Be encouraged to remain in the ongoing study,
- Be given the option to be unblinded as to their original group assignment (placebo vs. mRNA-1273 vaccine),
- Be counselled about the importance of continuing other public health measures to limit the spread of disease including social distancing, wearing a mask, and hand-washing,
- Sign a revised informed consent form (ICF),
- Provide a NP swab for RT-PCR for SARS-CoV-2 and a blood sample for serology prior to unblinding.

Figure 2 shows the Part B Study Flow schematic.

The following participants will continue with the original study SoEs presented in Table 16, Table 17, Table 18, and Table 19 (Figure 2):

- participants who request to not be unblinded,
- participants who request to be unblinded and received mRNA-1273, and
- participants who request to be unblinded and received placebo and choose to remain on placebo.

Participants who are unblinded, received placebo, are eligible, and request to receive mRNA-1273 (as long as investigational vaccine is available) (Figure 2), will have the following clinic visits as shown in the Supplemental SoE (Table 21):

- Open-label Dose 1 (OL-D1): To occur at the Participant Decision clinic visit or at a scheduled subsequent visit.
- Open-label Dose 2 (OL-D29): To occur 28 days after Dose 1 on OL-D1.
- Open-label Clinic Visit (OL-D57): To occur 1-month after Dose 2 on OL-D29.
- And in addition, continue to comply with the Original Study Schedule of Events (Table 16, Table 17, Table 18, and Table 19) as applicable.

Participants who are unblinded, received ONLY 1 dose of mRNA-1273:

- They had a dosing error in Part A of the study that resulted in 1 dose of mRNA-1273 and 1 dose of placebo being administered.
- They did not have an AE that contraindicated the second dose of mRNA-1273 in Part A of the study.
- They did not withdraw consent in Part A of the study.
- They did not complete their second dose in Part A of the study for reasons other than the above.

If participants are eligible, and request to receive mRNA-1273 (Figure 2) will proceed to have the following clinic visits as shown in the Modified Supplemental Schedule of Events (Table 22):

- Their second dose on OL-D1: To occur at the Participant Decision clinic visit or at a scheduled subsequent visit.
- Open-label Clinic Visit (OL-D29): To occur 1-month after Dose 2 on OL-D1.
- And in addition, continue to comply with the Original Study Schedule of Events (Table 16, Table 17, Table 18, and Table 19) as applicable.

All participants remain on their original SoE to complete 24 months of follow-up after Dose 2; study participants who enter the Supplemental/Modified Supplemental SoE as described above do so in addition to their original SoE. Accordingly, all study participants will complete the full study follow-up to 24 months after the second dose of their original inoculation (mRNA-1273 or placebo).

At the point when the mRNA-1273 vaccine is no longer available for study use, any participant who schedules a Participant Decision Visit after this time-point will be unblinded (with no option to stay blinded), but will not be offered to receive mRNA-1273 study vaccine. These participants will have all of the same procedures performed outlined in Table 20 with the exception of being offered to receive mRNA-1273 vaccine There will be no scientific metric or ethical advantage for participants to remain blinded, when the majority of the study has already been unblinded. If the participant wants to receive EUA vaccine outside of the study after being unblinded, they will need to be withdrawn from the study.

After the Biologics License Application (BLA) database lock, any remaining participants who have not been unblinded will be unblinded by the Moderna study team and will receive their unblinding treatment assignment (placebo vs. mRNA vaccine) via certified letter from the site. These participants will not be required to return to the site for a NP swab or blood draw.

Figure 3: Event/Case-Driven Study with 24 Months of Planned Follow-Up

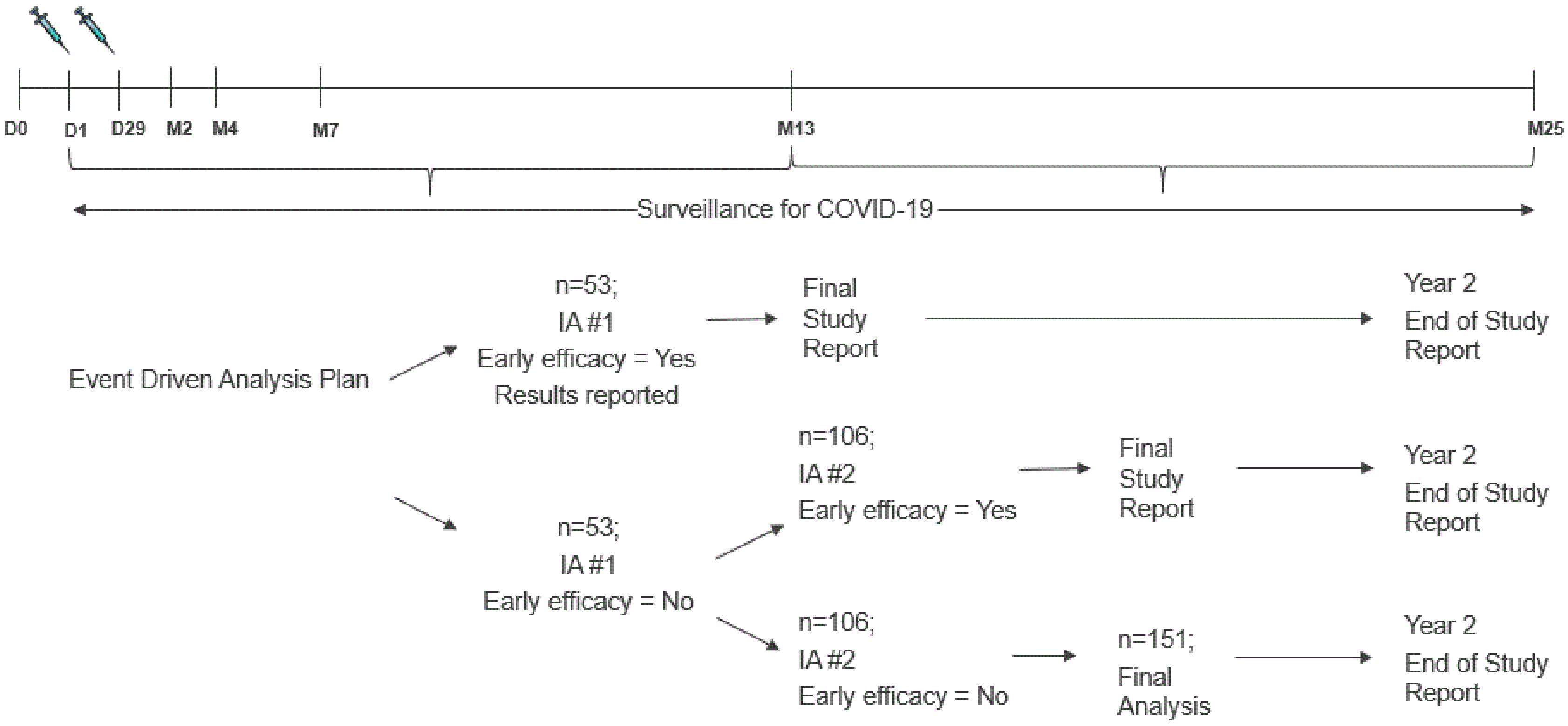

FA=final analysis; IA=interim analysis; n= number of cases

4.2. Scientific Rationale for Study Design

The mRNA-1273 vaccine is being developed to prevent COVID-19, the disease resulting from SARS-CoV-2 infection. The study is designed to primarily evaluate the clinical efficacy and safety of mRNA-1273 to prevent COVID-19 for up to 2 years after the second dose of mRNA-1273. The immunogenicity endpoints and detection of SARS-CoV-2 infection are secondary objectives.

The design and focus of the study are dependent on the current COVID-19 Pandemic, requiring identification of participant candidates at high risk of SARS-CoV-2 infection. The Sponsor may adjust the size of the study or duration of follow-up based on the blinded review of the total number of cases of COVID-19 accrued during the study, in addition to estimated percentages of study participants with immunologic evidence of SARS-CoV-2 infection at baseline. If achieving 151 cases of COVID-19 is manifestly unattainable based on plausible expansions of sample size or increased follow-up, an analysis of blinded data may be performed and change of the study design such as changing lower bound or the endpoint may be proposed.

This Phase 3 study was designed to be a randomized, stratified, observer-blind, placebo controlled-study to evaluate the efficacy, safety, and immunogenicity of mRNA-1273 SARS-CoV-2 vaccine compared to placebo in adults 18 years of age and older who have no known history of SARS-CoV-2 infection but whose locations or circumstances put them at appreciable risk of acquiring COVID-19 and/or SARS-CoV-2 infection.

Following authorization of a COVID-19 vaccine under EUA, the study design was previously amended to include a transition to Part B, the Open-Label Observational Phase (Figure 2). The demonstration of efficacy against COVID-19, and satisfactory safety data, at a time when the COVID-19 pandemic remains critical, warranted allowing those study participants who actively request to know their original assignment (placebo or mRNA-1273 vaccine) on study. Transitioning the study to Part B, the Open-Label Observational Phase, permits all ongoing study participants to (a) be informed of the availability and eligibility criteria of any COVID-19 vaccine made available under an EUA and (b) the option to offer all ongoing study participants who request unblinding an opportunity to schedule a study visit to know their original group assignment (placebo vs. mRNA-1273 vaccine). Part B, the Open-Label Observational Phase of the study, also provides the opportunity for eligible study participants who previously received placebo to actively request to receive 2 doses of mRNA-1273 vaccine (as long as investigational vaccine is available).

4.3. Choice of Dose and Control Product

The dose selected for this study (100 µg of mRNA-1273) is based on assessment of available safety and immunogenicity data from Phase 1 studies of mRNA-1647 (NCT03382405), the DMID study 20-0003 entitled "Phase I, Open-Label, Dose-Ranging Study of the Safety and Immunogenicity of 2019-nCoV Vaccine (mRNA-1273) in Healthy Adults" (NCT04283461) and the mRNA-1273-P201 study (Section 2.2.2). The schedule (2 doses administered 28 days apart) is being used in these studies.

There is no licensed SARS-CoV-2 vaccine currently available to serve as a reference control; accordingly, 0.9% sodium chloride (normal saline) injection (USP) will be used as a placebo control.

4.4. End of Study Definition

Participants are considered to have completed the study if they complete the final visit at Day 759 (Month 25), 24 months following their receipt of the original second dose of IP (where original refers to the IP received following randomization).

The study duration will be approximately 26 months for each participant. This includes a screening period of up to 1 month and a study period of 25 months that includes the first dose of IP on Day 1 and the second dose on Day 29. The participant's final scheduled visit will be on Day 759 (Month 25), 24 months after the second dose of IP on Day 29 (Month 1).

The end of study will be the final participant's final scheduled visit at Day 759 (Month 25).

5. STUDY POPULATION

The study population, adults at risk of SARS-CoV-2 infection who have no known history of SARS-CoV-2 infection, is a subset of the planned target population. Additionally, potential study participants at increased risk of complications from COVID-19 will be included since it is hypothesized that these participants might derive the greatest benefit from a vaccine. Participants ≥ 65 years of age will be eligible for enrollment with or without underlying medical conditions further increasing their risk of severe COVID-19.

Given the disproportionate disease burden of COVID-19 in racial and ethnic minorities, the study will also aim to enroll a representative sample of participants from these minority population and adjust site selection and enrollment accordingly, per FDA Draft Guidance "Enhancing the Diversity of Clinical Trial Populations - Eligibility Criteria, Enrollment Practices, and Trial Designs" (June, 2019). Study sites may be selected on the basis of SARS-COV-2 infection risk of the local population as well. Approximately 30,000 participants will be enrolled.

There will be no prospective approval of protocol deviations to recruitment and enrollment criteria (also known as protocol waivers or exemptions).

5.1. Inclusion and Exclusion Criteria (Part A: Blinded Phase and Part B: Open-Label Phase)

5.1.1. Inclusion Criteria

Participants are eligible to be included in the study only if all the following criteria apply:

1. Adults, ≥ 18 years of age at time of consent, who are at high risk of SARS-CoV-2 infection, defined as adults whose locations or circumstances put them at appreciable risk of exposure to SARS-CoV-2 and COVID-19.
2. Understands and agrees to comply with the study procedures and provides written informed consent.
3. Able to comply with study procedures based on the assessment of the investigator.
4. Female participants of nonchildbearing potential may be enrolled in the study. Nonchildbearing potential is defined as surgically sterile (history of bilateral tubal ligation, bilateral oophorectomy, hysterectomy) or postmenopausal (defined as amenorrhea for ≥ 12 consecutive months prior to Screening without an alternative medical cause). A follicle-stimulating hormone (FSH) level may be measured at the discretion of the investigator to confirm postmenopausal status (see additional information in Appendix 11.3).

5. Female participants of childbearing potential may be enrolled in the study if the participant fulfills all the following criteria:
   - Has a negative pregnancy test at Screening and on the day of the first dose (Day 1).
   - Has practiced adequate contraception or has abstained from all activities that could result in pregnancy for at least 28 days prior to the first dose (Day 1).
   - Has agreed to continue adequate contraception through 3 months following the second dose (Day 29).
   - Is not currently breastfeeding.

   Adequate female contraception is defined as consistent and correct use of a Food and Drug Administration (FDA) approved contraceptive method in accordance with the product label. For example:
   - Barrier method (such as condoms, diaphragm, or cervical cap) used in conjunction with spermicide
   - Intrauterine device
   - Prescription hormonal contraceptive taken or administered via oral (pill), transdermal (patch), subdermal, or IM route
   - Sterilization of a female participant's monogamous male partner prior to entry into the study

   Note: periodic abstinence (eg, calendar, ovulation, symptothermal, post-ovulation methods) and withdrawal are not acceptable methods of contraception.
6. *<Inclusion criterion regarding male contraception has been removed by Amendment 2>*
7. Healthy adults or adults with pre-existing medical conditions who are in stable condition. A stable medical condition is defined as disease not requiring significant change in therapy or hospitalization for worsening disease during the 3 months before enrollment.

5.1.2. Exclusion Criteria

Participants are excluded from the study if any of the following criteria apply:

1. Is acutely ill or febrile 72 hours prior to or at Screening. Fever is defined as a body temperature ≥ 38.0°C/100.4°F. Participants meeting this criterion may be rescheduled within the relevant window periods. Afebrile participants with minor illnesses can be enrolled at the discretion of the investigator.
2. Is pregnant or breastfeeding.

3. (Part A Only) Known history of SARS-CoV-2 infection.
4. Prior administration of an investigational coronavirus (SARS-CoV, MERS-CoV) vaccine or current/planned simultaneous participation in another interventional study to prevent or treat COVID-19.
5. (Part A Only) Demonstrated inability to comply with the study procedures.
6. (Part A Only) An immediate family member or household member of this study's personnel.
7. Known or suspected allergy or history of anaphylaxis, urticaria, or other significant AR to the vaccine or its excipients.
8. Bleeding disorder considered a contraindication to intramuscular injection or phlebotomy.
9. Has received or plans to receive a non-study vaccine within 28 days prior to or after any dose of IP (except for seasonal influenza vaccine which is not permitted within 14 days before or after any dose of IP, see Section 6.4.3).
10. Has participated in an interventional clinical study within 28 days prior to the day of enrollment.
11. Immunosuppressive or immunodeficient state, asplenia, recurrent severe infections (HIV-positive participants with CD4 count ≥350 cells/mm^3 and an undetectable HIV viral load within the past year [low level variations from 50-500 viral copies which do not lead to changes in antiretroviral therapy [ART] are permitted]).
12. Has received systemic immunosuppressants or immune-modifying drugs for >14 days in total within 6 months prior to Screening (for corticosteroids ≥ 20 mg/day of prednisone equivalent).
13. Has received systemic immunoglobulins or blood products within 3 months prior to the day of screening.
14. Has donated ≥ 450 mL of blood products within 28 days prior to Screening.

5.2. Participant Restrictions

Participants must not eat or drink anything hot or cold within 10 minutes before oral temperature is taken.

5.3. Screen Failures (Part A: Blinded Phase Only)

Screen failures are defined as participants who consent to participate in the clinical study but are not subsequently randomly assigned to treatment. A minimum set of screen failure information is

required to ensure transparent reporting of screen failures to meet the Consolidated Standards of Reporting Trials (CONSORT) publishing requirements and to respond to queries from regulatory authorities. Minimum information includes date of informed consent, demography, screen failure details, eligibility criteria, and information on any SAE that may have occurred from the time informed consent was obtained to the time of withdrawal.

Participants meeting the exclusion criterion #1, acutely ill or febrile prior to or at the Screening Visit (exclusion criterion #1, Section 5.1.2), may be rescheduled within the relevant window periods and will retain their initially assigned participant number.

6. STUDY TREATMENT

6.1. Investigational Product

The term "investigational product" refers to both mRNA-1273 vaccine (blinded and open label) and placebo administered in this study.

The mRNA-1273 IP is an LNP dispersion of an mRNA encoding the prefusion stabilized S protein of SARS-CoV-2 formulated in LNPs composed of 4 lipids (1 proprietary and 3 commercially available): the proprietary ionizable lipid SM-102; cholesterol; 1,2-distearoyl-sn-glycero-3-phosphocholine (DSPC); and 1-monomethoxypolyethyleneglycol-2,3-dimyristylglycerol with polyethylene glycol of average molecular weight 2000 (PEG2000-DMG). The mRNA-1273 vaccine is provided as a sterile liquid for injection and is a white to off white dispersion in appearance, at a concentration of 0.2 mg/mL in 20 mM Tris buffer containing 87 mg/mL sucrose and 10.7 mM sodium acetate at pH 7.5.

The placebo is 0.9% sodium chloride (normal saline) injection, which meets the criteria of the United States Pharmacopeia (USP).

6.2. Dosing and Management of Investigational Product

6.2.1. Method of Randomly Assigning Participants to Treatment Groups (Part A, Blinded Phase Only)

Approximately 30,000 participants will be randomly assigned in 1:1 ratio to receive either mRNA-1273 100 µg or placebo. The randomization will be in a blinded manner using a centralized Interactive Response Technology (IRT), in accordance with pre-generated randomization schedules. Only the unblinded personnel (Section 6.2.8.1) will have controlled access to which arm the participant is randomly assigned.

Dose group assignment in is summarized in Table 2.

Table 2: Summary of Treatment Groups

Treatment Groups	Investigational Product	Age (years)	Estimated Total Participants
mRNA-1273	mRNA-1273 100 µg	≥ 18	15,000
Placebo	Placebo	≥ 18	15,000
**Total**			**30,000**

6.2.1.1. Stratification

Randomization in Part A, Blinded Phase of the study will be stratified based on age and, if they are < 65 years of age, based on the presence or absence of risk factors for severe illness from COVID-19 based on CDC recommendation as of Mar 2020 (CDC 2020b). There will be 3 strata

for randomization: ≥ 65 years, < 65 years and categorized to be at increased risk ("at risk") for the complications of COVID-19, and < 65 years "not at risk." Risk will be defined based on the study participants' relevant past and current medical history. At least 25% of enrolled participants, up to 50%, will be either ≥ 65 years of age or < 65 years of age and at risk at Screening.

Participants who are < 65 years old will be categorized as at risk for severe COVID-19 illness if they have at least 1 of the following risk factors at Screening:

- Chronic lung disease (eg, emphysema and chronic bronchitis), idiopathic pulmonary fibrosis and cystic fibrosis) or moderate to severe asthma
- Significant cardiac disease (eg, heart failure, coronary artery disease, congenital heart disease, cardiomyopathies, and pulmonary hypertension)
- Severe obesity (body mass index ≥ 40 kg/m^2)
- Diabetes (Type 1, Type 2 or gestational)
- Liver disease
- HIV infection

6.2.2. Administration of Investigational Product

Investigational product will be administered as an IM injection into the deltoid muscle on a 2-dose injection schedule on Day 1 and Day 29. Each injection will have a volume of 0.5 mL and contain mRNA-1273 100 µg or saline placebo. Preferably, vaccine should be administered into the nondominant arm. The second dose of IP should be administered in the same arm as the first dose.

The IP will be prepared for injection as a single 0.5 mL dose for each participant based on the randomization assignment, as detailed in the Pharmacy Manual. Unblinded personnel who will not participate in any other aspect of the study during Part A, the Blinded Phase, will perform IP accountability, dose preparation, and IP administration. The investigator will designate unblinded medically qualified personnel (not involved in assessments of study endpoints) to administer the IP according to the procedures stipulated in this study protocol (Part A, the Blinded Phase), and Pharmacy Manual. Study-specific training will be provided. Study site personnel who were blinded during the Blinded Phase, will be unblinded at the participant level at the Participant Decision clinic visit.

At each visit when IP is administered, participants will be monitored for a minimum of 30 minutes after administration. Assessments will include vital sign measurements and monitoring for local or systemic reactions (Table 16, Table 21, and Table 22).

Eligibility for a subsequent dose of IP is determined by following the criteria outlined in Section 7.

The study site will be appropriately staffed with individuals with basic CPR training/certification. Either on site resuscitation equipment and personnel or appropriate protocols for the rapid transport of participant to a resuscitation area/facility are required.

6.2.3. Delivery and Receipt

The Sponsor or designee is responsible for the following:

- Supplying the IP
- Confirming the appropriate labeling of mRNA-1273 IP, so that it complies with the legal requirements of the US

The investigator is responsible for acknowledging the receipt of the IP by a designated staff member at the site, including the following:

- Confirming that the IP was received in good condition
- Confirmation that the temperature during shipment from the Sponsor to the investigator's designated storage location was appropriate
- Confirming whether the Sponsor has authorized the IP for use
- Ensuring the appropriate dose level of mRNA-1273 is properly prepared using aseptic technique

Further description of the IP and instructions for the receipt, storage, preparation, administration, accountability, and destruction of the IP are described in the mRNA-1273-P201 Pharmacy Manual.

6.2.4. Packaging and Labeling

The Sponsor will provide the investigator and study site with adequate quantities of mRNA-1273. The sterile vaccine product is packaged in a 10R glass vial with a 6-dose, 5.0-mL or 10-dose, 6.3-mL fill volume. mRNA-1273 vaccine will have all required labeling per regulations and will be supplied to the pharmacy in an unblinded manner. Each vial will be individually labeled for future participant identification purposes.

mRNA-1273 will be packaged and labeled in accordance with the standard operating procedures of the Sponsor or of its designee, Code of Federal Regulations Title 21 (CFR), Good Manufacturing Practice (GMP) guidelines, International Council for Harmonisation (ICH) GCP guidelines, guidelines for Quality System Regulations, and applicable regulations.

The Sponsor or Sponsor's designee will supply the 0.9% sodium chloride injection for use as both a placebo and a diluent to mRNA-1273. The 0.9% sodium chloride bears a commercial label and does not contain study-specific identification.

6.2.5. Storage

The 6-dose mRNA-1273 vaccine must be stored at 2°C to 8°C in a secure area with limited access (unblinded personnel only) and protected from moisture and light until it is prepared for administration (Section 6.2.2). The 10-dose mRNA-1273 vaccine must be stored at -20°C in a secure area with limited access (unblinded personnel only) and protected from moisture and light until it is prepared for administration. The 10-dose mRNA-1273 vaccine can be stored at 2°C to 8°C for up to 30 days, but site personnel must account for this manually as it will not be managed in the IRT system. The freezer or refrigerator should have automated temperature recording and a 24-hour alert system in place that allows for rapid response in case of freezer or refrigerator malfunction. There must be an available backup freezer or refrigerator. The freezers or refrigerators must be connected to a backup generator. In addition, vaccine accountability study staff (eg, the unblinded personnel) are required to keep a temperature log to establish a record of compliance with these storage conditions. The site is responsible for reporting any mRNA-1273 vaccine that was not temperature controlled during shipment or during storage to the unblinded site monitor. Such mRNA-1273 will be retained for inspection by the unblinded monitor and disposed of according to approved methods.

The 0.9% sodium chloride injection (USP) should be stored at 20ºC to 25ºC (68ºF to 77ºF) in a restricted access area.

6.2.6. Investigational Product Accountability

It is the investigator’s responsibility that the unblinded personnel maintain accurate records in an IP accountability log of receipt of all IP, inventory at the site, dispensing of mRNA-1273 and placebo, IP injections, and return to the Sponsor or alternative disposition of used/unused products.

An unblinded site monitor will review the inventory and accountability log during site visits and at the completion of the study. Additional details are found in the mRNA-1273-P301 Pharmacy Manual.

6.2.7. Handling and Disposal

An unblinded site monitor will reconcile the IP during the conduct and at the end of the study for compliance. Once fully reconciled at the site at the end of the study, the IP can be destroyed at the investigational site or at a Sponsor-selected third party, as appropriate.

Investigational product may be destroyed at the study site only if permitted by local regulations and authorized by the Sponsor. A Certificate of Destruction must be completed and sent to the Sponsor or designee.

6.2.8. Unblinding

6.2.8.1. Planned Unblinding

See Section 4.1.2 regarding the Participant Decision clinic visit.

- Part A, the Blinded Phase of this study is observer-blind. The investigator, study staff, study participants, site monitors, and Sponsor personnel (or its designees) will be blinded to the IP administered until study end, with the following exceptions:
- Unblinded personnel (of limited number) will be assigned to vaccine accountability procedures and will prepare IP for all participants. These personnel will have no study functions other than study vaccine management, documentation, accountability, preparation, and administration. They will not be involved in participant evaluations and will not reveal the identity of IP to either the participant or the blinded study site personnel involved in the conduct of the study unless this information is necessary in the case of an emergency.
- Unblinded medically qualified study site personnel will administer the IP. They will not be involved in assessments of any study endpoints.
- The dosing assignment will be concealed by having the unblinded personnel prepare the IP in a secure location that is not accessible or visible to other study staff. An opaque sleeve over the syringe used for injection will maintain the blind at the time of injection, as the doses containing mRNA-1273 will look different than placebo. Only delegated unblinded site staff will conduct the injection procedure. Once the injection is completed, only the blinded study staff will perform further assessments and interact with the participants. Access to the randomization code will be strictly controlled at the pharmacy.
- Unblinded site monitors, not involved in other aspects of monitoring, will be assigned as the IP accountability monitors. They will have responsibilities to ensure that sites are following all proper IP accountability, preparation, and administration procedures.
- An unblinded statistical and programming team will perform the pre-planned IAs (Section 9.6).
- An independent DSMB will review the interim data to safeguard the interests of clinical study participants and to help ensure the integrity of the study. The DSMB will review unblinded statistical outputs and IA results, provided by the independent unblinded statistician, and make recommendations to the Sponsor (Section 8.4.2).

- At the initiation of Part B, the Open-Label Observational Phase of this study (Section 4.1.3), study site personnel who were blinded during the Blinded Phase will be unblinded at the participant level at the Participant Decision clinic visit.

If prespecified criteria for early efficacy are met by an IA or if the primary efficacy analysis is completed based on accrual of prespecified COVID-19 cases, pre-identified Sponsor and CRO team members responsible for the analysis and reporting will be unblinded to treatment assignments in order to prepare a final study report. In order to maintain an observer-blind design, investigators, site staff, participants, and Sponsor and CRO staff with oversight of study conduct will remain blinded to treatment allocation for the study duration. All study participants will be followed for efficacy and safety endpoints through the remainder of planned study period and results will be summarized in an end of study report (Sections 4.1 and 9.1).

6.2.8.2. Unplanned Unblinding

A participant or participants may be unblinded in the event of an SAE or other severe event, or if there is a medical emergency requiring the identity of the product to be known to properly treat a participant. If a participant becomes seriously ill or pregnant during the study, the study investigator may request that the blind will be broken if knowledge of the administered vaccine will affect that participant’s dosing options. In this situation or in the event of a medical emergency requiring identification of the IP administered to an individual participant, the investigator will make every attempt to contact the Sponsor medical lead to explain the need for opening the code within 24 hours of opening the code. The investigator will be responsible for documenting the time, date, reason for the code break, and the names of the personnel involved.

In addition to the situations described above, where the blind may be broken, the data will also be unblinded to a statistical team at specified time points for IAs as outlined in Section 9.6.

In December 2020, COVID-19 vaccines have started to become available under EUA as an alternative option to some participants based on evolving recommended populations by the CDC and local supply chain distribution. While all participants should be encouraged to stay blinded in the study for as long as possible, their participation should not otherwise deny them the opportunity to receive a COVID-19 vaccine under EUA. Clinical investigators can exercise discretion as to whether individual participants should be unblinded upon request to allow them to make an informed decision regarding receipt of a COVID-19 vaccine outside of this study. Investigator judgment should consider a participant’s risk status under CDC recommendations, any current local public health guidance, and their access to imminently receive a COVID-19 vaccine under an EUA.

If a decision is made to unblind a participant to support an informed decision to receive a COVID-19 vaccine under an EUA outside of this study, investigators are asked to take the following steps:

- Obtain a final assessment of safety from the participant to collect and resolve any outstanding safety experience. Verbal interview is sufficient.
- Unblind the participant in the IRT and inform the participant of their treatment assignment.
- Inform the Sponsor Medical Lead within 24 hours to confirm rationale for unblinding.

Participants who are confirmed to have received placebo upon unblinding and intend to receive a COVID-19 vaccine under an EUA outside of this study will be withdrawn from the study at the point of unblinding (Section 7.3.1).

Note: Participants who requested unblinding because of a stated imminent access to a COVID-19 vaccine under an EUA outside of this study, were withdrawn from study, and then were unable to in fact receive a COVID-19 vaccine, will be permitted to re-enter this study upon re-consent to receive the mRNA-1273 vaccine, once they are eligible (as long as investigational vaccine is available).

6.3. Study Treatment Compliance

All doses of IP will be administered at the study site under direct observation of unblinded medically qualified study personnel and appropriately recorded (date and time) in the eCRF. Unblinded personnel will confirm that the participant has received the entire dose of vaccine. If a participant does not receive vaccine or does not receive all of the planned doses, the reason for the missed dose will be recorded. Data will be reconciled with site accountability records to assess compliance.

Participants who miss the second dose of IP due to noncompliance with the visit schedule and not due to a safety pause will still be required to follow the original visit and testing schedule as described in the protocol. Unless consent is withdrawn, a participant who withdraws or is withheld from receiving the second dose of study vaccine will remain in the study and complete all efficacy, safety and immunogenicity assessments required through the participant’s scheduled end of study.

The study site is responsible for ensuring that participants comply with the study windows allowed. If a participant misses a visit, every effort should be made to contact the participant and complete a visit within the defined visit window (SoE Tables, Section 11.1). If a participant does not complete a visit within the time window, that visit will be classified as a missed visit (with the exception of Dose 2 visits in Part A and Part B, as applicable) and the participant will continue with subsequent scheduled study visits. All safety requirements of the missed visit will be captured

and included in the subsequent visit (eg, clinical laboratory testing, eDiary review for reactogenicity, immunologic testing, as applicable).

6.4. Prior and Concomitant Therapy

6.4.1. Prior Medications and Therapies

Information about prior medications (including any prescription or over-the-counter medications, vaccines, or blood products) taken by the participant within the 28 days before providing informed consent (or as designated in the inclusion/exclusion requirements) will be recorded in the participant's eCRF.

6.4.2. Concomitant Medications and Therapies

Study site staff must question the participant regarding any medications taken and vaccinations received by the participant and record the following information in the eCRF:

- All non-study vaccinations administered within the period starting 28 days before the first dose of IP.
- Seasonal influenza vaccine administered for the current influenza season (typically October through April in the Northern Hemisphere).
- Part A only: All concomitant medications and non-study vaccinations taken through 28 days after each dose of IP. Antipyretics and analgesics taken prophylactically (ie, taken in the absence of any symptoms in anticipation of an injection reaction) will be recorded as such.
- Any concomitant medications used to prevent or treat COVID-19.
- Any concomitant medications relevant to or for the treatment of an SAE or a MAAE.
- Part A only: Participant will be asked in the eDiary if they have taken any antipyretic or analgesic to treat or prevent fever or pain within 7 days after each IP injection, including the day of dosing. Reported antipyretic or analgesic medications should be recorded in the source document by the site staff during the post-injection study visits or via other participant interactions (eg, phone calls).

6.4.3. Concomitant Medications and Vaccines that May Lead to the Elimination of a Participant from Per-protocol Analyses

The use of the following concomitant medications and/or vaccines will not require withdrawal of the participant from the study but may determine a participant's eligibility to receive a second dose or evaluability in the per-protocol (PP) analysis (Analysis Sets are described in Section 9.4):

- Any investigational or nonregistered product (drug or vaccine) other than the study vaccine used during the study period.
- A non-study vaccine administered during the period from 28 days before through 28 days after each dose of IP or any seasonal influenza vaccine that was administered within 14 days before or after any dose of IP.
- Immunoglobulins and/or any blood products administered during the study period (except for treatment of COVID-19).
- Medications that suppress the immune system (except for treatment of COVID-19).

If a participant takes a prohibited drug therapy, the investigator and the contract research organization's (CRO's) medical monitor will make a joint decision about continuing or withholding further dosing from the participant based on the time the medication was administered, the drug's pharmacology and pharmacokinetics, and whether use of the medication will compromise the participant's safety or the interpretation of data. It is the investigator's responsibility to ensure that details regarding the concomitant medications are adequately recorded in the eCRF.

All medication and interventions necessary for the appropriate care for the study participant, particularly to treat COVID-19, should be administered and appropriately documented along with the AE.

7. DELAYING OR DISCONTINUING STUDY TREATMENT AND PARTICIPANT WITHDRAWAL FROM THE STUDY

7.1. Criteria for Delay of Study Treatment

Body temperature must be measured at the Day 1 and Day 29 visits prior to any study treatment administration. The following events constitute criteria for delay of study treatment, and if either of these events occur at the time scheduled for dosing, the participant may be injected at a later date within the time window specified in Table 16, or the participant may be discontinued from dosing at the discretion of the investigator (Section 7.2):

- Acute moderate or severe infection with or without fever at the time of dosing
- Fever, defined as body temperature ≥ 38.0°C (100.4°F) at the time of dosing

Participants with a minor illness without fever, as assessed by the investigator, can be administered IP. Participants with a fever of 38.0°C (100.4°F) or higher will be contacted within the time window acceptable for participation and reevaluated for eligibility. If the investigator determines that the participant's health on the day of administration temporarily precludes dosing with IP, the visit should be rescheduled within the allowed interval for that visit.

7.2. Discontinuation of Study Treatment

Every reasonable attempt will be made to follow up with participants for safety throughout the entire study period, even if further dosing is discontinued or the participant misses one or more visits. Unless consent is withdrawn, a participant who withdraws or is withheld from receiving the second dose of study vaccine will remain in the study and complete all scheduled visits and assessments (Section 11.1).

The investigator, in consultation with the Sponsor's medical monitor, may withhold a participant from further dosing if the participant experiences any of the following:

- Becomes pregnant (Section 8.3.5)
- Develops, during the course of the study, symptoms or conditions listed in the exclusion criteria
- Experiences an AE (other than reactogenicity) after dosing that is considered by the investigator to be related to IP (Section 8.3.4) and is of Grade 3 (severe) or greater intensity (Section 8.3.8)
- Experiences an AE or SAE that, in the judgment of the investigator, requires study IP withdrawal due to its nature, severity, or required treatment, regardless of the causal relationship to vaccine
- Experiences a clinically abnormal vital sign measurement or finding on physical examination, or general condition that, in the judgment of the investigator, requires IP withdrawal

The reason(s) for discontinuation from further dosing will be recorded in the eCRF.

Prior to receiving a second dose of IP, participants will be reassessed to ensure that they continue to meet eligibility requirements as outlined below.

The following events in a participant constitute absolute contraindications to any further dosing of the IP to that participant. If any of these events occur during the study, the participant must not receive additional doses of vaccine but will be encouraged to continue study participation for safety through 24 months following last dose.

- Part A only: Diagnosed COVID-19 by detection of SARS-CoV-2 in Day 1 NP swab or COVID-19 diagnosed prior to Day 29. If COVID-19 is suspected on or prior to Day 29, further administration of IP must be withheld until COVID-19 test results are available.
- Anaphylaxis or systemic hypersensitivity reaction following the administration of vaccine.
- Any SAE judged by investigator or Sponsor to be related to study vaccine.
- Any clinically significant medical condition that, in the opinion of the investigator, poses an additional risk to the participant if he/she continues to participate in the study.

The following events constitute contraindications to administration of study vaccine at certain points in time, and if any of these events occur at the time scheduled for dosing, the participant may be injected at a later date, within the time window specified in the SoE (Table 16), or the participant may be withdrawn from dosing at the discretion of the investigator (Section 7.3):

- Acute moderate or severe infection with or without fever at the time of dosing
- Fever, defined as body temperature ≥ 38.0°C (100.4°F) at the time of dosing
- Part B only: Diagnosed COVID-19 by detection of SARS-CoV-2 by RT-PCR and accompanying symptoms. Participant must be asymptomatic at the time of dosing although may still be RT-PCR positive, with the exception of loss of sense of smell or taste and fatigue, if by the investigator’s judgement these are deemed to be mild chronic sequelae.

Participants with a minor illness without fever, as assessed by the investigator, can be administered IP. Participants with a fever of 38.0°C (100.4°F) or higher will be contacted within the time window acceptable for participation and reevaluated for eligibility.

7.3. Participant Withdrawal from the Study

7.3.1. Participant Withdrawal

Participants who withdraw from the study will not be replaced. A "withdrawal" from the study refers to a situation wherein a participant does not return for the final visit planned in the protocol. The statistical management of participant withdrawals is discussed in Section 9.

Participants can withdraw consent and withdraw from the study at any time, for any reason, without prejudice to further treatment the participant may need to receive. The investigator will request that the study participant complete all study procedures pending at the time of withdrawal.

If participant desires to withdraw from the study because of an AE, the investigator will try to obtain agreement to follow up with the participant until the event is considered resolved or stable and will then complete the end of study eCRF.

Information related to the withdrawal will be documented in the eCRF. The investigator will document whether the decision to withdraw a participant from the study was made by the participant or by the investigator, as well as which of the following possible reasons was responsible for withdrawal:

- AE (specify)
- SAE (specify)
- Death
- Lost to follow-up
- Physician decision (specify)
- Pregnancy
- Protocol deviation
- Study terminated by Sponsor
- Withdrawal of consent by participant (specify); this includes participants who, at the Participant Decision clinic visit, withdraw consent from continuing in the study.
- Other (specify)

Participants who are withdrawn from the study because of AEs (including SAEs) must be clearly distinguished from participants who are withdrawn for other reasons. Investigators will follow up with participants who are withdrawn from the study as result of an SAE or AE until resolution of the event.

Participants who are confirmed to have received placebo upon unblinding and intend to receive a COVID-19 vaccine under an EUA outside of this study will be withdrawn from the study.

A participant withdrawing from the study may request destruction of any samples taken and not tested, and the investigator must document this in the site study records.

If the participant withdraws consent for disclosure of future information, the Sponsor may retain and continue to use any data collected before such a withdrawal of consent (see Section 11.2.10).

The Sponsor will continue to retain and use all research results that have already been collected for the study evaluation, unless the participant has requested destruction of these samples. All biological samples that have already been collected may be retained and analyzed at a later date (or as permitted by local regulations).

7.4. Lost to Follow-up

Participants will be considered lost to follow-up (LTFU) if they repeatedly fail to return for scheduled visits without stating an intention to withdraw consent and they cannot be contacted by the study site. The following actions must be taken if a participant fails to return to the clinic for a required study visit:

- The site must attempt to contact the participant and reschedule the missed visit as soon as possible, counsel the participant on the importance of maintaining the assigned visit schedule and ascertain whether the participant wishes to and/or should continue in the study.
- Before a participant is deemed lost to follow-up, the investigator or designee must make every effort to regain contact with the participant (where possible, 3 telephone calls and, if necessary, a certified letter to the participant's last known mailing address or local equivalent methods). These contact attempts (eg, dates of telephone calls and registered letters) should be documented in the participant's medical record. A participant should not be considered LTFU until these efforts have been made.
- Should the participant continue to be unreachable, he/she will be considered to have withdrawn from the study.

8. STUDY ASSESSMENTS AND PROCEDURES

Before performing any study procedures, all potential participants will sign an ICF (as detailed in Section 11.2.6). Participants will undergo study procedures at the time points specified in the SoEs (Section 11.1).

A participant also can be seen for an unscheduled visit at any time during the study. An unscheduled visit may be prompted by reactogenicity issues, Illness Visit criteria for COVID-19, or new or ongoing AEs. The site also has the discretion to make reminder telephone calls or send text messages to inform the participant about visits, review eDiary requirements, or follow-up on ongoing or outstanding issues.

In accordance with "FDA Guidance on Conduct of Clinical Trials of Medical Products during the COVID-19 Pandemic" (DHHS 2020), investigators may convert study site visits to telemedicine visits with the approval of the Sponsor. Such action should be taken to protect the safety and well-being of study participants and study site staff or to comply with state or municipal mandates.

General considerations for study assessments and procedures include the following:

- Protocol waivers or exemptions are not allowed. The study procedures and their timing must be followed as presented in Section 11.1. Adherence to the study design requirements is essential and required for study conduct.
- Immediate safety concerns should be discussed with the Sponsor immediately upon occurrence or awareness to determine if the participant should continue study treatment or participation in the study.
- All screening evaluations must be completed and reviewed to confirm that potential participants meet all eligibility criteria. The investigator will maintain a screening log to record details of all participants screened and to confirm eligibility or record reasons for screening failure, as applicable.
- Procedures conducted as part of the participant's routine clinical management (eg, blood count) and obtained before signing of the ICF may be utilized for screening or baseline purposes provided the procedures met the protocol-specified criteria and were performed within the time frame defined in the SoE.

8.1. Efficacy and Immunogenicity Assessments and Procedures

8.1.1. Efficacy Assessments Related to COVID-19 and SARS-CoV-2 Infection

Each study participant will have an NP swab sample collected for SARS-CoV-2 testing by RT-PCR on Day 1 and Day 29, prior to receiving a dose of the IP as specified in the SoE (Section 11.1).

COVID-19:

To be considered as a case of COVID-19 for the evaluation of the Primary Efficacy Endpoint, the following case definition must be met:

- The participant must have experienced at least TWO of the following systemic symptoms: Fever (≥ 38ºC), chills, myalgia, headache, sore throat, new olfactory and taste disorder(s), OR
- The participant must have experienced at least ONE of the following respiratory signs/symptoms: cough, shortness of breath or difficulty breathing, OR clinical or radiographical evidence of pneumonia; AND
- The participant must have at least one NP swab, nasal swab, or saliva sample (or respiratory sample, if hospitalized) positive for SARS-CoV-2 by RT-PCR.

Severe COVID-19:

To be considered severe COVID-19, the following criteria must be met:

- Confirmed COVID-19 as per the Primary Efficacy Endpoint case definition, plus any of the following:
  - Clinical signs indicative of severe systemic illness, Respiratory Rate ≥ 30 per minute, Heart Rate ≥ 125 beats per minute, SpO_2 ≤ 93% on room air at sea level or PaO_2/FIO_2 < 300 mm Hg, OR
  - Respiratory failure or Acute Respiratory Distress Syndrome (ARDS), (defined as needing high-flow oxygen, non-invasive or mechanical ventilation, or ECMO), evidence of shock (systolic blood pressure < 90 mmHg, diastolic BP < 60 mmHg or requiring vasopressors), OR
  - Significant acute renal, hepatic or neurologic dysfunction, OR
  - Admission to an intensive care unit or death.

The secondary case definition of COVID-19 is defined as the following systemic symptoms: fever (temperature ≥ 38ºC), or chills, cough, shortness of breath or difficulty breathing, fatigue, muscle aches, or body aches, headache, new loss of taste or smell, sore throat, nasal congestion or

rhinorrhea, nausea, or vomiting or diarrhea AND a positive NP swab, nasal swab, or saliva sample (or respiratory sample, if hospitalized) for SARS-CoV-2 by RT-PCR.

Death attributed to COVID-19 is defined as any participant who dies during the study with a cause directly attributed to a complication of COVID-19.

SARS-CoV-2 Infection:

- SARS-CoV-2 infection is defined by seroconversion due to infection measured by bAb against SARS-CoV-2 nucleocapsid. Seroconversion is defined differently for participants seronegative at Baseline and seropositive at Baseline:
    - Participants seronegative at Baseline: bAb levels against SARS-CoV-2 nucleocapsid either from below the limit of detection (LOD) or lower limit of quantification (LLOQ) at Study Day 1 that increase to above or equal to LOD or LLOQ starting at Study Day 57 or later.
    - Participants seropositive at Baseline: bAb levels against SARS-CoV-2 nucleocapsid above the LOD or LLOQ at study Day 1 that increase by 4-fold or more in participants starting at Study Day 57 or later.

8.1.2. Surveillance for COVID-19 Symptoms

Surveillance for COVID-19 symptoms will be conducted via weekly telephone calls in Part A, Blinded Phase and Part B, Open-Label Observational Phase of the study and/or eDiary prompts as specified in Section 8.2.2; Figure 4 starting after enrollment and throughout the study. If there is no response to an eDiary prompt for 2 days, the site staff will contact the study participant by phone.

According to the CDC as of 10 June 2020 (CDC 2020c), patients with COVID-19 have reported a wide range of symptoms ranging from mild symptoms to severe illness. Throughout the study, to surveil for COVID-19, the following prespecified symptoms that meet the criteria for suspicion of COVID-19 will be elicited weekly from the participant and the presence of any one of these symptoms lasting at least 48 hours (except for fever and/or respiratory symptoms) will result in the site arranging an Illness Visit to collect an NP swab within 72 hours:

- Fever (temperature ≥ 38ºC) or chills (of any duration, including ≤ 48 hours)
- Shortness of breath or difficulty breathing (of any duration, including ≤ 48 hours)
- Cough (of any duration, including ≤ 48 hours)
- Fatigue
- Muscle or body aches

- Headache
- New loss of taste or smell
- Sore throat
- Congestion or runny nose
- Nausea or vomiting
- Diarrhea

It is important to note that some of the symptoms of COVID-19 overlap with solicited systemic ARs that are expected after vaccination with mRNA-1273 (eg, myalgia, headache, fever, and chills). During the first 7 days after vaccination, when these solicited ARs are common, investigators should use their clinical judgement to decide if an NP swab should be collected (Part A, the Blinded Phase only). The collection of an NP swab prior to the Day 1 and Day 29 vaccination can help ensure that cases of COVID-19 are not overlooked. Any study participant reporting respiratory symptoms during the 7-day period after vaccination should be evaluated for COVID-19.

Figure 4: Surveillance for COVID-19 Symptoms and the Corresponding Clinical Data Pathways

Symptoms that meet the criteria for suspicion of COVID-19 infection

- Fever (temperature ≥ 38°C) or chills
- Cough
- Shortness of breath or difficulty breathing
- Fatigue
- Muscle or body aches
- Headache
- New loss of taste or smell
- Sore throat
- Congestion or runny nose
- Nausea or vomiting
- Diarrhea

Elicited through interaction with the (in person or phone) and/or clinical evaluation by investigator

- Clinical signs indicative of severe systemic illness, Respiratory Rates ≥ 30 per minute, Heart Rate ≥ 125 beats per minute, SpO2 ≤ 93% on room air at sea level or PaO2/FIO2 < 300 mm Hg, OR
- Respiratory failure or Acute Respiratory Distress Syndrome (ARDS), (defined as needing high-flow oxygen, non-invasive or mechanical ventilation, or ECMO), evidence of shock (systolic blood pressure < 90 mmHg, diastolic BP < 60 mmHg or requiring vasopressors), OR
- Significant acute renal, hepatic or neurologic dysfunction, OR
- Admission to an intensive care unit or death.

From Medically-Attended HCP Visit (site or external)

- Positive virologic result by RT-PCR for SARS-CoV-2 infection

PCR results on NP Swab, Nasal Swab, or Saliva Sample

During the course of the study, participants with symptoms of COVID-19 will be asked to return within 72 hours or as soon as possible to the study site or medically qualified staff from the study site will conduct a home visit as soon as possible to collect an NP swab sample (for RT-PCR), collect a blood sample for immunologic analysis of SARS-CoV-2 infection, and to evaluate for COVID-19. Both study site visits and home visits are referred to as Illness Visits. The NP swab sample will also be tested for the presence of other respiratory infections.

In addition, the study site may collect an additional respiratory sample for SARS-CoV-2 testing to be able to render appropriate medical care for the study participant as determined by local standards of care. If neither a study site visit or home visit is possible, participants will be sent a

saliva kit via courier or other Sponsor-approved method. The study site will arrange to retrieve a saliva (or nasal swab) sample by local courier or other Sponsor-approved method, and the sample will be tested by RT-PCR for SARS-CoV-2. If participants are confirmed to have SARS-CoV-2 infection, the investigator will notify the participant, and the participant's primary care physician, of the diagnosis. If the study participant does not have a primary care physician, the investigator will assist them to obtain one. The participant will also be instructed on infection prevention measures consistent with local public health guidance.

If scheduled, a study site Illness Visit may include assessments such as medical history, physical examination, blood sampling for clinical laboratory testing, and NP swab sampling for viral PCR (including multiplex PCR for respiratory viruses including SARS-CoV-2) to evaluate the severity of the clinical case. Radiologic imaging studies may be conducted. Blood samples will be collected for potential future immunologic assessment of SARS-CoV-2 infection.

Cases are defined as participants meeting clinical criteria based both on symptoms for COVID-19 and on RT-PCR detection of SARS-CoV-2 from samples collected within 72 hours of the study participant reporting symptoms meeting the definition of COVID-19. Participants who are hospitalized for COVID-19 without the opportunity for a clinic or home visit will also be considered cases, assuming that the symptomology criteria for COVID-19 are met and a respiratory sample is positive for SARS-CoV-2 by PCR at a Clinical Laboratory Improvement Amendments (CLIA)-certified or CLIA-certified waiver laboratory. Investigators are encouraged to try to obtain a respiratory sample during the course of hospitalization for submission to the study central laboratory, if feasible. The investigator should determine if the criteria for severe COVID-19 has been met.

Evidence of severe COVID-19 is defined as in Section 8.1.1.

All clinical findings will be recorded in the eCRF. All confirmed cases of COVID-19 will be captured as MAAEs, along with relevant concomitant medications and details about severity, seriousness, and outcome.

8.1.3. Convalescent Period Starting with the Illness Visit

All study participants who experience COVID-19 symptoms and subsequently present for an Illness Visit (in-clinic or at home) will be given an instruction card listing symptoms and severity grading system, a thermometer, an oxygen saturation monitor, and saliva collection tubes. Participants will be trained on the use of the oxygen saturation monitor and how to take saliva specimens. The list of symptoms is presented in Section 8.1.2 and the severity scoring system is presented in Table 3.

Table 3: Grading of COVID-19 Symptoms

Grading	All Symptoms	For Nausea/Vomiting ONLY	For Sense of Smell/Taste ONLY
None	No symptom		
Mild	I had the symptom, but I could still do my normal activities	I was able to eat and drink normally	I had the symptom, but I retained some taste/smell
Moderate	The symptom really bothered me. It was hard to do my normal activities	It bothered me enough that I did not eat or drink normally	My taste/smell was significantly affected
Severe	The symptom was very bad. I was not able to do activities that I usually do	I could not eat or drink	I have no taste or smell

The initial Illness Visit is considered Day 1 for the Convalescent Period. Starting on Day 2 of the Convalescent Period, the investigator (or medically qualified staff appropriately delegated by the investigator) will contact participants daily with telemedicine visits through Day 14 or until symptoms have resolved, whichever is later. Telemedicine visits may be conducted by videoconference or by audio only (telephone). During the telemedicine visit (preferably done in the evening), the participant will be asked to verbally report the severity of each symptom, their highest body temperature and lowest oxygen saturation for that day, and the investigator will determine if medical attention is required due to worsening of COVID-19 symptoms. The presence and severity of each symptom reported by the participant will be noted in the appropriate source document (Table 19).

During the telemedicine visits, participants will be reminded both to collect their own saliva (or nasal swab) sample on 3, 5, 7, 9, 14, and 21 days after the initial Illness Visit and to return the sample to the study site. Immediately upon receipt of a saliva (or nasal swab) sample, the study site will send it for testing to the study central virology laboratory.

During the telemedicine visits, if the participant has a positive result for SARS-CoV-2 from the Day 1 Illness Visit, the participant will continue the Convalescent Period. If the participant has a negative result for SARS-CoV-2 from the Day 1 Illness Visit, the participant will exit the Convalescent Period, including discontinuation of daily telemedicine visits and collection of saliva (or nasal swab) samples, and will return to their respective study schedule (Table 16, Table 17, and Table 18). Participants who are pending a central laboratory PCR may exit from the convalescent period based on a negative PCR result from a CLIA-certified or CLIA-certified waiver local laboratory, at the investigator’s discretion.

All participants confirmed to be COVID-19 cases will be scheduled for a convalescent visit (study site or home visit) 28 days after the initial Illness Visit. At this visit, a saliva (or nasal swab) sample

will be collected and a blood sample will be drawn for immunologic assessment of SARS-CoV-2 infection (Table 19).

If the participant is hospitalized, medically qualified site personnel will try to obtain medical records and SARS-CoV-2 diagnostic results and document if the criteria for COVID-19 or severe COVID-19 have been met. If the participant is later discharged from the hospital during the 28-day period following diagnosis of COVID-19, the study site personnel will arrange for a resumption of a schedule for telemedicine visits and sampling for the remainder of the Convalescent Period, followed by a return to their respective study schedule (Table 16, Table 17, and Table 18).

8.1.4. Ancillary Supplies for Participant Use

Study sites will distribute Sponsor-provided oral thermometers and rulers for use by participants in assessing body temperature and injection site reactions for recording solicited ARs in eDiaries (Section 8.2.2). Based on availability, smartphone devices may be provided to those participants who do not have their own device to use for eDiary activities.

Participants will also receive the following Sponsor-provided supplies at Illness Visits where COVID-19 is suspected:

- An instruction card listing symptoms and severity grading system
- A pulse oximeter for measuring oxygen saturation
- Saliva collection tubes and instructions/means for returning saliva samples collected at home to the study site
- Additional oral thermometer, if required.

8.1.5. Immunogenicity Assessments

Blood samples for immunogenicity assessments will be collected at the time points indicated in the SoEs (Section 11.1). On Day 1 and Day 29, as specified in Table 16, on OL-D1 and OL-D57 as specified in Table 21, and on OL-D1 and OL-D29 as specified in Table 22, blood samples for immunogenicity assessment will be collected before administration of study treatment. The following analytes will be measured:

- Serum bAb levels against SARS-CoV-2 as measured by ligand-binding assay specific to the SARS-CoV-2 S protein
- Serum bAb levels against SARS-CoV-2 as measured by ligand-binding assay specific to the SARS-CoV-2 nucleocapsid protein
- Serum nAb titer against SARS-CoV-2 as measured by pseudovirus and/or live virus neutralization assays

Serum will be tested using the ligand-binding assay specific to the SARS-CoV-2 nucleocapsid to determine the immunologic status of study participants at baseline and assess for seroconversion due to infection during the course of the study. Serum from a subset of participants will be tested in the other assays. The selection of the subset and timepoints to be tested will be described in the statistical analysis plan (SAP). Sample aliquots will be designed to ensure that backup samples are available and that adequate vial volumes may allow for further testing. The actual time and date of each sample collected will be recorded in the eCRF, and unique sample identification will be used to maintain the blind at the laboratory at all times and to allow for automated sample tracking and storage. Handling and preparation of the samples for analysis, as well as shipping and storage requirements, will be provided in a separate study manual.

The ligand-binding assay and measurement of nAb titers will be performed in laboratories designated by the Sponsor.

8.2. Safety Assessments

Safety assessments will include monitoring and recording of the following for each participant:

- Solicited local and systemic ARs (Section 8.3.4) that occur during the 7 days following each injection (ie, the day of dosing and 6 subsequent days). Solicited ARs will be recorded daily using eDiaries (Section 8.2.2); (Part A, the Blinded Phase only).
- Unsolicited AEs observed or reported during the 28 days following each dose of IP (ie, the day of dosing and 27 subsequent days, (Part A, the Blinded Phase only). Unsolicited AEs are AEs that are not included in the protocol-defined solicited ARs (Section 8.3.4).
- AEs leading to discontinuation from dosing and/or study participation from Day 1 through Day 759 or withdrawal from the study.
- MAAEs from Day 1 through Day 759 or withdrawal from the study.
- SAEs from Day 1 through Day 759 or withdrawal from the study.
- Abnormal vital sign measurements.
- Physical examination findings.
- Pregnancy and accompanying outcomes.
- Concomitant medications and non-study vaccinations.

8.2.1. Safety Phone Calls

A safety phone call is a telephone call made to the participant by medically qualified study staff. Medically qualified staff are those appropriately delegated individuals who are permitted to elicit verbal medical history from participants based on local regulations and local licensing requirements.

This call will follow a script, which will facilitate the collection of relevant safety information. The participant will be interviewed according to the script about occurrence of unsolicited AEs, MAAEs, SAEs, or AEs leading to study withdrawal and concomitant medications associated with those events, receipt of any non-study vaccinations, and pregnancy. Occurrence of AEs will only be collected by safety phone call during the Vaccination Phase (Table 16 and Table 21).

The timing of the safety phone calls and the relevant safety information collected is provided in the SoEs (Section 11.1).

All safety information described by the participant must be documented in source documents and not documented on the script used for the safety telephone contact. All AEs, MAAEs, SAEs, and AEs leading to study withdrawal must be recorded in the eCRF as specified in Section 8.3.6.

8.2.2. Use of Electronic Diaries

In Part A, the Blinded Phase of the Study, at the time of consent, the participants must confirm they will be willing to complete an eDiary (for 7-day reactogenicity and to surveil weekly for COVID-19 symptoms) using either an application downloaded to their smartphone or using a device that is provided at the time of enrollment. This study will utilize the Medidata Patient Cloud Application as the eDiary for both collection of 7-day reactogenicity and weekly eDiary prompts to elicit an unscheduled Illness Visit if the participant is experiencing COVID-19 symptoms. This application allows for real-time data collection on a 21 CFR Part 11 compliant system directly from participants.

In Part A, the Blinded Phase of the Study, before enrollment on Day 1, the participant will be instructed to download the eDiary application on their personal smartphone or will be provided a Sponsor-provisioned device to record solicited ARs (Section 8.3.4) and also to be utilized for eDiary prompts through the COVID-19 surveillance period.

In Part A, the Blinded Phase of the Study, at each dosing visit, participants will record data into the eDiary starting approximately 30 minutes after dosing under supervision of the study site staff to ensure successful entry of assessments. The 30-minute assessment is an opportunity for site staff to train the participant. The site staff will perform any retraining as necessary. Study participants will continue to record data in an eDiary after they leave the study site, preferably in the evening and at the same time each day, on the day of dosing and for 6 days following dosing.

In Part A, the Blinded Phase of the Study, the following local ARs will be solicited by the eDiary: pain at injection site, erythema (redness) at injection site, swelling/induration (hardness) at injection site, and localized axillary swelling or tenderness ipsilateral to the injection arm.

The following systemic ARs will be solicited by the eDiary: headache, fatigue, myalgia (muscle aches all over the body), arthralgia (aching in several joints), nausea/vomiting, body temperature (potentially fever), and chills.

In Part A, the Blinded Phase of the Study, solicited local and systemic reactogenicity ARs, as defined in Section 8.3.4, will be collected on the day of each IP injection and during the 7 days after IP injection (ie, the day of dosing and 6 subsequent days). Any solicited AR that is ongoing beyond Day 7 will be reported in an eDiary until resolution. Adverse reactions recorded in diaries beyond Day 7 should be reviewed by study site staff either during the next scheduled phone call or at the next study site visit (Table 16).

If eDiary prompts result in identification of relevant safety events according to the study period-or symptoms of COVID-19, a follow-up safety call will be triggered.

In Part A, the Blinded Phase of the Study, at each dosing visit, participants will be instructed or reminded on thermometer usage to measure body temperature, ruler usage to measure injection site erythema and swelling/induration (hardness), and self-assessment for localized axillary swelling or tenderness on the same side as the injection arm.

In Part A, the Blinded Phase of the Study, daily oral body temperature measurement should be performed at approximately the same time each day using the thermometer provided by the study site.

Surveillance for signs and symptoms of COVID-19 will start at Part A Day 1 and continue through the entire Surveillance Phase of the study (Table 17 and Table 18). After participants complete Part A Vaccination Phase of the Study (Table 16 and Table 21), the weekly eDiary Safety Follow-up Prompts will be triggered on Day 61 (Table 17). The weekly eDiary prompts will utilize the same Medidata Patient Cloud Application. Each week (ie, every 7 days) the participant will receive a prompt on their smartphone device to regularly surveil for signs and symptoms of COVID-19 and any other changes in health status. The participant will be trained on how to complete the weekly eDiary prompts at the Part A Day 57 clinic visit and also reminded to call the site immediately if they experience any COVID-19 symptoms. The weekly eDiary prompt will inquire about the following:

- Changes in health since the last time completing the weekly eDiary prompt or the last contact with the study staff
- Any known exposure to someone with SARS-CoV-2 infection or COVID-19 since the last time completing the weekly eDiary prompt or the last contact with the study staff
- Capture of any COVID-19 symptoms currently being experienced or experienced since the last time completing the weekly check-in as defined in Section 8.1.2
- Any contact with a healthcare provider that may indicate a MAAE.

A positive response by the participant to these prompts will result in a notification to both the participant and study staff to arrange a call. A follow-up safety call will be performed to the participant to determine if an unscheduled Illness Visit for the participant should be arranged as defined in Section 8.1.2. The results of the safety call should be recorded in the appropriate source documentation.

If a participant does not respond to the weekly eDiary within a 2-day window around the scheduled timepoint, study staff will follow-up directly with the participant via phone call or text to confirm their health status and to remind the participant of the importance of maintaining weekly contact via the eDiary prompt.

In addition, there will be an eDiary prompt to solicit the collection of information regarding participant's history of facial injections or dermal fillers, for cosmetic or medical indications such as migraine headaches.

8.2.3. Demographics/Medical History

Demographic information relating to the participant's sex, age, and race will be recorded at Screening on the appropriate eCRF page.

Additionally, information regarding participant occupational circumstances (eg, essential worker status) will be collected at Screening.

Medical history of each participant, including risk factors for severe COVID-19 as defined in Section 6.2.1.1, will be collected and recorded on the Medical History eCRF page. Significant findings that were present prior to the signature of the informed consent will also be included in the Medical History eCRF page.

Study participants will also be asked to report history of receipt of seasonal influenza vaccine during the current influenza season (typically October through April in the Northern Hemisphere) as a concomitant medication.

8.2.4. Physical Examination

A full physical examination, including vital signs, height, and weight, will be performed at Screening and on Day 1, and symptom-directed physical examinations at other scheduled time points as indicated in the SoEs (Section 11.1). The full examination will include assessment of skin, head, ears, eyes, nose, throat, neck, thyroid, lungs, heart, cardiovascular, abdomen, lymph nodes, and musculoskeletal system/extremities. Symptom directed- physical examinations may be performed at other timepoints at the discretion of the investigator.

On each dosing day before injection, the arm receiving the injection should be examined and the associated lymph nodes should be evaluated.

Body mass index will be calculated at the Screening Visit (Day 0).

Any clinically significant finding identified during a study visit after the first dose should be reported as a MAAE.

Significant new findings that begin or worsen after informed consent must be recorded on the AE eCRF page.

8.2.5. Vital Sign Measurements

Vital signs will be measured at the time points indicated in the SoEs (Section 11.1). The participant will be seated for at least 5 minutes before all measurements are taken. On Day 1 and Day 29, vital sign measurements will be collected once before IP injection and at least 30 minutes after IP injection (before participants are discharged from the study site). When applicable, vital sign measurements should be performed before blood collection.

Febrile participants at Day 1 and Day 29 visits (fever is defined as a body temperature ≥ 38.0°C/100.4°F) may be rescheduled within the relevant window periods. Criteria for delay of study treatment are provided in Section 7.1. Afebrile participants with minor illnesses may be injected at the discretion of the investigator.

If any of the vital sign measurements meet the toxicity grading criteria for clinical abnormalities of Grade 3 or greater, the abnormal value and Grade will be documented on the AE page of the eCRF (unless there is another known cause of the abnormality that would result in an AE classification). The investigator will continue to monitor the participant with additional assessments until the vital sign value has reached the reference range, returns to the vital sign value at baseline, is considered stable, or until the investigator determines that follow-up is no longer medically necessary.

8.2.6. Blood Sampling Volumes

The maximum planned volumes of blood sampled per participant are approximately 50 mL for 1 day, 100 mL for 28 days, and 300 to 340 mL for the complete study (Table 4).

Table 4: Maximum Planned Blood Sampling Volumes per Participant by Visit

Study Visit Day	Part A D1	Part A D29	Part A D57	Part A D209	Part A D394	Part A D759	Part A Total
Immunogenicity blood samples [a,b]	50 mL	50 mL	50 mL	50 mL	50 mL	50 mL	300 mL
	**Open-Label Day 1**	**Open-Label Day 29[b]**	**Open-Label Day 57[b]**	NA	NA	NA	NA
	20 mL	20 mL	20 mL	NA	NA	NA	40 mL

Abbreviations: D = Day; NA = not applicable; RT-PCR = reverse transcriptase polymerase chain reaction.

[a] If a Part A and Part B (Open-Label) visit overlap, only the Part B sample will be taken.

[b] For participants who received placebo during the Blinded Phase and receive mRNA-1273 during the Open-Label Observational Phase (Table 21). Note that immunogenicity blood samples are collected only on Open-Label Days 1 and 29 or Day 57 during the Open-Label Observational Phase.

[c] Additional blood samples (16 mL) will be taken at Illness Day 1 and Convalescent Visit Day 28 for those participants who report symptoms of COVID-19 and who have RT-PCR confirmed symptomatic COVID-19, respectively.

8.3. Safety Definitions and Procedures

8.3.1. Adverse Event

An AE is defined as any untoward medical occurrence associated with the use of a drug in humans, whether or not considered drug related.

A TEAE is defined as any event not present before exposure to IP or any event already present that worsens in intensity or frequency after exposure.

Events Meeting the Adverse Event Definition

- Exacerbation of a chronic or intermittent pre-existing condition including either an increase in frequency and/or intensity of the condition.
- New conditions detected or diagnosed after the first dose of IP even though they may have been present before the start of the study.

Events NOT Meeting the Adverse Event Definition

- Medical or surgical procedure (eg, endoscopy, appendectomy): the condition that leads to the procedure should be the AE.
- Situations in which an untoward medical occurrence did not occur (social and/or convenience admission to a hospital).

An AR is any AE for which there is a reasonable possibility that the IP caused the AE (Section 8.3.4). For the purposes of investigational new drug safety reporting, "reasonable possibility" means that there is evidence to suggest a causal relationship between the IP and the AE.

An unsolicited AE is any AE reported by the participant that is not specified as a solicited AR in the protocol; or is specified as a solicited AR in the protocol, but starts outside the protocol-defined period for reporting solicited ARs (ie, for the 7 days after each dose of IP, Part A, Blinded Phase only).

8.3.2. Medically Attended Adverse Events

An MAAE is an AE that leads to an unscheduled visit (including a telemedicine visit) to a healthcare practitioner (HCP). This would include visits to a study site for unscheduled assessments (eg, rash assessment, abnormal laboratory follow-up, COVID-19 [Section 8.1.1]) and visits to HCPs external to the study site (eg, urgent care, primary care physician). Investigators will review unsolicited AEs for the occurrence of any MAAEs. All MAAEs must be fully reported on the MAAE page of the eCRF.

8.3.2.1. Anaphylaxis

All suspected cases of anaphylaxis should be recorded as MAAEs and reported as an SAE, based on criteria for a medically important event, unless the event meets other serious criteria. As an SAE, the event should be reported to the Sponsor or designee immediately and in all circumstances within 24 hours as per Section 8.3.11 (Reporting SAEs). The investigator will submit any updated anaphylaxis case data to the Sponsor within 24 hours of it being available. For reporting purposes, a participant who displays signs/symptoms consistent with anaphylaxis as shown below should be reported as a potential case of anaphylaxis. This is provided as general guidance for investigators and is based on the Brighton Collaboration case definition (Rüggeberg et al 2007).

Anaphylaxis is an acute hypersensitivity reaction with multi-organ system involvement that can present as, or rapidly progress to, a severe life-threatening reaction. It may occur following exposure to allergens from a variety of sources. Anaphylaxis is a clinical syndrome characterized by:

- Sudden onset AND
- Rapid progression of signs and symptoms AND
- Involving 2 or more organ systems, as follows:
  - **Skin/mucosal**: urticaria (hives), generalized erythema, angioedema, generalized pruritus with skin rash, generalized prickle sensation, red and itchy eyes

- **Cardiovascular**: measured hypotension, clinical diagnosis of uncompensated shock, loss of consciousness or decreased level of consciousness, evidence of reduced peripheral circulation
- **Respiratory**: bilateral wheeze (bronchospasm), difficulty breathing, stridor, upper airway swelling (lip, tongue, throat, uvula, or larynx), respiratory distress, persistent dry cough, hoarse voice, sensation of throat closure, sneezing, rhinorrhea
- **Gastrointestinal**: diarrhea, abdominal pain, nausea, vomiting

8.3.3. Serious Adverse Events

An AE (including an AR) is considered an SAE if, in the view of either the investigator or Sponsor, it results in any of the following outcomes:

- **Death**
  A death that occurs during the study or that comes to the attention of the investigator during the protocol-defined follow-up period must be reported to the Sponsor, whether or not it is considered related to IP.
- **Is life-threatening**
  An AE is considered life-threatening if, in the view of either the investigator or the Sponsor, its occurrence places the participant at immediate risk of death. It does not include an AE that, had it occurred in a more severe form, might have caused death.
- **Inpatient hospitalization or prolongation of existing hospitalization**
  In general, inpatient hospitalization indicates the participant was admitted to the hospital or emergency ward for at least one overnight stay for observation and/or treatment that would not have been appropriate in the physician's office or outpatient setting. The hospital or emergency ward admission should be considered an SAE regardless of whether opinions differ as to the necessity of the admission. Complications that occur during inpatient hospitalization will be recorded as an AE; however, if a complication/AE prolongs hospitalization or otherwise fulfills SAE criteria, the complication/AE will be recorded as a separate SAE.
- **Persistent or significant incapacity or substantial disruption of the ability to conduct normal life functions**
  This definition is not intended to include experiences of relatively minor medical significance such as uncomplicated headache, nausea/vomiting, diarrhea, influenza, and accidental trauma (eg, sprained ankle) which may interfere with or prevent everyday life functions but do not constitute a substantial disruption.
- **Congenital anomaly or birth defect**

- **Medically important event**
  Medical judgment should be exercised in deciding whether SAE reporting is appropriate in other situations, such as important medical events that may not be immediately life-threatening or result in death or hospitalization but may jeopardize the participant or require medical or surgical intervention to prevent one of the other outcomes listed in the above definition. These events should usually be considered serious. Examples of such medical events include allergic bronchospasm requiring intensive treatment in an emergency room or at home, blood dyscrasias or convulsions that do not result in inpatient hospitalization, or the development of drug dependency or drug abuse.

8.3.4. Solicited Adverse Reactions (Part A, Blinded Phase Only)

The term "reactogenicity" refers to the occurrence and intensity of selected signs and symptoms (ARs) occurring after IP injection. The eDiary will solicit daily participant reporting of ARs using a structured checklist (Section 8.2.2). Participants will record such occurrences in an eDiary on the day of each IP injection and for the 6 days after the day of dosing.

Severity grading of reactogenicity will occur automatically based on participant entry into the eDiary according to the grading scales presented in Table 5 modified from the Toxicity Grading Scale for Healthy Adult and Adolescent Volunteers Enrolled in Preventative Vaccine Clinical Trials (DHHS 2007).

If a solicited local or systemic AR continues beyond 7 days after dosing, the participant will be prompted daily to capture solicited local or systemic AR in the eDiary until resolution. Adverse reactions recorded in eDiaries beyond Day 7 should be reviewed by the investigator either via phone call or at the following study visit. All solicited ARs (local and systemic) will be considered causally related to dosing.

Table 5: Solicited Adverse Reactions and Grades

Reaction	Grade 0	Grade 1	Grade 2	Grade 3	Grade 4
Injection site pain	None	Does not interfere with activity	Repeated use of over-the-counter pain reliever > 24 hours or interferes with activity	Any use of prescription pain reliever or prevents daily activity	Requires emergency room visit or hospitalization
Injection site erythema (redness)	< 25 mm/ < 2.5 cm	25 - 50 mm/ 2.5 - 5 cm	51 - 100 mm/ 5.1 - 10 cm	> 100 mm/ > 10 cm	Necrosis or exfoliative dermatitis
Injection site swelling/induration (hardness)	< 25 mm/ < 2.5 cm	25 - 50 mm/ 2.5 - 5 cm	51 - 100 mm/ 5.1 - 10 cm	> 100 mm/ > 10 cm	Necrosis
Axillary (underarm) swelling or tenderness ipsilateral to the side of injection	None	No interference with activity	Repeated use of over-the-counter (non-narcotic) pain reliever > 24 hours or some interference with activity	Any use of prescription (narcotic) pain reliever or prevents daily activity	Emergency room visit or hospitalization
Headache	None	No interference with activity	Repeated use of over-the-counter pain reliever > 24 hours or some interference with activity	Significant; any use of prescription pain reliever or prevents daily activity	Requires emergency room visit or hospitalization
Fatigue	None	No interference with activity	Some interference with activity	Significant; prevents daily activity	Requires emergency room visit or hospitalization
Myalgia (muscle aches all over body)	None	No interference with activity	Some interference with activity	Significant; prevents daily activity	Requires emergency room visit or hospitalization
Arthralgia (joint aches in several joints)	None	No interference with activity	Some interference with activity	Significant; prevents daily activity	Requires emergency room visit or hospitalization

Reaction	Grade 0	Grade 1	Grade 2	Grade 3	Grade 4
Nausea/vomiting	None	No interference with activity or 1-2 episodes/ 24 hours	Some interference with activity or > 2 episodes/24 hours	Prevents daily activity, requires outpatient intravenous hydration	Requires emergency room visit or hospitalization for hypotensive shock
Chills	None	No interference with activity	Some interference with activity not requiring medical intervention	Prevents daily activity and requires medical intervention	Requires emergency room visit or hospitalization
Fever (oral)	< 38.0°C<br>< 100.4°F	38.0 – 38.4°C<br>100.4 – 101.1°F	38.5 – 38.9°C<br>101.2 – 102.0°F	39.0 – 40.0°C<br>102.1 – 104.0°F	> 40.0°C<br>> 104.0°F

Any solicited AR that meets any of the following criteria must be entered into the participant's source document and must also be recorded as an AE in the participant's Adverse Event eCRF:

- Solicited local or systemic AR that results in a visit to an HCP (MAAE)
- Solicited local or systemic AR leading to the participant withdrawing from the study or the participant being withdrawn from the study by the investigator (AE leading to withdrawal)
- Solicited local or systemic AR lasting beyond 7 days post injection
- Solicited local or systemic AR that leads to participant withdrawal from IP
- Solicited local or systemic AR that otherwise meets the definition of an SAE

8.3.5. Recording and Follow-up of Pregnancy

Female participants who have a positive pregnancy test at Screening should not be enrolled; participants who have a positive pregnancy test any time during the study should receive no further dosing with IP but should be asked to remain in the study and be followed-up for safety. Pregnancy testing is scheduled to occur at Screening, Blinded and Open-label Day 1, and Blinded and Open-label Day 29 (Table 16, Table 21, and Table 22).

Details of all pregnancies in female participants will be collected after the start of study treatment and until Day 759.

- If a pregnancy is reported, the investigator should inform the Sponsor within 24 hours of learning of the pregnancy and should follow the procedures outlined in this section.
- Abnormal pregnancy outcomes (eg, spontaneous abortion, fetal death, stillbirth, congenital anomalies, ectopic pregnancy) are considered SAEs.

Pregnancies occurring in participants after enrollment must be reported to Sponsor or designee within 24 hours of the site learning of its occurrence. If the participant agrees to submit this information, the pregnancy must be followed to determine the outcome, including spontaneous or voluntary termination, details of the birth, and the presence or absence of any birth defects, congenital abnormalities, or maternal and/or newborn complications. This follow-up should occur even if intended duration of the safety follow-up for the study has ended. Pregnancy report forms will be distributed to the study site to be used for this purpose. The investigator must immediately (within 24 hours of awareness) report to the Sponsor any pregnancy resulting in an abnormal outcome according to the procedures described for SAEs.

8.3.6. Recording and Follow-up of an AE and/or SAE

The investigator is responsible for ensuring that all AEs and SAEs are recorded in the eCRF and reported to the Sponsor.

Solicited ARs will be collected from Day 1 through 7 days after each dose (Part A: Blinded Phase only). Other (unsolicited) AEs will be collected from Day 1 through 28 days after each dose up to Day 57 (Part A: Blinded Phase only).

Both MAAEs and SAEs will be collected from Blinded and Open-label Day 1 throughout entire study duration (Day 759 for all participants), as specified in the SoEs (Table 16, Table 17, Table 18, Table 19, Table 21, and Table 22). Any AEs occurring before receipt of IP will be analyzed separately from TEAEs.

At every study site visit or telephone contact, participants will be asked a standard question to elicit any medically related changes, including surveillance for COVID-19 symptoms, in their well-being according to the scripts provided. Participants will also be asked if they have been hospitalized, had any accidents, used any new medications, changed concomitant medication regimens (both prescription and over-the-counter medications), or had any non-study vaccinations.

In addition to participant observations, physical examination findings or other documents relevant to participant safety classified as an AE will be documented on the AE page of the eCRF.

After the initial AE/SAE report, the investigator is required to proactively follow each participant at subsequent visits/contacts. All AEs and SAEs will be treated as medically appropriate and followed until resolution, stabilization, the event is otherwise explained, or the participant is LTFU (as defined in Section 7.4).

8.3.7. Time Period and Frequency for Collecting AE and SAE Information

All confirmed serious COVID-19 cases and SAEs will be recorded and reported to the Sponsor or designee immediately and under no circumstance should this exceed 24 hours, as indicated in Section 8.3.10. The investigator will submit any updated serious COVID-19 cases and SAE data to the Sponsor within 24 hours of it being available. COVID-19 cases are defined in Section 8.1.1.

Investigators are not obligated to actively seek AEs or SAEs after conclusion of the study participation (Day 759). However, if an investigator learns of any SAE, including a death, at any time after a participant has been discharged from the study and considers the event to be reasonably related to the study IP or study participation, the investigator must promptly notify the Sponsor.

8.3.8. Assessment of Intensity

An event is defined as "serious" when it meets at least one of the predefined outcomes as described in the definition of an SAE (Section 8.3.3), NOT when it is rated as severe.

The severity (or intensity) of an AR or AE refers to the extent to which it affects the participant's daily activities. The Toxicity Grading Scale for Healthy Adult and Adolescent Volunteers Enrolled in Preventative Vaccine Clinical Trials (DHHS 2007) will be used to categorize local and systemic reactogenicity events (solicited ARs), clinical laboratory test results, and vital sign measurements observed during this study. Specific criteria for local and systemic reactogenicity events are presented in Section 8.3.4.

The determination of severity for all unsolicited AEs should be made by the investigator based upon medical judgment and the definitions of severity as follows:

- Mild: These events do not interfere with the participant's daily activities.
- Moderate: These events cause some interference with the participant's daily activities and require limited or no medical intervention.
- Severe: These events prevent the participant's daily activity and require intensive therapeutic intervention.

Study staff should elicit from the participant the impact of AEs on the participant's activities of daily living to assess severity and document appropriately in the participant's source documentation. Changes in the severity of an AE should be documented in the participant's source documentation to allow an assessment of the duration of the event at each level of intensity to be performed. An AE characterized as intermittent requires documentation of onset and duration of each episode. An AE that fluctuates in severity during the course of the event is reported once in the eCRF at the highest severity observed.

8.3.9. Assessment of Causality

The investigator's assessment of an AE's relationship to IP is part of the documentation process but is not a factor in determining what is or is not reported in the study.

The investigator will assess causality (ie, whether there is a reasonable possibility that the IP caused the event) for all AEs and SAEs. The relationship will be characterized using the following classification:

Not related: There is not a reasonable possibility of a relationship to the IP. Participant did not receive the IP OR temporal sequence of the AE onset relative to administration of the IP is not reasonable OR the AE is more likely explained by another cause than the IP.

Related: There is a reasonable possibility of a relationship to the IP. There is evidence of exposure to the IP. The temporal sequence of the AE onset relative to the administration of the IP is reasonable. The AE is more likely explained by the IP than by another cause.

8.3.10. Reporting Adverse Events

The investigator is responsible for reporting all AEs that are observed or reported during the study, regardless of their relationship to IP or their clinical significance. If there is any doubt as to whether a clinical observation is an AE, the event should be reported.

All unsolicited AEs reported or observed during the study will be recorded on the AE page of the eCRF. Information to be collected includes, type of event, time of onset, investigator -specified assessment of severity (impact on activities of daily living) and relationship to IP, time of resolution of the event, seriousness, as well as any required treatment or evaluations, and outcome. The unsolicited AEs resulting from concurrent illnesses, reactions to concurrent illnesses, reactions to concurrent medications, or progression of disease states must also be reported. All AEs will be followed until they are resolved or stable or judged by the investigator to be not clinically significant. The Medical Dictionary for Regulatory Activities (MedDRA) will be used to code all unsolicited AEs.

Any medical condition that is present at the time that the participant is screened but does not deteriorate should not be reported as an unsolicited AE. However, if it deteriorates at any time during the study, it should be recorded as an unsolicited AE.

Any AE or COVID-19 case considered serious by the investigator or that meets SAE criteria (Section 8.3.3) must be reported to the Sponsor immediately (within 24 hours of becoming aware of the SAE or COVID-19 case). The investigator will assess whether there is a reasonable possibility that the IP caused the SAE. The Sponsor will be responsible for notifying the relevant regulatory authorities of any SAE as outlined in the 21 US CFR Parts 312 and 320. The investigator is responsible for notifying the institutional review board (IRB) directly.

If the eCRF is unavailable at the time of the SAE, the following contact information is to be used for SAE reporting:

- SAE Mailbox: Safety_Moderna@iqvia.com
- SAE Hotline (USA and Canada): +1-866-599-1341
- SAE Fax line (USA and Canada): +1-866-599-1342

8.3.11. Regulatory Reporting Requirements for SAEs

Prompt notification by the investigator to the Sponsor of a SAE is essential so that legal obligations and ethical responsibilities towards the safety of participants and the safety of a study intervention under clinical investigation are met.

The Sponsor has a legal responsibility to notify both the local regulatory authority and other regulatory agencies about the safety of a study intervention under clinical investigation. The Sponsor will comply with country-specific regulatory requirements relating to safety reporting to the regulatory authority, IRBs, and investigators.

Investigator safety reports must be prepared for suspected unexpected serious ARs according to local regulatory requirements and Sponsor policy and forwarded to investigators as necessary.

An investigator who receives an investigator safety report describing a SAE or other specific safety information (eg, summary or listing of SAEs) from the Sponsor will review and then file it along with the IB and will notify the IRB/IEC, if appropriate according to local requirements.

8.4. Monitoring Committees

8.4.1. Protocol Safety Review Team

A Protocol Safety Review Team will be formed to review interim and cumulative blinded safety data on a regular basis with a remit to escalate concerns to the DSMB.

8.4.2. Data and Safety Monitoring Board

An independent DSMB will periodically review blinded and unblinded data, including both safety and cases of COVID-19 at scheduled data review meetings and at 2 planned IAs.

- In addition to blinded and unblinded review of safety data, at each data review meeting the DSMB will review the numbers and rate of COVID-19 cases, including rate of severe COVID disease with prespecified thresholds for imbalance in the treatment groups which would trigger halting rules.
- At the IA, the DSMB will review the IA results and make recommendations to an Oversight Group in terms of study results reporting and unblinding based on the boundaries of early efficacy as described in Section 9.6 of the protocol. The Oversight Group will be comprised of a voting member each from the Sponsor, Biomedical Advance Research and Development Authority (BARDA), and NIAID.
- The DSMB will monitor the study for non-efficacy at the IA. The boundary for non-efficacy is non-binding and will be provided in the DSMB analysis plan.

- The DSMB will also monitor the study for vaccine harm based on severe COVID-19. Continuous harm monitoring will be provided for COVID-19 and severe COVID-19 separately. For harm monitoring, cases will be counted starting after the first dose of study vaccination. Boundaries will be provided based on the exact 1-sided binomial tests conditional on the total number of cases under the assumption of VE=0%. If the prespecified stopping boundary is reached for either COVID-19 or severe COVID-19, then the unblinded statisticians will immediately inform the DSMB that the harm rules have been met. Details will be provided in the DSMB analysis plan.
- The boundaries are considered guidelines, ie, a recommendation to modify the study would not be based solely on statistical rules, as many other factors (ie, totality of the data from the study including additional efficacy, safety and immunogenicity endpoints as well as data external to the study) may be part of the decision process. In the case of a recommendation to continue the trial regardless of crossing the boundaries for efficacy or inefficacy, the reason for disregarding the boundary must be documented in the meeting minutes and communicated to the Sponsor.

After each data review meeting or IA, the DSMB will make a recommendation to the Sponsor through an Oversight Group to take one of the following courses of action:

- Stop further enrollment due to meeting criteria for early efficacy or due to a safety concern.
- Pause enrollment and consider a change in study design.
- Continue enrollment and/or study conduct as planned.

The Sponsor may also request that the DSMB conduct ad hoc reviews of safety events from this study or other data, including new nonclinical or clinical information related to mRNA-1273 external to this study. The DSMB will review all available study data to adjudicate such events in accordance with the DSMB charter.

The DSMB composition, its remit, and frequency of data review will be further defined in the DSMB charter and analysis plan.

8.4.3. Adjudication Committee

An Adjudication Committee (AC) will be assembled for the purpose of reviewing potential cases to determine if the criteria for the primary and secondary endpoints have been met. The AC will remain blinded to treatment assignment. The AC composition, its remit, and frequency of data review will be further defined in a Charter.

8.5. Management of Overdose

As the study treatment is to be administered by a healthcare professional, it is unlikely that an overdose will occur. Dose deviations will be tracked as protocol deviations (Section 11.2.8).

8.6. Pharmacokinetics

Pharmacokinetic parameters are not evaluated in this study.

8.7. Pharmacodynamics

Pharmacodynamic parameters are not evaluated in this study.

8.8. Exploratory Assessments and Biomarkers

Exploratory assessments may include assessment of serologic markers of disease severity, immune response to SARS-CoV-2, RT-PCR of NP swab, or nasal swab, or saliva samples, and genetic sequences of SARS-CoV-2 strains isolated from participants' samples.

8.9. Medical Resource Utilization and Health Economics

Medical resource utilization and health economics parameters are not evaluated in this study.

9. STATISTICAL CONSIDERATIONS

This section summarizes the planned statistical analysis strategy and procedures for the study. The details of statistical analysis will be provided in the SAP, which will be finalized before the clinical database lock for the study and treatment unblinding. If, after the study has begun, but prior to any unblinding, changes are made to primary and/or key secondary objectives/hypotheses, or the statistical methods related to those hypotheses, then the protocol will be amended (consistent with ICH Guideline E9). Changes to other secondary or exploratory analyses made after the protocol has been finalized, along with an explanation as to when and why they occurred, will be listed in the SAP or Clinical Study Report (CSR) for the study. Ad hoc exploratory analyses, if any, will be clearly identified in the CSR. Statistical analysis strategy incorporating the Open-Label Phase will be provided in a SAP.

9.1. Blinding and Responsibility for Analyses

Blinding during the Part A, the Blinded Phase of the study will be conducted as described in Section 6.2.8. The Sponsor Biostatistics department or designee will generate the randomized allocation schedule(s) for study treatment assignment. Randomization will be implemented via an IRT.

Planned interim and primary analyses are described in Section 9.6. Participant-level unblinding will be restricted to an independent unblinded statistician and, as needed, a statistical programmer performing the IAs, who will have no other responsibilities associated with the study.

In addition to the routine study monitoring outlined in this protocol, an external DSMB will review interim data to safeguard the interests of clinical study participants and to enhancing the integrity of the study. The DSMB will review treatment-level results of the IAs, provided by the independent unblinded statistician. Limited additional Sponsor personnel may be unblinded to the treatment-level results of the IAs, if required, in order to act on the recommendations of the DSMB. The extent to which individuals are unblinded with respect to results of IAs will be documented. Depending on the recommendation of the DSMB, the Sponsor may prepare a regulatory submission after an IA. In this case, pre-identified Sponsor members including the analysis and reporting team will be unblinded to treatment assignments and remain unblinded for the remainder of the study. Participants and investigators will remain blinded.

9.2. Statistical Hypotheses

For the primary efficacy objective, the null hypothesis of this study is that the VE of mRNA-1273 to prevent first occurrence of COVID-19 is ≤ 30% (ie, $H_0^{efficacy}$: VE ≤ 0.3). The study will be considered to meet the primary efficacy objective if the corresponding CI of VE rules out 30% at either one of the IAs or at the primary analysis.

Vaccine efficacy is defined as the percent reduction in the hazard of the primary endpoint (mRNA-1273 vs. placebo). Equivalently, the null hypothesis is:

$H_0^{efficacy}$: HR ≥ 0.7 (equivalently, proportional hazards VE ≤ 0.3).

A stratified Cox proportional hazard model will be used to assess the magnitude of the treatment group difference (ie, HR) between mRNA-1273 and placebo at a 1-sided 0.025 significance level.

The primary analysis population for efficacy will be the PP Set, defined in Section 9.4. In the primary analysis of efficacy, cases will be counted starting 14 days after the second dose of IP.

9.3. Sample Size Determination

The sample size is driven by the total number of cases to demonstrate VE (mRNA-1273 vs. placebo) to prevent COVID-19. Under the assumption of proportional hazards over time and with 1:1 randomization of mRNA-1273 and placebo, a total of 151 COVID-19 cases will provide 90% power to detect a 60% reduction in hazard rate (60% VE), rejecting the null hypothesis H_0: VE ≤ 30%, with 2 IAs at 35% and 70% of the target total number of cases using a 1-sided O'Brien-Fleming boundary for efficacy and a log-rank test statistic with a 1-sided false positive error rate of 0.025. The total number of cases pertains to the PP Set accruing at least 14 days after the second dose. There are 2 planned IAs in this study, which will be performed when approximately 35% and 70% of the target total number of cases have been observed. Approximately 30,000 participants will be randomized with the following assumptions:

- The target VE against COVID-19 is 60% (with 95% CI lower bound ruling out 30%, rejecting the null hypothesis H_0: VE ≤ 30%)
- A 6-month COVID-19 incidence rate of 0.75% in the placebo arm
- An annual dropout rate of 2% (loss of evaluable participants)
- Two IAs at 35% and 70% of total target cases across the 2 treatment groups with O'Brien-Fleming boundaries for efficacy monitoring
- 3-month uniform accrual
- Approximately 15% of participants will be excluded from the PP population, and participants are at risk for COVID-19 starting 14 days after the second dose

Table 6 provides sample size with 90% power to demonstrate VE on COVID-19.

Table 6: Conditions and Sample Size to Demonstrate Vaccine Efficacy

Target VE	Lower Bound	Randomization Ratio	Total # of Cases	6-Month Incidence Rate		Total Sample Size[a]
--	--	--	--	Placebo	mRNA-1273	--
60%	30%	1:1	151	0.75%	0.30%	30,000

[a] Sample size to account for 15% participants to be excluded from the PP Set (eg, seropositive at baseline, have not received planned IP).

The sample size is calculated using R package gsDesign (Anderson 2020).

Under these above assumptions including 6-month incidence rate of 0.75% on placebo, with 30,000 participants, it will take approximately 5, 8, and 10 months from study start (first subject first dose), respectively, to accrue 35% (approximately 53), 70% (approximately 106) and 100% (151) of the target number of cases in the PP Set.

Figure 5 shows the power of the primary efficacy endpoint under true VE at the 2 planned IAs and the primary efficacy analysis assuming a total of 151 events.

Figure 5: Boundary Crossing Probabilities by Effect Size

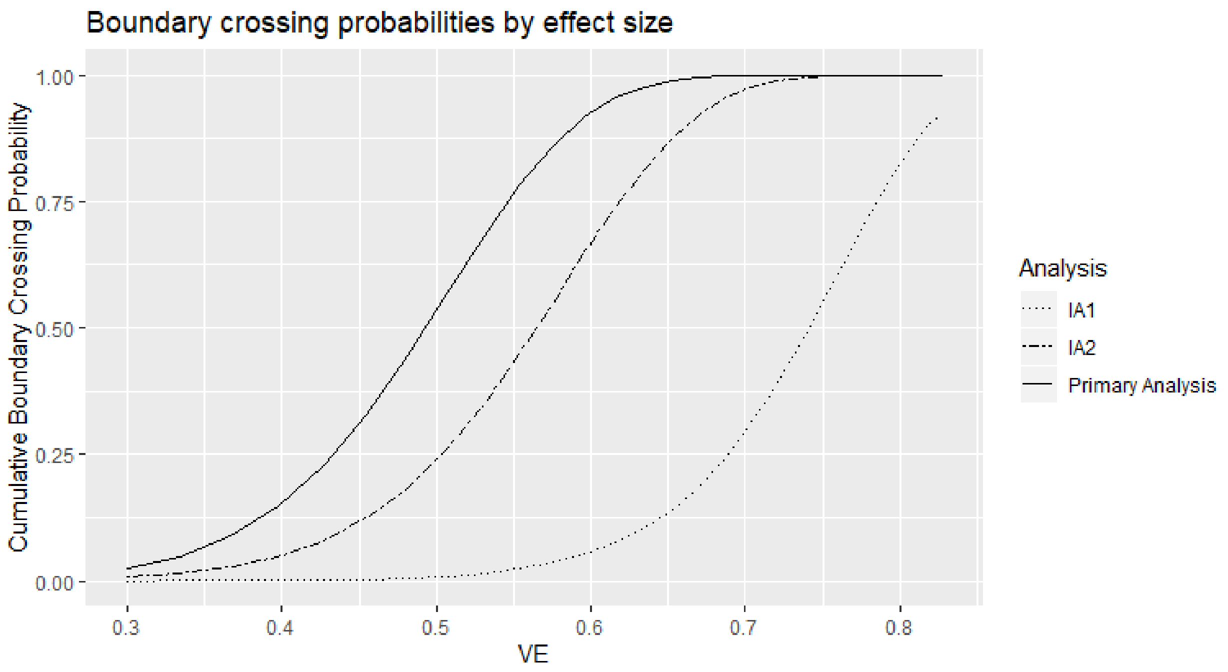

The Sponsor may adjust the size of the study or duration of follow-up based on the blinded review of the total number of cases of COVID-19 accrued during the study, in addition to estimated percentages of study participants with serologic evidence of SARS-CoV-2 infection at baseline.

9.3.1. Power for Selected Secondary Efficacy Endpoints

For the secondary objective on VE against virologically confirmed SARS-CoV-2 infection or COVID-19 regardless of symptomology or severity (COV-INF), the study will have ≥ 90% power to demonstrate the VE is above 30% (to reject null hypothesis VE ≤ 30%) at 1-sided alpha of 2.5% if the true VE to prevent COV-INF is 60% because every COVID-19 endpoint is necessarily a COV-INF endpoint.

For the secondary objective on VE against severe COVID-19, Table 7 provides power of demonstrating VE based on a total of 30 and 60 events under different scenarios of true VE and VE criteria.

Table 7: Power of Demonstrating Vaccine Efficacy Against Severe COVID-19

True VE	Total No. of Severe COVID-19 Cases	Severe COVID-19 (Secondary Objective)	
		To Reject Null Hypothesis	Power
60%	30	VE≤ 0%	70.90%
		VE≤ 10%	60.30%
		VE≤ 20%	47.50%
70%		VE≤ 0%	> 90%
		VE≤ 10%	85.30%
		VE≤ 20%	76.60%
80%		VE≤ 0%	> 90%
		VE≤ 10%	> 90%
		VE≤ 20%	> 90%
60%	60	VE≤ 0%	> 90%
		VE≤ 10%	88.10%
		VE≤ 20%	76.60%
70%		VE≤ 0%	> 90%
		VE≤ 10%	> 90%
		VE≤ 20%	> 90%
80%		VE≤ 0%	> 90%
		VE≤ 10%	> 90%
		VE≤ 20%	> 90%

Abbreviation: VE = vaccine efficacy.

9.4. Analysis Populations

Analysis populations for statistical analyses are Randomization Set, Full Analysis Set (FAS), modified intent-to-treat (mITT) set, PP Set, Immunogenicity Subset, Solicited Safety Set, and Safety Set, as shown in Table 8.

Table 8: Populations for Analyses

Population	Description
Randomization Set	All participants who are randomized, regardless of the participants' treatment status in the study.
Full Analysis Set (FAS)	All randomized participants who received at least one dose of IP.<br>Participants will be analyzed according to the group to which they were randomized.
Modified Intent-to-Treat (mITT) Set	All participants in the FAS who had no immunologic or virologic evidence of prior COVID-19 (ie, negative NP swab test at Day 1, and/or bAb against SARS-CoV-2 nucleocapsid below LOD or LLOQ) at Day 1 before the first dose of IP.<br>Participants will be analyzed according to the group to which they were randomized.
Per-protocol (PP) Set	All participants in the mITT Set who received planned doses of IP per schedule and have no major protocol deviations, as determined and documented by Sponsor prior to database lock and unblinding, that impact critical or key study data.<br>Participants will be analyzed according to the group to which they were randomized.
Immunogenicity Subset	All participants in the FAS who were sampled into a subset for characterizing mRNA-1273 immunogenicity and had a valid immunogenicity test result prior to the first dose of IP and at least 1 valid result after the first dose of IP. Participants in the subset who had major protocol deviations that impact critical or key immunogenicity or study data may be excluded from the Immunogenicity Subset. The details of the Immunogenicity Subset will be documented prior to analysis of immunogenicity data.
Solicited Safety Set	The Solicited Safety Set consists of all randomized participants who received at least one dose of IP and contributed any solicited AR data.<br>The Solicited Safety Set will be used for the analyses of solicited ARs and participants will be included in the treatment group corresponding to the IP that they actually received.
Safety Set	All randomized participants who received at least one dose of IP. The Safety Set will be used for all analyses of safety except for the solicited ARs.<br>Participants will be included in the treatment group corresponding to the IP that they actually received.

9.5. Statistical Analyses

This section provides a summary of the planned statistical analyses of the primary and secondary endpoints.

The overall Type I error rate for the primary endpoint at the IAs and the primary analysis is strictly controlled at 2.5% (1-sided) based on the Lan-DeMets O'Brien-Fleming approximation spending function (see Section 9.6 for details). The primary efficacy results that will be considered statistically significant after consideration of the strategy for controlling the Type I error as described in Section 9.6. Statistical significance of the primary efficacy endpoint can be achieved at either one of the IAs or at the primary analysis. A sequential/hierarchical testing procedure will be used to control type 1 error rate over the primary efficacy endpoint and the secondary efficacy endpoints. Secondary efficacy endpoints will only be tested when the primary efficacy endpoint achieves statistical significance. Multiplicity adjustments among the secondary efficacy endpoints may be performed for secondary efficacy endpoints, in that case, will be specified in the SAP.

No formal multiple comparison adjustments will be employed for multiple safety endpoints or multiple efficacy endpoints. Nominal p-values and confidence intervals (CIs) may be computed for other efficacy analyses without controlling for multiplicity as a measure of VE.

9.5.1. Efficacy Analyses

Efficacy analyses will be performed using the FAS, mITT and PP populations, and participants will be included in the treatment group to which they are randomized. The primary analysis population will be the PP Set.

Table 9 summarizes the analysis approach for primary and secondary efficacy endpoints. Sensitivity analysis methods are described for each endpoint as applicable.

Table 9: Statistical Analysis Methods of Efficacy Endpoints

Endpoint	Statistical Analysis Methods
**Primary endpoint:**<br>Vaccine Efficacy (VE) of mRNA-1273 to prevent COVID-19	• Primary analysis: VE will be estimated with 1 - HR (mRNA-1273 vs placebo) using a Cox proportional hazard regression model with treatment group as a fixed effect and adjust for stratification factor based on the PP Set, with cases counted starting 14 days after the second dose of IP.<br>• Analysis using the same model based on the mITT Set.<br>• Sensitivity analysis using the same model based on the PP Set, with cases counted starting either immediately after the second dose of IP or immediately after the first dose of IP.

Endpoint	Statistical Analysis Methods
	• Subgroup analysis of the primary efficacy endpoint will be performed to assess consistency of VE, such as in the age groups ≥ 18 and < 65 years and ≥ 65 years.<br>• Supportive analysis of VE to be estimated with 1 - ratio of incidence rates with 95% CI using the exact method conditional upon the total number of cases.<br>• Supportive analysis of cumulative incidence VE .
**Secondary endpoints:**<br>• Vaccine efficacy of mRNA-1273 to prevent severe COVID-19<br>• Vaccine efficacy of mRNA-1273 to prevent serologically confirmed SARS-CoV-2 infection or COVID-19 regardless of symptomatology or severity<br>• Vaccine efficacy of mRNA-1273 to prevent COVID-19 using a secondary definition of symptoms<br>• Vaccine efficacy of mRNA-1273 to prevent death due to COVID-19<br>• Vaccine efficacy of mRNA-1273 to prevent COVID-19 after the first dose of IP<br>• Vaccine efficacy of mRNA-1273 to prevent asymptomatic SARS-CoV-2 infection	Similar analysis method as for the primary endpoint analysis.<br>For each of the secondary endpoints:<br>• Primary analysis: VE will be estimated with 1 - HR (mRNA-1273 vs placebo) using a Cox proportional hazard regression model with treatment group as a fixed effect and adjusting for stratification factor based on the PP Set, with cases counted starting 14 days after the second dose of IP.<br>• Analysis using the same model based on the mITT Set.<br>• Sensitivity analyses with cases counted starting immediately after the second dose of IP, 14 days after the first dose of IP, immediately after the first dose of IP, and immediately after randomization.<br>• Vaccine efficacy and 95% CI based on the case incidence will be estimated with 1 - ratio of incidence rates using the exact method conditional upon the total number of cases.
• Vaccine efficacy of mRNA-1273 to prevent COVID-19 in all study participants, regardless of evidence of prior SARS-CoV-2 infection	The FAS population will be used for this secondary objective, using similar analysis methods as for the primary endpoint analysis.<br>• Primary analysis: VE will be estimated with 1 - HR (mRNA-1273 vs placebo) using a Cox proportional hazard regression model with treatment group as a fixed effect and adjusting for stratification factor based on the FAS, with cases counted starting 14 days after the second dose of IP.<br>• Sensitivity analyses with cases counted starting immediately after the second dose of IP, 14 days after the first dose of IP, immediately after the first dose of IP, and immediately after randomization.

9.5.1.1. Efficacy Analysis on Primary Endpoint

To assess the primary efficacy endpoint of VE of mRNA-1273 in preventing the first occurrence of COVID-19 from 14 days after second dose of IP, Cox proportional hazards regression will be used to estimate proportional hazards VE (PH VE), measured by one minus the HR (mRNA-1273 vs. placebo), with a 2-sided score-based 95% CI and 2-sided p-value for testing H_0: VE ≤ 30%.

Vaccine efficacy is defined as the percent reduction in the hazard of the primary endpoint (mRNA-1273 vs placebo). The VE will be estimated using one minus the HR (mRNA-1273 vs placebo) estimand. A stratified Cox proportional hazard model will be used to assess the magnitude of the treatment group difference (ie, HR) between mRNA-1273 and placebo. The HR and its 95% CI from the stratified Cox model with Efron's method of tie handling and with treatment group as covariate will be reported. The same stratification factors used for randomization will be applied to the stratified Cox model.

For the primary efficacy endpoints, participants without documented COVID-19 will be censored at the last study assessment date. Potential intercurrent events may include: 1) death unrelated to COVID-19 and 2) early COVID-19 up to 14 days after second study dose.

In the estimand of the primary analysis on the primary endpoint, a hypothetical strategy will be used for death unrelated to COVID-19; a hypothetical strategy will also be used for early COVID-19, where the time to COVID-19 will be censored at the onset day of early case. The details of intercurrent event description and estimand strategies are presented in Section 11.4.1.

For the primary efficacy analysis, cases will be counted starting 14 days after the second vaccination. Sensitivity analyses with cases counted immediately after the second vaccination, and after randomization will also be carried out.

Analyses of the primary endpoint will be also performed based on the mITT Set using the same methods described above.

For the primary efficacy analysis, cases will be counted starting 14 days after the second dose of IP. Sensitivity analyses with cases counted starting immediately after the second dose of IP and starting immediately after randomization will also be carried out.

Subgroup analysis of the primary efficacy endpoint will be performed in selected subgroups, such as age groups ≥18 and <65 year and ≥65 years to assess consistency of VE as described in Section 9.5.5.

As a supportive analysis, VE will also be estimated by one minus the infection rate ratio, where the number of cases (ie, participants with first occurrence of COVID-19) will be used and the CI will be computed using the exact method conditional upon the total number of cases. Cumulative incidence VE, one minus the ratio of cumulative incidences (mRNA-1273 vs placebo) of COVID--19, may also be assessed, the cumulative incidence for each arm will

be estimated using a covariate adjustment method based on Zeng (2004) that makes use of baseline characteristics.

Additional analysis to evaluate VE against COVID-19 incorporating duration and presence/severity of symptoms will also be performed; the details will be provided in the SAP.

9.5.1.2. Efficacy Analysis on Secondary Endpoint

For each of the below secondary objectives:

- Vaccine efficacy to prevent severe COVID-19
- Vaccine efficacy to prevent serologically confirmed SARS-CoV-2 infection or COVID-19 regardless of symptomology or severity
- Vaccine efficacy to prevent COVID-19 using a broad definition of symptoms
- Vaccine efficacy to prevent death caused by COVID-19
- Vaccine efficacy to prevent asymptomatic SARS-CoV-2 infection

For each of the above secondary objectives, the same Cox proportional hazard model described above for the primary objective will be applied using the PP Set, with cases counted starting 14 days after the second dose of IP. Sensitivity analyses with cases counted starting after the second dose of IP, 14 days after the first dose of IP, after the first dose of IP, and after randomization will also be performed.

The same model will be applied using the mITT population with cases counted starting 14 days after the second dose of IP.

Vaccine efficacy will be estimated with 1- ratio of incidence rates with the 95% CI using the exact method conditional upon the total number of cases.

<u>Vaccine efficacy to prevent COVID-19 after the first dose of IP</u>

The same Cox proportional hazard model described above for the primary objective will be applied using the PP Set, with cases counted starting 14 days after the first dose of IP.

<u>Vaccine efficacy to prevent COVID-19 regardless of prior SARS-CoV-2 infection</u>

The FAS will be used for analysis to evaluate VE to prevent COVID-19 regardless of prior SARS-CoV-2 infection. The same methods described above for the primary objective will be applied with cases counted starting 14 days after the second dose of IP. Sensitivity analyses with cases counted starting after the second dose of IP, 14 days after the first dose of IP, the first dose of IP, and randomization will also be performed.

Vaccine efficacy will be estimated with 1- ratio of incidence rates with the 95% CI using the exact method conditional upon the total number of cases.

9.5.1.3. Long-term Efficacy Analysis

Long-term efficacy will be evaluated after the primary analysis by including data collected in the Open-Label Observational Phase. Long-term efficacy data will be summarized descriptively by treatment cohort described in Table 10 without cohort comparison. The long-term efficacy endpoints are listed in Table 11 with the same case definitions specified in Table 1. In the primary approach, cases will be counted starting 14 days after the second dose of IP for participants in treatment cohorts of mRNA-1273 and Placebo, or starting 14 days after the second dose of mRNA-1273 for participants in the Placebo-mRNA-1273 Cohort. Sensitivity analyses with cases starting from the second dose, 14 days after the first dose, or first dose of IP for participants in the cohorts of mRNA-1273 and placebo, or mRNA-1273 for participants in the Placebo-mRNA-1273 Cohort will be provided. Incidences of cases assessed by numbers, rates, and 2-sided 95% CI based on the exact method adjusting for person-time will be summarized by treatment cohort. The Kaplan-Meier analysis will be used to estimate cumulative incidences of time to first cases by treatment cohort. Long-term efficacy analysis will be performed using FAS for the case of COVID-19 regardless of prior SARS-CoV-2 infection, and long-term efficacy analysis of other cases will be conducted using the PP Set and mITT Set.

In addition, efficacy analyses based on data collected in the Open-Label Observational Phase only will be provided in the SAP.

Table 10: Treatment Cohorts for the Long-term Efficacy Analyses

Treatment Cohort	Description
**mRNA-1273**	Participants randomized to mRNA-1273 in the Blinded Phase.
**Placebo**	Participants randomized to Placebo in the Blinded Phase and did not crossover to mRNA-1273 in the Open-Label Observational Phase.
**Placebo-mRNA-1273**	Participants randomized to Placebo in the Blinded phase and crossed over to mRNA-1273 in the Open-Label Observational Phase.

Table 11: Long-term Efficacy Endpoints

Long-term Efficacy Endpoint
Cases of COVID-19 starting 14-days after the second injection of:<br>• IP for participants in the mRNA-1273 Cohort and the Placebo Cohort,<br>or<br>• mRNA-1273 for participants in the Placebo-mRNA-1273 Cohort.

**Long-term Efficacy Endpoint**
Cases of severe COVID-19 starting 14-days after the second injection of:<br>• IP for participants in the mRNA-1273 Cohort and the Placebo Cohort,<br>or<br>• mRNA-1273 for participants in the Placebo-mRNA-1273 Cohort.
Cases of either COVID-19 or SARS-CoV-2 infection starting 14-days after the second injection of:<br>• IP for participants in the mRNA-1273 Cohort and the Placebo Cohort,<br>or<br>• mRNA-1273 for participants in the Placebo-mRNA-1273 Cohort.
Cases with a secondary (less restrictive) definition of COVID-19 starting 14-days after the second injection of:<br>• IP for participants in the mRNA-1273 Cohort and the Placebo Cohort,<br>or<br>• mRNA-1273 for participants in the Placebo-mRNA-1273 Cohort.
Cases of death due to a cause directly attributed to a complication of COVID-19, starting 14-days after the second injection of:<br>• IP for participants in the mRNA-1273 Cohort and Placebo Cohort,<br>or<br>• mRNA-1273 for participants in the Placebo-mRNA-1273 Cohort.
Cases of COVID-19 starting 14-days after the first injection of:<br>• IP for participants in the mRNA-1273 Cohort and the Placebo Cohort,<br>or<br>• mRNA-1273 for participants in the Placebo-mRNA-1273 Cohort.
Regardless of evidence of prior SARS-CoV-2 infection determined by serologic titer against SARS-CoV-2 nucleocapsid, cases of COVID-19 starting 14-days after the second injection of:<br>• IP for participants in the mRNA-1273 Cohort and the Placebo Cohort,<br>or<br>• mRNA-1273 for participants in the Placebo-mRNA-1273 Cohort.
Cases of SARS-CoV-2 infection in the absence of symptoms defining COVID-19 starting 14-days after the second injection of:<br>• IP for participants in the mRNA-1273 Cohort and the Placebo Cohort,<br>or<br>• mRNA-1273 for participants in the Placebo-mRNA-1273 Cohort.

9.5.2. Safety Analyses

All safety analyses will be based on the Safety Set, except summaries of solicited ARs, which will be based on the Solicited Safety Set. All safety analyses will be provided by treatment group, and by treatment cohort as applicable, unless otherwise specified. Pregnancies and their known outcomes will be summarized (Section 8.3.5).

9.5.2.1. Adverse Events

Safety and reactogenicity will be assessed by clinical review of all relevant parameters.

The number and percentage of participants with any solicited local AR, with any solicited systemic AR, and with any solicited AR during the 7-day follow-up period after each dose (Blinded Phase only) will be provided. A 2-sided 95% exact CI using the Clopper-Pearson method will be also provided for the percentage of participants with any solicited AR for each treatment group.

Number and percentage of participants with unsolicited AEs, SAEs, MAAEs, Grade 3 or higher ARs and AEs, and AEs leading to discontinuation from IP or withdrawal from the study will be summarized. Unsolicited AE will be presented by MedDRA preferred term and system organ class.

Number of events of solicited ARs, unsolicited AEs/SAEs, and MAAEs will be reported in summarization tables accordingly.

For all other safety parameters, descriptive summary statistics will be provided, and Table 12 summarizes analysis strategy for safety parameters. Further details will be described in the SAP.

Table 12: Analysis Strategy for Safety Parameters

Safety Endpoint	Number and Percentage of Participants, Number of Events	95% CI
Any Solicited AR (overall and by local, systemic)	X	X
Any Unsolicited AE	X	
Any SAE	X	
Any Unsolicited MAAE	X	
Any Unsolicited Treatment-Related AE	X	
Any Treatment-Related SAE	X	
Discontinuation due to AE	X	
Any Grade 3 and above AE	X	
Any Treatment-Related Grade 3 and above AE	X	

Notes: 95% CI using the Clopper-Pearson method, X = results will be provided. Unsolicited AEs will be summarized by System Organ Class and Preferred Term coded by MedDRA.

9.5.2.2. Baseline Descriptive Statistics

Demographic variables and baseline characteristics will be summarized by treatment group, and by treatment cohort as applicable, by descriptive statistics (mean, standard deviation for continuous variable, and number and percentage for categorical variables).

9.5.3. Immunogenicity Analyses

The secondary immunogenicity endpoints will be analyzed using the Immunogenicity Subset by treatment group, by treatment cohort as applicable, and by baseline SARS-CoV-2 serostatus, unless otherwise specified. Details for immunogenicity analyses on data collected in the Open-Label Observational Phase will be provided in the SAP.

The SAP will describe the complete set of immunogenicity analyses, including the approach to sample individuals into an Immunogenicity Subset for characterizing mRNA-1273 immunogenicity and assessing immunological correlates of risk and protection.

Data from quantitative immunogenicity assays will be summarized for each treatment group using positive response rates and geometric means with 95% CIs, for each timepoint for which an assessment is performed. Data from qualitative (ie, yielding a positive or negative result) assays will be summarized by tabulating the frequency of positive responses for each assay by group at each timepoint that an assessment is performed. Analyses will focus on the 2 key immunogenicity time points and the change in marker response between them: Day 1 before the first dose of IP and Day 57 (28 days after the second dose of IP). The SAP will describe the complete set of immunogenicity analyses.

Quantitative levels or GMT of specific bAb with corresponding 95% CI at each timepoint and GMFR of specific bAb with corresponding 95% CI at each post-baseline timepoint over pre-dose baseline at Day 1 will be provided by study arm. Descriptive summary statistics including median, minimum, and maximum will also be provided.

GMT of specific nAb with corresponding 95% CI at each timepoint and GMFR of specific nAb with corresponding 95% CI at each post-baseline timepoint over pre-dose baseline at Day 1 will be provided by study arm. Descriptive summary statistics including median, minimum, and maximum will also be provided. For summarizations of group values, antibody values reported as below the LLOQ will be replaced by 0.5 × LLOQ. Values that are greater than the ULOQ will be converted to the ULOQ.

The number and percentage of participants with a fold rise ≥ 2, ≥ 3, and ≥ 4 of serum SARS-CoV-2-specific nAb titers and participants with seroconversion due to vaccination from baseline will be provided with 2-sided 95% CI using the Clopper-Pearson method at each post-baseline timepoint.

Seroconversion due to vaccination at a participant level may be defined as a change from below the LLOQ to equal or above LLOQ, or at least 4-fold rise in terms of neutralizing antibody or vaccine antigen-specific bAb in participants with pre-existing bAb or nAb. The definition of seroconversion will be finalized and documented in the SAP prior to analyzing the immunogenicity data.

The GMT of specific nAb for each group and the geometric mean ratio (GMR) of mRNA-1273 versus placebo with corresponding 2-sided 95% CI will be estimated at each study timepoint using an analysis of covariance (ANCOVA) model with the treatment group and baseline values, if applicable, as explanatory variables, the analysis may adjust for the stratification factor (Table 13).

Table 13: Immunogenicity Endpoints and Statistical Methods

Endpoint	Statistical Analysis Methods
Specific bAb and nAb titers/values	• GMT of each group, GMR (mRNA-1273 vs. Placebo)<br>• GMT estimated by the ANCOVA model
Fold rise	• GMFR - descriptive statistics<br>• Binomial endpoints of fold rise ≥2, 3, and 4, and seroconversion due to vaccination - the Clopper-Pearson method

9.5.4. Exploratory Analyses

The endpoint of viral infection kinetics will be assessed by determining the number of days until testing of nasal swab or saliva samples becomes negative after COVID-19 is established.

The endpoint of duration of symptoms will be assessed by determining the total number of days that a study participant with COVID-19 remains symptomatic through daily assessments after diagnosis.

Vaccine efficacy to prevent all-cause mortality will be assessed by similar analysis methods as used for the primary endpoint analysis, using PP Set, mITT Set, and FAS. All deaths, regardless of cause from the time of randomization, will be included. If the number of deaths becomes large enough to warrant analysis, the same Cox proportional hazard model described above for the primary objective will be applied using the PP Set, the mITT Set, and the FAS. Death, regardless of cause, from randomization will be included.

This endpoint of BOD is defined based on the post SARS-CoV-2 infection follow-up. A BOD score will be used to reflect the severity of symptoms with maximum score at COVID-19 death (Table 14). All other analyses of exploratory endpoints will be described in the SAP before database lock.

Table 14: Burden of Disease Score

Patient State	BOD Score
Uninfected/Asymptomatic infection	0
Symptomatic without hospitalization	1
Hospitalization	2
Death	3

9.5.5. Subgroup Analyses

To determine whether the VE is consistent across various subgroups, the VE and its 95% CI may be estimated using the similar model within each category of the following classification variables.

- Age groups: ≥ 18, < 65, and ≥ 65
- Age and health risk for severe disease: ≥ 18 and < 65 and not at risk; ≥ 18 and < 65 and at risk, and ≥ 65
- Sex (female, male)
- Race
- At risk for severe COVID-19 illness (Section 6.2.1.1)

Subgroup analysis for the long-term efficacy may be performed descriptively by treatment cohort within above categories.

9.6. Interim Analyses

There are 2 planned IAs at 35% and 70% of total target cases across the 2 treatment groups. The primary objective of the IAs is for early detection of reliable evidence that VE is above 30%. The Lan-DeMets O'Brien-Fleming approximation spending function is used for calculating efficacy bounds and to preserve the (1-sided) 0.025 false positive error rate over the IAs and the primary analysis (when the target number of cases have been observed), relative to the hypothesis:

$H_0^{efficacy}$: HR ≥ 0.7 (equivalently, proportional hazards VE ≤ 0.3).

There is no intention to stop the study early if the efficacy has been demonstrated at any of the IAs. If efficacy is demonstrated at an IA, the subsequent IA or primary analysis will be considered supportive in nature. The DSMB will review the IA results and make recommendations to the Sponsor in terms of study results reporting and unblinding based on the boundaries of early efficacy as described in this section, safety data, and data external to this study. In addition to possible early efficacy at IAs, the DSMB will monitor for non-efficacy and vaccine harm; the guiding principles (non-binding) is provided in Section 8.4.2, and the details will be provided in the SAP.

Table 15 summarizes the timing, number of cases and decision guidance at each IA and primary analysis.

The first IA will occur when approximately 35% of the total cases have been observed (across both treatment groups). The study will be considered positive at the first IA if the p-value for rejecting HR ≥ 0.7 is less than 0.0002 based on the Lan-DeMets O'Brien-Fleming approximation spending function. This corresponds to an observed HR of approximately 0.259, or an observed VE approximately 0.741.

The second IA will occur when approximately 70% of the total cases have been observed. The study will be considered positive (VE has been demonstrate) if the p-value for rejecting HR ≥ 0.7 is less than 0.0073 based on the Lan-DeMets O'Brien-Fleming approximation spending function. This corresponds to an observed HR of approximately 0.435, or an observed VE of approximately 0.565.

The primary analysis will be performed when approximately 151 cases have been observed in the study. The study will be considered positive at the primary analysis if the 1-sided p-value for rejecting HR ≥ 0.7 is less than 0.0227. This corresponds to an observed HR of approximately 0.505 or observed VE of approximately 0.495.

Table 15: Interim Boundaries Using O'Brien-Fleming Spending Function, Calculation Based on the PP Set for the Primary Efficacy Endpoint

Information Fraction (% of total #cases)	Number of Cases	Nominal Alpha	Efficacy Boundary Rejecting H0: VE ≤ 30%	Cum Prob (crossing efficacy boundary if the true VE = 60%)
IA1 35%	53	0.0002	VE ≥ 0.741 (HR ≤ 0.259)	4.6%
IA2 70%	106	0.0073	VE ≥ 0.565 (HR ≤ 0.435)	61.5%
Primary analysis 100%	151	0.0227	VE ≥ 0.495 (HR ≤ 0.505)	90.0%

Abbreviations: HR = hazard ratio; IA: interim analysis; LB = lower boundary; PP = per-protocol; VE = vaccine efficacy.

10. REFERENCES

Anderson K. gsDesign, version 3.1.1. 2020. Available from The Comprehensive R Archive Network at https://cran.r-project.org/web/packages/gsDesign/.

Centers for Disease Control and Prevention (CDC). 2020a. Coronavirus Disease 2019 (COVID-19). Retrieved from: https://www.cdc.gov/coronavirus/2019-ncov/cases-updates/cases-in-us.html. (accessed 2020 May 29).

Centers for Disease Control and Prevention (CDC). 2020b. Coronavirus Disease 2019 (COVID-19). Retrieved from: https://www.cdc.gov/coronavirus/2019-ncov/need-extra-precautions/people-at-higher-risk.html (accessed 2020 May 29).

Centers for Disease Control and Prevention (CDC). 2020c. Coronavirus Disease 2019 (COVID-19). Retrieved from: https://www.cdc.gov/coronavirus/2019-ncov/symptoms-testing/symptoms.html (accessed 2020 Jun 11).

Centers for Disease Control and Prevention (CDC). 2020d. Coronavirus Disease 2019 (COVID-19). Retrieved from: https://www.cdc.gov/coronavirus/2019-ncov/need-extra-precautions/people-with-medical-conditions.html. (accessed 2020 July 28).

Chen Y, Lu S, Jia H, Deng Y, Zhou J, Huang B, et al. A novel neutralizing monoclonal antibody targeting the N-terminal domain of the MERS-CoV spike protein. Emerg Microbes Infect. 2017 May 24;6(5):e37.

Chin J, Magoffin RL, Shearer LA, Schieble JH, Lennette EH. Field evaluation of a respiratory syncytial virus vaccine and a trivalent parainfluenza virus vaccine in a pediatric population. Am J Epidemiol 1969;89:449–63

ClinicalTrials.gov [Internet]. Bethesda (MD): National Library of Medicine (US). - Identifier NCT03076385, Safety, tolerability, and immunogenicity of VAL-506440 in healthy adult subjects. Retrieved from: https://clinicaltrials.gov/ct2/keydates/NCT03076385. (accessed 2020 Jun 01).

ClinicalTrials.gov [Internet]. Bethesda (MD): National Library of Medicine (US). - Identifier NCT03325075, Safety, tolerability, and immunogenicity of VAL-181388 in healthy subjects. Retrieved from: https://clinicaltrials.gov/ct2/keydates/NCT03325075. (accessed 2020 Jun 01).

ClinicalTrials.gov [Internet]. Bethesda (MD): National Library of Medicine (US). - Identifier NCT03345043, Safety, tolerability, and immunogenicity of VAL-339851 in healthy adult subjects. Retrieved from: https://clinicaltrials.gov/ct2/keydates/NCT03345043. (accessed 2020 Jun 01).

ClinicalTrials.gov [Internet]. Bethesda (MD): National Library of Medicine (US). - Identifier NCT03382405, Safety, reactogenicity, and immunogenicity of cytomegalovirus vaccines mRNA-1647 and mRNA-1443 in healthy adults. Retrieved from: https://clinicaltrials.gov/ct2/keydates/NCT03382405. (accessed 2020 Jun 01).

ClinicalTrials.gov [Internet]. Bethesda (MD): National Library of Medicine (US). - Identifier NCT03392389, Safety, reactogenicity, and immunogenicity of mRNA-1653 in healthy adults. Retrieved from: https://clinicaltrials.gov/ct2/keydates/NCT03392389. (accessed 2020 Jun 01).

ClinicalTrials.gov [Internet]. Bethesda (MD): National Library of Medicine (US). - Identifier NCT04283461, Safety and immunogenicity study of SARS-CoV-2 vaccine (mRNA-1273) to treat novel coronavirus; 2020 Feb 22. Retrieved from: https://clinicaltrials.gov/ct2/keydates/NCT04283461. (accessed 2020 Jun 01).

See draft guidance for industry Enhancing the Diversity of Clinical Trial Populations - Eligibility Criteria, Enrollment Practices, and Trial Designs Guidance for Industry (June 2019), available at https://www.fda.gov/media/127712/download.

Corti D, Zhao J, Pedotti M, Simonelli L, Agnihothram S, Fett C, et al. Prophylactic and postexposure efficacy of a potent human monoclonal antibody against MERS coronavirus. Proc Natl Acad Sci U S A. 2015 Aug 18;112(33):10473-8.

Department of Health and Human Services (DHHS), Food and Drug Administration, Center for Biologics Evaluation and Research (US). Guidance Document. FDA guidance on conduct of clinical trials of medical products during COVID-19 public health emergency. March 2020. Updated on April 16, 2020. [cited 2020 Apr 17]. Available from: https://www.fda.gov/media/136238/download.

Department of Health and Human Services (DHHS), Food and Drug Administration, Center for Biologics Evaluation and Research (US). Guidance for industry: Toxicity grading scale for healthy adult and adolescent volunteers enrolled in preventative vaccine clinical trials. September 2007 [cited 2019 Apr 10] [10 screens]. Available from:

https://www.fda.gov/downloads/BiologicsBloodVaccines/GuidanceComplianceRegulatoryInformation/Guidances/Vaccines/ucm091977.pdf.

Feldman RA, Fuhr R, Smolenov I, Mick Ribeiro A, Panther L, Watson M, et al. mRNA vaccines against H10N8 and H7N9 influenza viruses of pandemic potential are immunogenic and well tolerated in healthy adults in phase 1 randomized clinical trials. Vaccine. 2019 May 31;37(25):3326-3334.

Fulginiti VA, Eller JJ, Downie AW, Kempe CH. Altered reactivity to measles virus. Atypical measles in children previously immunized with inactivated measles virus vaccines. JAMA. 1967 202:1075-80.

Johnson RF, Bagci U, Keith L, Tang X, Mollura DJ, Zeitlin L, et al. 3B11-N, a monoclonal antibody against MERS-CoV, reduces lung pathology in rhesus monkeys following intratracheal inoculation of MERS-CoV Jordan-n3/2012. Virology. 2016 Mar;490:49-58.

Kim Y, Lee H, Park K, Park S, Lim JH, So MK, et al. Selection and characterization of monoclonal antibodies targeting middle east respiratory syndrome coronavirus through a human synthetic fab phage display library panning. Antibodies (Basel). 2019 Jul 31;8(3).

Rüggeberg JU, Gold MS, Bayas JM, Blum MD, Bonhoeffer J, Friedlander S, et al. Anaphylaxis: case definition and guidelines for data collection, analysis, and presentation of immunization safety data. Vaccine. 2007;25(31):5675-84.

Thomas SJ and Yoon IK. A review of Dengvaxia®: development to deployment. Hum Vaccin Immunother. 2019;15(10), 2295-2314.

Wang L, Shi W, Joyce MG, Modjarrad K, Zhang Y, Leung K, et al. Evaluation of candidate vaccine approaches for MERS-CoV. Nat Commun. 2015 Jul 28;6:7712.

Wang L, Shi W, Chappell JD, Joyce MG, Zhang Y, Kanekiyo M, et al. Importance of neutralizing monoclonal antibodies targeting multiple antigenic sites on the middle east respiratory syndrome coronavirus spike glycoprotein to avoid neutralization escape. J Virol. 2018 Apr 27;92(10).

Widjaja I, Wang C, van Haperen R, Gutiérrez-Álvarez J, van Dieren B, Okba NMA, et al. Towards a solution to MERS: protective human monoclonal antibodies targeting different domains and functions of the MERS-coronavirus spike glycoprotein. Emerg Microbes Infect. 2019;8(1):516-30.

World Health Organization (WHO). Dengue vaccine: WHO position paper, September 2018 – recommendations. Vaccine. 2019 Aug;37(35):4848-4849

World Health Organization (WHO). 2020 (Data reported as of 2020, May 28). Coronavirus disease 2019 (COVID-19) Situation Report – 129. Retrieved from: https://www.who.int/docs/default-source/coronaviruse/situation-reports/20200528-covid-19-sitrep-129.pdf?sfvrsn=5b154880_2. (accessed 2020 May 29).

Wrapp D, Wang N, Corbett KS, Goldsmith JA, Hseih CL, Abiona O, et al. Cryo-EM structure of the 2019-nCoV spike in the prefusion conformation. Science. 2020;367:1260-3.

Yu X, Zhang S, Jiang L, Cui Y, Li D, Wang D, et al. Structural basis for the neutralization of MERS-CoV by a human monoclonal antibody MERS-27. Sci Rep. 2015 Aug 18;5:13133.

Zeng D. 2004. Estimating marginal survival function by adjusting for dependent censoring using many covariates. The Annals of Statistics, 32(4), 1533-1555.

Zent O, Arras-Reiter C, Broeker M, Hennig R. Immediate allergic reactions after vaccinations – a post-marketing surveillance review. Eur J Pediatr. 2002;161(1):21-25.

11. SUPPORTING DOCUMENTATION AND OPERATIONAL CONSIDERATIONS

11.1. APPENDIX 1: Schedules of Events

If a participant cannot attend a study site visit (scheduled or unscheduled, with the exception of Screening, Day 1, Day 29, Participant Decision clinic visit, OL-D1, and OL-D29), a home visit is acceptable if performed by appropriately delegated study site staff or a home healthcare service provided by the Sponsor. If neither a participant visit to the study site nor a home visit to the participant is possible (with the exception of Screening, Day 1, Day 29, Participant Decision clinic visit, OL-D1, and OL-D29), a safety phone call should be performed that includes the assessments scheduled for the biweekly safety phone calls (Table 16).

The Supplemental SoE (Table 21) and the Modified Supplemental SoE (Table 22) are intended to occur in addition to the original Schedules of Events, as applicable (Table 16, Table 17, Table 18, and Table 19) and therefore there is a possibility for study visits to overlap. If visits overlap according to respective visit windows, a single visit may be done with the combined study procedures completed once.

Table 16: Schedule of Events (Vaccination Phase, Day 1 – Day 57)

Visit Number	0	1				2				3
Type of Visit	C	C	SC	SC	SC	C	SC	SC	SC	C
Month/Weekly Timepoint		M0				M1				M2
**Study Visit Day**	D0[1] (Screening)	D1 (Baseline)	7 days after D1 D8	14 days after D1 D15	21 days after D1 D22	D29	7 days after D29 D36	14 days after D29 D43	21 days after D29 D50	D57
**Window Allowance (Days)**	-28		+3	+3	+3	-3/+7	+3	+3	+3	-3/+7
**Days Since Most Recent Vaccination**	-	0	7	14	21	28/0	7	14	21	28
ICF, demographics, concomitant medications, medical history	X									
Confirm participant meets inclusion and exclusion criteria	X	X								
Physical examination[2]	X	X				X				X
Pregnancy testing[3]	X	X				X				
Randomization		X								
**Dosing**										
Study injection (including 30-minute post-dosing observation period)		X				X				
**Efficacy Assessment**										
Surveillance for COVID-19/Unscheduled Visit[4]		X	X	X	X	X	X	X	X	X
Nasopharyngeal swab[5]		X				X				
**Immunogenicity Assessment**										
Blood for immunologic analysis[5]		X				X				X

**Visit Number**	0	1				2				3
**Type of Visit**	C	C	SC	SC	SC	C	SC	SC	SC	C
**Month/Weekly Timepoint**		M0				M1				M2
**Study Visit Day**	D0[1] (Screening)	D1 (Baseline)	7 days after D1 D8	14 days after D1 D15	21 days after D1 D22	D29	7 days after D29 D36	14 days after D29 D43	21 days after D29 D50	D57
**Window Allowance (Days)**	-28		+3	+3	+3	-3/+7	+3	+3	+3	-3/+7
**Days Since Most Recent Vaccination**	-	0	7	14	21	28/0	7	14	21	28
**Safety Assessments**										
eDiary activation for recording solicited adverse reactions (7 days)[6]		X				X				
Review of eDiary			X				X			
Follow-up Safety[7]			X	X	X		X	X	X	
Recording of Unsolicited AEs (Blinded Phase only)		X	X	X	X	X	X	X	X	X
Recording of MAAEs, AE leading to withdrawal and concomitant medications relevant to or for the treatment of the MAAE[8]		X	X	X	X	X	X	X	X	X
Recording of SAEs and concomitant medications relevant to or for the treatment of the SAE[8]	X	X	X	X	X	X	X	X	X	X
Recording of concomitant medications and non-study vaccinations[8]		X	X	X	X	X	X	X	X	X

Abbreviations: AE = adverse event; C = clinic visit; D = day; eDiary= electronic diary; ICF = informed consent form; M = month; MAAE = medically attended AE; SAE = serious adverse event; SC = safety (phone) call.

Note: In accordance with FDA Guidance on Conduct of Clinical Trials of Medical Products during the COVID-19 Pandemic (FDA March 2020), investigators may convert study site visits to telemedicine visits with the approval of the Sponsor. All scheduled study visits should be completed within the respective visit windows. If the participant is not able to come on site for a study site visit as a result of the COVID-19 Pandemic (self-quarantine or disruption of study site activities following business continuity plans and/or local government mandates for “stay at home” or “shelter in place”), a safety call to the participant should

be made in place of the study site visit. The safety call should encompass all scheduled visit assessments that can be completed remotely, such as assessment for AEs and concomitant medications (eg, as defined in scheduled biweekly safety phone calls). Home visits will be permitted for all non-dosing visits except for Screening if a participant cannot come to the study site as a result of the COVID19 pandemic.

1. Day 0 and Day 1 may be combined the same day. Additionally, the Day 0 visit may be performed over multiple visits if within the 28-day screening window.
2. Physical examination: a full physical examination, including vital signs, height, and weight, will be performed at Screening and Day 1. Body mass index will be calculated at the Screening Visit (Day 0) only. Symptom-directed physical examinations will be performed on Day 29 and Day 57. On each dosing day before injection, the arm receiving the injection should be examined and the associated lymph nodes should be evaluated. Any clinically significant finding identified during a study visit should be reported as a MAAE. Vital signs are to be collected pre- and post-dosing on days of injection (Day 1 and Day 29). When applicable, vital sign measurements should be performed before blood collection. Participants who are febrile (body temperature ≥ 38.0°C/100.4°F) before dosing on Day 1 or Day 29 must be rescheduled within the relevant window period to receive the injection. Afebrile participants with minor illnesses can be enrolled at the discretion of the investigator.
3. Pregnancy test at Screening and Day 1 and before the second vaccination will be a point-of-care urine test. At the discretion of the investigator a pregnancy test either via blood or point-of-care urine test can be performed. Follicle-stimulating hormone level may be measured to confirm menopausal status at the discretion of the investigator.
4. Participants with symptoms of COVID-19 will be asked to return within 72 hours or as soon as possible to the study site for an unscheduled visit, to include an NP swab sample (for RT-PCR testing) and other clinical evaluations. If a study site visit is not possible, a home visit may be arranged to collect the NP sample and conduct clinical evaluations. If a home visit is not possible, the participant will be asked to submit a saliva sample to the study site by a Sponsor-approved method. At this visit, NP swab and blood samples will be collected to evaluate for the presence of SARS-CoV-2 infection. NP swab samples will also be tested for the presence of other respiratory pathogens. In addition, the study site may collect an additional respiratory sample for SARS-CoV-2 testing to be able to render appropriate medical care for the study participant as determined by local standards of care. Additionally, clinical information will be carefully collected to evaluate the severity of the clinical case. It is important to note that some of the symptoms of COVID-19 overlap with solicited systemic ARs that are expected after vaccination with mRNA-1273 (eg, myalgia, headache, fever, and chills). During the first 7 days after vaccination, when these solicited ARs are common, investigators should use their clinical judgement to decide if an NP swab should be collected. The collection of an NP swab prior to the Day 1 and Day 29 vaccination can help ensure that cases of COVID-19 are not overlooked. Any study participant reporting respiratory symptoms during the 7-day period after vaccination should be evaluated for COVID-19.
5. Sample must be collected prior to dosing on days of injection (Day 1 and Day 29).
6. The participant will record entries in the eDiary approximately 30 minutes after dosing while at the study site, with instruction provided by study staff. Study participants will continue to record in the eDiary each day after they leave the study site, preferably in the evening, on the day of dosing and for 6 days following. Any solicited AR that is ongoing beyond Day 7 will be reported until resolution. Adverse reactions recorded in eDiaries beyond Day 7 should be reviewed either via phone call or at the following study visit. Participants will be given thermometers to record their temperatures and rulers to measure any injection site reactions.
7. Trained study personnel will call all participants to collect information relating to any unsolicited AEs through Day 57 (including any signs and symptoms of COVID-19), MAAEs, AEs leading to withdrawal, SAEs, information on concomitant medications associated with those events, and any non-study vaccinations.
8. All concomitant medications and non-study vaccinations will be recorded through 28 days after each injection; all concomitant medications relevant to or for the treatment of an SAE or MAAE will be recorded from Screening through the final visit (Day 759).

Table 17: Schedule of Events (Surveillance Phase, Day 64 – Day 394)

**Visit Number**						4						5
**Type of Visit**	eD	SC	SC	SC	SC	C	SC	SC	SC	SC	SC	C
**Month (M)/Weekly (W) Timepoint**	W	M3	M4	M5	M6	M7	M8	M9	M10	M11	M12	M13
**Study Visit Day**	D64 [1]	D85[1]	D119 [1] 90 days after D29	D149[1]	D179[1]	D209[1] 180 days after D29	D239[1]	D269[1]	D299[1]	D329[1]	D359[1]	D394[1] 365 days after D29
**Window Allowance (Days)**	±2	±3	±3	±3	±3	±14	±3	±3	±3	±3	±3	±14
**Days Since Most Recent Vaccination**	-	56	90	120	150	180	210	240	270	300	330	365
Physical examination[2]						X						X
**Efficacy Assessments**												
eDiary activation for surveillance for COVID-19/Unscheduled Visit	X											
eDiary Weekly Prompts for surveillance for COVID-19	□-------------------Weekly eDiary Prompts (Day 64 through Day 387) -----------□											
Surveillance for COVID-19/Unscheduled Visit[3]		X	X	X	X	X	X	X	X	X	X	X
**Immunogenicity Assessment**												
Blood for immunologic analysis						X						X
**Safety Assessments**												
eDiary activation for safety follow-up	X[4]											
eDiary Weekly Prompts for safety follow-up	□--------------Weekly eDiary Prompts (Day 64 through Day 387) ----------------□											

Visit Number						4						5
**Type of Visit**	eD	SC	SC	SC	SC	C	SC	SC	SC	SC	SC	C
**Month (M)/Weekly (W) Timepoint**	W	M3	M4	M5	M6	M7	M8	M9	M10	M11	M12	M13
**Study Visit Day**	D64 [1]	D85[1]	D119 [1] 90 days after D29	D149[1]	D179[1]	D209[1] 180 days after D29	D239[1]	D269[1]	D299[1]	D329[1]	D359[1]	D394[1] 365 days after D29
**Window Allowance (Days)**	±2	±3	±3	±3	±3	±14	±3	±3	±3	±3	±3	±14
**Days Since Most Recent Vaccination**	-	56	90	120	150	180	210	240	270	300	330	365
Follow-up Safety[5]		X	X	X	X	X	X	X	X	X	X	X
Recording of MAAEs, AE leading to withdrawal and concomitant medications relevant to or for the treatment of the MAAE[6]		X	X	X	X	X	X	X	X	X	X	X
Recording of SAEs and concomitant medications relevant to or for the treatment of the SAE[6]		X	X	X	X	X	X	X	X	X	X	X
Recording of concomitant medications and non-study vaccinations[6,7]		X	X	X	X	X	X	X	X	X	X	X

Abbreviations: AE = adverse event; C = clinic visit; D = day; eD = electronic diary; IRB = institutional review board; M = month; MAAE = medically attended AE; SAE = serious adverse event; SC = safety (phone) call; W = week.

Note: In accordance with FDA Guidance on Conduct of Clinical Trials of Medical Products during the COVID-19 Pandemic (FDA March 2020), investigators may convert study site visits to telemedicine visits with the approval of the Sponsor.

1. All scheduled study visits should be completed within the respective visit windows. If the participant is not able to come on site for a study site visit as a result of the COVID-19 Pandemic (self-quarantine or disruption of study site activities following business continuity plans and/or local government mandates for "stay at home" or "shelter in place"), a safety phone call to the participant should be made in place of the study site visit. The safety call should encompass all scheduled visit assessments that can be completed remotely, such as assessment for AEs and concomitant medications (eg, as defined in scheduled safety phone calls). Home visits will be permitted for all non-dosing visits except for Screening if a participant cannot come to the study site as a result of the COVID-19 pandemic. Home visits must be permitted by the site IRB and the participant via informed consent and have prior approval from the Sponsor (or its designee).
2. Symptom-directed physical examinations may be performed at the discretion of the investigator.

3. Participants with symptoms of COVID-19 will be asked to return within 72 hours or as soon as possible to the study site for an unscheduled visit, to include an NP swab sample (for RT-PCR testing) and other clinical evaluations. If a study site visit is not possible, a home visit may be arranged to collect the NP sample and conduct clinical evaluations. If a home visit is not possible, the participant will be asked to submit a saliva sample to the study site by a Sponsor-approved method. At this visit, NP swab and blood samples will be collected to evaluate for the presence of SARS-CoV-2 infection. NP swab samples will also be tested for the presence of other respiratory pathogens. In addition, the study site may collect an additional respiratory sample for SARS-CoV-2 testing to be able to render appropriate medical care for the study participant as determined by local standards of care. Additionally, clinical information will be carefully collected to evaluate the severity of the clinical case.
4. The eDiary Safety Follow-up Prompts will be triggering off of Day 61 to take into consideration the (-3 day) window allowance at Day 29. This first Safety Follow-Up prompt at D61 will follow the same 7-day surveillance per protocol.
5. Trained study personnel will call all participants to collect information relating to any sign/symptoms of COVID-19, MAAEs, AEs leading to withdrawal, SAEs, information on concomitant medications associated with those events, and any non-study vaccinations.
6. All concomitant medications relevant to or for the treatment of an SAE or MAAE will be recorded from Screening through the final visit (Day 759).
7. One additional ePRO prompt will be sent to participants specifically to solicit the collection of information regarding participant’s history of facial injections or dermal fillers, for cosmetic or medical indications such as migraine headaches.

Table 18: Schedule of Events (Surveillance Phase, Day 401 – Day 759)

**Visit Number**												6
**Type of Visit**	SC	SC	SC	SC	SC	SC	SC	SC	SC	SC	SC	C
**Month (M)/Weekly (W) Timepoint**	M14	M15	M16	M17	M18	M19	M20	M21	M22	M23	M24	M25
**Study Visit Day**	D424[1]	D454[1]	D484[1]	D514[1]	D544[1]	D574[1]	D604[1]	D634[1]	D664[1]	D694[1]	D724[1]	D759[1] 365 days after Year 1
**Window Allowance (Days)**	±3	±3	±3	±3	±3	±3	±3	±3	±3	±3	±3	±14
**Days Since Most Recent Vaccination**												730
Physical examination[2]												X
eDiary Weekly Prompts for surveillance for COVID-19		□--------Weekly eDiary Prompts (Day 401 through Day 752) --------------□										
Surveillance for COVID-19/Unscheduled Visit[3]	X	X	X	X	X	X	X	X	X	X	X	X
Blood for immunologic analysis												X
eDiary Weekly Prompts for safety follow-up		□--------Weekly eDiary Prompts (Day 401 through Day 752) --------------□										
Follow-up Safety[4]	X	X	X	X	X	X	X	X	X	X	X	X
Recording of MAAEs, AE leading to withdrawal and concomitant medications relevant to or for the treatment of the MAAE[5]	X	X	X	X	X	X	X	X	X	X	X	X
Recording of SAEs and concomitant medications relevant to or for the treatment of the SAE[5]	X	X	X	X	X	X	X	X	X	X	X	X
Recording of concomitant medications and non-study vaccinations[5]	X	X	X	X	X	X	X	X	X	X	X	X

Abbreviations: AE = adverse event; C = clinic visit; D = day; eD = electronic diary; IRB = institutional review board; M = month; MAAE = medically attended AE; SAE = serious adverse event; SC = safety (phone) call.

Note: In accordance with FDA Guidance on Conduct of Clinical Trials of Medical Products during the COVID-19 Pandemic (FDA March 2020), investigators may convert study site visits to telemedicine visits with the approval of the Sponsor.

1. All scheduled study visits should be completed within the respective visit windows. If the participant is not able to come on site for a study site visit as a result of the COVID-19 Pandemic (self-quarantine or disruption of study site activities following business continuity plans and/or local government mandates for “stay at home” or “shelter in place”), a safety phone call to the participant should be made in place of the study site visit. The safety phone call should encompass all scheduled visit assessments that can be completed remotely, such as assessment for AEs and concomitant medications (eg, as defined in scheduled safety phone calls). Home visits will be permitted for all non-dosing visits except for Screening if a participant cannot come to the study site as a result of the COVID-19 pandemic. Home visits must be permitted by the site IRB and the participant via informed consent and have prior approval from the Sponsor (or its designee).
2. Symptom-directed physical examinations may be performed at the discretion of the investigator.
3. Participants with symptoms of COVID-19 will be asked to return within 72 hours or as soon as possible to the study site for an unscheduled visit, to include an NP swab sample (for RT-PCR testing) and other clinical evaluations. If a study site visit is not possible, a home visit may be arranged to collect the NP sample and conduct clinical evaluations. If a home visit is not possible, the participant will be asked to submit a saliva sample to the study site by a sponsor-approved method. At this visit, NP swab and blood samples will be collected to evaluate for the presence of SARS-CoV-2 infection. NP swabs will also be tested for the presence of other respiratory pathogens. In addition, the study site may collect an additional NP sample for SARS-CoV-2 testing to be able to render appropriate medical care for the study participant as determined by local standards of care. Additionally, clinical information will be carefully collected to evaluate the severity of the clinical case.
4. Trained study personnel will call all participants to collect information relating to any MAAEs, AEs leading to withdrawal, SAEs, information on concomitant medications associated with those events, and any non-study vaccinations.
5. All concomitant medications relevant to or for the treatment of an SAE or MAAE will be recorded from Screening through the final visit (Day 759).

Table 19: Schedule of Events (Convalescent Period, Starting with the Illness Visit)

**Unscheduled Visit**	1								2
**Type of Visit**	C/H								C/H
**Daily Timepoint**	D1	D2- D6	D7	D8-D13	D14	D15-D20	D21	D22-D27	D28
**Window Allowance (Days)**	-	±1	±1	±1	±1	±1	±1	±1	+7
**Safety Assessments**									
Symptom-directed Physical examination[1]	X								X
Follow-up Safety[2]	X								X
Recording of MAAEs, AE leading to withdrawal and concomitant medications relevant to or for the treatment of the MAAE[3]	X								X
Recording of SAEs and concomitant medications relevant to or for the treatment of the SAE[3]	X								X
Recording of concomitant medications and non-study vaccinations[3]	X								X
**Efficacy Assessments**									
Daily Telemedicine Visit[4]		□--Daily--------------------------□							
Respiratory illness sample[5]	X								
Blood sample for immunologic assessment of SARS-CoV-2 infection[6]	X								X
Saliva sample		D3[7], D5[7]	D7[7]	D9[7]	D14[7]		D21[7]		X[8]

Abbreviations: C = clinic visit; CLIA = Clinical Laboratory Improvement Amendments; D = day; H = home visit; MAAE = medically attended AE; SAE = serious adverse event

Note: In accordance with FDA Guidance on Conduct of Clinical Trials of Medical Products during the COVID-19 Pandemic (FDA March 2020), investigators may convert study site visits to telemedicine visits with the approval of the Sponsor.

1. Physical examination: a symptom-directed physical examination, including vital signs will be performed at the initial visit to confirm the diagnosis (denoted as D1 in this table) and at the Convalescent Visit (28 days after diagnosis [D28 in this table]).
2. Trained study personnel will call all participants to collect information relating to any AEs, MAAEs, AEs leading to withdrawal, SAEs, information on concomitant medications associated with those events, and any non-study vaccinations. All safety events will be followed until resolution.

3. All concomitant medications relevant to or for the treatment of an SAE or MAAE will be recorded from Screening through the final visit (Day 759).
4. Participants will have daily telemedicine visits (via video or phone) for 14 days (or until symptoms resolve, whichever is longer). Telemedicine visits may be performed by medically qualified staff appropriately delegated by the investigator. During the telemedicine visit (preferably done in the evening) the participant will be asked to verbally report the severity of each symptom, their highest body temperature and lowest oxygen saturation for that day and the investigator will determine if medical attention is required due to worsening of COVID-19. The participant will also be reminded to collect a saliva sample (or nasal swab) and return it to the study site, on the appropriate days.
5. Participants with symptoms of COVID-19 will be asked to return within 72 hours or as soon as possible to the study site or medically qualified staff from the site will conduct a home visit as soon as possible to collect an NP swab sample (for RT-PCR) and collect a blood sample for immunologic assessment of SARS-CoV-2 infection and evaluate for COVID-19. NP swab sample will also be tested for the presence of other respiratory infections. In addition, the study site may collect an additional respiratory sample for SARS-CoV-2 testing to be able to render appropriate medical care for the study participant as determined by local standards of care. Additionally, clinical information will be carefully collected to evaluate the severity of the clinical case. If the RT-PCR test from the NP swab sample from the Illness Visit is negative for SARS-CoV-2, the participant will exit the Convalescent Period and resume the study schedule (Table 16, Table 17, and Table 18).
6. This can be a home visit if necessary.
7. Participants will collect their own saliva sample using the saliva collection tubes provided, or a nasal swab, on 3, 5, 7, 9, 14, and 21 days after the initial Illness Visit, and return them to the study site according to Sponsor instructions. Participants who are pending a central laboratory PCR may exit from the convalescent period based on a negative PCR result from a CLIA-certified or CLIA-certified waiver local laboratory at the investigator’s discretion.
8. At this visit, a saliva (or nasal swab) sample will be collected.

Table 20: Part B: Participant Decision Clinic Visit

	All Participants				
Sign revised Informed Consent Form	X				
Confirm participant's request to be unblinded or not to be unblinded[a]	X				
Nasopharyngeal swab	X				
Blood for immunologic analysis	X				
Counselling the importance of public health measures[b]	X				
		Open-label Cohort			
**Participant Status after Participant Decision Clinic Visit**	**Blinded Cohort**	**Previously Receiving mRNA-1273**	**Remaining on Placebo**	**Placebo Requesting mRNA-1273**	**mRNA-1273 who received 1 dose ONLY**
Continue with original Schedules of Events, as applicable (Table 16, Table 17, Table 18, and Table 19)	X	X	X	X	X
Supplemental Schedule of Events: Open-Label Days 1-57 (Table 21)[c]	--	--	--	X	--
Modified Supplemental Schedule of Events: Open-Label Days 1-57 (Table 22)[c]	--	--	--	--	X

[a] At the point when the mRNA-1273 vaccine is no longer available for study use, any participant who schedules a Participant Decision Visit after this time-point will be unblinded (with no option to stay blinded), but will not be offered m-RNA-1273 study vaccine.

[b] All participants are counselled about the importance of continuing other public health measures to limit the spread of disease including physical-social distancing, wearing a mask, and hand-washing.

[c] The Supplemental and the Modified Supplemental Schedule of Events are intended to occur in addition to the original Schedules of Events, as applicable (Table 16, Table 17, Table 18, and Table 19) and therefore there is a possibility for study visits to overlap. If visits overlap according to respective visit windows, a single visit may be done with the combined study procedures completed once.

Table 21: Supplemental Schedule of Events: Open-Label Observational Phase - Placebo Participants who Request to Receive mRNA-1273

NOTE: Supplemental Schedule of Events: Open-Label Observational Phase is to be used ONLY for participants who received placebo during the blinded phase of this study (Part A), and request to receive mRNA-1273. These participants will comply with this Supplemental SoE (Table 21) in addition to the original SoEs as applicable (Table 16, Table 17, Table 18, and Table 19). As this Supplemental SoE is intended to occur in addition to the original Schedules of Events, there is a possibility for study visits to overlap. If visits overlap according to respective visit windows, a single visit may be done with the combined study procedures completed once.

**Visit Number**	0	OL-1		OL-2		OL-3
**Type of Visit**	C	C	SC	C	SC	C
**Study Visit Day**	Participant Decision Visit[1] and OL-D1[1]		7 days after OL-D1 OL-D8	OL-D29	7 days after OL-D29 OL-D36	OL-D57
**Window Allowance (Days)**	?		+3	-3/+14	+3	-3/+14
**Days Since Most Recent Vaccination**	0		7	28/0	7	28
Confirm signing of ICF, concomitant medications, medical history	X					
Confirm participant meets inclusion and exclusion criteria	X					
Physical examination[2]	X			X		X
Pregnancy testing[3]	X			X		
**Immunogenicity Assessment**						
Blood for immunologic analysis[5]	X					X
**Dosing**						
Study injection (including 30-minute post-dosing observation period[6])	X			X		
**Efficacy Assessment**						

NOTE: Supplemental Schedule of Events: Open-Label Observational Phase is to be used ONLY for participants who received placebo during the blinded phase of this study (Part A), and request to receive mRNA-1273. These participants will comply with this Supplemental SoE (Table 21) in addition to the original SoEs as applicable (Table 16, Table 17, Table 18, and Table 19). As this Supplemental SoE is intended to occur in addition to the original Schedules of Events, there is a possibility for study visits to overlap. If visits overlap according to respective visit windows, a single visit may be done with the combined study procedures completed once.

**Visit Number**	0	OL-1		OL-2		OL-3
**Type of Visit**	C	C	SC	C	SC	C
**Study Visit Day**	Participant Decision Visit[1] and OL-D1[1]		7 days after OL-D1 OL-D8	OL-D29	7 days after OL-D29 OL-D36	OL-D57
**Window Allowance (Days)**	?		+3	-3/+14	+3	-3/+14
**Days Since Most Recent Vaccination**	0		7	28/0	7	28
Surveillance for COVID-19/Unscheduled Visit[4]	X		X	X	X	X
Nasopharyngeal swab[5]	X					
**Safety Assessments**						
Follow-up Safety[7]			X		X	
Recording of MAAEs, AE leading to withdrawal and concomitant medications relevant to or for the treatment of the MAAE[8]	X		X	X	X	X
Recording of SAEs and concomitant medications relevant to or for the treatment of the SAE[8]	X		X	X	X	X
Recording of concomitant medications and non-study vaccinations[8]	X		X	X	X	X

Abbreviations: AE = adverse event; C = clinic visit; D = day; eDiary = electronic diary; ICF = informed consent form; OL = open-label; MAAE = medically attended AE; SAE = serious adverse event; SC = safety (phone) call.

Note: In accordance with FDA Guidance on Conduct of Clinical Trials of Medical Products during the COVID-19 Pandemic (FDA March 2020), investigators may convert study site visits to telemedicine visits with the approval of the Sponsor. All scheduled study visits should be completed within the respective visit windows. If the participant is not able to come on site for a study site visit as a result of the COVID-19 Pandemic (self-quarantine or disruption of study site activities following business continuity plans and/or local government mandates for “stay at home” or “shelter in place”), a safety call to the participant should be made in place of the study site visit. The safety call should encompass all scheduled visit assessments that can be completed remotely, such as assessment for AEs and concomitant medications (eg, as defined in scheduled biweekly safety phone calls). Home visits will be permitted for all non-dosing visits except for Screening if a participant cannot come to the study site as a result of the COVID19 pandemic.

1. Day 0 and OL-D1 visit may be performed on 2 separate visits.
2. Symptom-directed physical examination will be performed at the Participant Decision Visit and on OL-D29. On each dosing day before injection, the arm receiving the injection should be examined and the associated lymph nodes should be evaluated. Any clinically significant finding identified during a study visit should be reported as a MAAE. Vital signs are to be collected pre- and post-dosing on days of injection (OL-D1 and OL-D29). Participants who are febrile (body temperature ≥ 38.0°C/100.4°F) before dosing (OL-D1 and OL-D29) must be rescheduled within the relevant window period to receive the injection. Afebrile participants with minor illnesses can be enrolled at the discretion of the investigator.
3. Pregnancy test at the Participant Decision Visit and OL-D29 will be a point-of-care urine test. At the discretion of the investigator, a pregnancy test either via blood or point-of-care urine test can be performed. Follicle-stimulating hormone level may be measured to confirm menopausal status at the discretion of the investigator.
4. Participants with symptoms of COVID-19 will be asked to return within 72 hours or as soon as possible to the study site for an unscheduled visit, to include an NP swab sample (for RT-PCR testing) and other clinical evaluations. If a study site visit is not possible, a home visit may be arranged to collect the NP sample and conduct clinical evaluations. If a home visit is not possible, the participant will be asked to submit a saliva sample to the study site by a Sponsor-approved method. At this visit, NP swab and blood samples will be collected to evaluate for the presence of SARS-CoV-2 infection. NP swab samples will also be tested for the presence of other respiratory pathogens. In addition, the study site may collect an additional respiratory sample for SARS-CoV-2 testing to be able to render appropriate medical care for the study participant as determined by local standards of care. Additionally, clinical information will be carefully collected to evaluate the severity of the clinical case. It is important to note that some of the symptoms of COVID-19 overlap with solicited systemic ARs that are expected after vaccination with mRNA-1273 (eg, myalgia, headache, fever, and chills). During the first 7 days after vaccination, when these solicited ARs are common, investigators should use their clinical judgement to decide if an NP swab should be collected. The collection of an NP swab prior to the Participant Decision Visit can help ensure that cases of COVID-19 are not overlooked. Any study participant reporting respiratory symptoms during the 7-day period after vaccination should be evaluated for COVID-19.
5. Sample must be collected prior to unblinding and injection at the Participant Decision Visit.
6. Post-dosing, participants will have a 30-minute observation period.
7. Trained study personnel will call all participants to collect information relating to any MAAEs (including any signs and symptoms of COVID-19), AEs leading to withdrawal, SAEs, information on concomitant medications associated with those events, and any non-study vaccinations.
8. All concomitant medications relevant to or for the treatment of an SAE or MAAE will be recorded from Screening through the final visit (Day 759).

Table 22: Modified Supplemental Schedule of Events: Open-Label Observational Phase – mRNA-1273 Participants who ONLY received 1 dose of mRNA-1273

NOTE: Modified Supplemental Schedule of Events: Open-Label Observational Phase is to be used ONLY for participants who ONLY received 1 dose of mRNA-1273 during the blinded phase of this study (Part A), and consented to receive mRNA-1273 (see Section 4.1.2 for details). These participants will comply with this SoE (Table 22) in addition to the original SoEs as applicable (Table 16, Table 17, Table 18, and Table 19). As this Modified Supplemental SoE is intended to occur in addition to the original Schedules of Events, there is a possibility for study visits to overlap. If visits overlap according to respective visit windows, a single visit may be done with the combined study procedures completed once.

**Visit Number**	0	OL-1		OL-2
**Type of Visit**	C	C	SC	C
**Study Visit Day**	Participant Decision Visit[1] and OL-D1[1]		7 days after OL-D1 OL-D8	OL-D29
**Window Allowance (Days)**	-		+3	-3/+14
**Days Since Most Recent Vaccination**	0		7	28/0
Confirm signing of ICF, concomitant medications, medical history	X			
Confirm participant meets inclusion and exclusion criteria	X			
Physical examination[2]	X			X
Pregnancy testing[3]	X			
**Immunogenicity Assessment**				
Blood for immunologic analysis[5]	X			X
**Dosing**				
Study injection (including 30-minute post-dosing observation period[6])	X			

**NOTE: Modified Supplemental Schedule of Events: Open-Label Observational Phase is to be used ONLY for participants who ONLY received 1 dose of mRNA-1273 during the blinded phase of this study (Part A), and consented to receive mRNA-1273 (see Section 4.1.2 for details). These participants will comply with this SoE (Table 22) in addition to the original SoEs as applicable (Table 16, Table 17, Table 18, and Table 19). As this Modified Supplemental SoE is intended to occur in addition to the original Schedules of Events, there is a possibility for study visits to overlap. If visits overlap according to respective visit windows, a single visit may be done with the combined study procedures completed once.**				
**Visit Number**	0	OL-1		OL-2
**Type of Visit**	C	C	SC	C
**Study Visit Day**	Participant Decision Visit[1] and OL-D1[1]		7 days after OL-D1 OL-D8	OL-D29
**Window Allowance (Days)**	-		+3	-3/+14
**Days Since Most Recent Vaccination**	0		7	28/0
**Efficacy Assessment**				
Surveillance for COVID-19/Unscheduled Visit[4]	X		X	X
Nasopharyngeal swab[5]	X			
**Safety Assessments**				
Follow-up Safety[7]			X	
Recording of MAAEs, AE leading to withdrawal and concomitant medications relevant to or for the treatment of the MAAE[8]	X		X	X
Recording of SAEs and concomitant medications relevant to or for the treatment of the SAE[8]	X		X	X
Recording of concomitant medications and non-study vaccinations[8]	X		X	X

Abbreviations: AE = adverse event; C = clinic visit; D = day; eDiary = electronic diary; ICF = informed consent form; OL = open-label; MAAE = medically attended AE; SAE = serious adverse event; SC = safety (phone) call.

Note: In accordance with FDA Guidance on Conduct of Clinical Trials of Medical Products during the COVID-19 Pandemic (FDA March 2020), investigators may convert study site visits to telemedicine visits with the approval of the Sponsor. All scheduled study visits should be completed within the respective visit windows. If the participant is not able to come on site for a study site visit as a result of the COVID-19 Pandemic (self-quarantine or disruption of study site activities following business continuity plans and/or local government mandates for "stay at home" or "shelter in place"), a safety call to the participant should be made in place of the study site visit. The safety call should encompass all scheduled visit assessments that can be completed remotely, such as assessment for AEs and concomitant medications (eg, as defined in scheduled biweekly safety phone calls). Home visits will be permitted for all non-dosing visits except for Screening if a participant cannot come to the study site as a result of the COVID19 pandemic.

1. Day 0 and OL-D1 visit may be performed on 2 separate visits.
2. Symptom-directed physical examination will be performed at the Participant Decision Visit. On dosing day before injection, the arm receiving the injection should be examined and the associated lymph nodes should be evaluated. Any clinically significant finding identified during a study visit should be reported as a MAAE. Vital signs are to be collected pre- and post-dosing on the days of injection (OL-D1). Participants who are febrile (body temperature ≥ 38.0°C/100.4°F) before dosing (OL-D1) must be rescheduled within the relevant window period to receive the injection. Afebrile participants with minor illnesses can be enrolled at the discretion of the investigator.
3. Pregnancy test at the Participant Decision Visit will be a point-of-care urine test. At the discretion of the investigator, a pregnancy test either via blood or point-of-care urine test can be performed. Follicle-stimulating hormone level may be measured to confirm menopausal status at the discretion of the investigator.
4. Participants with symptoms of COVID-19 will be asked to return within 72 hours or as soon as possible to the study site for an unscheduled visit, to include an NP swab sample (for RT-PCR testing) and other clinical evaluations. If a study site visit is not possible, a home visit may be arranged to collect the NP sample and conduct clinical evaluations. If a home visit is not possible, the participant will be asked to submit a saliva sample to the study site by a Sponsor-approved method. At this visit, NP swab and blood samples will be collected to evaluate for the presence of SARS-CoV-2 infection. NP swab samples will also be tested for the presence of other respiratory pathogens. In addition, the study site may collect an additional respiratory sample for SARS-CoV-2 testing to be able to render appropriate medical care for the study participant as determined by local standards of care. Additionally, clinical information will be carefully collected to evaluate the severity of the clinical case. It is important to note that some of the symptoms of COVID-19 overlap with solicited systemic ARs that are expected after vaccination with mRNA-1273 (eg, myalgia, headache, fever, and chills). During the first 7 days after vaccination, when these solicited ARs are common, investigators should use their clinical judgement to decide if an NP swab should be collected. The collection of an NP swab prior to the Participant Decision Visit can help ensure that cases of COVID-19 are not overlooked. Any study participant reporting respiratory symptoms during the 7-day period after vaccination should be evaluated for COVID-19.
5. Sample must be collected prior to unblinding and injection at the Participant Decision Visit.
6. Post-dosing, participants will have a 30-minute observation period.
7. Trained study personnel will call all participants to collect information relating to any MAAEs (including any signs and symptoms of COVID-19), AEs leading to withdrawal, SAEs, information on concomitant medications associated with those events, and any non-study vaccinations.
8. All concomitant medications relevant to or for the treatment of an SAE or MAAE will be recorded from Screening through the final visit (Day 759).

11.2. APPENDIX 2: Study Governance Considerations

11.2.1. Regulatory and Ethical Considerations

This study will be conducted in accordance with the protocol and with the following:

- Consensus ethical principles derived from international guidelines including the Declaration of Helsinki and Council for International Organizations of Medical Sciences (CIOMS) International Ethical Guidelines.
- Applicable ICH GCP Guidelines.
- Applicable laws and regulatory requirements.
- The protocol, protocol amendments, ICF, Investigator Brochure, and other relevant documents (eg, advertisements) must be submitted to an IRB/IEC by the investigator and reviewed and approved by the IRB/IEC before the study is initiated.
- Any amendments to the protocol will require IRB/IEC approval before implementation of changes made to the study design, except for changes necessary to eliminate an immediate hazard to study participants.
- The investigator will be responsible for the following:

– Providing written summaries of the status of the study to the IRB/IEC annually or more frequently in accordance with the requirements, policies, and procedures established by the IRB/IEC

– Notifying the IRB/IEC of SAEs or other significant safety findings as required by IRB/IEC procedures

– Providing oversight of the conduct of the study at the site and adherence to requirements of 21 CFR, ICH guidelines, the IRB/IEC, European regulation 536/2014 for clinical studies (if applicable), and all other applicable local regulations.

11.2.2. Study Monitoring

Before an investigational site can enter a participant into the study, a representative of Moderna or its representatives will visit the investigational study site to:

- Determine the adequacy of the facilities.
- Discuss with the investigator(s) and other personnel their responsibilities with regard to protocol adherence, and the responsibilities of Moderna or its representatives. This will be documented in a Clinical Study Agreement between Moderna, designated CRO, and the investigator.

According to ICH GCP guideline, the Sponsor of the study is responsible for ensuring the proper conduct of the study with regard to protocol adherence and validity of data recorded on the eCRFs. The study monitor's duties are to aid the investigator and Moderna in the maintenance of complete, accurate, legible, well-organized, and easily retrievable data. The study monitor will advise the investigator of the regulatory necessity for study-related monitoring, audits, IRB/IEC review, and inspection by providing direct access to the source data/documents. In addition, the study monitor will explain to and interpret for the investigator all regulations applicable to the clinical evaluation of an IP as documented in ICH guidelines.

It is the study monitor's responsibility to inspect the eCRFs and source documentation throughout the study to protect the rights of the participants; to verify adherence to the protocol; to verify completeness, accuracy, and consistency of the data; and to confirm adherence of study conduct to any local regulations. Details will be outlined in the Clinical Monitoring Plan. During the study, a monitor from Moderna or a representative will have regular contacts with the investigational site, for the following:

- Provide information and support to the investigator(s).
- Confirm that facilities remain acceptable.
- Confirm that the investigational team is adhering to the protocol, that the data are being accurately recorded in the eCRFs, and that IP accountability checks are being performed.
- Perform source data verification. This includes a comparison of the data in the eCRFs with the participant's medical records at the hospital or practice, and other records relevant to the study. This will require direct access to all original records for each participant (eg, clinical charts or electronic medical record system).
- Record and report any protocol deviations not previously sent.
- Confirm AEs and SAEs have been properly documented on eCRFs and confirm any SAEs have been forwarded to the SAE Hotline, and those SAEs that met criteria for reporting have been forwarded to the IRB/IEC.

The monitor will be available between visits if the investigator(s) or other staff needs information or advice.

In accordance with FDA Guidance on Conduct of Clinical Trials of Medical Products during the COVID-19 Pandemic (FDA March 2020), investigators may convert study site visits to telemedicine visits with the approval of the Sponsor (Section 8 for Procedures).

The DSMB will also have responsibility for safety monitoring (Section 8.4.1).

11.2.3. Audits and Inspections

Moderna, their designee(s), the IRB/IEC, or regulatory authorities will be allowed to conduct site visits to the investigational facilities for the purpose of monitoring or inspecting any aspect of the study. The investigator agrees to allow Moderna, their designee(s), the IRB/IEC, or regulatory authorities to inspect the IP storage area, IP stocks, IP records, participant charts and study source documents, and other records relative to study conduct.

Authorized representatives of Moderna, a regulatory authority, and any IRB/IEC may visit the site to perform audits or inspections, including source data verification. The purpose of a Sponsor audit or inspection is to systematically and independently examine all study-related activities and documents to determine whether these activities were conducted, and data were recorded, analyzed, and accurately reported according to the protocol, ICH GCP E6R2, and any applicable regulatory requirements. The investigator should contact Moderna immediately if contacted by a regulatory agency about an inspection.

The Principal Investigator must obtain IRB approval for the investigation. Initial IRB approval, and all materials approved by the IRB for this study including the participant consent form and recruitment materials must be maintained by the investigator and made available for inspection.

11.2.4. Financial Disclosure

The investigator is required to provide financial disclosure information to allow the Sponsor to submit the complete and accurate certification or disclosure statements required under 21 CFR 54. In addition, the investigator must provide the Sponsor with a commitment to promptly update this information if any relevant changes occur during the course of the investigation and for 1 year following the completion of the study.

The Sponsor, the CRO, and the study site are not financially responsible for further testing or treatment of any medical condition that may be detected during the screening process. In addition, in the absence of specific arrangements, the Sponsor, the CRO, and the study site are not financially responsible for further treatment of the disease under study.

11.2.5. Recruitment Procedures

Advertisements to be used for the recruitment of study participants, and any other written information regarding this study to be provided to the participant should be submitted to the Sponsor for approval. All documents must be approved by the IRB.

11.2.6. Informed Consent Process

The informed consent document(s) must meet the requirements of 21 CFR 50, local regulations, ICH guidelines, Health Insurance Portability and Accountability Act (HIPAA) requirements,

where applicable, and the IRB/IEC or study center. All consent documents will be approved by the appropriate IRB/IEC. The actual ICF used at each center may differ, depending on local regulations and IEC / IRB requirements. However, all versions must contain the standard information found in the sample ICF provided by the Sponsor. Any change to the content of the ICF must be approved by the Sponsor and the IEC / IRB prior to the form being used.

If new information becomes available that may be relevant to the participant's willingness to continue participation in the study, this will be communicated to him / her in a timely manner. Such information will be provided via a revised ICF or an addendum to the original ICF.

The investigator or his/her representative will explain the nature of the study to the participant and answer all questions regarding the study.

The investigator is responsible for ensuring that the participant fully understands the nature and purpose of the study. Information should be given in both oral and written form whenever possible.

No participant should be obliged to participate in the study. The participant must be informed that participation is voluntary. Participants, their relatives, guardians, or (if applicable) legal representatives must be given ample opportunity to inquire about details of the study. The information must make clear that refusal to participate in the study or withdrawal from the study at any stage is without any prejudice to the participant's subsequent care.

The participant must be allowed sufficient time to decide whether they wish to participate. The participant must be made aware of and give consent to direct access to his/her source medical records by study monitors, auditors, the IRB/IEC, and regulatory authorities. The participant should be informed that such access will not violate participant confidentiality or any applicable regulations. The participant should also be informed that he/she is authorizing such access by signing the ICF.

A copy of the ICF(s) must be provided to the participant.

A participant who is rescreened is not required to sign another ICF if the rescreening occurs within 28 days from the previous ICF signature date.

The ICF will also explain that excess serum from immunogenicity testing may be used for future research, which may be performed at the discretion of the Sponsor to further characterize the immune response to SARS-CoV-2, additional assay development, and the immune response across CoV.

11.2.7. Protocol Amendments

No change or amendment to this protocol may be made by the investigator or the Sponsor after the protocol has been agreed to and signed by all parties unless such change(s) or amendment(s) has (have) been agreed upon by the investigator or the Sponsor. Any change agreed upon will be recorded in writing, and the written amendment will be signed by the investigator and the Sponsor. Institutional review board approval is required prior to the implementation of an amendment, unless overriding safety reasons warrant immediate action, in which case the IRB(s)/IEC(s) will be promptly notified.

Any modifications to the protocol or the ICF, which may impact the conduct of the study, potential benefit of the study, or may affect participant safety, including changes of study objectives, study design, participant population, sample sizes, study procedures, or significant administrative aspects will require a formal amendment to the protocol. Such amendment will be released by the Sponsor, agreed by the investigator(s), and approved by the relevant IRB(s)/IEC(s) prior to implementation. A signed and dated statement that the protocol, any subsequent relevant amended documents and the ICF have been approved by relevant IRB(s)/IEC(s) must be provided to the Sponsor before the study is initiated.

Administrative changes of the protocol are minor corrections and/or clarifications that have no effect on the way the study is to be conducted. These administrative changes will be released by the Sponsor, agreed by the investigator(s), and notified to the IRB(s)/IEC(s).

11.2.8. Protocol Deviations

The noncompliance may be either on the part of the participant, the investigator, or the study site staff. As a result of deviations, corrective actions are to be developed by the site and implemented promptly.

It is the responsibility of the site investigator to use continuous vigilance to identify and report deviations to the Sponsor or its designee. All deviations must be addressed in study source documents, reported to study monitor. Protocol deviations must be sent to the reviewing IRB/IEC per their policies. The site investigator is responsible for knowing and adhering to the reviewing IRB/IEC requirements.

11.2.9. Data Protection

Participants will be assigned a unique identifier by the Sponsor. Any participant records or datasets that are transferred to the Sponsor will contain the identifier only; participant names or any information which would make the participant identifiable will not be transferred.

The participant must be informed that his/her personal study-related data will be used by the Sponsor in accordance with local data protection law. The level of disclosure must also be explained to the participant.

The participant must be informed that his/her medical records may be examined by Clinical Quality Assurance auditors or other authorized personnel appointed by the Sponsor, by appropriate IRB/IEC members, and by inspectors from regulatory authorities.

Individual participant medical information obtained as a result of this study is considered confidential, and disclosure to third parties is prohibited. Information will be accessible to authorized parties or personnel only. Medical information may be given to the participant's physician or to other appropriate medical personnel responsible for the participant's well-being. Each participant will be asked to complete a form allowing the investigator to notify the participant's primary health care provider of his/her participation in this study.

All laboratory specimens, evaluation forms, reports, and other records will be identified in a manner designed to maintain participant confidentiality. All records will be kept in a secure storage area with limited access. Clinical information will not be released without the written permission of the participant, except as necessary for monitoring and auditing by the Sponsor, its designee, relevant regulatory authority, or the IRB.

The investigator and all employees and coworkers involved with this study may not disclose or use for any purpose other than performance of the study, any data, record, or other unpublished, confidential information disclosed to those individuals for the purpose of the study. Prior written agreement from the Sponsor or its designee must be obtained for the disclosure of any confidential information to other parties.

11.2.10. Sample Retention and Future Biomedical Research

The retention period of laboratory samples will be 15 years, or as permitted by local regulations, to address further scientific questions related to mRNA-1273 or anti-respiratory virus immune response. In addition, identifiable samples can be destroyed at any time at the request of the participant. During the study, or during the retention period, in addition to the analysis outlined in the study endpoints, exploratory analysis may be conducted, using other antibody-based methodologies, on any remaining blood or serum samples, including participants who provide samples for screening, but are not subsequently enrolled. These analyses would extend the search for other potentially relevant biomarkers to investigate the effect of mRNA-1273, as well as to determine how changes in the markers may relate to exposure and clinical outcomes. A decision to perform such exploratory research may arise from new scientific findings related to the drug class or disease, as well as reagent and assay availability.

11.2.11. Data Quality Assurance and Quality Control

Data collection is the responsibility of the clinical study staff at the site under the supervision of the site investigator. The investigator is responsible for ensuring the accuracy, completeness, legibility, and timeliness of the data reported.

- All participant data relating to the study will be recorded eCRF unless transmitted to the Sponsor or designee electronically (eg, laboratory data). The investigator is responsible for verifying that data entries are accurate and correct by physically or electronically signing the eCRF.
- The investigator must maintain accurate documentation (source data) that supports the information entered in the eCRF.
- The investigator must permit study-related monitoring, audits, IRB/IEC review, and regulatory agency inspections and provide direct access to source data documents.
- Monitoring details describing strategy (eg, risk-based initiatives in operations and quality such as Risk Management and Mitigation Strategies and Analytical Risk-Based Monitoring), methods, responsibilities and requirements, including handling of noncompliance issues and monitoring techniques (central, remote, or on-site monitoring) are provided in the study Monitoring Plan, Centralized Monitoring Plan, and Risk Management Plan.
- The Sponsor or designee is responsible for the data management of this study including quality checking of the data.
- The Sponsor assumes accountability for actions delegated to other individuals (eg, Contract Research Organizations).
- Study monitors will perform ongoing source data verification to confirm that data entered into the CRF by authorized site personnel are accurate, complete, and verifiable from source documents; that the safety and rights of participants are being protected; and that the study is being conducted in accordance with the currently approved protocol and any other study agreements, ICH GCP, and all applicable regulatory requirements.
- Records and documents, including signed ICFs, pertaining to the conduct of this study must be retained by the investigator for 15 years after study completion unless local regulations or institutional policies require a longer retention period. No records may be destroyed during the retention period without the written approval of the Sponsor. No records may be transferred to another location or party without written notification to the Sponsor.

Quality assurance (QA) includes all the planned and systematic actions that are established to ensure that the clinical study is performed, and the data are generated, documented (recorded), and reported according to ICH GCP and local/regional regulatory standards.

A QA representative from the Sponsor or qualified designee, who is independent of and separated from routine monitoring, may periodically arrange inspections/audits of the clinical study by reviewing the data obtained and procedural aspects. These inspections may include on-site inspections/audits and source data checks. Direct access to source documents is required for the purpose of these periodic inspections/audits.

11.2.12. Data Collection and Management

This study will be conducted in compliance with ICH CGP guidelines. This study will also be conducted in accordance with the most recent version of the Declaration of Helsinki.

This study will use electronic data collection (to collect data directly from the investigational site using eCRFs). The investigator is responsible for ensuring that all sections of each eCRF are completed promptly and correctly and that entries can be verified against any source data.

Study monitors will perform source document verification to identify inconsistencies between the eCRFs and source documents. Discrepancies will be resolved in accordance with the principles of GCP. Detailed study monitoring procedures are provided in the Clinical Monitoring Plan.

AEs will be coded with the most current available version of MedDRA. Concomitant medications will be coded using WHO – Drug Reference List.

11.2.13. Source Documents

Source documents are original documents or certified copies, and include, but are not limited to, diaries, medical and hospital records, screening logs, informed consent / assent forms, telephone contact logs, and worksheets. Source documents provide evidence for the existence of the participant and substantiate the integrity of the data collected. Source documents are filed at the investigator's site.

Data reported on the CRF or entered in the eCRF that are transcribed from source documents must be consistent with the source documents or the discrepancies must be explained. The investigator may need to request previous medical records or transfer records, depending on the study. Also, current medical records must be available.

Moderna or its designee requires that the investigator prepare and maintain adequate and accurate records for each participant treated with the IP. Source documents such as any hospital, clinic, or office charts and the signed ICFs are to be included in the investigator's files with the participant's study records.

11.2.14. Retention of Records

The Principal Investigator must maintain all documentation relating to the study for a period of at least 2 years after the last marketing application approval or, if not approved, 2 years following the discontinuance of the test article for investigation. If this requirement differs from any local regulations, the local regulations will take precedence unless the local retention policy is less than 2 years.

If it becomes necessary for Moderna or the regulatory authority to review any documentation relating to the study, the investigator must permit access to such records. No records will be destroyed without the written consent of the Sponsor, if applicable. It is the responsibility of the Sponsor to inform the investigator when these documents no longer need to be retained.

11.2.15. Study and Site Closure

If the study is prematurely terminated or suspended, the Sponsor shall promptly inform the investigators, the IECs/IRBs, the regulatory authorities, and any CRO(s) used in the study of the reason for termination or suspension, as specified by the applicable regulatory requirements. The investigator shall promptly inform the participant and should assure appropriate participant therapy and/or follow-up.

The Sponsor or designee reserves the right to close the study site or terminate the study at any time for any reason at the sole discretion of the Sponsor.

The investigator may initiate study-site closure at any time, provided there is reasonable cause and sufficient notice is given in advance of the intended termination.

Reasons for the early closure of a study site by the Sponsor or investigator may include but are not limited to:

- Continuation of the study represents a significant medical risk to participants.
- Failure of the investigator to comply with the protocol, the requirements of the IRB/IEC or local health authorities, the Sponsor's procedures, or GCP guidelines.
- Inadequate recruitment of participants by the investigator.
- Discontinuation of further mRNA-1273 development.

Study sites will be closed upon study completion. A study site is considered closed when all required documents and study supplies have been collected and a study-site closure visit has been performed.

11.2.16. Publication Policy

The results of this study may be published or presented at scientific meetings. If this is foreseen, the investigator agrees to submit all manuscripts or abstracts to the Sponsor before submission. This allows the Sponsor to protect proprietary information and to provide comments.

The Sponsor will comply with the requirements for publication of study results. In accordance with standard editorial and ethical practice, the Sponsor will generally support publication of multicenter studies only in their entirety and not as individual site data. In this case, a coordinating investigator will be designated by mutual agreement.

Authorship will be determined by mutual agreement and in line with International Committee of Medical Journal Editors authorship requirements.

The clinical study plan and the results of the study will be published on www.ClinicalTrials.gov in accordance with 21 CFR 50.25(c). The results of and data from this study belong to Moderna.

11.3. APPENDIX 3: Contraceptive Guidance

Woman of Childbearing Potential (WOCBP)

Females of childbearing potential are those who are considered fertile following menarche and until becoming postmenopausal unless permanently sterile (see below). If fertility is unclear (eg, amenorrhea in adolescents or athletes) and a menstrual cycle cannot be confirmed before first dose of study treatment, additional evaluation should be considered.

Women in the following categories are not considered WOCBP:

1. Premenarchal
2. Premenopausal, surgically sterile female with 1 of the following:
   a. Documented complete hysterectomy
   b. Documented bilateral salpingectomy
   c. Documented bilateral oophorectomy

For individuals with permanent infertility due to an alternate medical cause other than the above, (eg, mullerian agenesis, androgen insensitivity), investigator discretion should be applied in determining study entry.

Note: Documentation can come from the site personnel's: review of the participant's medical records, medical examination, or medical history interview.

3. Postmenopausal female

- A postmenopausal state is defined as no menses for 12 months without an alternative medical cause. The following age-specific requirements apply:
  - Women <50 years of age would be considered postmenopausal if they have been amenorrheic for 12 months or more following cessation of exogenous hormonal treatments and if they have luteinizing hormone and FSH levels in the postmenopausal range for the institution.
  - Women ≥ 50 years of age would be considered postmenopausal if they have been amenorrheic for 12 months or more, had radiation-induced menopause with last menses >1 year ago or had chemotherapy-induced menopause with last menses > 1 year ago.
- A high FSH level in the postmenopausal range may be used to confirm a postmenopausal state in women not using hormonal replacement therapy (HRT).
- Females on HRT and whose menopausal status is in doubt will be required to use one of the non-estrogen hormonal highly effective contraception methods if they wish to continue their HRT during the study. Otherwise, they must discontinue HRT to allow confirmation of postmenopausal status before study enrollment.

11.4. APPENDIX 4: Statistical Appendices

11.4.1. Estimands and Estimand Specifications

Table 23: Intercurrent Event Types

Label	Intercurrent Event Type	Comment
IcEv1 (death without confirmation of cases, ie, unrelated death)	Unrelated death without documented confirmed COVID-19	Participants in PP Set who die due to reasons unrelated to COVID-19 without confirmation of being a case will all be included in statistical analysis.
IcEv2 (early COVID-19)	COVID-19 starting up to 14 days after the second dose of IP	Participants in PP Set who experience early COVID-19 up to 14 days after the second dose of IP will all be included in statistical analysis.

Abbreviations: IcEv: intercurrent event; IP = investigational product; PP: per-protocol.

Table 24: Primary Objective and Estimands with Rationale for Strategies to Address Intercurrent Events for Per-Protocol Analysis

**Objective: To demonstrate the efficacy of mRNA-1273 to prevent COVID-19**	
**Estimand Description**	Vaccine efficacy will be measured using 1 – HR (mRNA-1273/Placebo) of COVID-19 from 14 days after second dose of IP in adults. A hypothetical strategy will be used for deaths unrelated to COVID-19, and a hypothetical strategy will also be used for early COVID-19 in participants in the PP Set.
**Target Population**	Adults aged 18 years and older in circumstances at a high risk of SARS-CoV-2 infection but without medical conditions that pose additional risk of developing severe disease.<br>The population excludes those previously infected or vaccinated for SARS-CoV-2 or with a medical condition, on treatment that poses additional risks (including those requiring immunosuppressants or immune-modifying drugs), or SARS-CoV-2 pre-positive.
**Variable/Endpoint**	Time to COVID-19 disease, censoring at early discontinuation, early COVID-19, or last assessment for an event not being observed, whichever comes earlier.
**Treatment Condition(s)**	**Test:** mRNA-1273<br>**Reference:** Placebo
**Estimand Label**	**Estimand 1**
**Population-Level Summary**	Vaccine efficacy defined as 1 - HR of mRNA-1273/Placebo
**Intercurrent Event Strategy**	
**IcEv1 (Unrelated death):**	Hypothetical
**IcEv2 (early infection):**	Hypothetical
**Rationale for Strategy(s)**	Hypothetical: unrelated death without confirmation of COVID-19 will be censored at the time of death as if there is no event, handled with independent censoring.<br>Hypothetical: early case in PP Set will be censored at the time of case onset if it is not a case, handled with independent censoring.

11.4.2. Statistical Methods and Sensitivity Analyses

Table 25: Summary of Statistical Methods and Sensitivity Analyses

Estimand Label	Estimand Description	Main Estimation			Sensitivity Analysis
		Analysis Set	Imputation/Data/Censoring Rules	Analysis Model/Method	
Estimand 1	See Table 24	PP	Participants who did not develop COVID-19, ongoing or complete the study will be censored at the last assessment.<br>Participants who discontinue early or die without COVID-19 will be censored at the date of discontinuation or death respectively.<br>Participants who is a confirmed case prior to 14 days after the second dose of IP will be censored on the date of confirmation.	The (1 - HR) with 95% CI will be estimated using Cox proportional hazard regression analysis with treatment arm as a fixed effect stratified by randomization stratification factors. And the null hypothesis will be tested using Wald Chi-square method.	As described in Section 9.5.1 with cases counted starting after the second dose of IP

11.5. APPENDIX 5: Protocol Amendment History

The document history table for this protocol and the Protocol Amendment Summary of Changes Table for the current Amendment 8 is located directly before the Table of Contents.

A description of Amendment 7, Amendment 6, Amendment 5, Amendment 4, Amendment 3, Amendment 2, and Amendment 1 is presented in this appendix.

Amendment 7, 10 Feb 2021

Main Rationale for the Amendment:

The purpose of this amendment is to collect safety information on suspected cases of anaphylaxis and to include participant history of facial injections or dermal fillers in the eDiary.

The summary of changes table provided here describes the major changes made in Amendment 7 relative to Amendment 6, including the sections modified and the corresponding rationales. The synopsis of Amendment 7 has been modified to correspond to changes in the body of the protocol.

Summary of Major Changes from Protocol Amendment 6 to Protocol Amendment 7:

Section # and Name	Description of Change	Brief Rationale
Title Page, Protocol Approval Page, Headers, Protocol Amendment Summary of Changes	Updated the protocol version and date.	To reflect the new version and date of the protocol.
Sections 1.1 (Synopsis), 6.2.2 (Administration of Investigational Product), 6.2.8.1 (Planned Unblinding)	Clarified that study site personnel who were blinded during the Blinded Phase will be unblinded **at the participant level** at the Participant Decision clinic visit.	To clarify that the site personnel is being unblinded only at the Participant Decision clinic visit.
Sections 1.1 (Synopsis), 4.1.2 (Part B, the Open-label Observational Phase)	Clarified that with regards to EUA eligibility in Part B, the CDC-EUA guidance specifications will supersede eligibility criteria in the protocol.	To clarify that the CDC-EUA guidance specifications supersede protocol specified criteria for EUA eligibility in Part B.
Section 4.1 (General Design)	Corrected the number of scheduled clinic visits and added mention of the Participant Decision Visit.	To clarify the number of scheduled visits.
Section 4.1.2 (Part B, the Open-Label Observational Phase)	Updated text to clarify requirements for Part B subjects who are unblinded and received only 1 dose of mRNA-1273 during Part A.	Clarification of requirements for Part B subjects who received only 1 dose of mRNA-1273 during Part A.

Section # and Name	Description of Change	Brief Rationale
Section 6.2.4 (Packaging and Labeling)	Updated to indicate that vaccine product is packaged in a 10R glass vial with a 6-dose, 5.0-mL or 10-dose, 6.3-mL fill volume.	To include IP presentation for additional supplies that may be used in Part B of the study.
Section 6.2.5 (Storage)	Updated to specify storage requirements for the 6-dose and 10-dose mRNA vaccine.	To include IP storage conditions for additional supplies that may be used in Part B of the study.
Sections 6.4.2 (Concomitant Medications and Therapies), 7.2 (Discontinuation of Study Treatment), 8.2.2 (Use of Electronic Diaries), 8.2.6 (Blood Sampling Volumes, Table 4)	Clarified requirements for Part A and Part B.	Clarification of concomitant medication, eDiary, and blood sampling procedures during Part A and Part B.
Section 8.2.2 (Use of Electronic Diaries), Appendix 1 (Schedule of Events, Table 17 footnote 7).	Clarified that eDiary prompts to surveil for weekly COVID-19 symptoms will be performed for all participants in Part A and Part B.<br><br>Added eDiary prompt to solicit collection of participant history of facial injections or dermal fillers.	Clarification.<br><br>To assure all concomitant medications are recorded.
Section 8.2.4 (Physical Examination)	Removed body mass index from the Participant Decision Visit.	Clarification.
Section 8.2.6 (Blood Sampling Volumes)	Updated maximum planned volume of blood collection for the complete study to include a range.	Clarification.
Section 8.3.2 (Medically Attended Adverse Events)	Added suspected cases of anaphylaxis as an MAAE and requirement to report as an SAE.	Sponsor collecting anaphylaxis information.
Section 9.4 (Analysis Populations, Table 8); Synopsis	Clarified Immunogenicity Subset	Clarification
Section 9.5.1.1 (Efficacy Analysis on Primary Endpoint), Appendix 4 (Statistical Appendices, Table 23 and Table 24)	Updated intercurrent events and estimands.	Clarification of estimand.
Section 9.5.1.3 (Long-term Efficacy Analysis); Synopsis	Updated case count parameters.	Provide long-term efficacy analysis strategy relevant to change in study design.

Section # and Name	Description of Change	Brief Rationale
Appendix 1 (Schedule of Events, Table 21 footnote 2, and Table 22 footnote 2)	Updated physical examination to symptom-directed physical examination and removed height and weight requirements from the Open-Label Clinic Visits.	Clarification

Amendment 6, 23 Dec 2020

Main Rationale for the Amendment:

The purpose of this amendment is to inform all ongoing study participants of the availability of and eligibility criteria of any COVID-19 vaccine made available under an EUA and to offer participants who originally received placebo in this study the potential benefit of vaccination against COVID-19, given that the primary efficacy endpoint for mRNA-1273 against COVID-19 was met per the protocol-defined IA.

The summary of changes table provided here describes the major changes made in Amendment 6 relative to Amendment 5, including the sections modified and the corresponding rationales. The synopsis of Amendment 6 has been modified to correspond to changes in the body of the protocol.

Summary of Major Changes from Protocol Amendment 5 to Protocol Amendment 6:

Section # and Name	Description of Change	Brief Rationale
Title Page, Protocol Approval Page, Headers, Protocol Amendment Summary of Changes	Updated the protocol version and date	To reflect the new version and date of the protocol
Sections 1.1 (Synopsis), 1.2 (Schema), 4.1.2 (Part B, the Open-Label Observational Phase), 6.2.8.1 (Planned Unblinding), 7.3.1 (Participant Withdrawal), and 11.1 (Schedules of Events)	Added a "Participant Decision clinic visit".	This visit provides the opportunity for study site personnel to discuss with and offer to participants, the choice to be unblinded, as well as offering to participants who originally received placebo, the choice to receive active vaccination with mRNA-1273 and possible vaccination against COVID-19. Participants will also sign a revised Informed Consent Form at this visit.

Section # and Name	Description of Change	Brief Rationale
Sections 1.1 (Synopsis), 1.2 (Schema), 4.1 (General Design), 4.2 (Scientific Rationale for Study), 6.2 (Method of Randomly Assigning Participants to Treatment Groups [Part A], Blinded Phase Only), and 11.1 (Schedules of Events)	Changes to study design that split the study into a Blinded Phase and an Open-Label Observational Phase.	These changes accommodate the breaking of the blind and offering of open-label vaccination to all participants. This change also distinguishes the Open-Label Supplemental Schedule of Events (Vaccination Phase) for participants who received placebo, and who meet EUA eligibility, and request to receive active vaccine.
Sections 1.1 (Synopsis), 1.2 (Schema), 4.1.2 (Part B, the Open-Label Observational Phase), and 11.1 (Schedules of Events)	Added a Supplemental SoE and a Modified Supplemental SoE	These provide paths for participants who choose to be unblinded and receive mRNA-1273, and for participants who previously received only 1 dose of blinded mRNA-1273 during the study (Blinded Phase), to transition into the Open-Label Phase of the study.
Section 2.2.2 (Clinical Studies)	Updated status of ongoing clinical studies, including this study (mRNA-1273-P301).	The status of the 3 clinical studies (one Phase 1, one Phase 2a, and this Phase 3 study) have changed since Amendment 5. In addition, the results of the interim analyses in this study of the primary efficacy endpoint (prevention of COVID-19 infection), a major secondary endpoint (prevention of severe COVID-19), and safety and reactogenicity endpoints are now available and are provided here. These results provide the justification for offering participants the opportunity to receive active IP (mRNA-1273) and the potential benefit of vaccination against COVID-19.

Section # and Name	Description of Change	Brief Rationale
Section 1.1 (Synopsis) and 9.5.1.3 (Long-term Efficacy Analysis)	Addition of long-term efficacy analyses	Allows for the analysis of the long-term efficacy of mRNA-1273 in the following treatment cohorts:<br>• mRNA-1273 Cohort: Participants randomized to mRNA-1273 in the Blinded Phase.<br>• Placebo Cohort: Participants randomized to Placebo in the Blinded Phase and did not crossover to mRNA-1273 in the Open-label Phase.<br>• Placebo-mRNA-1273 Cohort: Participants randomized to Placebo in the Blinded phase and crossed over to mRNA-1273 in the Open-label Phase.
Section 2.3.3 (Overall Benefit/Risk Conclusion)	Updated the Benefit/Risk Assessment	Based on the interim results from this pivotal Phase 3 study, mRNA-1273 prevents COVID-19 and severe COVID-19. The demonstrated clinical benefit of mRNA-1273 is supported by evidence of a robust immune response both in terms of binding antibodies (bAbs) and neutralizing antibodies (nAbs) as well as the induction of CD4+ T-cells with a Th-1 dominant phenotype.
Section 11.1	Added footnote to Table 16	To clarify that the eDiary Safety Prompts will be triggering off on Day 61 to take into consideration the (-3 day) window allowance on Day 29.

Amendment 5, 11 Nov 2020

Main Rationale for the Amendment:

The main purpose of this amendment is to clarify that the eDiary prompts for safety surveillance will be weekly and to add Month 19 safety call.

The summary of changes table provided here describes the major changes made in Amendment 5 relative to Amendment 4, including the sections modified and the corresponding rationales. The synopsis of Amendment 5 has been modified to correspond to changes in the body of the protocol.

Summary of Major Changes from Protocol Amendment 4 to Protocol Amendment 5:

Section # and Name	Description of Change	Brief Rationale
Title Page, Protocol Approval Page, Headers, Protocol Amendment Summary of Changes	Updated the protocol version and date	To reflect the new version and date of the protocol
Section 11.1	Updated Appendix 1 (Tables 16 and 17)	To clarify the weekly schedule for eDiary prompts through Year 1 and Year 2 of follow-up
Section 11.1	Added Month 19 Safety Call	To clarify that participants will have continuous weekly (eDiary prompts) and monthly (safety calls) through Year 1 and Year 2 of follow-up

Amendment 4, 30 Sep 2020

Main Rationale for the Amendment:

The main purpose of this amendment is to increase the upper limit for stratification of enrolled participants considered "at risk" at Screening to 50%.

The summary of changes table provided here describes the major changes made in Amendment 4 relative to Amendment 3, including the sections modified and the corresponding rationales. The synopsis of Amendment 4 has been modified to correspond to changes in the body of the protocol.

Summary of Major Changes from Protocol Amendment 3 to Protocol Amendment 4:

Section # and Name	Description of Change	Brief Rationale
Title Page, Protocol Approval Page, Headers, Protocol Amendment Summary of Changes	Updated the protocol version and date	To reflect the new version and date of the protocol
Section 6.2.1.1 (Stratification)	Increased the upper limit for stratification of enrolled participants considered “at risk” at Screening to up to 50%, from 40%.	To enhance the diversity of the study population by increasing the number of racial and ethnic minority participants in the study. as participants from these communities often have higher rates of comorbidities.
Section 9.5.1.1 (Efficacy Analysis on Primary Endpoint) and Section 11.4.1 (Estimands and Estimand Specifications)	Updated the description of an intercurrent event (unrelated death) and its strategy	To align with the description in the Statistical Analysis Plan.

Amendment 3, 20 Aug 2020

Main Rationale for the Amendment:

The main purpose of this amendment is to make changes to the protocol in response to feedback from CBER.

The summary of changes table provided here describes the major changes made in Amendment 3 relative to Amendment 2, including the sections modified and the corresponding rationales. Minor editorial or formatting changes are not included in this summary table. The synopsis of Amendment 3 has been modified to correspond to changes in the body of the protocol.

Summary of Major Changes from Protocol Amendment 2 to Protocol Amendment 3:

Section # and Name	Description of Change	Brief Rationale
Title Page, Protocol Approval Page, Headers, Protocol Amendment Summary of Changes	Updated the protocol version and date	To reflect the new version and date of the protocol
Section 5 (Study Population)	Added a sentence to describe the intent to enroll a representative sample of racial and ethnic minority participants in the study	To enhance the diversity of the study population
Section 5.2 (Exclusion Criteria)	Added clarification to exclusion criterion #11 to define the parameters based on screening CD4 count and viral load for exclusion of study participants	To clarify the definition of controlled HIV disease in the exclusion criterion such that only participants with well-controlled HIV disease are enrolled in the study

Section # and Name	Description of Change	Brief Rationale
Section 5.2 (Exclusion Criteria)	Removed “topical tacrolimus” from exclusion criterion #12	No evidence to support any systemic effect of topical tacrolimus to warrant excluding them
Section 6.2.1.1 (Stratification)	Added HIV infection to the risk factors at Screening	To stratify participants based on certain risk factors
Section 8.2.3 (Demographics/Medical History)	Added collection of risk factors for complications of COVID-19	To document the diagnosis of any risk factor for complications of COVID19 used for stratification
Section 9.3 (Sample Size Determination)	Removed redundant bullet	To remove redundancy in the assumptions listed
Section 9.5.2 (Safety Analyses)	Removed safety analysis by serostatus	No added value for this analysis. Other subgroup analyses may be specified in the SAP, as needed.
Section 9.5.5 (Subgroup Analyses)	Removed the categories (white, non-white) from the Race Variable	To allow for more refined Race categorization being collected in the eCRF
Appendix 11.3	Removed “cessation of exogenous hormonal therapy”	To allow post-menopausal women to take these medications if needed for the treatment of the symptoms of menopause
Appendix 11.3	Removed “using hormonal contraception” from postmenopausal female with high FSH levels	Not a standard of care for women for treatment of menopausal symptoms

Amendment 2, 31 Jul 2020:

Main Rationale for the Amendment:

The main purpose of this amendment is to provide more intensive surveillance of symptoms and severity of cases of COVID-19 after the first dose of investigational product.

The summary of changes table provided here describes the major changes made in Amendment 2 relative to Amendment 1, including the sections modified and the corresponding rationales. Minor editorial or formatting changes are not included in this summary table. The synopsis of Amendment 2 has been modified to correspond to changes in the body of the protocol.

Summary of Major Changes from Protocol Amendment 1 to Protocol Amendment 2:

Section # and Name	Description of Change	Brief Rationale
Title Page, Protocol Approval Page, Headers, Protocol Amendment Summary of Changes	Updated the protocol version and date	Reflect the new version and date of the protocol

Section # and Name	Description of Change	Brief Rationale
Section 4.1 (General Design), Section 7.2 (Discontinuation of Study Treatment), Section 8.1.1 (Efficacy Assessments Related to COVID-19 and SARS-CoV-2 Infection), Section 8.1.2 (Surveillance for COVID-19 Symptoms),Table 14 (Schedule of Events [Vaccination Phase, Day 1 – Day 57])	Added a Day 29 NP swab prior to Dose 2	Improve surveillance for asymptomatic infection prior to Dose 2 to assist in discriminating COVID-19 symptoms from solicited systemic reactions after vaccination
Section 4.1 (General Design), Table 15 (Schedule of Events [Surveillance Phase, Day 64 – Day 394])	Changed the clinic visit at Month 4 to a safety call	Improve participant safety and adherence to protocol
Section 4.1 (General Design), Section 8.1.2 (Surveillance for COVID-19 Symptoms), Table 14 Footnote 5 (Schedule of Events [Vaccination Phase, Day 1 – Day 57])	Clarified symptom duration (> 48 hours) to trigger NP swab collection and investigator judgement whether to obtain an NP swab in the 7 days following vaccination due to overlap of solicited systemic symptoms and COVID-19	Improve surveillance for cases of COVID-19
Section 4.1 (General Design), Section 8.1.3 (Convalescent Period Starting with the Illness Visit), Section 8.1.4 (Ancillary Supplies for Participant Use)	Removed mention of continuous biometric monitoring	Simplify data management and clinical operations
Section 5.1 (Inclusion Criteria), Section	Removed the inclusion criterion regarding male contraception	Requirement not generally applicable for a Phase 3 vaccine study
Section 5.2 (Exclusion Criteria), Section 6.4.3 (Concomitant Medications and Vaccines that May Lead to the Elimination of a Participant from Per-Protocol Analyses)	Clarified language around influenza vaccination	Make restrictions on influenza vaccination less restrictive than for other licensed vaccines relative to administration of investigational product
Section 5.2 (Exclusion Criteria)	Removed restriction on enrollment of participants with human immunodeficiency virus (HIV) infection	Participants on stable antiretroviral therapy are not excluded, which diversifies the participant group
Section 8.3.7 (Time Period and Frequency for Collecting AE and SAE Information), Section 8.3.10 (Reporting Adverse Events)	Added expedited reporting of confirmed COVID-19 cases	Improve surveillance for cases of COVID-19

Section # and Name	Description of Change	Brief Rationale
Section 8.4.2 (Data and Safety Monitoring Board)	Added an Oversight Group	Clarify responsibility for declaring early efficacy or for taking action to stop, pause, or continue the study
Section 8.4.2 (Data and Safety Monitoring Board)	Updated description of monitoring for potential harm, including details on case counting for the purpose of harm monitoring to align with a DSMB analysis plan	Clarify description for harm monitoring, based on FDA feedback

Amendment 1, 26 Jun 2020:

Main Rationale for the Amendment:

The main purpose of this amendment is to provide more intensive surveillance of symptoms and severity of cases of COVID-19 after the first dose of investigational product.

The summary of changes table provided here describes the major changes made in Amendment 1 relative to the original protocol, including the sections modified and the corresponding rationales. Minor editorial or formatting changes are not included in this summary table.

Summary of Major Changes in Protocol Amendment 1:

Section # and Name	Description of Change	Brief Rationale
Title page, Signature page, and header	Updated the protocol version and date	Reflect the new version and date of the protocol
Synopsis; Section 4.1, General Design; Section 8.1.3, Convalescent Period; Section 11.1, Schedules of Events, Table 17	Increased the frequency of telemedicine contacts during the Convalescent Period following the Initial Illness Visit	Improve the amount and quality of COVID-19 symptom data collected during the Convalescent Period
Synopsis; Section 4.1, General Design; Section 8.1.3, Convalescent Period; Section 8.1.4, Ancillary Supplies for Participant Use; Section 11.1, Schedules of Events, Table 17	Added monitoring of oxygen saturation to the Convalescent Period	Improve surveillance for incidence of COVID-19 during the study

Section # and Name	Description of Change	Brief Rationale
Synopsis; Section 8.1.3, Convalescent Period; Section 8.1.4, Ancillary Supplies for Participant Use; Section 11.1, Schedules of Events, Table 17	Increased the frequency of monitoring for SARS-CoV-2, using saliva as the preferred sample matrix after the Illness Visit during the Convalescent Period	Improve the sensitivity of monitoring the time course of viral shedding during COVID-19
Synopsis; Section 3, Objectives and Endpoints	Added a respiratory sample for hospitalized participants as a matrix for confirming the presence of SARS-CoV-2	Increase the potential number of evaluable COVID-19 cases
Synopsis; Section 3, Objectives and Endpoints	Broadened the definition for seroconversion at a participant level	Included neutralizing antibody (nAb) in addition to binding antibody (bAb)
Section 8, Study Assessments and Procedures; Section 8.1.3, Convalescent Period; Section 8.1.4, Ancillary Supplies for Participant Use; Section 8.2.2, Use of Electronic Diaries; Section 11.1, Schedules of Events, Table 17	Eliminated paper diaries, substituting an instruction card listing symptoms and a severity grading system to enhance the quality of data obtained by telemedicine contacts	Reduce fomite transmission of SARS-CoV-2 and increase frequency of investigative staff interactions with participants during the Convalescent Period
Section 8.1.2, Surveillance for COVID-19 Symptoms	Decreased the number of symptoms (to one of the 11 CDC symptoms) that would result in an Illness Visit	Increase the likelihood of capturing all COVID-19 cases in the earliest stage of disease
Section 8.2.2, Use of Electronic Diaries	Expanded the scope of eDiary prompts and data collected during the Surveillance Phase	Increase the likelihood of capturing all COVID-19 cases in the earliest stage of disease

Signature Page for VV-CLIN-002035 v3.0

Approval	Allison August<br>Medical<br>29-Mar-2021 14:17:57 GMT+0000

Signature Page for VV-CLIN-002035 v3.0

CLINICAL STUDY PROTOCOL
Summary of Changes

**Protocol Title:**	A Phase 3, Randomized, Stratified, Observer-Blind, Placebo-Controlled Study to Evaluate the Efficacy, Safety, and Immunogenicity of mRNA-1273 SARS-CoV-2 Vaccine in Adults Aged 18 Years and Older
**Protocol Number:**	mRNA-1273-P301
**Sponsor Name:**	ModernaTX, Inc.
**Legal Registered Address:**	200 Technology Square<br>Cambridge, MA 02139
**Sponsor Contact and Medical Monitor:**	Allison August, MD<br>VP, Clinical Development<br>ModernaTX, Inc.<br>200 Technology Square, Cambridge, MA 02139<br>Telephone: 1-617-678-8937<br>e-mail: Allison.August@modernatx.com
**Regulatory Agency Identifier Number(s):**	IND: 19745
**Amendment Number:**	8
**Date of Amendment 8:**	23 Mar 2021
**Date of Amendment 7:**	10 Feb 2021
**Date of Amendment 6:**	23 Dec 2020
**Date of Amendment 5:**	11 Nov 2020
**Date of Amendment 4:**	30 Sep 2020
**Date of Amendment 3:**	20 Aug 2020
**Date of Amendment 2:**	31 Jul 2020
**Date of Amendment 1:**	26 Jun 2020
**Date of Original Protocol:**	15 Jun 2020

Protocol Amendment Summary of Changes

DOCUMENT HISTORY	
**Document**	**Date**
Amendment 8	23 Mar 2021
Amendment 7	10 Feb 2021
Amendment 6	23 Dec 2020
Amendment 5	11 Nov 2020
Amendment 4	30 Sep 2020
Amendment 3	20 Aug 2020
Amendment 2	31 Jul 2020
Amendment 1	26 Jun 2020
Original Protocol	15 Jun 2020

Amendment 8, 23 Mar 2021

Main Rationale for the Amendment:

The purpose of this amendment is to update language around unblinding and open-label dosing inthe context of the majority of participants already having completed their blinded follow-up. In addition, the amendment updates the reporting of serious COVID-19 cases.

The summary of changes table provided here describes the major changes made in Amendment 8relative to Amendment 7, including the sections modified and the corresponding rationales. As applicable, the synopsis of Amendment 8 has been modified to correspond to changes in the bodyof the protocol.

Summary of Major Changes from Protocol Amendment 7 to Protocol Amendment 8:

Section # and Name	Description of Change	Brief Rationale
Section 4.1.2 (Part B, the Open-label Observational Phase)	States that, due to ethical and scientific concerns, all participants will be unblinded once investigational product is no longer available. In addition, the text indicates that if participants want to receive emergency use authorization (EUA) vaccine outside of the study, participants will need to be withdrawn from the study.<br>The amendment also provides directions for unblinding after Biologics License Application	At the time of this amendment, more than 95% of the enrolled participants have completed blinded follow-up, either through being unblinded or study discontinuation. Therefore, there is no scientific value for participants to remain blinded.<br>In addition, non-study EUA vaccines are widely available, so ethically, participants shouldbe unblinded to ensure they can make an informed choice if they

	(BLA) database lock.	wish to receive EUA COVID-19 vaccine outside of the study.
Section 8.3.7 (Time Period and Frequency for collecting Adverse Events and Serious Adverse Event Information) and Section 8.3.10 (Reporting Adverse Events)	Reporting of only serious COVID-19 cases within 24 hours to sponsor or it's designee.	Non-serious COVID-19 cases are an efficacy endpoint, so reporting to Pharmacovigilance is no longer warranted.

Amendment 7, 10 Feb 2021

Main Rationale for the Amendment:

The purpose of this amendment is to collect safety information on suspected cases of anaphylaxisand to include participant history of facial injections or dermal fillers in the eDiary.

The summary of changes table provided here describes the major changes made in Amendment 7 relative to Amendment 6, including the sections modified and the corresponding rationales. The synopsis of Amendment 7 has been modified to correspond to changes in the body of the protocol.

Summary of Major Changes from Protocol Amendment 6 to Protocol Amendment 7:

Section # and Name	Description of Change	Brief Rationale
Title Page, Protocol Approval Page, Headers, Protocol Amendment Summary of Changes	Updated the protocol version and date.	To reflect the new version and date of the protocol.
Sections 1.1 (Synopsis), 6.2.2 (Administration of Investigational Product), 6.2.8.1 (Planned Unblinding)	Clarified that study site personnel who were blinded during the Blinded Phase will be unblinded **at the participant level** at the Participant Decision clinic visit.	To clarify that the site personnel is being unblinded only at the Participant Decision clinic visit.
Sections 1.1 (Synopsis), 4.1.2 (Part B, the Open-label Observational Phase)	Clarified that with regards to EUA eligibility in Part B, the CDC-EUA guidance specifications will supersede eligibility criteria in the protocol.	To clarify that the CDC-EUA guidance specifications supersede protocol specified criteria for EUA eligibility in Part B.
Section 4.1 (General Design)	Corrected the number of scheduled clinic visits and added mention of the Participant Decision Visit.	To clarify the number of scheduled visits.
Section 4.1.2 (Part B, the Open-Label Observational Phase)	Updated text to clarify requirements for Part B subjects who are unblinded and received only 1 dose of mRNA-1273 during Part A.	Clarification of requirements for Part B subjects who received only 1 dose of mRNA-1273 during Part A.

Section 6.2.4 (Packaging and Labeling)	Updated to indicate that vaccine product is packaged in a 10R glass vial with a 6-dose, 5.0-mL or 10-dose, 6.3-mL fill volume.	To include IP presentation for additional supplies that may be usedin Part B of the study.
Section 6.2.5 (Storage)	Updated to specify storage requirements for the 6-dose and 10-dose mRNA vaccine.	To include IP storage conditions foradditional supplies that may be usedin Part B of the study.
Sections 6.4.2 (Concomitant Medications and Therapies), 7.2 (Discontinuation of Study Treatment), 8.2.2 (Use of Electronic Diaries), 8.2.6 (Blood Sampling Volumes, Table 4)	Clarified requirements for Part Aand Part B.	Clarification of concomitant medication, eDiary, and blood sampling procedures during Part Aand Part B.
Section 8.2.2 (Use of Electronic Diaries), Appendix 1 (Schedule of Events, Table 17 footnote 7).	Clarified that eDiary prompts to surveil for weekly COVID-19 symptoms will be performed for all participants in Part A and Part B.<br>Added eDiary prompt to solicit collection of participant history offacial injections or dermal fillers.	Clarification.<br>To assure all concomitant medications are recorded.
Section 8.2.4 (Physical Examination)	Removed body mass index fromthe Participant Decision Visit.	Clarification.
Section 8.2.6 (Blood Sampling Volumes)	Updated maximum planned volume of blood collection for thecomplete study to include a range.	Clarification.
Section 8.3.2 (Medically Attended Adverse Events)	Added suspected cases of anaphylaxis as an MAAE and requirement to report as an SAE.	Sponsor collecting anaphylaxis information.
Section 9.4 (Analysis Populations, Table 8); Synopsis	Clarified Immunogenicity Subset	Clarification

Section 9.5.1.1 (Efficacy Analysis on Primary Endpoint), Appendix 4 (Statistical Appendices, Table 23 and Table 24)	Updated intercurrent events and estimands.	Clarification of estimand.
Section 9.5.1.3 (Long-term Efficacy Analysis); Synopsis	Updated case count parameters.	Provide long-term efficacy analysisstrategy relevant to change in studydesign.
Appendix 1 (Schedule of Events, Table 21 footnote 2, and Table 22 footnote 2)	Updated physical examination to symptom-directed physical examination and removed height and weight requirements from theOpen-Label Clinic Visits.	Clarification

Amendment 6, 23 Dec 2020

Main Rationale for the Amendment:

The purpose of this amendment is to inform all ongoing study participants of the availability of and eligibility criteria of any COVID-19 vaccine made available under an EUA and to offer participants who originally received placebo in this study the potential benefit of vaccination against COVID-19, given that the primary efficacy endpoint for mRNA-1273 against COVID-19was met per the protocol-defined IA.

The summary of changes table provided here describes the major changes made in Amendment 6 relative to Amendment 5, including the sections modified and the corresponding rationales. The synopsis of Amendment 6 has been modified to correspond to changes in the body of the protocol.

Summary of Major Changes from Protocol Amendment 5 to Protocol Amendment 6:

Section # and Name	Description of Change	Brief Rationale
Title Page, Protocol Approval Page, Headers, Protocol Amendment Summary of Changes	Updated the protocol version and date	To reflect the new version and date of the protocol
Sections 1.1 (Synopsis), 1.2 (Schema), 4.1.2 (Part B, the Open-Label Observational Phase), 6.2.8.1 (Planned Unblinding), 7.3.1 (Participant Withdrawal), and 11.1 (Schedules of Events)	Added a "Participant Decision clinic visit".	This visit provides the opportunity for study site personnel to discuss with and offer to participants, the choice to be unblinded, as well as offering to participants who originally received placebo, the choice to receive active vaccination with mRNA-1273 and possible vaccination against COVID-19. Participants will also sign a revised Informed Consent Form at this visit.

Section # and Name	Description of Change	Brief Rationale
Sections 1.1 (Synopsis), 1.2 (Schema), 4.1 (General Design), 4.2 (Scientific Rationale for Study), 6.2 (Method of Randomly Assigning Participants to Treatment Groups [Part A], Blinded Phase Only), and 11.1 (Schedules of Events)	Changes to study design that split the study into a Blinded Phase and an Open-Label Observational Phase.	These changes accommodate the breaking of the blind and offering of open-label vaccination to all participants. This change also distinguishes the Open-Label Supplemental Schedule of Events (Vaccination Phase) for participants who received placebo, and who meet EUA eligibility, and request to receive active vaccine.
Sections 1.1 (Synopsis), 1.2 (Schema), 4.1.2 (Part B, the Open-Label Observational Phase), and 11.1 (Schedules of Events)	Added a Supplemental SoE and a Modified Supplemental SoE	These provide paths for participants who choose to be unblinded and receive mRNA-1273, and for participants who previously received only 1 dose of blinded mRNA-1273 during the study (Blinded Phase), to transition into the Open-Label Phase of the study.
Section 2.2.2 (Clinical Studies)	Updated status of ongoing clinical studies, including this study (mRNA-1273-P301).	The status of the 3 clinical studies (one Phase 1, one Phase 2a, and this Phase 3 study) have changed since Amendment 5. In addition, the results of the interim analyses in this study of the primary efficacy endpoint (prevention of COVID-19 infection), a major secondary endpoint (prevention of severe COVID-19), and safety and reactogenicity endpoints are now available and are provided here. These results provide the justification for offering participants the opportunity to receive active IP (mRNA-1273) and the potential benefit of vaccination against COVID-19.

Section # and Name	Description of Change	Brief Rationale
Section 1.1 (Synopsis) and 9.5.1.3 (Long-term Efficacy Analysis)	Addition of long-term efficacy analyses	Allows for the analysis of the long-term efficacy of mRNA-1273 in the following treatment cohorts:<br>• mRNA-1273 Cohort: Participants randomized to mRNA-1273 in the Blinded Phase.<br>• Placebo Cohort: Participants randomized to Placebo in the Blinded Phase and did not crossover to mRNA-1273 in the Open-label Phase.<br>• Placebo-mRNA-1273 Cohort: Participants randomized to Placebo in the Blinded phase and crossed over to mRNA-1273 in the Open-label Phase.
Section 2.3.3 (Overall Benefit/Risk Conclusion)	Updated the Benefit/Risk Assessment	Based on the interim results from this pivotal Phase 3 study, mRNA-1273 prevents COVID-19 and severe COVID-19. The demonstrated clinical benefit of mRNA-1273 is supported by evidence of a robust immune response both in terms of binding antibodies (bAbs) and neutralizing antibodies (nAbs) as well as the induction of CD4+ T-cells with a Th-1 dominant phenotype.
Section 11.1	Added footnote to Table 16	To clarify that the eDiary Safety Prompts will be triggering off on Day 61 to take into consideration the (-3 day) window allowance on Day 29.

Amendment 5, 11 Nov 2020

Main Rationale for the Amendment:

The main purpose of this amendment is to clarify that the eDiary prompts for safety surveillance will be weekly and to add Month 19 safety call.

The summary of changes table provided here describes the major changes made in Amendment 5 relative to Amendment 4, including the sections modified and the corresponding rationales. The synopsis of Amendment 5 has been modified to correspond to changes in the body of the protocol.

Summary of Major Changes from Protocol Amendment 4 to Protocol Amendment 5:

**Section # and Name**	**Description of Change**	**Brief Rationale**
Title Page, Protocol Approval Page, Headers, Protocol Amendment Summary of Changes	Updated the protocol version and date	To reflect the new version and date of the protocol
Section 11.1	Updated Appendix 1 (Tables 16 and 17)	To clarify the weekly schedule for eDiary prompts through Year 1 and Year 2 of follow-up
Section 11.1	Added Month 19 Safety Call	To clarify that participants will have continuous weekly (eDiary prompts) and monthly (safety calls) through Year 1 and Year 2 of follow-up

Amendment 4, 30 Sep 2020

Main Rationale for the Amendment:

The main purpose of this amendment is to increase the upper limit for stratification of enrolled participants considered "at risk" at Screening to 50%.

The summary of changes table provided here describes the major changes made in Amendment 4 relative to Amendment 3, including the sections modified and the corresponding rationales. The synopsis of Amendment 4 has been modified to correspond to changes in the body of the protocol.

Summary of Major Changes from Protocol Amendment 3 to Protocol Amendment 4:

Section # and Name	Description of Change	Brief Rationale
Title Page, Protocol Approval Page, Headers, Protocol Amendment Summary of Changes	Updated the protocol version and date	To reflect the new version and date of the protocol
Section 6.2.1.1 (Stratification)	Increased the upper limit for stratification of enrolled participants considered "at risk" at Screening to up to 50%, from 40%.	To enhance the diversity of the study population by increasing the number of racial and ethnic minority participants in the study. as participants from these communities often have higher rates of comorbidities.
Section 9.5.1.1 (Efficacy Analysis on Primary Endpoint) and Section 11.4.1 (Estimands and Estimand Specifications)	Updated the description of an intercurrent event (unrelated death) and its strategy	To align with the description in the Statistical Analysis Plan.

Amendment 3, 20 Aug 2020

Main Rationale for the Amendment:

The main purpose of this amendment is to make changes to the protocol in response to feedback from CBER.

The summary of changes table provided here describes the major changes made in Amendment 3 relative to Amendment 2, including the sections modified and the corresponding rationales. Minor editorial or formatting changes are not included in this summary table. The synopsis of Amendment 3 has been modified to correspond to changes in the body of the protocol.

Summary of Major Changes from Protocol Amendment 2 to Protocol Amendment 3:

Section # and Name	Description of Change	Brief Rationale
Title Page, Protocol Approval Page, Headers, Protocol Amendment Summary of Changes	Updated the protocol version and date	To reflect the new version and date of the protocol
Section 5 (Study Population)	Added a sentence to describe the intent to enroll a representative sample of racial and ethnic minority participants in the study	To enhance the diversity of the study population
Section 5.2 (Exclusion Criteria)	Added clarification to exclusion criterion #11 to define the parameters based on screening CD4 count and viral load for exclusion of study participants	To clarify the definition of controlled HIV disease in the exclusion criterion such that only participants with well-controlled HIV disease are enrolled in the study
Section 5.2 (Exclusion Criteria)	Removed “topical tacrolimus” from exclusion criterion #12	No evidence to support any systemic effect of topical tacrolimus to warrant excluding them
Section 6.2.1.1 (Stratification)	Added HIV infection to the risk factors at Screening	To stratify participants based on certain risk factors
Section 8.2.3 (Demographics/Medical History)	Added collection of risk factors for complications of COVID-19	To document the diagnosis of any risk factor for complications of COVID19 used for stratification
Section 9.3 (Sample Size Determination)	Removed redundant bullet	To remove redundancy in the assumptions listed
Section 9.5.2 (Safety Analyses)	Removed safety analysis by serostatus	No added value for this analysis. Other subgroup analyses may be specified in the SAP, as needed.
Section 9.5.5 (Subgroup Analyses)	Removed the categories (white, non-white) from the Race Variable	To allow for more refined Race categorization being collected in the eCRF

Section # and Name	Description of Change	Brief Rationale
Appendix 11.3	Removed “cessation of exogenous hormonal therapy”	To allow post-menopausal women to take these medications if needed for the treatment of the symptoms of menopause
Appendix 11.3	Removed “using hormonal contraception” from postmenopausal female with high FSH levels	Not a standard of care for women for treatment of menopausal symptoms

Amendment 2, 31 Jul 2020:

Main Rationale for the Amendment:

The main purpose of this amendment is to provide more intensive surveillance of symptoms and severity of cases of COVID-19 after the first dose of investigational product.

The summary of changes table provided here describes the major changes made in Amendment 2 relative to Amendment 1, including the sections modified and the corresponding rationales. Minor editorial or formatting changes are not included in this summary table. The synopsis of Amendment 2 has been modified to correspond to changes in the body of the protocol.

Summary of Major Changes from Protocol Amendment 1 to Protocol Amendment 2:

Section # and Name	Description of Change	Brief Rationale
Title Page, Protocol Approval Page, Headers, Protocol Amendment Summary of Changes	Updated the protocol version and date	Reflect the new version and date of the protocol
Section 4.1 (General Design), Section 7.2 (Discontinuation of Study Treatment), Section 8.1.1 (Efficacy Assessments Related to COVID-19 and SARS-CoV-2 Infection), Section 8.1.2 (Surveillance for COVID-19 Symptoms),Table 14 (Schedule of Events [Vaccination Phase, Day 1 – Day 57])	Added a Day 29 NP swab prior to Dose 2	Improve surveillance for asymptomatic infection prior to Dose 2 to assist in discriminating COVID-19 symptoms from solicited systemic reactions after vaccination
Section 4.1 (General Design), Table 15 (Schedule of Events [Surveillance Phase, Day 64 – Day 394])	Changed the clinic visit at Month 4 to a safety call	Improve participant safety and adherence to protocol
Section 4.1 (General Design), Section 8.1.2 (Surveillance for COVID-19 Symptoms), Table 14 Footnote 5 (Schedule of Events [Vaccination Phase, Day 1 – Day 57])	Clarified symptom duration (> 48 hours) to trigger nasopharyngeal (NP) swab collection and investigator judgement whether to obtain an NP swab in the 7 days following vaccination due to overlap of solicited systemic symptoms and COVID-19	Improve surveillance for cases of COVID-19
Section 4.1 (General Design), Section 8.1.3 (Convalescent Period Starting with the Illness Visit), Section 8.1.4 (Ancillary Supplies for Participant Use)	Removed mention of continuous biometric monitoring	Simplify data management and clinical operations

Section # and Name	Description of Change	Brief Rationale
Section 5.1 (Inclusion Criteria), Section	Removed the inclusion criterion regarding male contraception	Requirement not generally applicable for a Phase 3 vaccine study
Section 5.2 (Exclusion Criteria), Section 6.4.3 (Concomitant Medications and Vaccines that May Lead to the Elimination of a Participant from Per-Protocol Analyses)	Clarified language around influenza vaccination	Make restrictions on influenza vaccination less restrictive than for other licensed vaccines relative to administration of investigational product
Section 5.2 (Exclusion Criteria)	Removed restriction on enrollment of participants with human immunodeficiency virus (HIV) infection	Participants on stable antiretroviral therapy are not excluded, which diversifies the participant group
Section 8.3.7 (Time Period and Frequency for Collecting AE and SAE Information), Section 8.3.10 (Reporting Adverse Events)	Added expedited reporting of confirmed COVID-19 cases	Improve surveillance for cases of COVID-19
Section 8.4.2 (Data and Safety Monitoring Board)	Added an Oversight Group	Clarify responsibility for declaring early efficacy or for taking action to stop, pause, or continue the study
Section 8.4.2 (Data and Safety Monitoring Board)	Updated description of monitoring for potential harm, including details on case counting for the purpose of harm monitoring to align with a DSMB analysis plan	Clarify description for harm monitoring, based on FDA feedback

Amendment 1, 26 Jun 2020:

Main Rationale for the Amendment:

The main purpose of this amendment is to provide more intensive surveillance of symptoms and severity of cases of COVID-19 after the first dose of investigational product.

The summary of changes table provided here describes the major changes made in Amendment 1 relative to the original protocol, including the sections modified and the corresponding rationales. Minor editorial or formatting changes are not included in this summary table.

Summary of Major Changes in Protocol Amendment 1:

Section # and Name	Description of Change	Brief Rationale
Title page, Signature page, and header	Updated the protocol version and date	Reflect the new version and date of the protocol
Synopsis; Section 4.1, General Design; Section 8.1.3, Convalescent Period; Section 11.1, Schedules of Events, Table 17	Increased the frequency of telemedicine contacts during the Convalescent Period following the Initial Illness Visit	Improve the amount and quality of COVID-19 symptom data collected during the Convalescent Period
Synopsis; Section 4.1, General Design; Section 8.1.3, Convalescent Period; Section 8.1.4, Ancillary Supplies for Participant Use; Section 11.1, Schedules of Events, Table 17	Added monitoring of oxygen saturation to the Convalescent Period	Improve surveillance for incidence of COVID-19 during the study
Synopsis; Section 8.1.3, Convalescent Period; Section 8.1.4, Ancillary Supplies for Participant Use; Section 11.1, Schedules of Events, Table 17	Increased the frequency of monitoring for SARS-CoV-2, using saliva as the preferred sample matrix after the Illness Visit during the Convalescent Period	Improve the sensitivity of monitoring the time course of viral shedding during COVID-19
Synopsis; Section 3, Objectives and Endpoints	Added a respiratory sample for hospitalized participants as a matrix for confirming the presence of SARS-CoV-2	Increase the potential number of evaluable COVID-19 cases
Synopsis; Section 3, Objectives and Endpoints	Broadened the definition for seroconversion at a participant level	Included neutralizing antibody (nAb) in addition to binding antibody (bAb)

Section # and Name	Description of Change	Brief Rationale
Section 8, Study Assessments and Procedures; Section 8.1.3, Convalescent Period; Section 8.1.4, Ancillary Supplies for Participant Use; Section 8.2.2, Use of Electronic Diaries; Section 11.1, Schedules of Events, Table 17	Eliminated paper diaries, substituting an instruction card listing symptoms and a severity grading system to enhance the quality of data obtained by telemedicine contacts	Reduce fomite transmission of SARS-CoV-2 and increase frequency of investigative staff interactions with participants during the Convalescent Period
Section 8.1.2, Surveillance for COVID-19 Symptoms	Decreased the number of symptoms (to one of the 11 CDC symptoms) that would result in an Illness Visit	Increase the likelihood of capturing all COVID-19 cases in the earliest stage of disease
Section 8.2.2, Use of Electronic Diaries	Expanded the scope of eDiary prompts and data collected during the Surveillance Phase	Increase the likelihood of capturing all COVID-19 cases in the earliest stage of disease

ModernaTX, Inc.

Protocol mRNA-1273-P301

A Phase 3, Randomized, Stratified, Observer-Blind, Placebo-Controlled Study to Evaluate the Efficacy, Safety, and Immunogenicity of mRNA-1273 SARS-CoV-2 Vaccine in Adults Aged 18 Years and Older

Statistical Analysis Plan

SAP Version 1.0

Version Date of SAP: 10 September 2020

Prepared by:

PPD
3575 Quakerbridge Road
Suite 201
Hamilton, NJ 08619

TABLE OF CONTENTS

LIST OF ABBREVIATIONS .... **VI**

1. INTRODUCTION **9**

2. STUDY OBJECTIVES **10**

2.1. PRIMARY OBJECTIVES 10
2.1.1. Primary Efficacy Objective 10
2.1.2. Primary Safety Objective 10
2.2. SECONDARY OBJECTIVES 10
2.2.1. Secondary Efficacy Objectives 10
2.2.2. Secondary Immunogenicity Objective 11
2.3. EXPLORATORY OBJECTIVES 11

3. STUDY ENDPOINTS **12**

3.1. PRIMARY ENDPOINTS 12
3.1.1. Primary Efficacy Endpoint 12
3.1.2. Primary Safety Endpoint 12
3.2. SECONDARY ENDPOINTS 13
3.2.1. Secondary Efficacy Endpoints 13
3.2.2. Secondary Immunogenicity Endpoints 14
3.3. EXPLORATORY ENDPOINTS 14

4. STUDY DESIGN **15**

4.1. OVERALL STUDY DESIGN 15
4.2. STATISTICAL HYPOTHESIS 18
4.3. SAMPLE SIZE AND POWER 18
4.4. RANDOMIZATION 19
4.5. BLINDING AND UNBLINDING 20

5. ANALYSIS POPULATIONS **21**

5.1. RANDOMIZATION SET 21
5.2. FULL ANALYSIS SET 21
5.3. MODIFIED INTENT-TO-TREAT SET 21
5.4. PER-PROTOCOL SET 21
5.5. IMMUNOGENICITY SUBSET 22
5.6. SOLICITED SAFETY SET 22
5.7. SAFETY SET 23

6. STATISTICAL ANALYSIS **23**

6.1. GENERAL CONSIDERATIONS 23
6.2. BACKGROUND CHARACTERISTICS 27

6.2.1. Subject Disposition 27
6.2.2. Demographics and Baseline Characteristics 29
6.2.3. Medical History 29
6.2.4. Prior and Concomitant Medications 30
6.2.5. Study Exposure 31
6.2.6. Major Protocol Deviations 31
6.2.7. COVID-19 Impact 31
6.3. EFFICACY ANALYSIS 31
6.3.1. Analysis of Primary Efficacy Endpoint 32
6.3.1.1. Primary Efficacy Endpoint Definition/Derivation 32
6.3.1.2. Primary Analysis Approach 34
6.3.1.3. Sensitivity Analyses with COVID-19 Cases Counted Starting at Various Timepoints 36
6.3.1.4. Subgroup Analysis 38
6.3.1.5. Supportive Analysis Using Exact Method Based on Incidence Rate 39
6.3.2. Analysis of Secondary Efficacy Endpoints 40
6.3.2.1. Derivation of Severe COVID-19 40
6.3.2.2. Derivation of Secondary Definition of COVID-19 40
6.3.2.3. Derivation of Serologically Confirmed SARS-CoV-2 Infection or COVID-19 Regardless of Symptomatology or Severity 41
6.3.2.4. Derivation of Asymptomatic SARS-CoV-2 Infection 44
6.3.2.5. Analysis of Secondary Efficacy Endpoints 44
6.4. INTERIM ANALYSES 46
6.5. MULTIPLICITY ADJUSTMENTS 48
6.6. SAFETY ANALYSIS 52
6.6.1. Unsolicited Treatment-emergent Adverse Events 52
6.6.1.1. Overview of Unsolicited TEAEs 53
6.6.1.2. TEAEs by System Organ Class and Preferred Term 54
6.6.1.3. TEAEs by Preferred Term 54
6.6.1.4. TEAEs by Toxicity Grade 55
6.6.1.5. Subgroup Analysis of TEAEs 55
6.6.2. Solicited Adverse Reactions 55
6.6.3. Vital Sign Measurements 57
6.7. IMMUNOGENICITY ANALYSIS 57
6.7.1. Immunogenicity Data 58
6.7.2. Summary of Antibody-Mediated Immunogenicity Endpoints 58
6.7.3. Analysis of Antibody-Mediated Immunogenicity Endpoints 60
6.8. EXPLORATORY ANALYSIS OF EFFICACY 61
6.8.1. Vaccine Efficacy Against Burden of Disease 61
6.8.2. Vaccine Efficacy Against Burden of Infection 63
6.8.3. Vaccine Efficacy on Duration and Presence/Severity of COVID-19 Symptoms 64

6.8.4. Vaccine Efficacy Against COVID-19 Over Time Based on Instantaneous Hazard Ratio ... 65
6.8.5. Durability of Vaccine Efficacy Against COVID-19 Using Covariate Adjustment Method Based on Cumulative Incidence ... 65
6.9.1. Protocol Safety Review Team ... 68
6.9.2. Data Safety Monitoring Board (DSMB) ... 68
6.9.2.1. Potential Vaccine Harm ... 69

7. CHANGES FROM PLANNED ANALYSES IN PROTOCOL ... 69

8. REFERENCES ... 69

9. LIST OF APPENDICES ... 71

9.1. Appendix A Standards for Variable Display in TFLs ... 71
9.2. Appendix B Analysis Visit Windows ... 72
9.3. Appendix C Imputation Rules for Missing Dates of Prior/Concomitant Medications and Non-Study Vaccinations ... 74
9.4. Appendix D Imputation Rules for Missing Dates of AEs ... 75
9.5. Appendix E Schedule of Events ... 76
9.6. Appendix F Estimands and Estimand Specifications ... 77

LIST OF TABLES

Table 1 Derivation for COVID-19 (primary efficacy endpoint) ... 33

Table 2 Censoring Rules for COVID-19 ... 37

Table 3: Interim Boundaries Using O'Brien-Fleming Spending function, Calculation Based on the PP Set for the Primary Efficacy Endpoint ... 48

Table 4 Burden of Disease Score ... 61

Table 5 Burden of Infection Score ... 63

Table 6 Grading of COVID-19 Symptoms ... 64

Table 7 Analysis Visit Windows ... 72

Table 8 Prior, Concomitant, and Post Categorization of Medications and Non-study Vaccinations ... 75

Table 9: Intercurrent Event Types ... 77

Table 10: Primary Objective and Estimands with Rationale for Strategies to Address Intercurrent Events for Per Protocol Analysis ... 78

List of Abbreviations

Abbreviation	Definition
AE	adverse event
ANCOVA	analysis of covariance
AR	adverse reaction
ARDS	acute respiratory distress syndrome
bAb	binding antibody
BARDA	Biomedical Advanced Research and Development Authority
BMI	body mass index
BOD	burden of disease
BOI	burden of infection
CDC	Centers for Disease Control and Prevention
CI	confidence interval
cLDA	constrained Longitudinal Data Analysis
COVID-19	coronavirus disease 2019
CRO	contract research organization
CSP	clinical study protocol
DHHS	Department of Health and Human Services
ECMO	extracorporeal membrane oxygenation
eCRF	electronic case report form
eDiary	electronic diary
ELISA	enzyme-linked immunosorbent assay
FAS	full analysis set
GM	geometric mean
GMFR	geometric mean fold rise
GMR	geometric mean ratio
GMT	geometric mean titer

Abbreviation	Definition
HR	hazard ratio
ICH	International Council for Harmonisation
IP	investigational product
IRT	interactive response technology
LOD	limit of detection
LLOQ	lower limit of quantification
MAAEs	medically-attended adverse events
MedDRA	Medical Dictionary for Regulatory Activities
mRNA	messenger ribonucleic acid
nAb	neutralizing antibody
NIAID	National Institute of Allergy and Infectious Diseases
NP	nasopharyngeal
PH	proportional hazard
PP	per-protocol
PT	preferred term
REML	restricted (or residual, or reduced) maximum likelihood
RT-PCR	reverse transcription polymerase chain reaction
SAE	serious adverse event
SAP	statistical analysis plan
SARS-CoV-2	severe acute respiratory syndrome coronavirus 2
SAS	Statistical Analysis System
SD	standard deviation
SOC	system organ class
TEAE	treatment-emergent adverse event
ULOQ	upper limit of quantification
VE	vaccine efficacy
WHO	World Health Organization

Abbreviation	Definition
WHODD	World Health Organization drug dictionary

1. Introduction

This statistical analysis plan (SAP), which describes the planned analyses for Study mRNA-1273-P301, is based on the most recent clinical study protocol (CSP) Amendment 3, dated 20-August-2020. The most recent approved electronic case report form (eCRF) Version 2.026, dated 21-AUG-2020.

In addition to the information presented in the statistical analysis plan section of the protocol (Section 9) which provides the principal features of analyses for this study, this SAP provides statistical analysis details/data derivations. It also documents modifications or additions to the analysis plan which are not "principal" in nature and result from information that was not available at the time of protocol finalization.

Study mRNA-1273-P301 is a Phase 3, randomized, stratified, observer-blind, placebo-controlled study to evaluate the efficacy, safety, and immunogenicity of messenger ribonucleic acid (mRNA)-1273 SARS-CoV-2 vaccine compared to placebo in adults 18 years of age and older who have no known history of SARS-CoV-2 infection but whose locations or circumstances put them at appreciable risk of acquiring COVID-19 and/or SARS-CoV-2 infection.

PPD Biostatistics and programming team, designee of Moderna Biostatistics and Programming department, will perform the statistical analysis; Statistical Analysis System (SAS) Version 9.4 or higher will be used, and R or RStudio may be used. The SAP will be finalized and approved prior to the first interim analysis clinical database lock and treatment unblinding for the study. If the methods in this SAP differ from the methods described in the protocol, the SAP will prevail.

In this document, subject and participant are used interchangeably; injection of IP, injection, and dose are used interchangeably; vaccination group and treatment group are used interchangeably.

2. Study Objectives

2.1. Primary Objectives

2.1.1. Primary Efficacy Objective

The primary efficacy objective is to demonstrate the efficacy of mRNA-1273 to prevent COVID-19.

2.1.2. Primary Safety Objective

The primary safety objective is to evaluate the safety and reactogenicity of 2 injections of the mRNA-1273 vaccine given 28 days apart.

2.2. Secondary Objectives

2.2.1. Secondary Efficacy Objectives

The secondary efficacy objectives are:

- To evaluate the efficacy of mRNA-1273 to prevent severe COVID-19.
- To evaluate the efficacy of mRNA-1273 to prevent serologically confirmed SARS-CoV-2 infection or COVID-19 regardless of symptomatology or severity.
- To evaluate vaccine efficacy (VE) against a secondary definition of COVID-19.
- To evaluate VE to prevent death caused by COVID-19.
- To evaluate the efficacy of mRNA-1273 to prevent COVID-19 after the first injection.
- To evaluate the efficacy of mRNA-1273 to prevent COVID-19 in all study participants, regardless of evidence of prior SARS-CoV-2 infection.
- To evaluate the efficacy of mRNA-1273 to prevent asymptomatic SARS-CoV-2 infection.

2.2.2. Secondary Immunogenicity Objective

The secondary immunogenicity objective is to evaluate the immunogenicity of 2 injections of the mRNA-1273 vaccine given 28 days apart.

2.3. Exploratory Objectives

The exploratory objectives are:

- To evaluate the effect of mRNA-1273 on the viral infection kinetics as measured by viral load at SARS-CoV-2 infection diagnosis by reverse transcription polymerase chain reaction (RT-PCR) and number of days from the estimated date of SARS-CoV-2 infection until undetectable SARS-CoV-2 infection by RT-PCR.
- To assess VE to reduce the duration of symptoms of COVID-19.
- To evaluate VE against all-cause mortality.
- To assess VE against burden of disease (BOD) due to COVID-19.
- To evaluate the genetic and/or phenotypic relationships of isolated SARS-CoV-2 strains to the vaccine sequence.
- To evaluate immune response markers after dosing with IP as correlates of risk of COVID-19 and as correlates of risk of SARS-CoV-2 infection.
- To conduct additional analyses related to furthering the understanding of SARS-CoV-2 infection and COVID-19, including analyses related to immunology, this or other vaccines, detection of viral infection, and clinical conduct.

3. Study Endpoints

3.1. Primary Endpoints

3.1.1. Primary Efficacy Endpoint

The primary efficacy objective will be evaluated by the VE of mRNA-1273 to prevent the first occurrence of COVID-19 starting 14 days after the second injection of IP, where COVID-19 is defined as symptomatic disease based on the following criteria:

- The participant must have experienced at least TWO of the following systemic symptoms: Fever (≥ 38ºC), chills, myalgia, headache, sore throat, new olfactory and taste disorder(s), OR
- The participant must have experienced at least ONE of the following respiratory signs/symptoms: cough, shortness of breath or difficulty breathing, OR clinical or radiographical evidence of pneumonia; AND
- The participant must have at least one nasopharyngeal (NP) swab, nasal swab, or saliva sample (or respiratory sample, if hospitalized) positive for SARS-CoV-2 by RT-PCR.

3.1.2. Primary Safety Endpoint

The primary safety objective will be evaluated by the following safety endpoints:

- Solicited local and systemic adverse reactions (ARs) through 7 days after each injection of IP.
- Unsolicited adverse events (AEs) through 28 days after each injection of IP.
- Medically-attended AEs (MAAEs) or AEs leading to withdrawal through the entire study period.
- Serious AEs (SAEs) throughout the entire study period.
- Pregnancies and perinatal outcomes.

3.2. Secondary Endpoints

3.2.1. Secondary Efficacy Endpoints

The secondary efficacy objectives will be evaluated by the following endpoints:

- The VE of mRNA-1273 to prevent severe COVID-19, defined as first occurrence of COVID-19 starting 14 days after the second injection of IP, (as per the primary endpoint) AND any of the following:
    - Clinical signs indicative of severe systemic illness, Respiratory Rates ≥ 30 per minute, Heart Rate ≥ 125 beats per minute, SpO_2 ≤ 93% on room air at sea level or PaO_2/FIO_2 < 300 mm Hg, OR
    - Respiratory failure or Acute Respiratory Distress Syndrome (ARDS), (defined as needing high-flow oxygen, non-invasive or mechanical ventilation, or ECMO), evidence of shock (systolic blood pressure < 90 mmHg, diastolic BP < 60 mmHg or requiring vasopressors), OR
    - Significant acute renal, hepatic or neurologic dysfunction, OR
    - Admission to an intensive care unit or death.
- The VE of mRNA-1273 to prevent the first occurrence of either COVID-19 or SARS-CoV-2 infection starting 14 days after the second injection of IP. This endpoint is a combination of COVID-19, defined as for the primary endpoint, and asymptomatic SARS-CoV-2 infection, assessed by seroconversion and with a negative NP swab sample for SARS-CoV-2 at Day 1
- The VE of mRNA-1273 to prevent the secondary case definition of COVID-19 starting 14 days after the second injection of IP. The secondary case definition of COVID-19 is defined as at least one of the following systemic symptoms: fever (temperature ≥ 38ºC), or chills, cough, shortness of breath or difficulty breathing, fatigue, muscle aches or body aches, headache, new loss of taste or smell, sore throat, nasal congestion or rhinorrhea, nausea or vomiting, or diarrhea AND a

positive NP swab, nasal swab, or saliva sample (or respiratory sample, if hospitalized) for SARS-CoV-2 by RT-PCR.

- The VE of mRNA-1273 to prevent death due to a cause directly attributed to a complication of COVID-19, starting 14 days after the second injection of IP.
- The VE of mRNA-1273 to prevent the first occurrence of COVID-19 starting 14 days after the first injection of IP.
- The VE of mRNA-1273 to prevent the first occurrence of COVID-19 starting 14 days after the second injection of IP regardless of evidence of prior SARS-CoV-2 infection determined by serologic titer against SARS-CoV-2 nucleocapsid.
- The VE to prevent the first occurrence of SARS-CoV-2 infection in the absence of symptoms defining COVID-19 starting 14 days after the second injection of IP. SARS-CoV-2 infection will be assessed by seroconversion and with a negative NP swab sample for SARS-CoV-2 at Day 1.

3.2.2. Secondary Immunogenicity Endpoints

The secondary immunogenicity objective will be evaluated by the following endpoints:

- Geometric mean titer (GMT) of SARS-CoV-2-specific neutralizing antibody (nAb) on Day 1, Day 29, Day 57, Day 209, Day 394, and Day 759.
- Geometric mean fold rise (GMFR) of SARS-CoV-2-specific nAb relative to Day 1 on Day 29, Day 57, Day 209, Day 394, and Day 759.
- Quantified levels or GMT of S protein-specific binding antibody (bAb) on Day 1, Day 29, Day 57, Day 209, Day 394, and Day 759.
- GMFR of S protein-specific bAb relative to Day 1 on Day 29, Day 57, Day 209, Day 394, and Day 759.

3.3. Exploratory Endpoints

The exploratory endpoints of the study are:

- The VE of mRNA-1273 on the viral infection kinetics as measured by viral load at SARS-CoV-2 infection diagnosis by RT-PCR and number of days from the estimated date of SARS-CoV-2 infection until undetectable SARS-CoV-2 infection by RT-PCR
- The VE of mRNA-1273 to reduce duration of COVID-19 symptoms
- The VE of mRNA-1273 against BOD based on the post SARS-CoV-2 infection follow-up
- The VE of mRNA-1273 against all-cause mortality
- The genetic and/or phenotypic relationships of isolated SARS-CoV-2 strains to the vaccine sequence
- Immune response markers after dosing with IP as correlates of risk of COVID-19 and as correlates of risk of SARS-CoV-2 infection
- Additional analyses related to furthering the understanding of SARS-CoV-2 infection and COVID-19, including analyses related to the immunology of this or other vaccines, detection of viral infection, and clinical conduct

4. Study Design

4.1. Overall Study Design

This is a Phase 3, randomized, stratified, observer-blind, placebo-controlled study to evaluate the efficacy, safety and immunogenicity of mRNA-1273 vaccine compared to placebo in adults 18 years of age and older who have no known history of SARS-CoV-2 infection but whose locations or circumstances put them at appreciable risk of acquiring COVID-19 and/or SARS-CoV-2 infection. The study schematic is presented in Figure 1.

Approximately 30,000 participants will be randomly assigned to receive either 100 µg of mRNA-1273 vaccine or a placebo control in a 1:1 randomization ratio. Assignment will be

stratified by age and health risk. This is a case-driven study and thus final study size will depend on the actual attack rate of COVID-19.

The study duration will be approximately 26 months for each participant, including a screening period of up to 1 month and a study period of 25 months that consists of 2 IP injections and 24 months afterward. Participants are considered to have completed the study if they complete the final visit at Day 759 (Month 25), 24 months following the last injection of IP. All participants will be assessed for efficacy and safety endpoints and provide a NP swab sample and blood sample before the first and second injection of IP in addition to a series of post-dose blood samples for immunogenicity through 24 months after the second injection of IP. Efficacy assessments will include surveillance for COVID-19 with RT-PCR confirmation of SARS-CoV-2 infection after the first injection of IP. If the pre-specified criteria for early efficacy are met at the time of either interim analysis (IA) or overall efficacy at the primary analysis, an initial study report describing the efficacy and safety of mRNA-1273 will be prepared based on the data available at that time. In the event that success criteria are met either at the time of the interim analyses or when the total number of cases toward the primary endpoint has accrued, subjects will continue to be followed in a blinded fashion until Month 25, to enable assessment of long-term safety and durability of VE (Figure 2). If the study concludes early, all participants will be requested to provide a final blood sample at the time of study conclusion.

Participants will use an electronic diary (eDiary) to report solicited adverse reactions (ARs) after each injection of IP and to prompt an unscheduled clinic visit if experiencing any symptoms of COVID-19. Surveillance for COVID-19 will be performed through weekly contacts with the participants via a combination of telephone calls and completion of an eDiary starting at Day 1 through the end of the study. An illness visit will be scheduled if the participant is experiencing COVID-19 symptoms.

Figure 1 Study Design Schematic

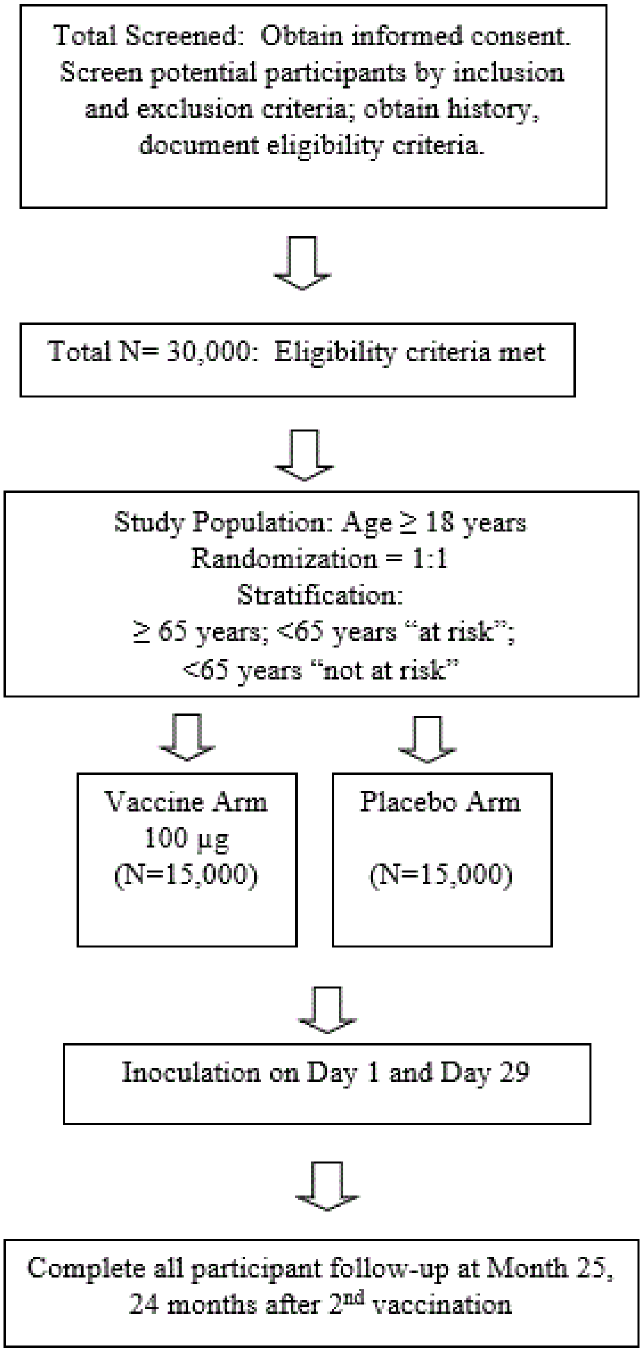

Figure 1 Event/Case-Driven Study with 24 Months of Planned Follow-Up

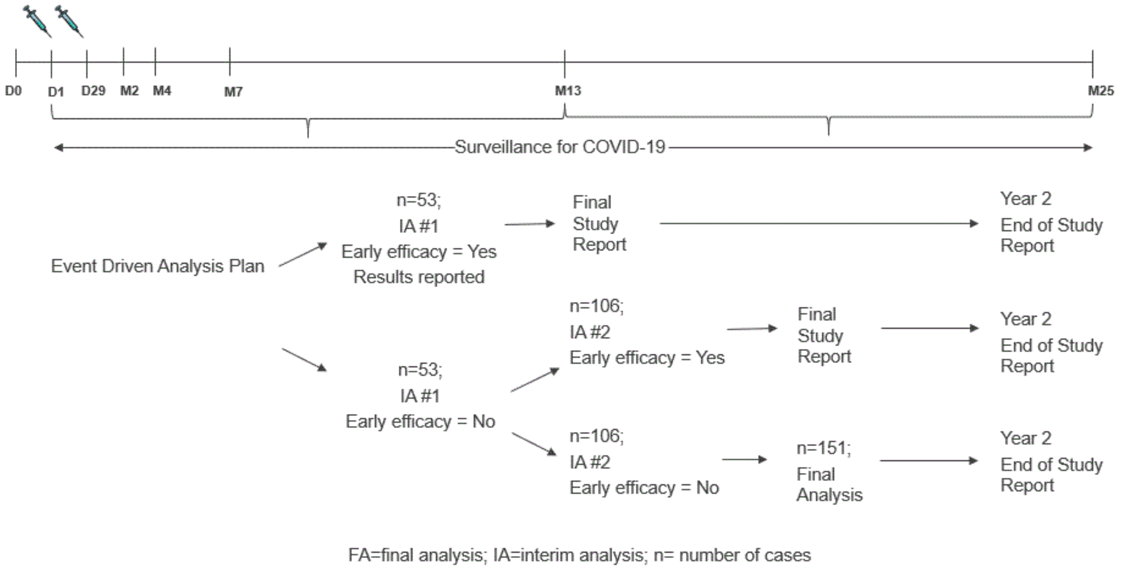

4.2. Statistical Hypothesis

For the primary efficacy objective, the null hypothesis of this study is the VE of mRNA-1273 to prevent first occurrence of COVID-19 is ≤ 30%, i.e. $H_0^{efficacy}$: VE ≤ 0.3.

The study will be considered meeting the primary efficacy objective if the confidence interval (CI) of VE rules out 30% at either one of the interim analyses or at the primary analysis.

4.3. Sample Size and Power

The sample size is driven by the total number of cases to demonstrate the VE (mRNA-1273 vs. placebo) to prevent COVID-19. Under the assumption of proportional hazards over time and with 1:1 randomization to vaccine: placebo, a total of 151 COVID-19 events will provide 90% power to detect a 60% reduction in hazard rate, rejecting the null hypothesis H_0: VE ≤ 30%, with 2 interim analyses at 35% and 70% of the

total events using a 1-sided O'Brien-Fleming boundary for efficacy and a log-rank test statistic with a 1-sided false positive error rate of 0.025. The total number of cases pertains to the PP population, starting 14 days after the second injection of IP (refer to the protocol Section 9.3). Under the specified assumptions, up to approximately 30,000 participants will be randomized. It will take approximately 5, 8, and 10 months from study start (first subject first dose), respectively, to accrue 35% (approximately 53), 70% (approximately 106) and 100% (151) of the target number of cases in the PP Set.

For the secondary objective on the VE against serologically confirmed SARS-CoV-2 infection or COVID-19 regardless of symptomology or severity (COV-INF), the study will have ≥ 90% power to demonstrate the VE is above 30% (to reject null hypothesis VE ≤ 30%) at 1-sided alpha of 2.5% if the true VE to prevent COV-INF is 60%, because every COVID-19 disease endpoint is necessarily a COV-INF endpoint. For the secondary objective on the VE against severe COVID-19, the power depends on the number of severe COVID-19 cases (Table 7 in the protocol).

4.4. Randomization

Approximately 30,000 participants will be randomly assigned in 1:1 ratio to receive either mRNA-1273 100 μg or placebo. The randomization will be in a blinded manner using a centralized Interactive Response Technology (IRT) at the Day 1 visit, in accordance with pre-generated randomization schedules. Participants will be stratified by age (≥ 18 and < 65, or ≥ 65 years) and, if they are < 65 years of age, based on the presence or absence of risk factors for severe illness from COVID-19 based on recommendation by the US Centers for Disease Control and Prevention (CDC) as of March 2020. There will be 3 strata for randomization:

- ≥ 18 and < 65 years and not at risk
- ≥ 18 and < 65 years and at risk
- ≥ 65 years

At least 25% of enrolled participants, but not to exceed 40%, will be from the ≥ 65 years age group and < 65 years at risk group.

4.5. Blinding and Unblinding

This study is an observer-blind study. The investigator, study staff, study participants, site monitors, and Sponsor personnel (or its designees) will be blinded to the IP administered until study end, with the certain exceptions specified in Section 6.2.8 of the protocol.

The Sponsor Biostatistics department or designee will generate the randomized allocation schedule(s) for study treatment assignment. Randomization will be implemented via an IRT.

Planned interim and primary analyses are described in Section 6.8 and Section 9.6 of the protocol. Participant-level unblinding will be restricted to an independent unblinded statistician and, as needed, statistical programmer(s) performing the IAs, who will have no other responsibilities associated with the study.

In addition to the routine study monitoring outlined in this protocol, an external DSMB will review interim data to safeguard the interests of clinical study participants and to enhancing the integrity of the study. The DSMB will review treatment-level results of the IAs, provided by the independent unblinded statistician. Limited additional Sponsor personnel may be unblinded to the treatment-level results of the IAs, if required, in order to act on the recommendations of the DSMB. The extent to which individuals are unblinded with respect to results of IAs will be documented. Depending on the recommendation of the DSMB, the Sponsor may prepare a regulatory submission after an IA. In this case, pre-identified Sponsor members including the analysis and reporting team will be unblinded to treatment assignments and remain unblinded for the remainder of the study. The limited Sponsor and CRO team members to be unblinded in this case will be pre-specified in the study Data Blinding Plan. Participants and investigators will remain blinded.

5. Analysis Populations

The following analysis sets are defined: Randomization Set, Full Analysis Set, Modified Intent-to-Treat, Per-protocol Set, Immunogenicity Subset, Solicited Safety Set, and Safety set.

5.1. Randomization Set

The Randomization Set consists of all subjects who are randomized, regardless of the participant's treatment status in the study. Participants will be analyzed according to the treatment group to which they were randomized.

5.2. Full Analysis Set

The Full Analysis Set (FAS) consists of all randomized participants who received at least one dose of IP. Participants will be analyzed according to the treatment group to which they were randomized.

5.3. Modified Intent-to-Treat Set

The Modified Intent-to-Treat (mITT) Set consists of all participants in FAS who had no immunologic or virologic evidence of prior COVID-19 (ie, negative NP swab test and/or bAb against SARS-CoV-2 nucleocapsid below limit of detection [LOD] or lower limit of quantification [LLOQ]) at Day 1 before the first dose of IP, i.e. all FAS participants excluding those with PCR and/or serology positive at baseline (Day 1 before the first dose of IP).

Participants will be analyzed according to the treatment group to which they were randomized.

5.4. Per-Protocol Set

The Per-protocol Set consists of all participants in mITT who received planned doses of IP per schedule and have no major protocol deviations, as determined and documented by Sponsor prior to database lock (DBL) and unblinding, which impact critical or key study

data. Participants will be analyzed according to the treatment group to which they were randomized.

5.5. Immunogenicity Subset

For characterizing immunogenicity of the vaccine, and for assessing correlates of risk and protection, a case-cohort sampling design will be used for measuring bAb and nAb data from a randomly sampled subset of trial participants [the case-cohort Immunogenicity Analysis Set (ccIAS)]. The ccIAS cohort consists of a stratified random sample of trial participants, augmented with a subset or all primary endpoint cases and SARS-CoV-2 infection endpoint cases. The immunogenicity samples of the ccIAS cohort will be processed and the immunogenicity data will be available for the ccIAS cohort. The Immunogenicity Subset consists of all participants in the ccIAS (i.e., all subjects in the FAS who had a valid immunogenicity test result) who had a valid baseline immunogenicity test result (prior to the first injection of IP) and at least one valid post-baseline result.

The details of the case-cohort sampling will be included in the Analysis Plan for the Immunogenicity Data.

5.6. Solicited Safety Set

The Solicited Safety Set consists of randomized participants who received at least one dose of IP and contributed any solicited AR data, i.e. have at least one post-baseline solicited safety (eDiary) assessment. The Solicited Safety Set will be used for the analyses of solicited ARs and participants will be included in the treatment group corresponding to the study vaccination they actually received.

In addition, the following Solicited Safety Set is defined for each injection separately. The First (Second) Injection Solicited Safety Set consists of all participants in the Solicited Safety Set who have received the first (second) study injection and have contributed any solicited AR data (eDiary) from the time of first (second) study injection through the following 6 days.

Participants will be analyzed according to the treatment group a subject received, rather than the vaccination group to which the participant was randomized. If a participant was randomized to placebo but received any dose of mRNA-1273 vaccine at any injection, the participants will be included in the mRNA-1273 group in the Solicited Safety Set.

5.7. Safety Set

The Safety Set consists of all randomized participants who received at least one dose of IP. The Safety Set will be used for all analysis of safety except for the solicited ARs. Participants will be included in the vaccination group corresponding to the IP they actually received. For a participant who was randomized to placebo but received any dose of mRNA-1273 at any injection, the participant will be included in the mRNA-1273 group in the Safety Set.

6. Statistical Analysis

6.1. General Considerations

The Schedule of Events is provided in the protocol Table 14 (Vaccination Phase, Day 1 – Day 57), Table 15 (Surveillance Phase, Day 64 – Day 394), Table 16 (Surveillance Phase, Day 401 – Day 759), and Table 17 (Convalescent Period, 28-Day Follow-up starting with the Illness Visit).

Continuous variables will be summarized using the following descriptive summary statistics: the number of subjects (n), mean, standard deviation (SD), median, minimum (min), and maximum (max).

Categorical variables will be summarized using counts and percentages.

Baseline value, unless specified otherwise, is defined as the most recent non-missing measurement (scheduled or unscheduled) collected before the first dose of IP.

For the summary statistics of all numerical variables unless otherwise specified, the display precision will follow programming standards. Please see Appendix A for variable display standards.

When count data are presented, the percentage will be suppressed when the count is zero in order to draw attention to the non-zero counts. A row denoted “Missing” will be included in count tabulations where specified on the shells to account for dropouts and missing values. The denominator for all percentages will be the number of subjects in that baseline SARS-CoV-2 status and treatment group within the analysis set of interest, unless otherwise specified.

Baseline SARS-CoV-2 status is determined by using virologic and serologic evidence of SARS-CoV-2 infection on or before Day 1.

Positive baseline SARS-CoV-2 status at Baseline is defined as a positive RT-PCR test for SARS-CoV-2, and/or a positive serology test based on bAb specific to SARS-CoV-2 nucleocapsid (as measured by Roche Elecsys Anti-SARS-CoV-2 assay) on or before Day 1. Subjects with positive status at Baseline will be excluded from the mITT and PP population.

Negative status at Baseline is defined as a negative RT-PCR test for SARS-CoV-2 and a negative serology test (as measured by Roche Elecsys Anti-SARS-CoV-2 assay) at Day 1.

In subgroup analyses on age, age at screening will be used for derivation of age group.

Study day relative to the first injection will be calculated as below:

a) study day prior to the first injection will be calculated as: date of assessment/event – date of the first injection;

b) study day on or after the date of the first injection will be calculated as: date of assessment/event – date of the first injection + 1;

Study day relative to the most recent injection will be calculated as below:

a) study day prior to the first injection will be calculated as: date of assessment/event – date of the first injection;

b) study day on or after the date of the first injection but before the second injection (if applicable) will be calculated as: date of assessment/event – date of the first injection + 1;

c) study day on or after the date of the second injection will be calculated as: date of assessment/event – date of the second injection + 1; if study day is on the same day as the second injection, date and time will be compared with the second injection date and time. If it is prior to the second injection, then study day is calculated as b); If it is after the second injection or the time is missing or not available then study day is calculated as: date of assessment/event – date of the second injection + 1.

For calculations regarding antibody values/titers reported as below LOD or LLOQ will be replaced by 0.5 × LOD or 0.5 × LLOQ. Values that are greater than the upper limit of quantification (ULOQ) will be converted to the ULOQ. Missing results will not be imputed.

The following analysis periods or stages for safety analyses will be used in this study:

- Up to 28 days after any vaccination: this stage starts at the day of each vaccination and continue through the earliest date of (the day of each vaccination and 27 subsequent days, next vaccination [if applicable]). This analysis period will be used as the primary analysis period for safety analyses including unsolicited AE, except for solicited AR, unless specified otherwise.

- Follow-up analysis period:

  For unsolicited AE or assessments that will be collected throughout the study, this analysis period starts from 28 days after the last injection date (i.e. the day of last injection + 28 days, regardless of number of injections received) and continues until the earliest date of (study completion, discontinuation from the study, or death).

For assessments that will be collected at study visits (e.g. vital sign), if a subject receives two injections, this stage starts from the day after Day 57 visit and continues until the earliest date of (study completion, discontinuation from the study, or death); if a subject receives first injection only, this stage starts from the day after Day 29 visit and continues until the earliest date of (study completion, discontinuation from the study, or death).

- Overall period: this analysis period starts at the first injection on Day 1 and continues through the earliest date of (study completion, discontinuation from the study, or death).

Unscheduled visits: Unscheduled visit measurements will be included in analysis as follows:

- In scheduled visit windows per specified visit windowing rules.
- In the derivation of baseline/last on-treatment measurements.
- In the derivation of maximum/minimum on-treatment values and maximum/minimum change from baseline values for safety analyses.
- In individual subject data listings as appropriate.

Visit windowing rules: The analysis visit windows for protocol-defined visits are provided in Appendix B.

Incomplete/missing data:

- Imputation rules for missing dates of prior/concomitant medications, non-study vaccinations and procedures are provided in Appendix C.
- Imputation rules for missing AE dates are provided in Appendix D.
- If the laboratory results are reported as below the LLOQ (e.g. < 0.1), the numeric values will be substituted by 0.5 × LLOQ in the summary. If the laboratory results are reported as greater than the ULOQ (e.g. > 3000), the numeric values will be substituted by ULOQ in the summary statistics for continuous variable.

- Other incomplete/missing data will not be imputed, unless specified otherwise.

Treatment groups

The following treatment groups will be used for summary purposes:

- mRNA-1273
- Placebo

If a subject received any dose of mRNA-1273 at any injection, regardless of the treatment group the subject was randomized to, the subject will be included to mRNA-1273 100 µg group as the actual treatment group received for safety analyses.

All analyses and data summaries/displays for disposition, baseline demographics and characteristics will be provided by Baseline SARS-CoV-2 Status (negative, positive, and overall) and treatment group unless otherwise specified. All analyses and data summaries/displays for efficacy will be provided by treatment group using appropriate analysis population unless otherwise specified.

All analyses will be conducted using SAS Version 9.4 or higher unless otherwise specified.

6.2. Background Characteristics

6.2.1. Subject Disposition

The number and percentage of subjects in the following categories (analysis sets defined in Section 5) will be summarized as defined in Section 6.1 based on Randomization Set:

- Randomization Set
- Full Analysis Set
- Modified Intent-to-Treat Set
- Per-protocol Set
- Immunogenicity Subset

- Solicited Safety Set
- Safety Set

The denominators of the percentages will be based on subjects in the Randomization Set.

The number of subjects in the following categories will be summarized based on subjects screened:

- Number of subjects screened
- Number and percentage of screen failure subjects and the reason for screen failure

The percentage of subjects who screen failed will be based on the number of subjects screened. The reason for screen failure will be based on the number of subjects who screen failed.

The number and percentage of randomized subjects will be summarized by site and by stratification factor at randomization (i.e. ≥ 18 and < 65 years and not at risk, ≥ 18 and < 65 years and at risk, or ≥ 65 years) separately based on the Randomization Set.

The number and percentage of subjects in each of the following disposition categories will be summarized based on the Randomization Set:

- Received each dose of IP
- Prematurely discontinued before receiving the second dose of IP and the reason for discontinuation
- Completed study
- Prematurely discontinued the study and the reason for discontinuation

A subject disposition listing will be provided, including informed consent, subjects who completed the study injection schedule, subjects who completed study, subjects who discontinued from study vaccine or who discontinued from participation in the study, with reasons for discontinuation. A separate listing will be provided for screen failure subjects with reasons for screen failure. Randomization data will be also provided in a listing.

In addition, subjects with any inclusion and exclusion criteria violation will also be provided in a listing.

6.2.2. Demographics and Baseline Characteristics

Descriptive statistics will be calculated for the following continuous demographic and baseline characteristics: age (years), weight (kg), height (cm), body mass index (BMI) (kg/m^2)). The number and percentage of subjects will be provided for categorical variables such as age group, sex, race, ethnicity, randomization stratum per IRT, and risk factors at Screening (have at least one of the following):

- Chronic lung disease (e.g., emphysema and chronic bronchitis, idiopathic pulmonary fibrosis, and cystic fibrosis) or moderate to severe asthma
- Significant cardiac disease (e.g., heart failure, coronary artery disease, congenital heart disease, cardiomyopathies, and pulmonary hypertension)
- Severe obesity (body mass index ≥ 40 kg/m^2)
- Diabetes (Type 1, Type 2 or gestational)
- Liver disease
- Human Immunodeficiency Virus (HIV) infection

The summaries will be provided separately based on the FAS, Safety Set, mITT Set and PP Set.

For screened failure subjects, age (years), gender, race, and ethnicity will be presented in a listing.

6.2.3. Medical History

Medical history data will be coded by system organ class (SOC) and preferred term (PT) using the Medical Dictionary for Regulatory Activities (MedDRA).

The number and percentage of participants with any medical history will be summarized by SOC and PT based on Safety Set. A participant will be counted only once for multiple events within each SOC and PT. SOC will be displayed in internationally agreed order. PT will be displayed in descending order of frequency of mRNA-1273 and then alphabetically within SOC.

6.2.4. Prior and Concomitant Medications

Prior and concomitant medications and non-study vaccinations will be coded using the WHO drug dictionary (WHODD). The summary of concomitant medications will be based on the Safety set. Categorization of prior, concomitant, and post medications is summarized in Appendix C Table 8.

The number and percentage of subjects using concomitant medications and non-study vaccinations during the 7-day follow-up period (i.e. on the day of injection and the 6 subsequent days) and during the 28-day follow-up period after each injection (i.e. on the day of injection and the 27 subsequent days) will be summarized by treatment group as defined in Section 6.1 as follows:

- Any concomitant medications throughout the study
- Any concomitant medications and non-study vaccinations within 7 Days Post Injection
- Any concomitant medications and non-study vaccinations within 28 Days Post Injection
- Seasonal influenza vaccine within 28 Days Post Injection
- Prophylactic antipyretics or analgesics medication within 28 Days Post Injection
- Antipyretic or analgesic medication within 28 Days Post Injection

A summary table of concomitant medications and non-study vaccinations that continued or newly received at or after the first injection through 28 days after the last injection will be provided by PT in descending frequency in the mRNA-1273 group.

Prior, concomitant and post medications and non-study vaccinations will be presented in a listing.

6.2.5. Study Exposure

Study IP administration data will be presented in a listing.

6.2.6. Major Protocol Deviations

Major protocol deviations are a subset of protocol deviations that may significantly impact the completeness, accuracy, or reliability of the study data or that may significantly affect a subject's rights, safety, or well-being. Major protocol deviations rules will be developed and finalized before database lock.

The number and percentage of the subjects with each major protocol deviation type will be provided by treatment group as defined in Section 6.1 based on the Randomization Set.

Major protocol deviations will be presented in a listing.

6.2.7. COVID-19 Impact

A listing will be provided for COVID-19 impact.

6.3. Efficacy Analysis

Efficacy analyses will be performed using the PP Set, mITT Set, and FAS. The PP Set is the primary analysis population used in the efficacy analyses, unless otherwise specified. Subjects will be included in the treatment group which they are randomized to.

Surveillance for COVID-19 will be performed starting after enrollment through the end of the study. An Adjudication Committee (AC) will review potential cases. The AC will be

blinded to treatment assignment. If the assessments of the AC are available, the primary endpoint and the secondary efficacy endpoints will be based on assessments of the AC.

Baseline SARS-CoV-2 status is described in Section 6.1. Baseline SARS-CoV-2 status, the serology test results based on Roche Elecsys assay at Baseline, the RT-PCR test results at baseline on or before Day 1 will be summarized by treatment group.

Participants with baseline positive SARS-CoV-2 status will be excluded from the mITT and PP Sets; participants with baseline positive SARS-CoV-2 status will be included in the FAS and Safety Sets.

In this study, RT-PCR test results will be summarized by visit. Summary tables of the primary and secondary efficacy endpoints will be also provided.

6.3.1. Analysis of Primary Efficacy Endpoint

6.3.1.1.Primary Efficacy Endpoint Definition/Derivation

The primary efficacy endpoint is the VE of mRNA-1273 to prevent occurrence of COVID-19 starting 14 days after the second dose of IP. COVID-19 is defined as symptomatic disease based on the criteria specified in Section 3.1.1 of the protocol. Cases are defined as participants meeting clinical criteria based both on symptoms for COVID-19 and positive RT-PCR test results.
Subjects reporting COVID-19 symptoms, as defined in Section 8.1.2 of the protocol, will be asked to use an eDiary to report COVID-19 symptoms, and an Illness Visit will be scheduled to collect an NP swab if COVID-19 symptoms are reported (refer to Section 8.1.2 of the protocol). In this study, there is a scheduled NP swab test at the Day 29 visit prior to the 2nd injection of IP, the RT-PCR results from the scheduled NP swab tests at the Day 29 visit prior to the 2nd injection of IP will not be considered for derivation of COVID-19 as they are not prompted by symptoms.

For the primary efficacy endpoint, a COVID-19 case will be identified as a positive post-baseline RT-PCR test result that is prompted by symptom(s), together with eligible

symptoms, i.e. a positive PCR result of the eligible symptoms summarized below in Table 1.

Table 1 Derivation for COVID-19 (primary efficacy endpoint)

	**COVID-19**
Post-baseline PCR results at illness visits prompted by symptom(s)<br>(Scheduled NP swab test at Day 29 visit [2nd injection] will not be used for derivation of efficacy endpoints derivation)	Positive, **AND**
Systemic Symptoms	at least **TWO** of the following **systemic symptoms**: Fever (≥ 38ºC), chills, myalgia, headache, sore throat, new olfactory and taste disorder(s); **OR**
Respiratory symptoms	at least **ONE** of the following **respiratory** signs/symptoms: cough, shortness of breath or difficulty breathing, OR clinical or radiographical evidence of pneumonia;

The date of documented COVID-19 (case) will be the later date of ([2 systemic symptoms reported, or respiratory symptom reported] and, [date of positive PCR test]). Specifically, the date of documented COVID-19 will be the later date of the following two dates (date of

positive PCR test, and the date of eligible symptom(s)), and the two dates should be within 14 days of each other.

- Date of positive PCR test,
- Date of eligible symptom(s), defined as earliest of
  - Respiratory symptom: earliest date of an eligible respiratory symptom is reported
  - Systemic symptoms: earliest date of the 2nd eligible systemic symptom is reported

The time to the first occurrence of COVID-19 will be calculated as:

Time to the 1st occurrence of COVID-19 = Date of documented COVID-19 – Date of randomization + 1.

In the primary analysis approach for the primary efficacy endpoint, cases will be counted start 14 days after the 2nd injection, i.e. date of documented COVID-19 － Date of the 2nd injection ≥ 14 (starting 14 days after the 2nd injection.

In the primary analysis approach for the primary efficacy endpoint, the first occurrence of COVID-19 starting 14 days after the 2nd injection will be considered event. In the various sensitivity analyses planned to examine the onset of COVID-19, the censoring rules of cases are outlined in Table 2 below.

Please note, subjects with Baseline Positive SARS-CoV-2 status will be excluded from the mITT and PP Set; subjects with Baseline Positive SARS-CoV-2 status will be included in the FAS and Safety Sets.

6.3.1.2.Primary Analysis Approach

The number and percentage of subjects who had an event (i.e. the first occurrence of COVID-19 starting 14 days after the second injection, date of documented COVID-19 － date of the second injection ≥ 14) and subjects who were censored will be summarized.

The non-parametric Kaplan-Meier method will be used to estimate the time to first occurrence of COVID-19 curve in each treatment group.

Vaccine efficacy is defined as the percent reduction in the hazard of the primary endpoint (mRNA-1273 vs. placebo), i.e. one minus the hazard ratio (HR). Equivalently, the null hypothesis is:

$$H_0^{efficacy}\text{: hazard ratio (HR)} \geq 0.7$$

A stratified Cox proportional hazard (PH) model with Efron's method of tie handling and with treatment group as covariate will be used to assess the magnitude of the treatment group difference (i.e. HR) between mRNA-1273 vs. placebo. The same stratification factors used for randomization (Section 4.4) will be applied to the stratified Cox model. The HR with corresponding alpha-adjusted confidence interval (CI), 95% CI, and 2-sided *p*-value for testing the null hypothesis from the stratified Cox model will be reported.

For the primary analysis, subjects who have no documented COVID-19 will be censored at the last study assessment date. Subjects who discontinue the study early or die due to cause unrelated to COVID-19 without documented COVID-19 will be censored at the date of early discontinuation or death. Subjects who experience an early COVID-19 up to 14 days after the second injection of IP will be censored at the time of documented COVID-19.

As in PP Set definition, subjects who missed study IP administration, who were seropositive at baseline, or who had major protocol deviations that impact critical or key data, will be excluded from the PP Set.

The number and percentage of subjects who had an event (i.e. the first occurrence of COVID-19 at least 14 days after the second injection) and subjects who were censored will be reported.

Potential intercurrent events to the estimand are listed in Appendix F Table 9. The primary estimand with rationale for strategies to address intercurrent events is summarized in Appendix F Table 10.

6.3.1.3. Sensitivity Analyses with COVID-19 Cases Counted Starting at Various Timepoints

Four sets of sensitivity analyses will be performed with COVID-19 cases counted starting at various timepoints as below using the PP Set. The censoring rules for analyses of the primary endpoint are summarized in Table 2.

- immediately after the second injection of IP
- 14 days after the first injection of IP (also as primary analysis approach for the secondary efficacy objective: To evaluate the efficacy of mRNA-1273 to prevent COVID-19 after the first dose of IP.)
- immediately after the first injection of IP
- immediately after randomization

Table 2 Censoring Rules for COVID-19

Situation	Primary Efficacy Approach (cases starting 14 days after the 2nd injection)	Sensitivity Analyses			
		Cases starting immediately after the 2nd injection	Cases starting 14 days after the 1st injection	Cases starting immediately after the 1st injection	Cases starting immediately after randomization
Early case from randomization up to first injection	Censored at date of case	Censored at date of case	Censored at date of case	Censored at date of case	Event
Early case from first injection up to 14 days after first injection	Censored at date of case	Censored at date of case	Censored at date of case	Event	Event
Early case from 14 days after first injection up to second injection	Censored at date of case	Censored at date of case	Event	Event	Event
Early case from second injection up to 14 days after second	Censored at date of case	Event	Event	Event	Event

**Situation**	**Primary Efficacy Approach (cases starting 14 days after the 2nd injection)**	**Sensitivity Analyses**			
		**Cases starting immediately after the 2nd injection**	**Cases starting 14 days after the 1st injection**	**Cases starting immediately after the 1st injection**	**Cases starting immediately after randomization**
injection					
Early case after 14 days after second injection	Event	Event	Event	Event	Event
Early discontinuation or death without documentation of COVID-19	Censored at date of discontinuation/death				

Analysis of the primary efficacy endpoint based on the mITT Set will also be performed using the same statistical methods used for the primary analysis. The above four sets of sensitivity analyses will be also performed with COVID-19 cases counted starting at various timepoints using the mITT Set.

6.3.1.4. Subgroup Analysis

To assess consistency of VE across various subgroups, subgroup analyses of the primary efficacy endpoint will be performed in select subgroups specified below based on the PP Set. The primary efficacy endpoint will be analysed by each of the subgroups using the

same methods described for the primary analysis, i.e. the stratified Cox proportional hazard model with Efron's method of tie handling with a single treatment covariate, and the estimate of VE and its 95% CI will be provided within each category of the following classification variables:

- Age groups: ≥ 18 and < 65 years, ≥ 65 years
- Age groups: ≥ 18 and < 65 years, ≥ 65 years and < 75 years, and, ≥ 75 years
- Stratification factor at randomization: ≥ 18 and < 65 years and not at risk, ≥ 18 and < 65 years at risk, ≥ 65 years
- Sex (female, male)
- Race
- Ethnicity
- Each risk factor for severe COVID-19 illness as listed in Section 6.2.1 of the protocol

If the number of subjects in the certain subgroups are too small, it may be combined with the other subgroups for the subgroup analyses. Forest plots will be provided for VE and its 95% CI for the subgroup analyses based on PP Set. Subgroup analyses based on the mITT Set may be performed.

6.3.1.5. Supportive Analysis Using Exact Method Based on Incidence Rate

As a supportive analysis, VE will also be estimated by one minus the ratio of incidence rate (mRNA-1273 vs. placebo), where incidence rate for each treatment group will be calculated as the number of subjects with an event (i.e. first occurrence of COVID-19 at least 14 days after the second injection) divided by the number of subjects at risk adjusted by person-time (weeks) in each treatment group. The 95% CI will be computed using the exact method conditional upon the total number of cases.

6.3.2. Analysis of Secondary Efficacy Endpoints

A sequential/hierarchical testing procedure will be used to control type 1 error rate over the primary efficacy endpoint and the secondary efficacy endpoints. Secondary efficacy endpoints will only be tested when the primary efficacy endpoint achieves statistical significance. Multiplicity adjustments among the secondary efficacy endpoints may be performed for secondary efficacy endpoints as described in Section 6.5.

6.3.2.1. Derivation of Severe COVID-19

Severe COVID-19 is defined as:

To be considered severe COVID-19, the following criteria must be met:

- Confirmed COVID-19 as per the Primary Efficacy Endpoint case definition, AND
- any of the conditions listed in Section 3.2.1 (Section 8.1.1 of the protocol).

The date of documented severe COVID-19 will be the later date of:

- Date of documented COVID-19,
- Date of eligible symptom for severe COVID-19, defined as the earliest of the first eligible severe symptom is reported

The date of eligible symptoms for severe COVID-19 should be within [-14, +28] days of the positive RT-PCR result used in the confirmation of COVID-19.

The time to the first occurrence of severe COVID-19 will be calculated as:

Time to the 1st occurrence of severe COVID-19 = Date of documented severe COVID-19 – Date of randomization +1,

6.3.2.2. Derivation of Secondary Definition of COVID-19

A secondary definition of COVID-19 is defined as any of the listed systemic symptom AND with a positive RT-PCR test.

As in the derivation of the primary definition of COVID-19, only RT-PCR tests prompted by symptoms will be considered, i.e. excluding RT-PCR results from the scheduled NP

swab tests at Day 29 prior to the 2nd IP dose. Date of the documented secondary definition of COVID-19 will be later date of:

- Date of the positive RT-PCR test (prompt by symptom)
- Date of eligible symptom for secondary definition of COVID-19, defined as the earliest date of first eligible symptom is reported

and the two dates should be within 14 days of each other.

6.3.2.3. Derivation of Serologically Confirmed SARS-CoV-2 Infection or COVID-19 Regardless of Symptomatology or Severity

For the secondary efficacy endpoint: serologically confirmed SARS-CoV-2 infection or COVID-19 regardless of symptomatology or severity, any post-baseline positive RT-PCR results will be considered, including those from the scheduled NP swab tests at Day 29 visit prior to the 2nd injection of IP as well as the those prompted by symptom(s). In addition, seroconversion due to infection will also be considered.

The date of documented infection regardless of symptom will be the earlier of:

- Date of positive post-baseline RT-PCR result, or
- Date of seroconversion due to infection

Seroconversion due to infection is defined differently for participants who were seropositive or seronegative at baseline as below:

- Participants seronegative at Baseline (as measured by *Roche Elecsys*): seropositive as measured by *Roche Elecsys* on study
- Participants seropositive at Baseline (as measured by *Roche Elecsys*): 4-fold or more increase in bAb from baseline as measured by *VAC66 Nucleocapsid IgG Antibody* on study

In the primary approach, documented infection is counted starting 14 days after the 2nd IP dose, which requires a positive RT-PCR result starting 14 days after the 2nd IP dose, or seroconversion at Day 57 visit or later.

Derivation of this secondary efficacy endpoint is summarized in Figure 3 and Table 3.

Figure 3 Scheme of Derivation of Secondary Efficacy Endpoint: Serologically Confirmed SARS-CoV-2 Infection or COVID-19 Regardless of Symptomatology or Severity

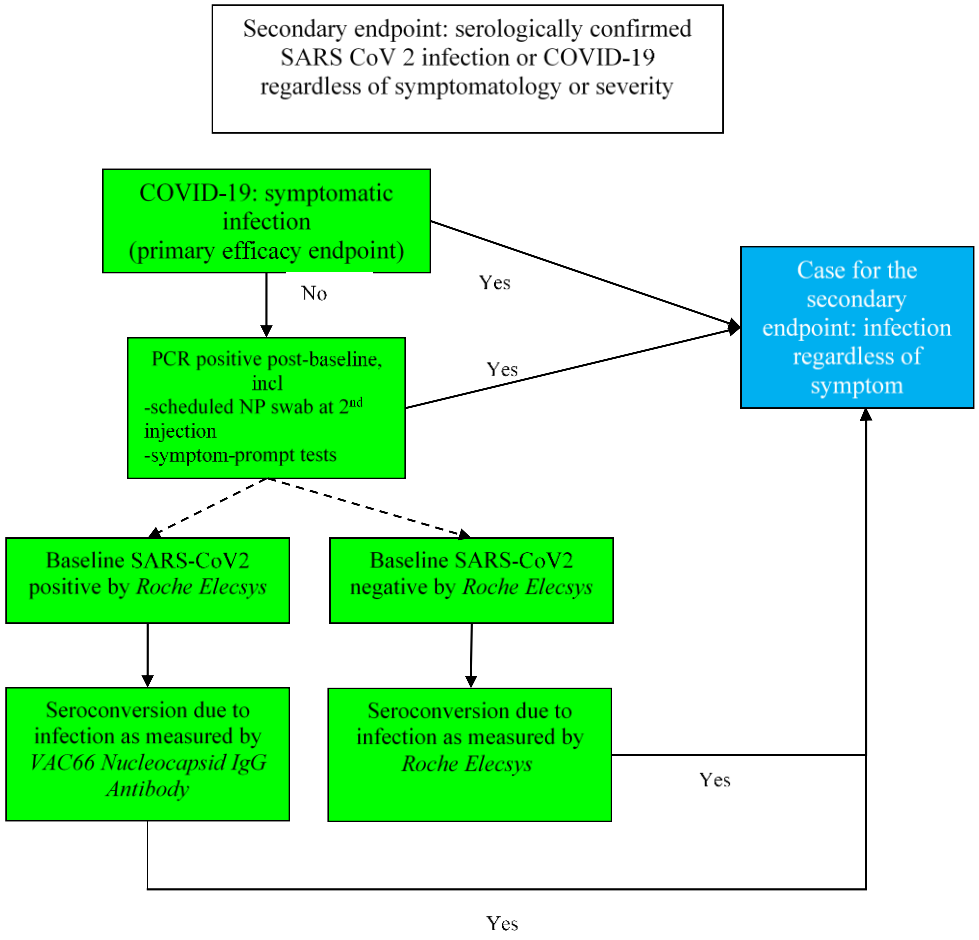

Table 3. Derivation of Secondary efficacy endpoint: Serologically confirmed SARS-CoV-2 infection or COVID-19 regardless of symptomatology or severity

**Baseline SARS-CoV-2 Status**	**Post-baseline assessments**		**Secondary endpoint**: serologically confirmed SARS-CoV-2 infection or COVID-19 regardless of symptomatology or severity
	PCR test post baseline	bAb levels against SARS-CoV-2	
Negative at Baseline	**positive** at symptom-prompt NP swab test (Illness visit), or at scheduled D29 NP swab test before 2nd dose	N/A	Case
Negative at Baseline	No positive PCR test	Seroconversion as meaured by Roche Elecsys	Case
Positive at Baseline			
PCR positive at baseline		N/A	already had prior infection, not at risk, subjects will NOT be a case, and will be censored at date of randomization
PCR negative at baseline, AND, detectable bAb by *Roche Elecsys*	PCR positive		Case
PCR negative at baseline, AND, detectable bAb by *Roche Elecsys*		Seroconversion due to infection, as measured by VAC66 Nucleocapsid IgG Antibody	Case

6.3.2.4. Derivation of Asymptomatic SARS-CoV-2 Infection

SARS-CoV-2 infection is defined by seroconversion due to infection measured by bAb against SARS-CoV-2 nucleocapsid in subjects who had a negative RT-PCR test at baseline. In protocol amendment 2, a Day 29 NP swab prior to Dose 2 is added and thus will be taken into consideration for the efficacy endpoint of SARS-CoV-2 infection. Please note asymptomatic infection is identified by seroconversion only at months 1, 2, 7, 13 and 25, when blood samples for immunogenicity are collected; at month 1 (Day 29), both RT-PCR test and bAb against SARS-CoV-2 nucleocapsid will be considered.

The date of documented asymptomatic infection is the date of seroconversion due to infection, defined as the date of achieving:

- Seropositive as measured by *Roche Elecsys* on study, for participants seronegative at Baseline (as measured by *Roche Elecsys*)
- 4-fold or more increase in bAb from baseline as measured by *VAC66 Nucleocapsid IgG Antibody* on study, for participants seropositive at Baseline (as measured by *Roche Elecsys*)
- Positive RT-PCR at scheduled Day 29 nasal swab test prior to 2nd dose

For participants who had a symptomatic infection (e.g., COVID-19 as primary endpoint, or secondary definition of COVID-19), following seroconversion will not be considered for derivation of asymptomatic infection, i.e. participants who had a symptomatic infection will be censored at the time of symptomatic infection for the analysis of asymptomatic infection. In the primary approach, documented asymptomatic infection is counted starting 14 days after the 2nd IP dose, which requires seroconversion at months 2 (Day 57 visit) or later.

6.3.2.5. Analysis of Secondary Efficacy Endpoints

Similar analysis methods as for the primary efficacy endpoint will be applied to the following secondary efficacy endpoints based on the PP set, unless otherwise specified:

- VE to prevent severe COVID-19
- VE to prevent serologically confirmed SARS-CoV-2 infection or COVID-19 regardless of symptomatology or severity.VE to prevent COVID-19 using a secondary definition of symptoms
- VE to prevent death caused by COVID-19
- VE to prevent asymptomatic SARS-CoV-2 infection

For each of the above secondary objectives, the following analyses will be performed:

- Primary analysis: VE will be estimated with 1 - HR (mRNA-1273 vs. placebo) using a Cox PH model based on the PP Set as described for the primary efficacy endpoint. Kaplan-Meier curves of time to event will be presented for each treatment group.

  Cases will be counted starting 14 days after the second injection of IP.
- Analysis using the same model based on the mITT Set as described for the primary efficacy endpoint. Kaplan-Meier curves of time to event will be presented for each treatment group.
- Sensitivity analyses with cases counted starting immediately after the second injection of IP, 14 days after the first injection of IP, immediately after the first injection of IP, and immediately after randomization respectively.
- VE and 95% CI based on the incidence will be estimated with 1 - ratio of incidence rates using the exact method conditional upon the total number of cases.

In addition, the following efficacy endpoints will be also analyzed:

<u>VE to prevent COVID-19 after the first injection of IP</u>

This endpoint will be analyzed as a sensitivity analysis of the primary efficacy endpoint with cases counted starting 14 days after the first injection of IP as described in Section 6.3.1.

VE to prevent all-cause mortality

This endpoint will be analyzed with the same Cox PH model described above based on the PP Set, mITT Set, and FAS. Death, regardless of cause, from randomization will be included. Time to death will be calculated as date of death – date of randomization + 1. Subjects with no documented death will be censored at the last date of study participation for study completion, early discontinuation, or lost to follow-up.

VE to prevent COVID-19 disease regardless of prior SARS-CoV-2 infection

This endpoint will be analyzed using the FAS. The same methods described above for the primary efficacy endpoint will be applied with cases counted starting at least 14 days after the second injection of IP. Subjects who received only the first injection of IP will be excluded from the statistical analysis. Sensitivity analyses with cases counted starting immediately after the second injection, 14 days after the first injection, immediately after the first injection, and after randomization will also be performed.

In sensitivity analysis with cases counted starting after the second injection, subjects who received only the first injection will be excluded from the statistical analysis. The VE will be also estimated with 1- ratio of incidence rates with the 95% CI using the exact method conditional upon the total number of cases.

In addition, an exploratory analysis with the same Cox model will be carried out in the subgroup of FAS whose baseline SARS-CoV-2 status is positive to assess the VE in those with positive baseline SARS-CoV-2 status, at baseline, if sample size permits.

6.4. Interim Analyses

There are two planned interim analyses in this study, which will be performed when approximately 35% and 70% of the target total number of COVID-19 cases across the two vaccine groups have been observed respectively. The primary objective of the IAs is for early detection of reliable evidence that VE is above 30%. The Lan-DeMets O'Brien-Fleming approximation spending function is used for calculating efficacy bounds and to preserve the (1-sided) 0.025 false positive error rate over the two IAs and the primary

analysis (when the target number of cases have been observed), relative to the null hypothesis. There is no intention to stop the study early if the efficacy has been demonstrated at any of the IAs. If efficacy is demonstrated at an IA, the subsequent IA or primary analysis will be considered supportive in nature. The IA results will be reviewed by the DSMB.

The first IA will occur when 35% of the total cases in the PP set have been observed. The study will be considered positive at this IA if the *p*-value for rejecting HR ≥ 0.7 is less than 0.0002 based on the Lan-DeMets O'Brien-Fleming approximation spending function. This corresponds to an observed HR of approximately 0.259, or an observed VE approximately 0.741.

The second IA will occur when 70% of the total cases have been observed in the PP set. The study will be considered positive at this IA if the *p*-value for rejecting HR ≥ 0.7 is less than 0.0073 based on the Lan-DeMets O'Brien-Fleming approximation spending function. This corresponds to an observed HR of approximately 0.435, or an observed VE of approximately 0.565.

The primary analysis will be performed when approximately 151 cases have been observed in the PP Set. The study will be considered positive at the primary analysis when a total of 151 cases have been observed and if the one-sided *p*-value for rejecting HR ≥ 0.7 is less than 0.0227. This corresponds to an observed hazard ratio of approximately 0.505 or observed VE of approximately 0.495.

An independent, unblinded statistics team will carry out the IAs. The unblinded statistics team will not be involved in either study design or the regular study conduct. The subjects and study sites will remain blinded throughout the study.

The final analysis will be performed after all subjects have completed the study and after the database is cleaned and locked. Results of this analysis will be presented in a CSR, including individual listings.

The timing, number of cases and decision guidance at each IA and primary analysis is summarized in the table below (Table 13 of the protocol).

Table 3: Interim Boundaries Using O'Brien-Fleming Spending function, Calculation Based on the PP Set for the Primary Efficacy Endpoint

Information fraction (% of total #cases)	Number of cases	Nominal Alpha	Efficacy Boundary Rejecting H0: VE ≤ 30% (HR ≥ 0.7)	Cum Prob (crossing efficacy boundary if the true VE = 60%)
IA1 35%	53	0.0002	VE ≥ 0.741 (HR ≤ 0.259)	4.6%
IA2 70%	106	0.0073	VE ≥ 0.565 (HR ≤ 0.435)	61.5%
Primary analysis 100%	151	0.0227	VE ≥ 0.495 (HR ≤ 0.505)	90.0%

Abbreviations: HR = hazard ratio; IA: interim analysis; LB = lower boundary; PP = per-protocol; VE = vaccine efficacy.

6.5. Multiplicity Adjustments

The overall type I error rate for this study is strictly controlled at 2.5% (one-sided). The overall Type I error rate for the primary efficacy endpoint at the IAs and the primary analysis is strictly controlled at 2.5% (1-sided) based on the Lan-DeMets O'Brien-Fleming approximation spending function. The primary efficacy endpoint will be considered statistically significant after consideration of the strategy for controlling the Type I error as described in Section 6.4 Interim Analyses. Statistical significance of the primary efficacy endpoint can be achieved at either one of the interim analyses or at the primary analysis.

A sequential/hierarchical testing procedure will be used to control type 1 error rate over the primary efficacy endpoint and the secondary efficacy endpoints. Secondary efficacy endpoints will only be tested when the primary efficacy endpoint achieves statistical significance.

If the primary efficacy endpoint achieves statistical significance at either one of the interim analyses or at the primary analysis, a fixed-sequence statistical strategy will be used to test the following secondary efficacy endpoints in a pre-defined order:

1. secondary efficacy endpoint: COVID-19 regardless of evidence of prior SARS -CoV-2 infection at the same analysis (through the same follow-up period)
2. secondary efficacy endpoint: infection regardless of symptomatology or severity at the same analysis (through the same follow-up period)
3. secondary efficacy endpoint: severe COVID-19 at the analysis with ≥ 20 cases, otherwise, to test at the end of the study

If the primary efficacy endpoint achieves statistical significance at either one of the interim analyses or at the primary analysis, sequential tests of the above 3 secondary efficacy endpoints will be performed at one-sided Type I error rate of 0.025.

All hypothesis tests against the above three secondary efficacy endpoints will be first against a VE ≤ 0% null hypothesis. If hypothesis test against VE ≤ 0% are rejected for all three, sequential testing against a VE ≤ 10% for these three secondary efficacy endpoints will be performed in the same order; if hypothesis test against VE ≤ 10% are rejected for all three, sequential testing against a VE ≤ 20% for these three secondary efficacy endpoints will be performed in the same order; if hypothesis test against VE ≤ 20% are rejected for all three, sequential testing against a VE ≤ 30% for these three secondary efficacy endpoints will be performed in the same order. No further testing will be performed once the sequence breaks, that is, further testing stops as soon as an endpoint in the sequence fails to show significance against corresponding null hypothesis.

No further testing will be performed once the sequence breaks, that is, further testing stops as soon as an endpoint in the sequence fails to show significance against corresponding null hypothesis regarding VE.

While achieving vaccine efficacy that rules out a lower bound of 30% against COVID-19 is the goal of the study, there is utility in formally assessing VEs that are less than 30%. Therefore, in the situation that the primary efficacy endpoint (VE against COVID-19) does

not achieve statistical significance against a VE ≤ 30% null hypothesis at neither the interim analyses nor the primary analysis, at the primary analysis, sequential testing against a VE ≤ 20%, a VE ≤ 10%, and a VE ≤ 0% will be performed, halting when a rejection occurs.

Figure 4. below demonstrates the testing strategy in this study:

Figure 4 Testing Strategy of Primary and Secondary Efficacy Endpoints

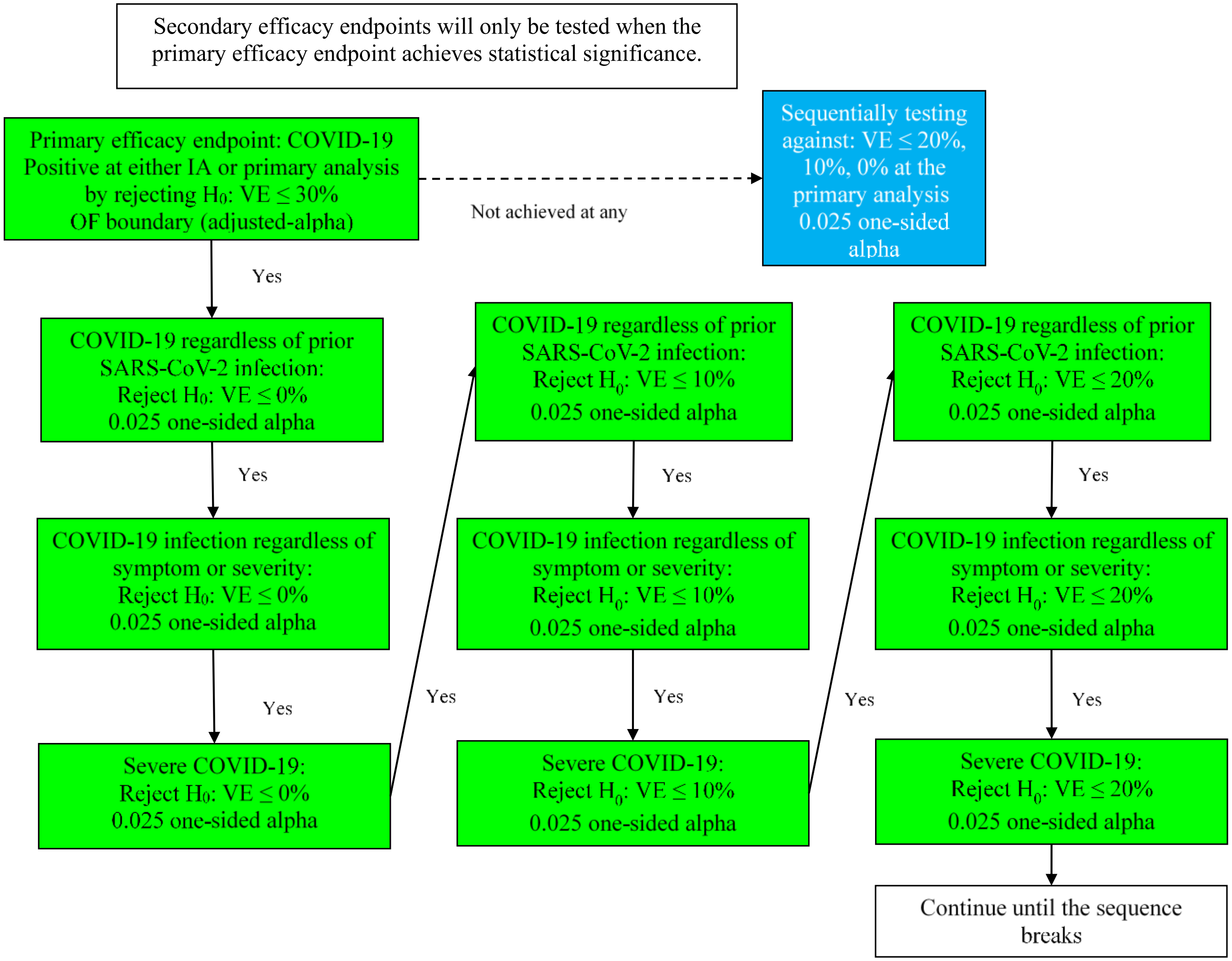

6.6. Safety Analysis

Safety and reactogenicity will be assessed by clinical review of all relevant parameters including solicited ARs (local and systemic), unsolicited AEs, SAEs, MAAEs, AEs leading to withdrawal from study vaccine and/or study participation, vital signs, and physical examination findings. Solicited ARs and unsolicited AEs will be coded by SOC and PT according to the MedDRA. The Toxicity Grading Scale for Healthy Adult and Adolescent Volunteers Enrolled in Preventative Vaccine Clinical Trials (DHHS 2007) is used in this study with modifications for solicited ARs as presented in Table 5 of the study protocol.

All safety analyses will be based on the Safety Set, except summaries of solicited ARs which will be based on the Solicited Safety Set. All safety analyses will be provided by treatment group, unless otherwise specified.

6.6.1. Unsolicited Treatment-emergent Adverse Events

A treatment-emergent AE (TEAE) is defined as any event occurring during the study not present before exposure to the IP or any event already present that worsens after exposure to study vaccine. Worsening of a pre-existing condition after vaccination will be reported as a new AE.

Adverse events will also be evaluated by the investigator for the coexistence of MAAE which is defined as an AE that leads to an unscheduled visit to a healthcare practitioner.

Unsolicited AEs will be coded by PT and SOC using MedDRA and summarized by vaccination group.

Unsolicited AEs will be collected for up to 28 days after each IP dose; SAEs, MAAEs, AEs leading to withdrawal will be collected throughout the study. Analyses of unsolicited AE will be provided for up to 28 days after any vaccination unless otherwise specified; and select analyses of unsolicited AE will be provided for Follow-up analysis period, and overall stage (Section 6.1).

All summary tables (except for the overall summary of AEs) for unsolicited AEs will be presented by SOC and PT or by PT only for TEAEs with counts of subjects included. SOC will be displayed in internationally agreed order. PT will be displayed in descending order of frequency of mRNA-1273 and then alphabetically within SOC. When summarizing the number and percentage of subjects with an event, subjects with multiple occurrences of the same AE or a continuing AE will be counted once. Only the maximum severity level will be presented in the severity summaries, and the strongest relationship level will be presented in the relationship summaries.

Percentages will be based upon the number of subjects in the Safety Set within each treatment group.

6.6.1.1. Overview of Unsolicited TEAEs

An overall summary of unsolicited TEAEs including the number and percentage of subjects who experience the following will be presented:

- Any unsolicited TEAEs
- Any serious AEs
- Any unsolicited AEs that are medically-attended
- Any unsolicited TEAEs leading to discontinuation from participation in the study
- Any unsolicited TEAEs of Grade 3 or higher
- Any unsolicited TEAEs that are fatal

The table will also include number and percentage of subjects with unsolicited TEAEs that are treatment-related in each of the above categories.

The overall summary will be provided for unsolicited TEAE up to 28 days after any injection, follow-up analysis period, and for the Overall Stage throughout the study.

In addition, separate listings containing individual subject adverse event data for unsolicited TEAEs, unsolicited treatment-related TEAEs, unsolicited TEAEs leading to

discontinuation from study vaccine, unsolicited TEAEs leading to discontinuation from participation in the study, unsolicited TEAEs of Grade 3 or higher, unsolicited treatment-related TEAEs of Grade 3 or higher, serious AEs, serious treatment-related AEs, and unsolicited medically-attended AEs will be provided separately.

6.6.1.2. TEAEs by System Organ Class and Preferred Term

The following summary tables of TEAEs will be provided by SOC and PT using frequency counts and percentages (i.e. number and percentage of subjects with an event):

- All unsolicited TEAEs
- All unsolicited TEAEs that are treatment-related
- All serious AEs
- All serious AEs that are treatment-related
- All unsolicited TEAEs leading to discontinuation from study vaccine
- All unsolicited TEAEs leading to discontinuation from participation in the study
- All unsolicited TEAEs of Grade 3 or higher
- All unsolicited TEAEs of Grade 3 or higher that are treatment-related
- All unsolicited AEs that are medically-attended

Summary tables of all unsolicited TEAEs, Serious AEs, treatment-related SAEs, MAAEs, and TEAE leading to discontinuation from participation in the study will be also be provided by SOC and PT for the Follow-up analysis period and Overall Stage

6.6.1.3. TEAEs by Preferred Term

The following summary tables of TEAEs will be provided by PT sorting by frequency on the mRNA-1273 group:

- All unsolicited TEAEs

6.6.1.4. TEAEs by Toxicity Grade

The following summary tables of TEAEs will be provided by SOC and PT and maximum Toxicity Grade using frequency counts and percentages:

- All unsolicited TEAEs
- All unsolicited TEAEs that are treatment-related

6.6.1.5. Subgroup Analysis of TEAEs

An overview of TEAE, TEAE summaries presented by SOC and PT will be provided for the following subgroups:

- Age group (<65, and ≥ 65 years)

6.6.2. Solicited Adverse Reactions

An AR is any AE for which there is a reasonable possibility that the test product caused the AE. The term “Solicited Adverse Reactions” refers to selected signs and symptoms occurring after injection administration during a specified post-injection follow-up period (day of injection and 6 subsequent days). The solicited ARs are recorded by the subject in eDiary. The occurrence and intensity of selected signs and symptoms is actively solicited from the subject during a specified post-injection follow-up period (day of injection and 6 subsequent days), using a pre-defined checklist in the eDiary (i.e. solicited ARs).

The following local ARs will be solicited by the eDiary: pain at injection site, erythema (redness) at injection site, swelling/induration (hardness) at injection site, and localized axillary swelling or tenderness ipsilateral to the injection arm.

The following systemic ARs will be solicited by the eDiary: headache, fatigue, myalgia (muscle aches all over the body), arthralgia (aching in several joints), nausea/vomiting, body temperature (potentially fever), and chills.

The solicited ARs will be graded based on the grading scales presented in Table 5 in the protocol, modified from the Toxicity Grading Scale for Healthy Adult and Adolescent

Volunteers Enrolled in Preventative Vaccine Clinical Trials (DHHS 2007). Investigator will assess the Grading for Grade 4 events (with exception of fever).

If a solicited local or systemic AR continues beyond 7 days post injection, the subject will be prompted to capture solicited local or systemic AR in the eDiary until resolution.

All solicited ARs (local and systemic) will be considered causally related to injection.

Analyses of solicited ARs will be provided by treatment group for each injection (first or second) based on the associated subset of Solicited Safety Set, i.e. First (Second) Injection Solicited Safety Set; and for any injection based on the Solicited Safety Set, unless otherwise specified.

The number and percentage of subjects who reported each individual solicited local AR (has a severity grade of Grade 1 or greater) and solicited systemic AR (has a severity grade of Grade 1 or greater) during the 7-day follow-up period after each injection will be provided by severity grade. The number and percentage of subjects who reported each individual solicited AR will also be summarized by severity grade, days of reporting and injection.

The number and percentage of subjects experiencing fever (a temperature greater than or equal to 38.0°C/100.4°F by the oral, axillary, or tympanic route) by severity grade and the number and percentage of subjects experiencing a fever of Grade 3 or higher temperature (a temperature greater than or equal to 39.0°C/102.1°F by the oral, axillary, or tympanic route) will be provided.

A two-sided 95% exact CI using the Clopper-Pearson method will be provided for the percentage of subjects who reported any solicited local AR, solicited systemic AR, or any solicited AR.

The onset of individual solicited AR is defined as the time point after each injection at which the respective solicited AR first occurred. The number and percentage of subjects with onset of individual solicited AR will be summarized by study day relative to the corresponding injection (Day 1 through Day 7).

The number of days will be calculated as the days of the solicited AR is reported within the 7 days of injection including the day of injection, no matter it is intermittent or continued. If the solicited AR continues beyond 7 days, the consecutive days a solicited AR is reported after 7 days will be included (e.g. an event that lasted 5 days in the first 7 days post injection and 3 consecutive days beyond 7 days post injection, the duration will be reported as 8 (5+3) days.)

All solicited ARs that continue beyond 7 days post injection will be summarized.

The above analyses of solicited ARs will be provided for the following subgroups:

- Age group (<65, and ≥ 65 years)

6.6.3. Vital Sign Measurements

Vital sign measurements, including systolic and diastolic blood pressures, heart rate, respiratory rate, and body temperature, will be presented in a data listing. The values meeting the toxicity grading criteria will be flagged in the data listing. The abnormalities meeting the toxicity grading criteria (Grade 2 or higher) in any vital sign measurement will be listed separately. If a subject has a vital sign result with Grade 2 or higher abnormality at any post injection visit, then all results of vital sign measurement for that subject will be presented in the listing.

Observed values and changes from baseline for all vital sign measurements will be summarized at each visit by treatment group as defined in Section 6.1. Shift from baseline in the toxicity grades will also be summarized at each visit by treatment group.

6.7. Immunogenicity Analysis

Immunogenicity Analysis will be based on the Immunogenicity Subset (Section 5.5). A standalone Analysis Plan for immunogenicity data will describe the details of the case-cohort sampling of the Subset, and analyses of exploratory objectives on immunogenicity including correlates of risk and protection. Analyses of immunogenicity will not be performed at the interim analyses, but at the Primary analysis.

This SAP includes analyses of immunogenicity data for the secondary immunogenicity objective: To evaluate the immunogenicity of 2 doses of mRNA-1273 given 28 days apart.

6.7.1. Immunogenicity Data

Below are the immunogenicity assessments included in this study:

- Serum bAb level against SARS-CoV-2 as measured by enzyme-linked immunosorbent assay (ELISA) specific to the SARS-CoV-2 S protein (vaccine antigen), including tests such as VAC58 Spike IgM Antibody, VAC58 Spike IgA Antibody, and VAC65 Spike IgG Antibody
- Serum nAb titer against SARS-CoV-2 as measured by pseudovirus and/or live virus neutralization assays, including tests such as PsVNT50, PsVNT80, and MN50 (live virus neutralization assay)

6.7.2. Summary of Antibody-Mediated Immunogenicity Endpoints

Data from quantitative immunogenicity assays will be summarized for each treatment group using positive response rates and geometric means with 95% CI at each timepoint when an assessment is performed. Data from qualitative (i.e., yielding a positive or negative result) assays will be summarized by tabulating the frequency of positive responses for each assay by treatment group at each timepoint that an assessment is performed.

- Geometric mean titer (GMT) or value with corresponding 95% CI will be provided at each time point. The 95% CIs will be calculated based on the t-distribution of the log-transformed values then back transformed to the original scale for presentation. GM level and corresponding 95% CI will be plotted at each timepoint. The following descriptive statistics will be also provided at each time point: the number of subjects (n), median, minimum and maximum.
- Geometric mean fold rise (GMFR) with corresponding 95% CI will be provided at each post-baseline timepoint over pre-injection baseline at Day 1. The 95% CIs will

be calculated based on the t-distribution of the log-transformed values then back transformed to the original scale for presentation. GMFR and corresponding 95% CI will be plotted at each timepoint. The following descriptive statistics will be also provided at each time point: the number of subjects (n), median, minimum and maximum.

- The number and percentage of subjects with fold-rise ≥ 2, fold-rise ≥ 3, and fold-rise ≥ 4 from Baseline at each post injection time points will be tabulated with 2-sided 95% Clopper Pearson CIs.

- Proportion of subjects with seroconversion due to vaccination will be tabulated with 2-sided 95% Clopper-Pearson CIs at each post-baseline timepoint. Seroconversion due to vaccination at a subject level is defined as a change from below the LOD or LLOQ to equal to or above LOD or LLOQ, or at least 4-fold rise if baseline is equal to or above LOD or LLOQ in terms of bAb specific to vaccine antigen (i.e. S protein) or nAb.

The GMT and GM levels will be calculated using the following formula:

$$10^{\left\{\frac{\sum_{i=1}^{n}\log_{10}(t_i)}{n}\right\}}$$

$$10^{\left\{\frac{\sum_{i=1}^{n}\log_{10}(t_i)}{n}\right\}}$$

where $t_1, t_2, \ldots, t_n$ $t_1, t_2, \ldots, t_n$ are *n* observed immunogenicity titers or levels.

The geometric mean fold rise (GMFR) measures the changes in immunogenicity titers within subjects. The GMFR will be calculated using the following formula:

$$10^{\left\{\frac{\sum_{i=1}^{n}\log_{10}\left(v_{ij}/v_{ik}\right)}{n}\right\}} = 10^{\left\{\frac{\sum_{i=1}^{n}\log_{10}(v_{ij})-\log_{10}(v_{ik})}{n}\right\}}$$

$$10^{\left\{\frac{\sum_{i=1}^{n}\log_{10}\left(v_{ij}/v_{ik}\right)}{n}\right\}} = 10^{\left\{\frac{\sum_{i=1}^{n}\log_{10}(v_{ij})-\log_{10}(v_{ik})}{n}\right\}}$$

where, for *n* subjects, v_{ij} v_{ij} and v_{ik} v_{ik} are observed immunogenicity titers or levels for subject *i* at time points *j* and *k*, $j \neq k$.

6.7.3. Analysis of Antibody-Mediated Immunogenicity Endpoints

To assess the magnitudes of the differences between the two treatment groups (mRNA-1273 vs. Placebo) in SARS-CoV-2-specific nAb and S protein-specific bAb, a mixed model for repeated measures (MMRM) using SAS PROC MIXED will be used.

For each of the SARS-CoV-2-specific nAb and S protein-specific bAb, the analysis will be performed separately for subjects who are seropositive and seronegative at baseline based on corresponding nAb or bAb respectively. The model will include all available log-transformed anti-body titers at each visit as the dependent variable, treatment groups, visit (as a class variable), and treatment-by-visit interaction visit as fixed effects, with adjustment for stratification factor, baseline log-transformed anti-body titers if applicable, and subject as a random effect. An unstructured covariance structure will be used to model the within-subject errors. A Kenward-Roger approximation will be used for the denominator degrees of freedom. If there is a convergence issue due to the unstructured covariance matrix, a compound symmetry covariance structure will be used to model the within-subject errors. No imputation of missing data will be done.

The geometric least squares mean (GLSM) and corresponding 2-sided 95% CI for the anti-body titers for each treatment group will be provided by visit. In addition, the ratio of GLSM and the corresponding 2-sided 95% CI will be provided to assess the treatment difference (mRNA-1273 vs. Placebo) at each visit. The GLSM, and corresponding 95% CI

results in log-transformed scale estimated from the model will be back-transformed to obtain these estimates in the original scale. Geometric mean ratio (GMR), estimated by the ratio of GLSM and the corresponding 2-sided 95% CI will be provided to assess the treatment difference between mRNA-1273 group vs. placebo group at each visit.

In addition, an analysis of covariance (ANCOVA) model with the treatment group and baseline values as explanatory variables, adjusting for the stratification factor at randomization, baseline value if applicable, will be used to assess the treatment effect at specific timepoints (scheduled visits) such as Day 57. GLSM with 95% CI for each treatment group, GMR with 95% CI for treatment difference will be estimated from the ANCOVA model.

6.8. Exploratory Analysis of Efficacy

Exploratory analyses of efficacy described in this section will not be performed at the interim analyses, and may be performed at the primary analysis, with the exception of exploratory analyses of Burden of Disease (BOD) and Burden of Infection (BOI) that will be performed at the interim analyses and provided to the DSMB.

6.8.1. Vaccine Efficacy Against Burden of Disease

Exploratory analysis of BOD due to COVID-19 will be performed. A BOD score is defined based on the post SARS-CoV-2 infection follow-up to reflect the worst severity of symptoms as shown in Table 4.

Table 4 Burden of Disease Score

**Subject State**<br>**(Worst Category Following Disease Detection)**	**BOD Score**
Without COVID-19<br>(uninfected/asymptomatic infection)	0

COVID-19 without hospitalization<br>(symptomatic without hospitalization)	1
COVID-19 with hospitalization	2
Death	3

Summary of BOD score, number and percentage of subject with each level of BOD score, will be provided by treatment group using the PP Set. A t-test of the BOD scores will be performed to compare mRNA-1273 vs. placebo group.

BOD score will be summarized by periods of follow-up time, subjects who are un-diseased at the beginning of each period will be included in the summary for the corresponding period. There will be 4 periods: from randomization through 6 months; 6 months through 12 months; 12 months through 18 months; and 18 months through completion. To assess impact of baseline risk of severe disease on the vaccine effect regarding disease severity, summary of BOD will be provided by randomization strata (i.e. ≥ 65 years, < 65 years at risk, and < 65 years not at risk).

In order to assess the disease burden in subjects with COVID-19, the above analyses will also be performed in participants with COVID-19, i.e. subjects with BOD score of zero will be excluded from the analysis.

While transparent, the proportions in the summary table will change with increased follow-up time as more subjects inevitably acquire disease. This makes comparisons across different periods of follow-up difficult. To augment this analysis, a proportional means model will be used to assess the treatment effect on BOD between mRNA-1273 and placebo in terms of ratio of mean severity score. The proportional means model allows for direct comparisons across different periods of follow-up, unaffected by differential follow-up. A proportional means model including vaccination group as fixed effect and stratified with stratification factor at randomization will be used to assess the vaccine effect on

BOD. The VE for the BOD score will be calculated as 1 minus the ratio of means as estimated by a weighted Cox model and reported with 95% confidence intervals.

6.8.2. Vaccine Efficacy Against Burden of Infection

To fully understand the impact of vaccination on disease severity, asymptomatic infections should also be evaluated. Similarly, a burden of infection (BOI) score will be calculated as in Table 5 and used to understand the impact. Because asymptomatic infection is identified by seroconversion only at months 1, 2, 7, 13 and 25, summary of BOI will be provided for relevant periods (i.e. Baseline through M1, M1 through M2, M2 through M7, M7 through M13, and M13 through M25) only using subjects who have serological data for that period. A t-test of the BOI scores will be performed to compare mRNA-1273 vs. placebo group. A proportional means model including vaccination group as fixed effect and stratified with stratification factor at randomization will be used to assess the vaccine effect on BOI. The VE for BOI will be calculated as 1 minus the ratio of mean BOI scores and reported with 95% confidence intervals. Similarly, in order to assess the infection/disease burden in subjects with infection regardless of symptoms, the above analyses will also be performed in participants with infection regardless of symptoms, i.e. subjects with BOI score of zero will be excluded from such analysis.

Table 5 Burden of Infection Score

**Patient State**<br>**(Worst Category Following Disease Detection)**	**BOI Score**
No infection	0
Asymptomatic infection	1/2
COVID-19 without hospitalization<br>(Symptomatic without hospitalization)	1
COVID-19 with Hospitalization	2

Death	3

6.8.3. Vaccine Efficacy on Duration and Presence/Severity of COVID-19 Symptoms

Subjects will report symptoms including fever (temperature ≥ 38ºC), chills, cough, shortness of breath, difficulty breathing, fatigue, muscle aches (myalgia), body aches, headache, new loss of taste, new loss of smell, sore throat, nasal congestion, runny nose (rhinorrhea), nausea, vomiting, and diarrhea. The severity scoring system of the symptoms is presented in Table 6. A score endpoint aggregating duration and presence/severity of COVID-19 symptoms may be calculated as the sum of daily severity scores of all recorded symptoms across all days with symptoms. A subject will have an aggregation score of 0 if never diagnosed with COVID-19. The VE of mRNA-1273 based on mean score of each treatment group will be calculated as below:

VE = [1 – Mean score of mRNA-1273 group / Mean score of placebo group] × 100%

Table 6 Grading of COVID-19 Symptoms

Grading	All Symptoms	For Nausea/Vomiting ONLY	For Sense of Smell/Taste ONLY	Score
None	No symptom			0
Mild	I had the symptom, but I could still do my normal activities	I was able to eat and drink normally	I had the symptom, but I retained some taste/smell	1
Moderate	The symptom really bothered me. It was hard to do my	It bothered me enough that I did not eat or drink	My taste/smell was significantly affected.	2

	normal activities.	normally.		
Severe	The symptom was very bad. I was not able to do activities that I usually do.	I could not eat or drink.	I have no taste or smell.	3

A proportional means model including vaccination group as fixed effect and stratified with stratification factor at randomization will be used to assess the VE (mRNA-1273 vs. placebo) on the score aggregating duration and presence/severity of COVID-19 symptoms based on the PP Set.

6.8.4. Vaccine Efficacy Against COVID-19 Over Time Based on Instantaneous Hazard Ratio

For exploratory analysis of the VE on the COVID-19 primary endpoint over time, the instantaneous hazard ratio (mRNA-1273 vs. placebo) of the endpoint may be estimated with pointwise and 95% confidence intervals, using nonparametric kernel smoothing estimation of each of the mRNA-1273 and placebo arm hazard functions over time, using the method of Gilbert *et al*. (Gilbert, 2002) with optimal bandwidths selected using the analytical formula in Andersen *et al*. (Andersen, 1993). At each interim analysis, a plot of the hazard ratio results, as well as plots of the point estimates and pointwise and simultaneous 95% confidence interval estimates for the treatment-arm specific hazard functions, may be provided. If the number of severe COVID-19 events (secondary efficacy endpoint) becomes large enough to warrant analysis (upon DSMB recommendation), the same analysis may be applied for this endpoint.

6.8.5. Durability of Vaccine Efficacy Against COVID-19 Using Covariate Adjustment Method Based on Cumulative Incidence

As an exploratory analysis to assess durability of VE against the primary endpoint, cumulative incidence VE may be assessed using a parameter $VE_{CI}(0 - t)$, defined as one minus the ratio of cumulative incidences (mRNA-1273 vs. placebo) of the COVID-19

endpoint over follow-up through time t post enrollment. All times t ranging from 0 to t^* will be considered, where t^* is defined as the final time through which at least 1,000 subjects are at risk for the COVID-19 endpoint. The cumulative incidence for each vaccination group will be estimated using a covariate adjustment method based on Zeng (2004) that makes use of baseline subjection information. These covariates will include demographic information such as age, sex, and race/ethnicity, and stratification factor at randomization. The most useful covariates to adjust for in producing potential efficiency gains are potential modifiers of VE.

The covariate adjustment method that will be used is more robust than unadjusted approaches (e.g., the Kaplan-Meier estimator), as it yields valid inferences even in cases in which loss to follow-up is informative, provided within each intervention arm the possible relationship between censoring and the COVID-19 endpoint can be explained by observed baseline covariates. Unadjusted methods, in contrast, can give biased answers in this setting, which manifests as 95% confidence intervals that contain the true VE less than 95% of the time, or as tests of the null H_0: VE $\leq$ 30% that reject with probability greater than the specified type 1 error level. Also, the covariate adjustment method often results in more precise estimates when available baseline covariates (e.g. stratification factor at randomization) are predictive of the COVID-19 endpoint, thereby yielding tighter confidence intervals for the cumulative VE and higher-powered tests of the null hypothesis. This efficiency gain may be especially pronounced in settings where the randomization is not stratified on factors such as age category or trial site.

The estimator for the survival function in arm v (mRNA-1273 or placebo) that adjusts for baseline covariates L is implemented using the following steps:

1. (a) Using only data from arm v, fit a main terms Cox regression to estimate the hazard of the COVID-19 endpoint conditionally on L. Refer to the fitted vector of coefficients as $\hat{\beta}$.

(b) Repeat step (a), but estimate the hazard of *censoring* conditionally on L. Refer to the fitted vector of coefficients as $\hat{\gamma}$.

2. For given coefficient $\hat{\alpha}$ (= $\hat{\beta}$ or $\hat{\gamma}$) and a given covariate value ℓ, let $f_{\hat{\alpha}}(\ell)$ denote the $j \in 1, \ldots, 4$ for which $q_{\hat{\alpha},j-1} < \hat{\alpha}^\top \ell \leq q_{\hat{\alpha},j}$, where $q_{\hat{\alpha},1}, q_{\hat{\alpha},2}, q_{\hat{\alpha},3}$ denote the empirical quartiles of $\hat{\alpha}^\top L$ in arm v and $q_{\hat{\alpha},0} = -\infty$ and $q_{\hat{\alpha},4} = \infty$.
3. Using only data from arm v, fit a stratified Kaplan Meier using the COVID-19 endpoint as outcome within all non-empty strata of $(f_{\hat{\beta}}(L), f_{\hat{\gamma}}(L))$ – there will be at most 16 such strata. If any strata are empty, estimate the survival function within those strata as the constant function 1.
4. Estimate the arm-specific survival function by taking a weighted average across the 16 strata, where each stratum-specific survival function receives weight equal to the empirical probability of a randomly selected Subject (from either the vaccine or placebo arm) having baseline covariates belonging to that stratum.

Estimates of the arm-specific cumulative incidences and cumulative incidence VE are obtained by transforming the estimated arm-specific survival functions. Missing covariates will be accounted for using median imputation, which preserves the randomization of treatment even given imputed covariates.

The validity of this estimator relies on the condition that, within all strata of L, there must be a positive probability that a Subject will have follow-up through time t^*. As such, covariates such as trial site should not be included in if the enrollment timeline is such that this condition fails to hold.

Two-sided Wald-based 95% confidence intervals for a log-transformed cumulative incidence ratio estimate will be provided, and these intervals will be transformed to yield intervals for $VE_I(0-t)$, $t \in [0, t^*]$. Accompanying uniform confidence bands, constructed using the multiplier bootstrap with Rademacher weights (Kosorok 2007), will also be provided. The covariate-adjusted arm-specific cumulative incidence estimates will also be plotted over time, with confidence intervals and uniform confidence bands defined

by transforming a Wald interval for a complementary log-log transformation of the corresponding survival functions.

6.9. Summary of Protocol Safety Review and Data Safety Review Board

6.9.1. Protocol Safety Review Team

A Protocol Safety Review Team (PSRT) will be formed to review interim and cumulative blinded safety data on a regular basis with a remit to escalate concerns to the DSMB. The PSRT will be composed of the study's three coordinating investigators and representatives from Moderna, National Institute of Allergy and Infectious Diseases (NIAID), Biomedical Advanced Research and Development Authority (BARDA), and the CRO. The PSRT composition, its remit, and the routine and systematic procedures for safety review and oversight of blinded interval and cumulative data during the mRNA-1273 P301 study by the PSRT is documented in the Moderna mRNA-1273 P301 Protocol Safety Review Team Charter (current version: Version 1.0)

6.9.2. Data Safety Monitoring Board (DSMB)

The Coronavirus Disease 2019 (COVID-19) Vaccine Data and Safety Monitoring Board (DSMB) will monitor all randomized COVID-19 vaccine studies supported by the United States Government (USG). The independent DSMB will periodically review blinded and unblinded data of mRNA-1273-P301, including

- continuous monitoring for potential vaccine harm and non-efficacy based on imbalance between mRNA-1273 vs. placebo for both COVID-19 and severe COVID-19 case counts.
- regular review of safety data at scheduled data review meetings
- review the two planned interim analyses

The statistical considerations and methods for DSMB is documented in the Analysis Plan for DSMB (current version: Version 2.0 dated 07-August-2020).

6.9.2.1.Potential Vaccine Harm

Potential vaccine harm will be assessed based on imbalance between mRNA-1273 vs. placebo for both COVID-19 and severe COVID-19 case counts using the Safety Set. Subjects will be analyzed according to the IP that they actually received regardless of the group to which they were randomized. For harm monitoring, cases will be counted starting after the first dose of study vaccination. Potential harm will be continuously monitored by DSMB with each case of COVID-19 and severe COVID-19.

Cases of COVID-19 for the harm monitoring are defined as for the primary efficacy endpoint but are based on the Safety Set rather than the PPS.

Details of harm monitoring are provided in the analysis plan for DSMB.

7. Changes from Planned Analyses in Protocol

Not applicable.

8. References

Andersen, P.K., Borgan, O., Gill, R.D., Keiding, N. Statistical Models Based on Counting Processes. Springer. 1993.

Estimands and Sensitivity Analysis in Clinical Trials. To The Guideline on Statistical Principles for Clinical Trials. International Council for Harmonisation (ICH) E9(R1). November 2019.

Gilbert, P.B., Wei, L. J., Kosorok, M. R., Clemens, J. D. Simultaneous Inferences on the Contrast of Two Hazard Functions with Censored Observations. Biometrics. 2002 Dec; 58(4): 773-80.

Guidance for Industry: Toxicity Grading Scale for Healthy Adult and Adolescent Volunteers Enrolled in Preventive Vaccine Clinical Trials. U. S. Department of Health and Human Services (DHHS). Food and Drug Administration. Center for Biologics Evaluation and Research. September 2007.

Zeng D. Estimating marginal survival function by adjusting for dependent censoring using many covariates. The Annals of Statistics. 2004, 32(4), 1533-1555.

9. List of Appendices

9.1. Appendix A Standards for Variable Display in TFLs

Continuous Variables: The precision for continuous variables will be based on the precision of the data itself. The mean and median will be presented to one more significant figure than the original results; the SD will be presented to two more significant figures than the original results; the minimum and maximum will be presented to the same precision as the original results.

Categorical Variables: Percentages will be presented to 1 decimal place. If the count is 0, the percentage will not be displayed. If the count equals the denominator, the percentage will be displayed as 100.

9.2. Appendix B Analysis Visit Windows

Analysis will be summarized using the following analysis visit window for post injection assessments:

Step 1: If the assessments are collected at a scheduled visit, the collected data will be mapped to the nominal scheduled visit.

Step 2: If the assessments are collected at an unscheduled visit, the collected data will be mapped using the analysis visit windows described in Table 7 below. For subjects with confirmed COVID-19, unscheduled assessments will be preferably mapped to the visits with respect to the confirmation of COVID-19 (i.e. Illness Visit Day xx) over nominal scheduled visits (i.e. Day xx).

If a subject has multiple assessments within the same analysis visit, the following rule will be used:

- If multiple assessments occur within a given analysis visit, the assessment closest to the target study day will be used.
- If there are 2 or more assessments equal distance to the target study day, the last assessment will be used.

Table 7 Analysis Visit Windows

Visit	Target Study Day	Visit Window in Study Day
**Nasopharyngeal swab (or saliva)**		
Day 1	1 (Date of First Injection)	1, Pre-first-dose
Day 29		
Illness Visit Day 1	X (Date of NP Swab test)	X
Illness Visit Day 3	X+2	[X+1, X+2]
Illness Visit Day 5	X+4	[X+3, X+4]
Illness Visit Day 7	X+6	[X+5, X+6]
Illness Visit Day 9	X+8	[X+7, X+10]

Illness Visit Day 14	X+13	[X+11, X+16]
Illness Visit Day 21	X+20	[X+17, X+23]
Illness Visit Day 28	X+27	[X+24, X+34]
**Vital Signs**		
Day 1	1 (Date of First Injection)	1, Pre-first-dose
Day 1	1 (Date of First Injection)	1, Post-first-dose
Day 29	29 (Date of Second Injection)	[2, 43] Pre-second-dose
Day 29	29 (Date of Second Injection)	[2, 43] Post-second-dose
Day 57	57	[44, 88]
Day 119	119	[89, 164]
Day 209	209	[165, 301]
Day 394	394	[302, 576]
Day 759	759	[577, 773]
Illness Visit Day 1	X (Date of COVID-19 Confirmation)	X
Illness Visit Day 28	X+27	[X+2, X+34]
**Immunogenicity**		
Day 1	1	1, Pre-first-dose
Day 29	29 (Date of Second Injection)	[23, 36] Pre-second-dose
Day 57	57	[44, 133]
Day 209	209	[134, 301]
Day 394	394	[302, 576]
Day 759	759	[577, 773]

Illness Visit Day 1	X (Date of NP Swab test)	X
Illness Visit Day 28	X+27	[X+2, X+34]

9.3. Appendix C Imputation Rules for Missing Dates of Prior/Concomitant Medications and Non-Study Vaccinations

Imputation rules for missing or partial start/stop dates of medication are defined below:

1. Missing or partial medication start date:
   - If only Day is missing, use the first day of the month, unless:
     - The medication end date is on/after the date of first injection or is missing/partial AND the start month and year of the medication coincide with the start month and year of the first injection. In this case, use the date of first injection.
   - If Day and Month are both missing, use the first day of the year, unless:
     - The medication end date is on/after the date of first injection or is missing/partial AND the start year of the medication coincide with the start year of the first injection. In this case, use the date of first injection.
   - If Day, Month, and Year are all missing, the date will not be imputed, but the medication will be treated as though it began prior to the first injection for purposes of determining if status as prior or concomitant.
2. Missing or partial medication stop date:
   a. If only Day is missing, use the earliest date of (last day of the month, study completion, discontinuation from the study, or death).
   b. If Day and Month are both missing, use the earliest date of (last day of the year, study completion, discontinuation from the study, or death).
   c. If Day, Month, and Year are all missing, the date will not be imputed, but the medication will be flagged as a continuing medication.

In summary, the prior, concomitant or post categorization of medications and non-study vaccinations is described in Table 8 below.

Table 8 Prior, Concomitant, and Post Categorization of Medications and Non-study Vaccinations

	Medication Stop Date		
**Medication Start Date**	**< First Injection Date of IP**	**≥ First Injection Date and ≤ 28 Days After Last Injection**	**> 28 Days After Last Injection [2]**
< First injection date of IP [1]	P	P, C	P, C, A
≥ First injection date and ≤ 28 days after last injection	-	C	C, A
> 28 days after last injection	-	-	A

A: Post; C: Concomitant; P: Prior

[1] includes medications with completely missing start date

[2] includes medications with completely missing end date

9.4. Appendix D Imputation Rules for Missing Dates of AEs

Imputation rules for missing or partial start dates and stop dates of AEs are defined below:

1. Missing or partial start date:

   - If only Day is missing, use the first day of the month, unless:

     - The AE end date is on/after the date of first injection or is missing/partial AND the start month and year of the AE coincide with the start month and year of the first injection. In this case, use the date and time of first injection, even if AE time was collected.

- If Day and Month are both missing, use the first day of the year, unless:
    - The AE end date is on/after the date of first injection or is missing/partial AND the start year of the AE coincides with the start year of the first injection. In this case, use the date and time of first injection, when time is available.
- If Day, Month, and Year are all missing, the date will not be imputed. However, if the AE end date is prior to the date of first injection, then the AE will be considered a pre-treatment AE. Otherwise, the AE will be considered treatment-emergent.

2. Missing or partial end dates will not be imputed.

9.5. Appendix E Schedule of Events

Please refer to Table 14, Table 15, Table 16, and Table 17 in Appendix 1 Schedule of Events in the protocol.

9.6. Appendix F Estimands and Estimand Specifications

Table 9: Intercurrent Event Types

Label	Intercurrent Event Type	Comment
IcEv1 (death without confirmation of cases, i.e., unrelated death)	Unrelated death without documented confirmed COVID-19	Participants who die due to reasons unrelated to COVID-19 without confirmation of being a case will all be included in statistical analysis.
IcEv2 (early infection)	Infection starting up to 14 days after the second dose of IP	Participants who experience an early infection up to 14 days after the second dose of IP will all be included in statistical analysis.
IcEv3 (missing dose of IP)	Not receiving the second dose of IP per protocol schedule	Participants who miss a dose of IP will be excluded from the PP, but in the FAS and the mITT.
IcEv4 (SARS-CoV-2 positive at baseline)	Participants with SARS-CoV-2 positive status at baseline	Participants who were SARS-CoV-2 positive at baseline will be excluded from the PP and the mITT but included in the FAS.

10. Abbreviation: IcEv: intercurrent event, PP: per protocol, FAS: full analysis set, mITT: modified intent-to-treat.

Table 10: Primary Objective and Estimands with Rationale for Strategies to Address Intercurrent Events for Per Protocol Analysis

**Objective: To demonstrate the efficacy of mRNA-1273 to prevent COVID-19**	
**Estimand Description**	Vaccine efficacy will be measured using 1 – HR (mRNA-1273/Placebo) of COVID-19 from 14 days after second dose of IP in adults. A while alive strategy will be used for deaths unrelated to COVID-19, and a treatment policy strategy for early infection. A principal stratum strategy is used to exclude participants missing a dose of IP or being SARS-CoV-2 positive at baseline.
**Target Population**	Adults aged 18 years and older in circumstances at a high risk of SARS-CoV-2 infection but without medical conditions that pose additional risk of developing severe disease.<br>The population excludes those previously infected or vaccinated for SARS-CoV-2 or with a medical condition, on treatment that poses additional risks (including those requiring immunosuppressants or immune-modifying drugs), or SARS-CoV-2 pre-positive.
**Variable/Endpoint**	Time to COVID-19 Disease, censoring at early discontinuation, early infection, or last assessment for an event not being observed, whichever comes earlier.
**Treatment Condition(s)**	**Test:** mRNA-1273<br>**Reference:** Placebo
**Estimand Label**	**Estimand 1**
**Population-Level Summary**	Vaccine efficacy defined as 1 - HR of mRNA-1273/Placebo
**Intercurrent Event Strategy**	
**IcEv1 (**unrelated death**):**	While alive
**IcEv2 (**early infection**):**	Treatment policy
**IcEv3 (**Missed dose of IP**):**	Principal stratum

Objective: To demonstrate the efficacy of mRNA-1273 to prevent COVID-19			
**IcEv4 (**SARS-CoV-2 positive at baseline**):**	Principal stratum	N/A	N/A
**Rationale for Strategy(s)**	While alive: unrelated death without confirmation of COVID-19 will be censored at the time of death.<br>Treatment policy: early case will be censored at the time of confirmation.<br>Principal stratum: Participants who were SARS-CoV-2 positive at baseline or missed dose of IP are excluded from this Estimand.		

ModernaTX, Inc.

Protocol mRNA-1273-P301

A Phase 3, Randomized, Stratified, Observer-Blind, Placebo-Controlled Study to Evaluate the Efficacy, Safety, and Immunogenicity of mRNA-1273 SARS-CoV-2 Vaccine in Adults Aged 18 Years and Older

Statistical Analysis Plan
Amendment 1, Part A

SAP Version 2.0
Version Date of SAP: 08 March 2021

Prepared by:

PPD
3575 Quakerbridge Road
Suite 201
Hamilton, NJ 08619

Confidential

DOCUMENT HISTORY

Version	Date	Description of main modifications
1.0	08 September 2020	Original Version (Version 1.0)
2.0	01 March 2021	Amendment 1 (Version 2.0)<br>• Updated SAP for final blinded analysis based on blinded data after protocol amendment 6 that offers participants opportunities to be unblinded and to receive mRNA-1273 for those who were randomized to Placebo<br>• Updated overall study design based on protocol amendment 6<br>• Updated Section 6.3.1 and 6.3.2 on derivation of efficacy endpoints to clarify how scheduled RT-PCR and bAb specific to SARS-CoV-2 NP at scheduled timepoints will be considered for disease efficacy endpoints<br>• Updated Section 6.3.2.2 on analysis of vaccine efficacy to prevent asymptomatic SARS-CoV-2 infection<br>• Analysis of exploratory objective VE against all-cause mortality was moved from Section 6.3.2.2. to Section 6.8.4.<br>• Added Section 6.4.2 to describe the analyses that have been conducted to date for the P301 study<br>• Added Section 6.6.1.6<br>• Updated Section 6.7 to include sampling plan for immunogenicity, update immunogenicity data and analyses planned to characterize immunogenicity of mRNA-1273

TABLE OF CONTENTS

LIST OF ABBREVIATIONS VII

1. INTRODUCTION 10

2. STUDY OBJECTIVES 11

2.1. PRIMARY OBJECTIVES 11
2.1.1. Primary Efficacy Objective 11
2.1.2. Primary Safety Objective 12
2.2. SECONDARY OBJECTIVES 12
2.2.1. Secondary Efficacy Objectives 12
2.2.2. Secondary Immunogenicity Objective 12
2.3. EXPLORATORY OBJECTIVES 12

3. STUDY ENDPOINTS 13

3.1. PRIMARY ENDPOINTS 13
3.1.1. Primary Efficacy Endpoint 13
3.1.2. Primary Safety Endpoint 14
3.2. SECONDARY ENDPOINTS 14
3.2.1. Secondary Efficacy Endpoints 14
3.2.2. Secondary Immunogenicity Endpoints 16
3.3. EXPLORATORY ENDPOINTS 16

4. STUDY DESIGN 17

4.1. OVERALL STUDY DESIGN 17
4.2. STATISTICAL HYPOTHESIS 20
4.3. SAMPLE SIZE AND POWER 20
4.4. RANDOMIZATION 21
4.5. BLINDING AND UNBLINDING 21

5. ANALYSIS POPULATIONS 22

5.1. RANDOMIZATION SET 22
5.2. FULL ANALYSIS SET 22
5.3. MODIFIED INTENT-TO-TREAT SET 23
5.4. PER-PROTOCOL SET 23
5.5. IMMUNOGENICITY SUBSET AND ANALYSIS POPULATIONS FOR IMMUNOGENICITY ... 23
5.6. SOLICITED SAFETY SET 24
5.7. SAFETY SET 25

6. STATISTICAL ANALYSIS 25

6.1. GENERAL CONSIDERATIONS 25
6.2. BACKGROUND CHARACTERISTICS 30
6.2.1. Subject Disposition 30

6.2.2. Demographics and Baseline Characteristics ... *31*
6.2.3. Medical History ... *32*
6.2.4. Prior and Concomitant Medications ... *32*
6.2.5. Study Exposure ... *33*
6.2.6. Major Protocol Deviations ... *33*
6.3. EFFICACY ANALYSIS ... 34
6.3.1. Analysis of the Primary Efficacy Endpoint ... *34*
6.3.1.1. Primary Efficacy Endpoint Definition/Derivation ... 34
6.3.1.2. Primary Analysis Approach ... 37
6.3.1.3. Sensitivity Analyses with COVID-19 Cases Counted Starting at Various Timepoints ... 39
6.3.1.4. Subgroup Analysis ... 42
6.3.1.5. Supportive Analysis Using Exact Method Based on Incidence Rate ... 43
6.3.2. Analysis of the Secondary Efficacy Endpoints ... *43*
6.3.2.1. Derivation of Secondary Efficacy Endpoint: Severe COVID-19 ... 43
6.3.2.1.1. Derivation of Secondary Efficacy Endpoint: Severe COVID-19 ... 43
6.3.2.1.2. Derivation of a secondary definition of COVID-19 ... 44
6.3.2.1.3. Derivation of serologically confirmed SARS-CoV-2 infection or COVID-19 regardless of symptomatology or severity ... 45
6.3.2.1.4. Derivation of asymptomatic SARS-CoV-2 infection ... 47
6.3.2.2. Analysis of the secondary efficacy endpoints ... 47
6.4. PLANNED ANALYSES AND ANALYSES OF EFFICACY ANALYSES THAT HAVE BEEN CONDUCTED ... 50
6.4.1. Planned Interim Analyses and Primary Analysis ... *50*
6.4.2. Analyses of Efficacy that Have been Conducted ... *52*
6.4.2.1. The First Interim Analysis ... 52
6.4.2.2. The Primary Analysis of Efficacy ... 53
6.4.3. Final Analysis of Efficacy for blinded phase ... *54*
6.5. MULTIPLICITY ADJUSTMENTS ... 55
6.6. SAFETY ANALYSIS ... 58
6.6.1. Unsolicited Treatment-emergent Adverse Events ... *58*
6.6.1.1. Overview of Unsolicited TEAEs ... 60
6.6.1.2. TEAEs by System Organ Class and Preferred Term ... 61
6.6.1.3. TEAEs by Preferred Term ... 61
6.6.1.4. TEAEs by Toxicity Grade ... 61
6.6.1.5. Subgroup Analysis of TEAEs ... 62
6.6.1.6. Summary of AEs in Part B at the Final Analysis of Blinded Data ... 62
6.6.2. Solicited Adverse Reactions ... *63*
6.6.3. Vital Sign Measurements ... *65*
6.7. IMMUNOGENICITY ANALYSIS ... 65
6.7.1. Sampling of the Immunogenicity Subset (Case-cohort Sampling Design) ... *66*
6.7.2. Per-Protocol Random Subcohort for Immunogenicity ... *69*
6.7.3. Immunogenicity Data ... *69*

6.7.4. Summary of Antibody-Mediated Immunogenicity Endpoints 70
6.7.5. Analysis of Antibody-Mediated Immunogenicity Endpoints 72
6.7.6. Summary of Immunogenicity in COVID-19 Cases on mRNA-1273 with negative SARS-CoV-2 status at baseline 73
6.8. EXPLORATORY ANALYSIS OF EFFICACY 73
6.8.1. Vaccine Efficacy Against Burden of Disease 74
6.8.2. Vaccine Efficacy Against Burden of Infection... 75
6.8.3. Vaccine Efficacy on Duration and Presence/Severity of COVID-19 Symptoms 76
6.8.4. Vaccine Efficacy Against All-cause Mortality... 77
6.8.5. Vaccine Efficacy Against COVID-19 Over Time 78
6.8.6. Durability of Vaccine Efficacy Against COVID-19 78
6.9. SUMMARY OF PROTOCOL SAFETY REVIEW AND DATA SAFETY MONITORING BOARD 80
6.9.1. Protocol Safety Review Team.. 81
6.9.2. Data Safety Monitoring Board (DSMB)... 81
6.9.2.1. Potential Vaccine Harm 81

7. CHANGES FROM PLANNED ANALYSES IN PROTOCOL 82

8. REFERENCES 82

9. LIST OF APPENDICES 84

9.1. APPENDIX A STANDARDS FOR VARIABLE DISPLAY IN TFLS..................................84
9.2. APPENDIX B ANALYSIS VISIT WINDOWS 85
9.3. APPENDIX C IMPUTATION RULES FOR MISSING DATES OF PRIOR/CONCOMITANT MEDICATIONS AND NON-STUDY VACCINATIONS..87
9.4. APPENDIX D IMPUTATION RULES FOR MISSING DATES OF AES...............................89
9.5. APPENDIX E SCHEDULE OF EVENTS 90
9.6. APPENDIX F ESTIMANDS AND ESTIMAND SPECIFICATIONS 91

LIST OF TABLES

Table 1 Derivation for COVID-19 (primary efficacy endpoint) 35

Table 2 Censoring Rules for COVID-19 40

Table 3 Derivation of Secondary efficacy endpoint: Serologically confirmed SARS-CoV-2 infection or COVID-19 regardless of symptomatology or severity – mITT Set or Per-Protocol Set 46

Table 4 Interim Boundaries Using O'Brien-Fleming Spending function, Calculation Based on the PP Set for the Primary Efficacy Endpoint . 52

Table 5 Part B groups and analysis period for safety 62

Table 6 Random Subcohort Sample Sizes by Baseline Strata 68

Table 7 Burden of Disease Score 74

Table 8 Burden of Infection Score 75

Table 9 Grading of COVID-19 Symptoms 76

Table 10 Analysis Visit Windows 85

Table 11 Prior, Concomitant, and Post Categorization of Medications and Non-study Vaccinations 89

Table 12 Intercurrent Event Types 91

Table 13 Primary Objective and Estimands with Rationale for Strategies to Address Intercurrent Events for Per Protocol Analysis 92

List of Abbreviations

Abbreviation	Definition
AE	adverse event
ANCOVA	analysis of covariance
AR	adverse reaction
ARDS	acute respiratory distress syndrome
bAb	binding antibody
BARDA	Biomedical Advanced Research and Development Authority
BMI	body mass index
BOD	burden of disease
BOI	burden of infection
CDC	Centers for Disease Control and Prevention
CI	confidence interval
COVID-19	coronavirus disease 2019
CRO	contract research organization
CSP	clinical study protocol
DHHS	Department of Health and Human Services
ECMO	extracorporeal membrane oxygenation
eCRF	electronic case report form
eDiary	electronic diary
ELISA	enzyme-linked immunosorbent assay
FAS	full analysis set
GM	geometric mean
GMFR	geometric mean fold rise
GMR	geometric mean ratio
GMT	geometric mean titer
HR	hazard ratio

Abbreviation	Definition
ICH	International Council for Harmonisation
IP	investigational product
IRT	interactive response technology
LOD	limit of detection
LLOQ	lower limit of quantification
MAAEs	medically-attended adverse events
MedDRA	Medical Dictionary for Regulatory Activities
mRNA	messenger ribonucleic acid
nAb	neutralizing antibody
NIAID	National Institute of Allergy and Infectious Diseases
NP	nasopharyngeal
PDV	Participant Decision Visit
PH	proportional hazard
PP	per-protocol
PT	preferred term
REML	restricted (or residual, or reduced) maximum likelihood
RT-PCR	reverse transcription polymerase chain reaction
SAE	serious adverse event
SAP	statistical analysis plan
SARS-CoV-2	severe acute respiratory syndrome coronavirus 2
SAS	Statistical Analysis System
SD	standard deviation
SOC	system organ class
TEAE	treatment-emergent adverse event
ULOQ	upper limit of quantification
VE	vaccine efficacy
WHO	World Health Organization

Abbreviation	Definition
WHODD	World Health Organization drug dictionary

1. Introduction

The previously approved SAP version 1.0, dated 08-September-2020 described the planned analyses for Study mRNA-1273-P301. SAP version 1.0 was based on the clinical study protocol (CSP) Amendment 3, dated 20-August-2020, and electronic case report form (eCRF) Version 2.026, dated 21-AUG-2020. SAP version 1.0 was finalized and approved prior to the planned first interim analysis (IA1, data snapshot 1 occurred on 11-November-2020). Primary efficacy objective based on COVID-19 was demonstrated based on pre-defined statistical criterion at the first interim analysis. The primary analysis of efficacy was performed based on Data Snapshot 2 (25-November-2020) with the total number of COVID-19 cases based on adjudication committee assessment > 151, the target total number of events at the originally planned primary analysis (Section 9.6 of the protocol). The results based on IA1 (DS1, 11-NOV-2020) and Primary analysis (DS2, 25-NOV-2020) served as the basis for application of mRNA-1273 for Emergency Use Authorization (EUA).

This statistical analysis plan (SAP), version 2.0, is based on clinical study protocol (CSP) Amendment 6, dated 23-December-2020 and the approved electronic case report form (eCRF) Version 9.015, dated 04-FEB-2021. The CSP Amendment 6 informs all ongoing study participants of the availability and eligibility criteria of any COVID-19 vaccine made available under an EUA and offers participants who originally received placebo in this study the potential benefit of vaccination against COVID-19, given that the primary efficacy endpoint for mRNA-1273 against COVID-19 was met per the protocol-defined interim analysis.

This SAP Amendment 1 (Part A) focuses on the blinded phase of the study, and a separate SAP (Part B) will be provided for the unblinded phase analyses not covered in this SAP. A final blinded efficacy analysis is planned to provide final efficacy update based on data from the blinded phase of the study when approximately 90% of the study participants have had the participant decision visit or have been unblinded or discontinued from the study. Unless specified otherwise, the language in this SAP pertains to Part A.

The updates in SAP version 2.0 are summarized in Document History.

In addition to the information presented in the statistical analysis plan section of the protocol (Section 9) which provides the principal features of analyses for this study, this SAP provides statistical analysis details/data derivations. It also documents modifications or additions to the analysis plan which are not "principal" in nature and result from information that was not available at the time of protocol finalization.

Study mRNA-1273-P301 is a Phase 3, randomized, stratified, observer-blind, placebo-controlled study to evaluate the efficacy, safety, and immunogenicity of messenger ribonucleic acid (mRNA)-1273 SARS-CoV-2 vaccine compared to placebo in adults 18 years of age and older who have no known history of SARS-CoV-2 infection but whose locations or circumstances put them at appreciable risk of acquiring COVID-19 and/or SARS-CoV-2 infection.

PPD Biostatistics and programming team, designee of Moderna Biostatistics and Programming department, will perform the statistical analysis; Statistical Analysis System (SAS) Version 9.4 or higher will be used, and R or RStudio may be used. SAP version 1.0, dated 08-September-2020 was finalized and approved prior to the first interim analysis clinical database lock and treatment unblinding for the study. If the methods in this SAP differ from the methods described in the protocol, the SAP will prevail.

In this document, subject and participant are used interchangeably; injection of IP, injection, and dose are used interchangeably; vaccination group and treatment group are used interchangeably.

2. Study Objectives

2.1. Primary Objectives

2.1.1. Primary Efficacy Objective

The primary efficacy objective is to demonstrate the efficacy of mRNA-1273 to prevent COVID-19.

2.1.2. Primary Safety Objective

The primary safety objective is to evaluate the safety and reactogenicity of 2 injections of the mRNA-1273 vaccine given 28 days apart.

2.2. Secondary Objectives

2.2.1. Secondary Efficacy Objectives

The secondary efficacy objectives are:

- To evaluate the efficacy of mRNA-1273 to prevent severe COVID-19.
- To evaluate the efficacy of mRNA-1273 to prevent serologically confirmed SARS-CoV-2 infection or COVID-19 regardless of symptomatology or severity.
- To evaluate vaccine efficacy (VE) against a secondary definition of COVID-19.
- To evaluate VE to prevent death caused by COVID-19.
- To evaluate the efficacy of mRNA-1273 to prevent COVID-19 after the first injection.
- To evaluate the efficacy of mRNA-1273 to prevent COVID-19 in all study participants, regardless of evidence of prior SARS-CoV-2 infection.
- To evaluate the efficacy of mRNA-1273 to prevent asymptomatic SARS-CoV-2 infection.

2.2.2. Secondary Immunogenicity Objective

The secondary immunogenicity objective is to evaluate the immunogenicity of 2 injections of the mRNA-1273 vaccine given 28 days apart.

2.3. Exploratory Objectives

The exploratory objectives are:

- To evaluate the effect of mRNA-1273 on the viral infection kinetics as measured by viral load at SARS-CoV-2 infection diagnosis by reverse transcription polymerase chain reaction (RT-PCR) and number of days from the estimated date of SARS-CoV-2 infection until undetectable SARS-CoV-2 infection by RT-PCR.
- To assess VE to reduce the duration of symptoms of COVID-19.
- To evaluate VE against all-cause mortality.
- To assess VE against burden of disease (BOD) due to COVID-19.
- To evaluate the genetic and/or phenotypic relationships of isolated SARS-CoV-2 strains to the vaccine sequence.
- To evaluate immune response markers after dosing with IP as correlates of risk of COVID-19 and as correlates of risk of SARS-CoV-2 infection.
- To conduct additional analyses related to furthering the understanding of SARS-CoV-2 infection and COVID-19, including analyses related to immunology, this or other vaccines, detection of viral infection, and clinical conduct.

3. Study Endpoints

3.1. Primary Endpoints

3.1.1. Primary Efficacy Endpoint

The primary efficacy objective will be evaluated by the VE of mRNA-1273 to prevent the first occurrence of COVID-19 starting 14 days after the second injection of IP, where COVID-19 is defined as symptomatic disease based on the following criteria:

- The participant must have experienced at least TWO of the following systemic symptoms: Fever (≥ 38ºC), chills, myalgia, headache, sore throat, new olfactory and taste disorder(s), OR

- The participant must have experienced at least ONE of the following respiratory signs/symptoms: cough, shortness of breath or difficulty breathing, OR clinical or radiographical evidence of pneumonia; AND
- The participant must have at least one nasopharyngeal (NP) swab, nasal swab, or saliva sample (or respiratory sample, if hospitalized) positive for SARS-CoV-2 by RT-PCR.

3.1.2. Primary Safety Endpoint

The primary safety objective will be evaluated by the following safety endpoints:

- Solicited local and systemic adverse reactions (ARs) through 7 days after each injection of IP (Part A: Blinded Phase only).
- Unsolicited adverse events (AEs) through 28 days after each injection of IP (Part A: Blinded Phase only).
- Medically-attended AEs (MAAEs) or AEs leading to withdrawal through the entire study period.
- Serious AEs (SAEs) throughout the entire study period.

3.2. Secondary Endpoints

3.2.1. Secondary Efficacy Endpoints

The secondary efficacy objectives will be evaluated by the following endpoints:

- The VE of mRNA-1273 to prevent severe COVID-19, defined as first occurrence of COVID-19 starting 14 days after the second injection of IP, (as per the primary endpoint) AND any of the following:
    - o Clinical signs indicative of severe systemic illness, Respiratory Rates ≥ 30 per minute, Heart Rate ≥ 125 beats per minute, $SpO_2 \leq 93\%$ on room air at sea level or $PaO_2/FIO_2 < 300$ mm Hg, OR

  - Respiratory failure or Acute Respiratory Distress Syndrome (ARDS), (defined as needing high-flow oxygen, non-invasive or mechanical ventilation, or ECMO), evidence of shock (systolic blood pressure < 90 mmHg, diastolic BP < 60 mmHg or requiring vasopressors), OR
  - Significant acute renal, hepatic or neurologic dysfunction, OR
  - Admission to an intensive care unit or death.
- The VE of mRNA-1273 to prevent the first occurrence of either COVID-19 or SARS-CoV-2 infection starting 14 days after the second injection of IP. This endpoint is a combination of COVID-19 (primary endpoint or secondary definition of COVID-19 cases) and asymptomatic SARS-CoV-2 infection assessed by seroconversion, for participants with negative SARS-CoV-2 status at baseline.
- The VE of mRNA-1273 to prevent the secondary case definition of COVID-19 starting 14 days after the second injection of IP. The secondary case definition of COVID-19 is defined as at least one of the following systemic symptoms: fever (temperature ≥ 38ºC), or chills, cough, shortness of breath or difficulty breathing, fatigue, muscle aches or body aches, headache, new loss of taste or smell, sore throat, nasal congestion or rhinorrhea, nausea or vomiting, or diarrhea AND a positive NP swab, nasal swab, or saliva sample (or respiratory sample, if hospitalized) for SARS-CoV-2 by RT-PCR.
- The VE of mRNA-1273 to prevent death due to a cause directly attributed to a complication of COVID-19, starting 14 days after the second injection of IP.
- The VE of mRNA-1273 to prevent the first occurrence of COVID-19 starting 14 days after the first injection of IP.
- The VE of mRNA-1273 to prevent the first occurrence of COVID-19 starting 14 days after the second injection of IP regardless of evidence of prior SARS-CoV-2 infection determined by serologic titer against SARS-CoV-2 nucleocapsid or RT-PCR at baseline.

- The VE to prevent the first occurrence of SARS-CoV-2 infection in the absence of symptoms defining COVID-19 starting 14 days after the second injection of IP. SARS-CoV-2 infection will be assessed by seroconversion and with a negative NP swab sample for SARS-CoV-2 at Day 1.

3.2.2. Secondary Immunogenicity Endpoints

The secondary immunogenicity objective will be evaluated by the following endpoints:

- Geometric mean titer (GMT) of SARS-CoV-2-specific neutralizing antibody (nAb) on Day 1, Day 29, Day 57, Day 209, Day 394, and Day 759.
- Geometric mean fold rise (GMFR) of SARS-CoV-2-specific nAb relative to Day 1 on Day 29, Day 57, Day 209, Day 394, and Day 759.
- Quantified levels or GMT of S protein-specific binding antibody (bAb) on Day 1, Day 29, Day 57, Day 209, Day 394, and Day 759.
- GMFR of S protein-specific bAb relative to Day 1 on Day 29, Day 57, Day 209, Day 394, and Day 759.

3.3. Exploratory Endpoints

The exploratory endpoints of the study are:

- The VE of mRNA-1273 on the viral infection kinetics as measured by viral load at SARS-CoV-2 infection diagnosis by RT-PCR and number of days from the estimated date of SARS-CoV-2 infection until undetectable SARS-CoV-2 infection by RT-PCR
- The VE of mRNA-1273 to reduce duration of COVID-19 symptoms
- The VE of mRNA-1273 against BOD based on the post SARS-CoV-2 infection follow-up
- The VE of mRNA-1273 against all-cause mortality

- The genetic and/or phenotypic relationships of isolated SARS-CoV-2 strains to the vaccine sequence
- Immune response markers after dosing with IP as correlates of risk of COVID-19 and as correlates of risk of SARS-CoV-2 infection
- Additional analyses related to furthering the understanding of SARS-CoV-2 infection and COVID-19, including analyses related to the immunology of this or other vaccines, detection of viral infection, and clinical conduct

4. Study Design

4.1. Overall Study Design

This is a two-part Phase 3 study: Part A and Part B. Participants in Part A, the Blinded Phase of this study are blinded to their treatment assignment. Given that the primary efficacy endpoint for mRNA-1273 against COVID-19 was met per the protocol-defined interim analysis, Part B, the Open-Label Observational Phase of this study is designed to offer participants who received placebo in Part A of this study and who meet Emergency Use Authorization (EUA) eligibility, an option to request open-label mRNA-1273 (Figure 1).

4.1.1. Part A, the Blinded Phase

The Blinded Phase of this study is a randomized, stratified, observer-blind, placebo-controlled evaluation of the efficacy, safety, and immunogenicity of mRNA-1273 SARS-CoV-2 vaccine compared to placebo in adults 18 years of age and older who have no known history of SARS-CoV-2 infection but whose locations or circumstances put them at appreciable risk of acquiring COVID-19 and/or SARS-CoV-2 infection. The study schematic is presented in Figure 1.

Approximately 30,000 participants will be randomly assigned to receive either 100 µg of mRNA-1273 vaccine or a placebo control in a 1:1 randomization ratio. Assignment will be

stratified by age and health risk. This is a case-driven study and thus final study size will depend on the actual attack rate of COVID-19.

The study duration will be approximately 26 months for each participant, including a screening period of up to 1 month and a study period of 25 months that consists of 2 IP injections and 24 months afterward. Participants are considered to have completed the study if they complete the final visit at Day 759 (Month 25), 24 months following the last injection of IP. All participants will be assessed for efficacy and safety endpoints and provide a NP swab sample and blood sample before the first and second injection of IP in addition to a series of post-dose blood samples for immunogenicity through 24 months after the second injection of IP. Efficacy assessments will include surveillance for COVID-19 with RT-PCR confirmation of SARS-CoV-2 infection after the first injection of IP.

Participants will use an electronic diary (eDiary) to report solicited adverse reactions (ARs) after each injection of IP and to prompt an unscheduled clinic visit if experiencing any symptoms of COVID-19. Surveillance for COVID-19 will be performed through weekly contacts with the participants via a combination of telephone calls and completion of an eDiary starting at Day 1 through the end of the study. An illness visit will be scheduled if the participant is experiencing COVID-19 symptoms.

Figure 1 Study Flow Diagram: Part A, the Blinded Phase followed by Part B, the Open-Label Observational Phase

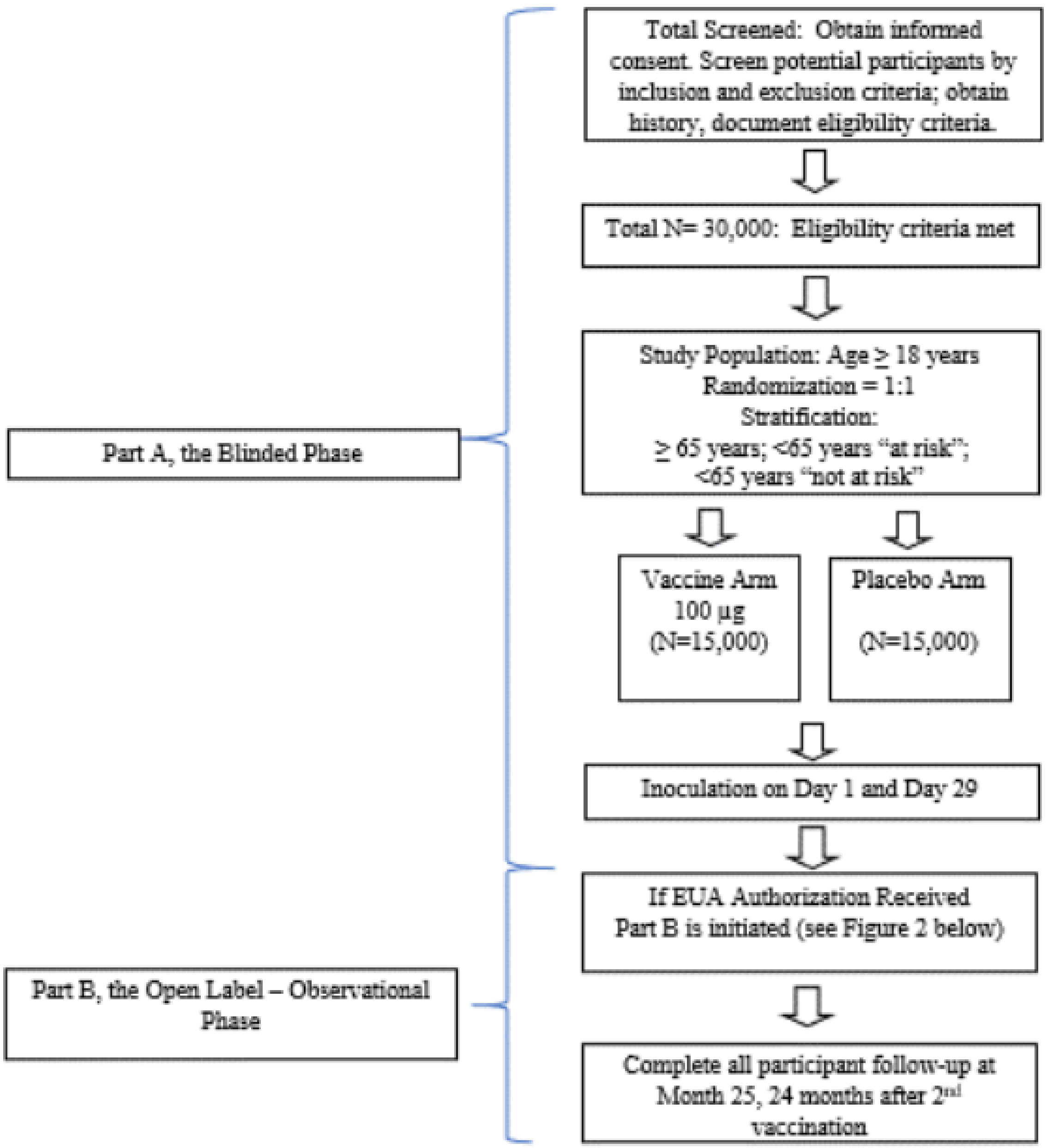

4.2. Statistical Hypothesis

For the primary efficacy objective, the null hypothesis of this study is the VE of mRNA-1273 to prevent first occurrence of COVID-19 is ≤ 30%, i.e. $H_0^{efficacy}$: VE ≤ 0.3.

The study will be considered meeting the primary efficacy objective if the confidence interval (CI) of VE rules out 30% at either one of the interim analyses or at the primary analysis.

4.3. Sample Size and Power

The sample size is driven by the total number of cases to demonstrate the VE (mRNA-1273 vs. placebo) to prevent COVID-19. Under the assumption of proportional hazards over time and with 1:1 randomization to vaccine: placebo, a total of 151 COVID-19 events will provide 90% power to detect a 60% reduction in hazard rate, rejecting the null hypothesis H_0: VE ≤ 30%, with 2 interim analyses at 35% and 70% of the total events using a 1-sided O'Brien-Fleming boundary for efficacy and a log-rank test statistic with a 1-sided false positive error rate of 0.025. The total number of cases pertains to the Per-Protocol (PP) population, starting 14 days after the second injection of IP (refer to the protocol Section 9.3). Under the specified assumptions, up to approximately 30,000 participants will be randomized. It will take approximately 5, 8, and 10 months from study start (first subject first dose), respectively, to accrue 35% (approximately 53), 70% (approximately 106) and 100% (151) of the target number of cases in the PP Set.

For the secondary objective on the VE against serologically confirmed SARS-CoV-2 infection or COVID-19 regardless of symptomology or severity (COV-INF), the study will have ≥ 90% power to demonstrate the VE is above 30% (to reject null hypothesis VE ≤ 30%) at 1-sided alpha of 2.5% if the true VE to prevent COV-INF is 60%, because every COVID-19 disease endpoint is necessarily a COV-INF endpoint. For the secondary objective on the VE against severe COVID-19, the power depends on the number of severe COVID-19 cases (Table 7 in the protocol).

4.4. Randomization

Approximately 30,000 participants will be randomly assigned in 1:1 ratio to receive either mRNA-1273 100 µg or placebo. The randomization will be in a blinded manner using a centralized Interactive Response Technology (IRT) at the Day 1 visit, in accordance with pre-generated randomization schedules. Participants will be stratified by age (≥ 18 and < 65, or ≥ 65 years) and, if they are < 65 years of age, based on the presence or absence of risk factors for severe illness from COVID-19 based on recommendation by the US Centers for Disease Control and Prevention (CDC) as of March 2020. There will be 3 strata for randomization:

- ≥ 18 and < 65 years and not at risk
- ≥ 18 and < 65 years and at risk
- ≥ 65 years

At least 25% of enrolled participants, but not to exceed 50%, will be from the ≥ 65 years age group and < 65 years at risk group.

4.5. Blinding and Unblinding

This study is an observer-blind study. The investigator, study staff, study participants, site monitors, and Sponsor personnel (or its designees) will be blinded to the IP administered until study end, with the certain exceptions specified in Section 6.2.8 of the protocol.

The Sponsor Biostatistics department or designee will generate the randomized allocation schedule(s) for study treatment assignment. Randomization will be implemented via an IRT.

Planned interim and primary analyses are described in Section 6.4.1 of this SAP and Section 9.6 of the protocol. Participant-level unblinding will be restricted to an independent unblinded statistician and, as needed, statistical programmer(s) performing the IAs, who will have no other responsibilities associated with the study.

In addition to the routine study monitoring outlined in this protocol, an external DSMB will review interim data to safeguard the interests of clinical study participants and to enhancing the integrity of the study. The DSMB will review treatment-level results of the IAs, provided by the independent unblinded statistician. Limited additional Sponsor personnel may be unblinded to the treatment-level results of the IAs, if required, in order to act on the recommendations of the DSMB. The extent to which individuals are unblinded with respect to results of IAs will be documented. Depending on the recommendation of the DSMB, the Sponsor may prepare a regulatory submission after an IA. In this case, pre-identified Sponsor members including the analysis and reporting team will be unblinded to treatment assignments and remain unblinded for the remainder of the study. The limited Sponsor and CRO team members to be unblinded in this case will be pre-specified in the study Data Blinding Plan. Participants and investigators will remain blinded until the unblinding or participant decision visit.

5. Analysis Populations

The following analysis sets are defined: Randomization Set, Full Analysis Set, Modified Intent-to-Treat, Per-protocol Set, Immunogenicity Subset, Solicited Safety Set, and Safety Set.

5.1. Randomization Set

The Randomization Set consists of all subjects who are randomized, regardless of the participant's treatment status in the study. Participants will be analyzed according to the treatment group to which they were randomized.

5.2. Full Analysis Set

The Full Analysis Set (FAS) consists of all randomized participants who received at least one dose of IP. Participants will be analyzed according to the treatment group to which they were randomized.

5.3. Modified Intent-to-Treat Set

The Modified Intent-to-Treat (mITT) Set consists of all participants in FAS who had no immunologic or virologic evidence of prior COVID-19 (ie, negative NP swab test and/or bAb against SARS-CoV-2 nucleocapsid below limit of detection [LOD] or lower limit of quantification [LLOQ]) at Day 1 before the first dose of IP, i.e. all FAS participants excluding those with PCR and/or serology positive at baseline (Day 1 before the first dose of IP).

Participants will be analyzed according to the treatment group to which they were randomized.

5.4. Per-Protocol Set

The Per-protocol Set consists of all participants in mITT who received planned doses of IP per schedule and have no major protocol deviations, as determined and documented by Sponsor prior to database lock (DBL) and unblinding, which impact critical or key study data. Participants will be analyzed according to the treatment group to which they were randomized. Participants who did not receive the second injection of IP within [21, 42] days after the first injection date will be excluded from the Per-Protocol Set.

5.5. Immunogenicity Subset and Analysis Populations for Immunogenicity

For characterizing immunogenicity of the vaccine, and for assessing correlates of risk and protection, a case-cohort sampling design will be used for measuring bAb and nAb data from a randomly sampled subset of trial participants [the case-cohort Immunogenicity Analysis Set (ccIAS)], or Immunogenicity SubSet. The ccIAS cohort consists of a stratified random sample of trial participants (Random SubCohort), augmented with a subset or all primary endpoint cases and SARS-CoV-2 infection endpoint cases. The immunogenicity samples of the ccIAS cohort will be processed and the immunogenicity data will be available for the ccIAS cohort. The Immunogenicity Subset consists of all

participants in the ccIAS who had a valid baseline immunogenicity test result (prior to the first injection of IP) and at least one valid post-baseline result.

The analysis of characterizing immunogenicity of mRNA-1273 is included in Section 6.7 of this SAP, the primary analysis population to characterize immunogenicity of mRNA-1273 is the Per-Protocol Random Subcohort for Immunogenicity (Section 6.7.2); the analysis of assessing correlates of risk and protection will be included in a separate Immune Correlates SAP. Details of the case-cohort sampling, and analysis populations for characterizing immunogenicity of mRNA-1273 are also included in Section 6.7 of this SAP.

5.6. Solicited Safety Set

The Solicited Safety Set consists of randomized participants who received at least one dose of IP and contributed any solicited AR data, i.e. have at least one post-baseline solicited safety (eDiary) assessment. The Solicited Safety Set will be used for the analyses of solicited ARs and participants will be included in the treatment group corresponding to the study vaccination they actually received.

In addition, the following Solicited Safety Set is defined for each injection separately. The First (Second) Injection Solicited Safety Set consists of all participants in the Solicited Safety Set who have received the first (second) study injection and have contributed any solicited AR data (eDiary) from the time of first (second) study injection through the following 6 days.

Participants will be analyzed according to the treatment group a subject received, rather than the vaccination group to which the participant was randomized. If a participant was randomized to placebo but received any dose of mRNA-1273 vaccine at any injection, the participants will be included in the mRNA-1273 group in the Solicited Safety Set.

5.7. Safety Set

The Safety Set consists of all randomized participants who received at least one dose of IP. The Safety Set will be used for all analysis of safety except for the solicited ARs. Participants will be included in the vaccination group corresponding to the IP they actually received. For a participant who was randomized to placebo but received any dose of mRNA-1273 at any injection, the participant will be included in the mRNA-1273 group in the Safety Set.

6. Statistical Analysis

6.1. General Considerations

The Schedule of Events is provided in the protocol Table 16-19.

Continuous variables will be summarized using the following descriptive summary statistics: the number of subjects (n), mean, standard deviation (SD), median, minimum (min), and maximum (max).

Categorical variables will be summarized using counts and percentages.

Baseline value, unless specified otherwise, is defined as the most recent non-missing measurement (scheduled or unscheduled) collected before the first dose of IP.

For the summary statistics of all numerical variables unless otherwise specified, the display precision will follow programming standards. Please see Appendix A for variable display standards.

When count data are presented, the percentage will be suppressed when the count is zero in order to draw attention to the non-zero counts. A row denoted "Missing" will be included in count tabulations where specified on the shells to account for dropouts and missing values. The denominator for all percentages will be the number of subjects in that baseline SARS-CoV-2 serostatus and treatment group within the analysis set of interest, unless otherwise specified.

Baseline SARS-CoV-2 status is determined by using virologic and serologic evidence of SARS-CoV-2 infection on or before Day 1.

Positive SARS-CoV-2 status at Baseline is defined as a positive RT-PCR test for SARS-CoV-2, and/or a positive serology test based on bAb specific to SARS-CoV-2 nucleocapsid (as measured by Roche Elecsys Anti-SARS-CoV-2 assay) on or before Day 1.

Negative status at Baseline is defined as a negative RT-PCR test for SARS-CoV-2 and a negative serology test (as measured by Roche Elecsys Anti-SARS-CoV-2 assay) at Day 1.

The baseline SARS-CoV-2 status is defined as missing for subjects with both tests missing, or with one test missing and one test negative.

Subjects with negative Baseline SARS-CoV-2 status will be included in the mITT population; subjects with positive or missing baseline SARS-CoV-2 status will be excluded from the mITT and PP population.

Age: unless otherwise specified, age is calculated as the age at screening. In subgroup analyses on age, age at screening will be used for derivation of age group.

Study day relative to the first injection will be calculated as below:

a) study day prior to the first injection will be calculated as: date of assessment/event – date of the first injection;

b) study day on or after the date of the first injection will be calculated as: date of assessment/event – date of the first injection + 1;

Study day relative to the most recent injection will be calculated as below:

a) study day prior to the first injection will be calculated as: date of assessment/event – date of the first injection;

b) study day on or after the date of the first injection but before the second injection (if applicable) will be calculated as: date of assessment/event – date of the first injection + 1;

c) study day on or after the date of the second injection will be calculated as: date of assessment/event – date of the second injection + 1; if study day is on the same day as the second injection, date and time will be compared with the second injection date and time. If it is prior to the second injection, then study day is calculated as b); If it is after the second injection or the time is missing or not available then study day is calculated as: date of assessment/event – date of the second injection + 1.

Unscheduled visits: Unscheduled visit measurements will be included in analysis as follows:

- In scheduled visit windows per specified visit windowing rules.
- In the derivation of baseline measurements.
- In the derivation of maximum/minimum values after 1st dose and maximum/minimum change from baseline values for safety analyses.
- In individual subject data listings as appropriate.

Visit windowing rules: The analysis visit windows for protocol-defined visits are provided in Appendix B.

Incomplete/missing data:

- Imputation rules for missing dates of prior/concomitant medications, non-study vaccinations and procedures are provided in Appendix C.
- Imputation rules for missing AE dates are provided in Appendix D.
- If the laboratory results are reported as below the LLOQ (e.g. < 0.1), the numeric values will be substituted by 0.5 × LLOQ in the summary when treating the results as continuous variables. If the laboratory results are reported as greater than the ULOQ (e.g. > 3000), the numeric values will be substituted by ULOQ in the summary statistics for continuous variable.
- Other incomplete/missing data will not be imputed, unless specified otherwise.

Treatment groups

The following treatment groups will be used for summary purposes in Part A:

- mRNA-1273
- Placebo

If a subject received any dose of mRNA-1273 at any injection, regardless of the treatment group the subject was randomized to, the subject will be included to mRNA-1273 100 µg group as the actual treatment group received for safety analyses.

All analyses and data summaries/displays for disposition, baseline demographics and characteristics will be provided by Baseline SARS-CoV-2 Status (negative, positive, and overall) and treatment group unless otherwise specified. All analyses and data summaries/display for efficacy will be provided by treatment group using appropriate analysis population unless otherwise specified.

Analysis Periods

The following analysis periods and treatment groups will be used for efficacy analyses for the blinded phase (Part A):

Part A Group	Description	Blinded/Part A Analysis Period for Efficacy
mRNA-1273	Participants randomized to mRNA-1273 in the Blinded Phase	From randomization to earlier date of Participant Decision Visit (PDV)/unblinding, study discontinuation, study completion, death, or data cutoff date for efficacy)
Placebo	Participants randomized to Placebo in the Blinded Phase	

The analysis period and treatment groups of the blinded phase (Part A) for Safety analyses is summarized below:

Part A Group	Description	Blinded/Part A Analysis Period for Safety

mRNA-1273	Participants received at least one dose of mRNA-1273 in the Blinded Phase	From the first dose of study IP to the earlier date of (study discontinuation, study completion, death, data cutoff for safety, PDV/unblinding) for those who did not receive study IP in Part B.<br>For participants received study IP in Part B, from the first dose of study IP in study to the earlier date of (data cutoff for safety, or 1st dose of study IP in Part B)
Placebo	Participants only received Placebo in the Blinded Phase	

The above analysis period for safety analysis in Part A is also referred to as Overall Period in Part A. For Part A, up to 28 days after any vaccination, starts at the day of each vaccination and continue through the earliest date of (the day of each vaccination and 27 subsequent days, next vaccination [if applicable]), will be used as the primary analysis period for safety analyses including unsolicited AE, except for solicited AR, unless specified otherwise.

Part B

Considering Part B/unblinded phase of the study, the following analysis periods may be used. SAP Part B will provide more details, and if the definition for Part B in SAP Part B differs from that described in this SAP, SAP Part B will prevail.

The following analysis period and treatment groups for Efficacy of the unblinded phase (Part B) may be used:

Part B Cohort	Description	Part B Analysis Period for Efficacy
mRNA-1273	Participants randomized to mRNA-1273 in the Blinded Phase	From PDV/unblinding to earlier date (data cutoff date for efficacy, last observed in Study) where last observed is earlier of (study discontinuation, study completion, death)
Placebo	Participants randomized to Placebo in the Blinded Phase and did not receive mRNA-1273 in Part B	
Placebo-mRNA-1273	Participants randomized to Placebo in the Blinded phase and received mRNA-1273 in Part B	

The following analysis period of the unblinded phase (Part B) and treatment groups for Safety analysis of the unblinded phase (Part B) may be used:

Part B Cohort	Description	Part B Analysis Period for Safety
mRNA-1273	Participants received at least one dose of mRNA-1273 in the Blinded Phase	From PDV/unblinding to earlier date of (data cutoff date for safety, last observed in Study). If a participant received mRNA-1273 in Part B, from 1st date of study IP in Part B to earlier date of (data cutoff date for safety, last observed in Study).
Placebo	Participants only received Placebo in the Blinded Phase and did not receive mRNA-1273 in Part B	From PDV/unblinding to earlier date of (data cutoff date for safety, last observed in Study)
Placebo-mRNA-1273	Participants only received Placebo in the Blinded phase and received mRNA-1273 in Part B	From first dose of mRNA-1273 in Part B to earlier date of (data cutoff date for safety, last observed in Study)

All analyses will be conducted using SAS Version 9.4 or higher unless otherwise specified.

6.2. Background Characteristics

6.2.1. Subject Disposition

The number and percentage of subjects in the following categories (analysis sets defined in Section 5) will be summarized as defined in Section 6.1 based on Randomization Set:

- Randomization Set
- Full Analysis Set
- Modified Intent-to-Treat Set
- Per-protocol Set
- Solicited Safety Set
- Safety Set

The denominators of the percentages will be based on subjects in the Randomization Set. The number of subjects in the following categories will be summarized based on subjects screened:

- Number of subjects screened
- Number and percentage of screen failure subjects and the reason for screen failure

The percentage of subjects who screen failed will be based on the number of subjects screened. The reason for screen failure will be based on the number of subjects who screen failed.

The number and percentage of randomized subjects will be summarized by site and by stratification factor at randomization (i.e. ≥ 18 and < 65 years and not at risk, ≥ 18 and < 65 years and at risk, or ≥ 65 years) separately based on the Randomization Set.

The number and percentage of subjects in each of the following disposition categories will be summarized based on the Randomization Set:

- Received each dose of IP
- Prematurely discontinued before receiving the second dose of IP and the reason for discontinuation
- Completed study
- Prematurely discontinued the study and the reason for discontinuation

Subjects with any inclusion and exclusion criteria violation will be provided in a listing.

6.2.2. Demographics and Baseline Characteristics

Descriptive statistics will be calculated for the following continuous demographic and baseline characteristics: age (years), weight (kg), height (cm), body mass index (BMI) (kg/m^2)). The number and percentage of subjects will be provided for categorical variables such as age group, sex, race, ethnicity, randomization stratum under which the subject was randomized, and risk factors at Screening (have at least one of the following):

- Chronic lung disease (e.g., emphysema and chronic bronchitis, idiopathic pulmonary fibrosis, and cystic fibrosis) or moderate to severe asthma
- Significant cardiac disease (e.g., heart failure, coronary artery disease, congenital heart disease, cardiomyopathies, and pulmonary hypertension)
- Severe obesity (body mass index $\geq$ 40 kg/m^2)
- Diabetes (Type 1, Type 2 or gestational)
- Liver disease
- Human Immunodeficiency Virus (HIV) infection

The summaries will be provided separately based on the FAS, Safety Set, mITT Set and PP Set.

6.2.3. Medical History

Medical history data will be coded by system organ class (SOC) and preferred term (PT) using the Medical Dictionary for Regulatory Activities (MedDRA).

The number and percentage of participants with any medical history will be summarized by SOC and PT based on Safety Set. A participant will be counted only once for multiple events within each SOC and PT. SOC will be displayed in internationally agreed order. PT will be displayed in descending order of frequency of mRNA-1273 and then alphabetically within SOC.

6.2.4. Prior and Concomitant Medications

Prior and concomitant medications and non-study vaccinations will be coded using the WHO drug dictionary (WHODD). The summary of concomitant medications will be based on the Safety set. Categorization of prior, concomitant, and post medications is summarized in Appendix C Table 11 and will be summarized.

The number and percentage of subjects who continued or newly received concomitant medications and non-study vaccinations will be summarized by treatment group by PT in descending frequency in the mRNA-1273 group:

- Any concomitant medications
- Any non-study vaccine
- Antipyretics or analgesics medication, summaries will be provided for during the 7-day follow-up period of each injection, and during the 28 days follow-up period after each injection (for 1st injection, up to 2nd injection or 28 days after the 1st injection)

6.2.5. Study Exposure

Number and percentage of subjects receiving 1st and 2nd injections will be summarized by treatment arm, the timing of the 2nd injection will be summarized by arm.

6.2.6. Major Protocol Deviations

Major protocol deviations are a subset of protocol deviations that may significantly impact the completeness, accuracy, or reliability of the study data or that may significantly affect a subject's rights, safety, or well-being. Major protocol deviations rules will be developed and finalized before database lock.

The number and percentage of the subjects with each major protocol deviation type will be provided by treatment group as defined in Section 6.1 based on the Randomization Set.

Select major protocol deviations impact critical or key study data, and subjects with such deviations will be excluded from the Per-Protocol Set for efficacy analyses, such major protocol deviations will be determined and documented by Sponsor prior to DBL and unblinding. Reasons of exclusion from Per-Protocol Set for efficacy will be summarized.

6.3. Efficacy Analysis

The PP Set is the primary analysis population for efficacy analyses, unless otherwise specified. PP Set, mITT Set, and FAS will be used for efficacy analyses and subjects will be included in the treatment group which they are randomized to.

Surveillance for COVID-19 will be performed starting after enrollment through the end of the study.

An Adjudication Committee (AC) blinded to treatment assignment will review potential cases and adjudicate COVID-19, the primary efficacy endpoint, and severe COVID-19. Adjudication committee assessments have been used in the two efficacy analyses that have been performed to date: the first interim analysis based on data snapshot occurred on 11-Nov-2020, the primary analysis based on data snapshot occurred on 25-Nov (please refer to Section 6.4.2). Adjudication committee assessments will be used in the final efficacy analysis of the blinded data (Section 6.4.3).

Baseline SARS-CoV-2 status is described in Section 6.1. Baseline SARS-CoV-2 status, RT-PCR test results at baseline, serology status at baseline (bAb specific to SARS-CoV-2 nucleocapsid [NP], as measured by Roche Elecsys Anti-SARS-CoV-2 assay) will be summarized by treatment group.

The mITT and PP Sets will only include participants with baseline negative SARS-CoV-2 status.

RT-PCR test results, and bAb specific to SARS-CoV-2 NP will be summarized by visit.

6.3.1. Analysis of the Primary Efficacy Endpoint

6.3.1.1. Primary Efficacy Endpoint Definition/Derivation

The primary efficacy objective is the VE of mRNA-1273 to prevent occurrence of COVID-19 starting 14 days after the second dose of IP. COVID-19 is defined as symptomatic disease based on the criteria specified in Section 8.1.1 of the protocol. Cases

are defined as participants meeting clinical criteria based both on symptoms for COVID-19 and positive RT-PCR test results.

Subjects reporting COVID-19 symptoms, as defined in Section 8.1.2 of the protocol, will be asked to use an eDiary to report COVID-19 symptoms, and an Illness Visit will be scheduled to collect an NP swab if COVID-19 symptoms are reported (refer to Section 8.1.2 of the protocol).

For the primary efficacy endpoint, a COVID-19 case will be identified as a positive post-baseline RT-PCR test result that is prompted by symptom(s), together with eligible symptoms, i.e. a positive PCR result of the eligible symptoms summarized below in Table 1.

An independent Adjudication Committee (AC) blinded to treatment assignments will review potential cases and adjudicate COVID19 and severe COVID-19 events and the date of COVID-19 and severe COVID-19.

Two sets of COVID-19 will be provided:

- Based on Adjudication Committee assessments
- Based on eligible COVID-19 symptoms (at least 2 systemic symptoms or 1 respiratory symptom) and positive RT-PCR.

Table 1 Derivation for COVID-19 (primary efficacy endpoint)

	**COVID-19**
Post-baseline PCR results at illness visits prompted by symptom(s)	Positive, **AND**
Systemic Symptoms	at least **TWO** of the following **systemic symptoms**: Fever (≥ 38ºC), chills, myalgia, headache, sore throat, new olfactory and taste disorder(s); **OR**

Respiratory symptoms	at least **ONE** of the following **respiratory** signs/symptoms: cough, shortness of breath or difficulty breathing, OR clinical or radiographical evidence of pneumonia;

The date of documented COVID-19 (case) will be the later date of ([2 eligible systemic symptoms reported, or 1 eligible respiratory symptom reported] and, [date of positive PCR test]). Specifically, the date of documented COVID-19 will be the later date of the following two dates (date of positive PCR test, and the date of eligible symptom(s)), and the two dates should be within 14 days of each other.

- Date of positive PCR test,
- Date of eligible symptom(s), defined as earliest of
    - Respiratory symptom: earliest date of an eligible respiratory symptom is reported
    - Systemic symptoms: earliest date of the 2nd eligible systemic symptom is reported

The time to the first occurrence of COVID-19 will be calculated as:

Time to the 1st occurrence of COVID-19 = Date of documented COVID-19 – Date of randomization +1.

In the primary analysis approach for the primary efficacy endpoint, cases will be counted starting 14 days after the 2nd injection, i.e. date of documented COVID-19 － Date of the 2nd injection ⩾ 14 (starting 14 days after the 2nd injection).

In the primary analysis approach for the primary efficacy endpoint, the first occurrence of COVID-19 starting 14 days after the 2nd injection will be considered event. In the various sensitivity analyses planned to examine the onset of COVID-19, the censoring rules of cases are outlined in Table 2 below.

In this study, during the blinded phase, there is a scheduled NP swab test at the Day 29 visit prior to the 2nd injection of IP; and blood samples are collected with bAb against SARS-CoV-2 nucleocapsid (NP) tested at months 1, 2, 7, 13 and 25. At IA1 and the Primary Analysis of efficacy, RT-PCR results at scheduled pre-dose 2 (Day 29) and bAb against SARS-CoV-2 nucleocapsid at Day 29 were not considered in the derivation of COVID-19.

At the final blinded efficacy analysis, subjects with early infection, as detected by positive RT-PCR at scheduled pre-dose 2 visit (Day 29), or positive bAb against SARS-CoV-2 NP in blood samples at the scheduled visits, are censored at the time of early infection. If a participant had eligible symptom(s) within 14 days of positive RT-PCR at scheduled pre-dose 2 visit, the participant is considered to be a case for COVID-19.

6.3.1.2. Primary Analysis Approach

The number and percentage of subjects who had an event (i.e. the first occurrence of COVID-19 starting 14 days after the second injection, date of documented COVID-19 – date of the second injection ≥ 14) and subjects who were censored will be summarized.

The non-parametric Kaplan-Meier method will be used to estimate the time to first occurrence of COVID-19 curve in each treatment group.

Vaccine efficacy (VE) is defined as the percent reduction in the hazard of the primary endpoint (mRNA-1273 vs. placebo), i.e. one minus the hazard ratio (HR). The null hypothesis is:

$$H_0^{efficacy}\text{: vaccine efficacy} \leq 0.3$$

Equivalently, the null hypothesis is:

$$H_0^{efficacy}\text{: hazard ratio (HR)} \geq 0.7$$

A stratified Cox proportional hazard (PH) model with Efron's method of tie handling and with treatment group as covariate will be used to assess the magnitude of the treatment group difference (i.e. HR) between mRNA-1273 vs. placebo. The same stratification

factors used for randomization (Section 4.4) will be applied to the stratified Cox model. VE with corresponding alpha-adjusted confidence interval (CI), 95% CI, and one-sided *p*-value for testing the null hypothesis from the stratified Cox model would be reported at a planned interim analysis up to the analysis at which vaccine efficacy is demonstrated. After vaccine efficacy was demonstrated, VE and 95% CI would be provided for subsequent analysis/updates of efficacy.

In this study, vaccine efficacy was demonstrated based on pre-specified statistical criterion (Section 6.4) at the first interim analysis, at which, one-sided p-value for testing the null hypothesis of VE≤ 0.3, 95% CI and alpha-adjusted CI were provided from the stratified Cox model. At the primary analysis where the total number of COVID-19 cases > 151, total target number of cases, VE, one-sided p-value for testing the null hypothesis of VE≤ 0.3 and 95% CI were provided. In subsequent analysis/updates of efficacy, VE and 95% CI will be provided.

For the primary analysis, subjects who have no documented COVID-19 will be censored at the last study assessment date. Subjects who discontinue the study early or die due to causes unrelated to COVID-19 without documented COVID-19 will be censored at the date of early discontinuation or death. Subjects who experience an early COVID-19 up to 14 days after the second injection of IP will be censored at the time of documented COVID-19.

As in PP Set definition, subjects who missed study IP administration, who were seropositive at baseline, or who had major protocol deviations that impact critical or key data, will be excluded from the PP Set.

The number and percentage of subjects who had an event (i.e. the first occurrence of COVID-19 at least 14 days after the second injection) will be reported.

Potential intercurrent events to the estimand are listed in Appendix F Table 9. The primary estimand with rationale for strategies to address intercurrent events is summarized in Appendix F 10.

6.3.1.3. Sensitivity Analyses with COVID-19 Cases Counted Starting at Various Timepoints

Sensitivity analyses will be performed with COVID-19 cases counted starting at various timepoints as below using the PP Set. The censoring rules for analyses of the primary endpoint are summarized in Table 2.

- immediately after the second injection of IP
- 14 days after the first injection of IP (also as primary analysis approach for the secondary efficacy objective: To evaluate the efficacy of mRNA-1273 to prevent COVID-19 after the first dose of IP.)
- immediately after the first injection of IP
- immediately after randomization

If <10% of the COVID-19 cases received the first injection after the day the subject was randomized, sensitivity analysis with COVID-19 cases counted starting after the first injection of IP may not be performed separately, as it would be very similar to the sensitivity analysis with COVID-19 cases counted starting from randomization.

Table 2 Censoring Rules for COVID-19

Situation	Primary Efficacy Approach (cases ≥14 days after Dose 2)	Sensitivity Analyses			
		Cases starting after Dose 2	Cases ≥14 days after Dose 1	Cases starting after Dose 1*	Cases starting after randomization
Early case from randomization up to first injection	Censored at date of case	Censored at date of case	Censored at date of case	Censored at date of case	Event
Early case from Dose 1 up to (<) 14 days after Dose 1	Censored at date of case	Censored at date of case	Censored at date of case	Event	Event
Early case from ≥14 days after Dose 1 up to (<) Dose 2	Censored at date of case	Censored at date of case	Event	Event	Event
Early case from Dose 2 up to (<)14 days after Dose 2	Censored at date of case	Event	Event	Event	Event
Case ≥14 days after Dose 2	Event	Event	Event	Event	Event
Early discontinuation or death without documentation of COVID-19	Censored at date of discontinuation/death				

**Situation**	**Primary Efficacy Approach (cases ≥14 days after Dose 2)**	**Sensitivity Analyses**			
		**Cases starting after Dose 2**	**Cases ≥14 days after Dose 1**	**Cases starting after Dose 1***	**Cases starting after randomization**
Early infection as detected by:<br>positive RT-PCR at scheduled visit (pre-dose 2),<br>or, positive bAb against SARS-CoV-2 NP in blood samples at the scheduled visits	Censored at date of early infection				

* To be performed if there are ≥ 10% of total cases with Dose 1 date > randomization date

Analysis of the primary efficacy endpoint based on the mITT Set will also be performed using the same statistical methods used for the primary analysis. The above sensitivity analyses will be also performed with COVID-19 cases counted starting at various timepoints using the mITT Set.

6.3.1.4. Subgroup Analysis

To assess consistency of VE across various subgroups, subgroup analyses of the primary efficacy endpoint will be performed in select subgroups specified below based on the PP Set. The primary efficacy endpoint will be analysed by each of the subgroups using the same methods described for the primary analysis, i.e. the stratified Cox proportional hazard model with Efron's method of tie handling with a single treatment covariate, and the estimate of VE and its 95% CI will be provided within each category of the following classification variables:

- Age groups: ≥ 18 and < 65 years, ≥ 65 years
- Age groups: ≥ 18 and < 65 years, ≥ 65 years and < 75 years, and, ≥ 75 years
- Stratification factor at randomization: ≥ 18 and < 65 years and not at risk, ≥ 18 and < 65 years at risk, ≥ 65 years
- Sex (female, male)
- Race
- Ethnicity
- Race and Ethnicity: non-Hispanic white, communities of color
- Each risk factor for severe COVID-19 illness as listed in Section 6.2.1 of the protocol

If the number of subjects in certain subgroups are too small, it may be combined with the other subgroups for the subgroup analyses. Forest plots will be provided for VE and its 95% CI for the subgroup analyses based on PP Set. Subgroup analyses based on the mITT Set may be performed.

Recently, new SARS-CoV-2 variants have emerged. COVID-19 cases may be sequenced and depending on the findings, summary of cases genotypically distinct and genotypically similar to vaccine by treatment assignment may be provided. Analysis of vaccine efficacy against variants may be performed if there are sufficient numbers of variants cases.

6.3.1.5. Supportive Analysis Using Exact Method Based on Incidence Rate

Incidence rate will be provided for each treatment group. Incidence rate will be calculated as the number of subjects with an event (i.e. first occurrence of COVID-19 at least 14 days after the second injection) divided by the number of subjects at risk adjusted by person-time (years) in each treatment group. The person-time is calculated as the time from randomization to the date of the first event for subjects with an event, and the time from randomization to the date of censoring for subjects without an event (or who are censored). Person-time, incidence rate, and 95% CI for incidence rate will be provided by treatment group.

As a supportive analysis, VE will also be estimated by one minus the ratio of incidence rate (mRNA-1273 vs. placebo), i.e. 1- ratio of incidence rate (mRNA-1273 vs. placebo) adjusting for person-time, and the 95% CI of VE will be computed using the exact method conditional upon the total number of cases adjusting for person-time.

6.3.2. Analysis of the Secondary Efficacy Endpoints

A sequential/hierarchical testing procedure will be used to control type 1 error rate over the primary efficacy endpoint and the secondary efficacy endpoints. Secondary efficacy endpoints will only be tested when the primary efficacy endpoint achieves statistical significance. Multiplicity adjustments among the secondary efficacy endpoints may be performed for secondary efficacy endpoints as described in Section 6.5.

6.3.2.1.Derivation of Secondary Efficacy Endpoint: Severe COVID-19

Derivation of select secondary efficacy endpoints are included in this section.

6.3.2.1.1. Derivation of Secondary Efficacy Endpoint: Severe COVID-19

Severe COVID-19 is defined as:

To be considered severe COVID-19, the following criteria must be met:

- Confirmed COVID-19 as per the Primary Efficacy Endpoint case definition, AND
- any of the conditions listed in Section 3.2.1 (Section 8.1.1 of the protocol).

The date of documented severe COVID-19 will be the later date of:

- Date of documented COVID-19,
- Date of eligible symptom for severe COVID-19, defined as the earliest of the first eligible severe symptom is reported

The date of eligible symptoms for severe COVID-19 should be within 28 days after the positive RT-PCR result used in the confirmation of COVID-19.

The time to the first occurrence of severe COVID-19 will be calculated as:

Time to the 1st occurrence of severe COVID-19 = Date of documented severe COVID-19 – Date of randomization +1

The independent AC blinded to treatment assignments will adjudicate severe COVID-19 events. Two sets of severe COVID-19 will be provided:

- Based on Adjudication Committee assessments
- Based on eligible symptoms for severe COVID-19, and positive RT-PCR.

6.3.2.1.2. Derivation of a secondary definition of COVID-19

A secondary definition of COVID-19 is defined as any of the listed systemic symptom in Section 8.1.1 of the protocol AND with a positive RT-PCR test.

Date of the documented secondary definition of COVID-19 will be later date of:

- Date of the positive RT-PCR test (prompt by symptom)
- Date of eligible symptom for secondary definition of COVID-19, defined as the earliest date of first eligible symptom is reported

and the two dates should be within 14 days of each other.

The same censoring rule as summarized in Table 2 for the primary definition of COVID-19 will be used for the secondary definition of COVID-19 and corresponding sensitivity analyses with cases starting at different timepoint.

6.3.2.1.3. Derivation of serologically confirmed SARS-CoV-2 infection or COVID-19 regardless of symptomatology or severity

For the secondary efficacy endpoint: serologically confirmed SARS-CoV-2 infection or COVID-19 regardless of symptomatology or severity [COV-INF], any post-baseline positive RT-PCR results will be considered, including those from the scheduled NP swab tests at Day 29 visit prior to the 2nd injection of IP as well as the those prompted by symptom(s). In addition, seroconversion due to infection will also be considered.

The analysis population for COV-INF will be the Per-Protocol Set and the mITT Set that include participants with negative SARS-CoV-2 status at baseline. Seroconversion due to infection is defined for participants with negative SARS-CoV-2 status at baseline as becoming seropositive (positive bAb specific to SARS-CoV-2 NP) as measured by *Roche Elecsys* on study (at scheduled visits post baseline). A COVID-19 case or secondary definition of COVID-19 case will always be an INF case.

The date of documented infection regardless of symptom [COV-INF] will be the earlier of:

- Date of positive post-baseline RT-PCR result, or
- Date of seroconversion due to infection

In the primary approach, documented infection is counted starting 14 days after the 2nd dose, which requires a positive RT-PCR result starting 14 days after the 2nd IP dose, or seroconversion at Day 57 visit or later.

Derivation of this secondary efficacy endpoint is summarized in Table 3.

Table 3 Derivation of Secondary efficacy endpoint: Serologically confirmed SARS-CoV-2 infection or COVID-19 regardless of symptomatology or severity – mITT Set or Per-Protocol Set

**Baseline SARS-CoV-2 Status**	**Post-baseline assessments**		**Secondary endpoint**: serologically confirmed SARS-CoV-2 infection or COVID-19 regardless of symptomatology or severity
	PCR test post baseline	bAb levels against SARS-CoV-2 Nucleocapsid	
Negative at Baseline	**positive** (either at symptom-prompt NP swab test [Illness visit], or at scheduled NP swab test)		Case
Negative at Baseline		Seroconversion (positive on study at scheduled visits) as measured by *Roche Elecsys*	Case

6.3.2.1.4. Derivation of asymptomatic SARS-CoV-2 infection

Asymptomatic SARS-CoV-2 infection will be analyzed using the Per-Protocol Set and mITT Set. Asymptomatic infection is identified by absence of symptoms and infections as detected by RT-PCR or seroconversion. Specifically:

- Absent of symptoms (no COVID-19 symptom for either primary efficacy endpoint of COVID-19, or secondary definition of COVID-19),
- AND at least one from below:
    - seroconversion (bAb specific to SARS-CoV-2 nucleocapsid) at scheduled visits (months 1, 2, 7, 13 and 25 if applicable in Part A, Participant Decision Visit and etc. in Part B), when blood samples for immunogenicity are collected, or
    - by RT-PCR at scheduled visits such as pre-dose 2 at Day 29 in Part A, both RT-PCR test and bAb against SARS-CoV-2 nucleocapsid will be considered.

The date of documented asymptomatic infection is the earlier date of seroconversion due to infection, or positive RT-PCR at scheduled visits, with absence of symptoms. Participants who had a symptomatic infection (COVID-19 or secondary definition of COVID-19) prior to an asymptomatic infection will be censored at the time of symptomatic infection for the analysis of asymptomatic infection. In the primary approach, documented asymptomatic infection is counted starting 14 days after the 2nd IP dose, which requires seroconversion at months 2 (Day 57 visit) or later.

6.3.2.2. Analysis of the secondary efficacy endpoints

Similar analysis methods as for the primary efficacy endpoint will be applied to the following secondary efficacy endpoints based on the PP set, unless otherwise specified:

- VE to prevent severe COVID-19
- VE to prevent serologically confirmed SARS-CoV-2 infection or COVID-19 regardless of symptomatology or severity.

- VE to prevent COVID-19 using a secondary definition of symptoms
- VE to prevent death caused by COVID-19

For each of the above secondary objectives, the following analyses will be performed:

- Primary analysis: VE will be estimated with 1 - HR (mRNA-1273 vs. placebo) using a Cox PH model based on the PP Set as described for the primary efficacy endpoint. Kaplan-Meier curves of time to event will be presented for each treatment group.

  Cases will be counted starting 14 days after the second injection of IP.

- Analysis using the same model based on the mITT Set as described for the primary efficacy endpoint. Kaplan-Meier curves of time to event will be presented for each treatment group.
- Sensitivity analyses with cases counted starting immediately after the second injection of IP (onset date >= date of dose 2, considering positive RT-PCR at Day 29), starting 14 days (>= 14 days) after the first injection of IP, and immediately after randomization respectively.
- VE and 95% CI based on the incidence will be estimated with 1 - ratio of incidence rates using the exact method conditional upon the total number of cases.

<u>For the secondary efficacy objective: VE to prevent asymptomatic SARS-CoV-2 infection</u>

As diseased cases (COVID-19 or secondary definition of COVID-19) are competing events for asymptomatic SARS-CoV-2 infections, competing risk method will be used to estimate the vaccine efficacy of mRNA-1273, specifically, Fine and Gray's (FG) sub-distribution hazard model will be used. Competing risk method will also be used to estimate the cumulative incidence function and the cumulative incidence of asymptomatic SARS-CoV-2 infections will be plotted.

In the primary approach with cases of asymptomatic SARS-CoV-2 infection counted starting 14 days after the second injection, only seroconversion at Day 57 or later will be considered.

As serum samples were taken at scheduled visits (e.g. Day 29[month 1], Day 57[month 2]), sensitivity analyses with cases counted starting after 2nd dose, 14 days after first dose, after first dose and after randomization are the same. Only one set of sensitivity analysis with cases counted starting from randomization will be provided. In this sensitivity analysis, asymptomatic cases detected by positive RT-PCR result at scheduled pre-dose 2 visit (Day 29), or seroconversion at timepoints with scheduled visits will be considered. Sensitivity analysis using the mITT Set with cases counted starting from 14 days after the second injection, and from randomization will also be performed.

Stratified Cox PH model may also be used.

For the secondary efficacy objective: VE to prevent COVID-19 after the first injection of IP

This endpoint will be analyzed as a sensitivity analysis of the primary efficacy endpoint with cases counted starting 14 days (>=14 days) after the first injection of IP as described in Section 6.3.1.

For the secondary efficacy objective: VE to prevent COVID-19 disease regardless of prior SARS-CoV-2 infection

This endpoint will be analyzed using the FAS. The same methods described above for the primary efficacy endpoint will be applied with cases counted starting 14 days after the second injection of IP.

Sensitivity analyses with cases counted starting immediately after the second injection, 14 days after the first injection, and after randomization will also be performed. In sensitivity analysis with cases counted starting after the second

injection, subjects who received only the first injection and is a case will be censored at the time of COVID-19.

The VE will also be estimated with 1- ratio of incidence rates with the 95% CI using the exact method conditional upon the total number of cases adjusting for person-time.

In addition, an exploratory analysis with the same Cox model will be carried out in the subgroup of FAS whose baseline SARS-CoV-2 status is positive with cases counted starting from randomization to assess the VE in those with positive baseline SARS-CoV-2 status, at baseline, if sample size permits. Such analysis in the subgroup of FAS whose baseline SARS-CoV-2 status is negative with cases counted starting from randomization is the same as the sensitivity analysis of COVID-19 starting from randomization in mITT.

6.4. Planned Analyses and Analyses of Efficacy Analyses that Have Been Conducted

6.4.1. Planned Interim Analyses and Primary Analysis

There were two planned interim analyses in this study, which would be performed when approximately 35% and 70% of the target total number of COVID-19 cases across the two vaccine groups had been observed respectively. The primary objective of the IAs was for early detection of reliable evidence that VE is above 30%. The Lan-DeMets O'Brien-Fleming approximation spending function was used for calculating efficacy bounds and to preserve the (1-sided) 0.025 false positive error rate over the two IAs and the primary analysis (when the target number of cases have been observed), relative to the null hypothesis ($H_0^{efficacy}$: VE ≤ 30%). There is no intention to stop the study early if the efficacy had been demonstrated at any of the IAs. If efficacy was demonstrated at an IA, the subsequent IA or primary analyses would be considered supportive in nature. The IA results would be reviewed by the DSMB.

The first IA would occur when 35% of the total cases in the PP set had been observed. The study would be considered positive at this IA if the one-sided *p*-value for rejecting VE ≤

30% (HR ≥ 0.7) was less than 0.0002 based on the Lan-DeMets O'Brien-Fleming approximation spending function. This corresponds to an observed HR of approximately 0.259, or an observed VE approximately 0.741.

The second IA would occur when 70% of the total cases have been observed in the PP set. The study will be considered positive at this IA if the *p*-value for rejecting VE ≤ 30% (HR ≥ 0.7) was less than 0.0073 based on the Lan-DeMets O'Brien-Fleming approximation spending function. This corresponds to an observed HR of approximately 0.435, or an observed VE of approximately 0.565.

The primary analysis would be performed when approximately 151 cases have been observed in the PP Set. The study would be considered positive at the primary analysis when a total of 151 cases had been observed and if the one-sided *p*-value for rejecting HR ≥ 0.7 was less than 0.0227. This corresponds to an observed hazard ratio of approximately 0.505 or observed VE of approximately 0.495.

An independent, unblinded statistics team would carry out the IAs. The unblinded statistics team would not be involved in either study design or the regular study conduct. The subjects and study sites will remain blinded throughout the study.

The final analysis would be performed after all subjects have completed the study and after the database is cleaned and locked. Results of this analysis would be presented in a CSR.

The timing, number of cases and decision guidance at each IA and primary analysis is summarized in the table below (Table 13 of the protocol).

Table 4 Interim Boundaries Using O'Brien-Fleming Spending function, Calculation Based on the PP Set for the Primary Efficacy Endpoint

Information fraction (% of total #cases)	Number of cases	Nominal Alpha	Efficacy Boundary Rejecting H0: VE ≤ 30% (HR ≥ 0.7)	Cum Prob (crossing efficacy boundary if the true VE = 60%)
IA1 35%	53	0.0002	VE ≥ 0.741 (HR ≤ 0.259)	4.6%
IA2 70%	106	0.0073	VE ≥ 0.565 (HR ≤ 0.435)	61.5%
Primary analysis 100%	151	0.0227	VE ≥ 0.495 (HR ≤ 0.505)	90.0%

Abbreviations: HR = hazard ratio; IA: interim analysis; PP = per-protocol; VE = vaccine efficacy.

6.4.2. Analyses of Efficacy that Have been Conducted

Two efficacy analyses have been performed in this study as of this SAP amendment.

6.4.2.1.The First Interim Analysis

The primary efficacy endpoint of mRNA-1273-P301 is COVID-19, defined as the first occurrence of COVID-19 starting 14 days after the 2nd injection.

For the primary efficacy objective, the null hypothesis of this study was that the vaccine efficacy (VE) of mRNA-1273 to prevent first occurrence of COVID-19 is ≤ 30%, i.e. $H_0^{efficacy}$: VE ≤ 0.3.

The first interim analysis was conducted based on a data snapshot occurred on 11-Nov-2020 whereas the data cut-off date for efficacy was 07-Nov-2020. At IA1, the median follow-up time was 49 days (7 weeks). The primary analysis of COVID-19 is based on the Per-Protocol (PP) set with cases starting 14 days after the 2nd dose. The IA1 efficacy analysis was performed by the independent biostatistician and the results were first reviewed by the independent external DSMB on 15-Nov-2020. The DSMB concluded that

"with 63% of the expected number of the total number of adjudicated primary endpoints, the study has passed the efficacy monitoring boundary for benefit." The DSMB "encourages the study team to prepare the data for the FDA package as rapidly as possible." Based on the DSMB's recommendation, select Sponsor and CRO personnel were unblinded to prepare for regulatory submissions.

Vaccine efficacy of mRNA-1273 was demonstrated at IA1 based on the pre-specified success criterion on efficacy. As efficacy was demonstrated at IA1 based on 95 cases per adjudication committee, all subsequent efficacy analyses, if any, would be considered supportive or supplementary.

At IA1, the total number of COVID-19 starting 14 days after the 2nd injection based on the adjudication committee assessments (i.e. primary efficacy endpoint) was 95, 63% of the information fraction based on the total target number of 151. Correspondingly, the nominal one-sided alpha is 0.0047. At IA1, the one-sided p-value to test null hypothesis of VE $\leqslant$ 30% from the stratified Cox proportional hazard model was <0.0001, and was compared to the nominal one-sided alpha (0.0047) based on the number of cases at IA1 (95 in total). Equivalently, the lower-bound of the alpha-adjusted confidence interval of VE, the two-sided 99.1% CI, can be compared to 30% at IA1. The lower bound of the two-sided 99.1% CI was above 30% at IA1.

6.4.2.2. The Primary Analysis of Efficacy

To support regulatory submissions, in particular, to provide data with a median follow-up duration for safety and efficacy of at least two months after completion of the full vaccination regimen, a decision was made to perform a second analysis based on the data snapshot as of 25-Nov-2020 (DS2, whereas the data cut-off date for efficacy was 21-Nov-2020). Both safety and efficacy analyses were performed to provide additional information on the vaccine's profile. As efficacy has been demonstrated at IA1, the analysis of efficacy based on the data snapshot of 25-Nov-2020 were supplementary in nature.

The date cutoff date for efficacy in the second analysis of efficacy was 21-Nov-2020. All potential COVID-19 cases starting 14 days after the 2nd injection confirmed by RT-PCR

(central or local), with corresponding minimal clinical data in electronic data capture (EDC) as of 21-Nov-2020 were sent to the Adjudication Committee. All these potential cases have been adjudicated for this analysis.

It was anticipated that the total number of COVID-19 cases based on the adjudication committee assessments starting 14 days after the 2nd injection in the Per-Protocol Set at DS2 on 25-Nov-2020 would be ≥ 151, the target total number of events at the originally planned primary analysis. Therefore, this analysis was considered the primary analysis of efficacy of at least 151 cases, as prespecified in the protocol. At DS2, the total number of COVID-19 cases based on the adjudication committee assessments starting 14 days after the 2nd injection in the Per-Protocol Set was 196.

6.4.3. Final Analysis of Efficacy for blinded phase

Results from DS1 and DS2 were used to support EUA application in US and other regions of the world. EUA was authorized for mRNA-1273 in US on 18-December-2020.

The clinical study protocol (CSP) Amendment 6, dated 23-December-2020, informs all ongoing study participants of the availability of and eligibility criteria of any COVID-19 vaccine made available under an EUA and offers participants who originally received placebo in this study the potential benefit of vaccination against COVID-19, given that the primary efficacy endpoint for mRNA-1273 against COVID-19 was met per the protocol-defined interim analysis. Participants in this study will have a participant decision visit, at which participants would have the opportunity to be unblinded, and those originally randomized to placebo would have the opportunity to receive mRNA-1273 in this study.

A final efficacy analysis is planned to provide final efficacy update based on data from the blinded phase (Part A) of the study when approximately 90% of the study participants have had the participant decision visit or have been unblinded or discontinued from the study. At this final efficacy analysis of the blinded phase of the study, efficacy data up to the earlier of participant decision visit or unblinding visit, or data cutoff date for efficacy will be included in the analysis.

The analysis period of the blinded phase (Part A) for efficacy analyses will be used:

**Part A Treatment Group**	**Description**	**Blinded/Part A Analysis Period for Efficacy**
mRNA-1273	Participants randomized to mRNA-1273 in the Blinded Phase	From randomization to earlier date of (PDV/unblinding, study discontinuation, study completion, death, or data cutoff for efficacy)
Placebo	Participants randomized to Placebo in the Blinded Phase	From randomization to earlier date of (PDV/unblinding, study discontinuation, study completion, death, or data cutoff for efficacy)

At the time of the final analysis of efficacy for blinded phase date, summary of COVID-19 cases occurred during the open-label phase may be provided.

6.5. Multiplicity Adjustments

The overall type I error rate for this study is controlled at 2.5% (one-sided). The overall Type I error rate for the primary efficacy endpoint at the IAs and the primary analysis is strictly controlled at 2.5% (1-sided) based on the Lan-DeMets O'Brien-Fleming approximation spending function. The primary efficacy endpoint will be considered statistically significant after consideration of the strategy for controlling the Type I error as described in Section 6.4 Interim Analyses. Statistical significance of the primary efficacy endpoint can be achieved at either one of the interim analyses or at the primary analysis.
A sequential/hierarchical testing procedure will be used to control type 1 error rate over the primary efficacy endpoint and the secondary efficacy endpoints. Secondary efficacy endpoints will only be tested when the primary efficacy endpoint achieves statistical significance.
If the primary efficacy endpoint achieves statistical significance at either one of the interim analyses or at the primary analysis, a fixed-sequence statistical strategy will be used to test the following secondary efficacy endpoints in a pre-defined order:

1. secondary efficacy endpoint: COVID-19 regardless of evidence of prior SARS -CoV-2 infection at the same analysis (through the same follow-up period)

2. secondary efficacy endpoint: infection regardless of symptomatology or severity at the same analysis (through the same follow-up period)
3. secondary efficacy endpoint: severe COVID-19 at the analysis with ≥ 20 cases, otherwise, to test at the end of the study

If the primary efficacy endpoint achieves statistical significance at either one of the interim analyses or at the primary analysis, sequential tests of the above 3 secondary efficacy endpoints will be performed at one-sided Type I error rate of 0.025.

All hypothesis tests against the above three secondary efficacy endpoints will be first against a VE ≤ 0% null hypothesis. If hypothesis test against VE ≤ 0% are rejected for all three, sequential testing against a VE ≤ 10% for these three secondary efficacy endpoints will be performed in the same order; if hypothesis test against VE ≤ 10% are rejected for all three, sequential testing against a VE ≤ 20% for these three secondary efficacy endpoints will be performed in the same order; if hypothesis test against VE ≤ 20% are rejected for all three, sequential testing against a VE ≤ 30% for these three secondary efficacy endpoints will be performed in the same order. No further testing will be performed once the sequence breaks, that is, further testing stops as soon as an endpoint in the sequence fails to show significance against corresponding null hypothesis.

No further testing will be performed once the sequence breaks, that is, further testing stops as soon as an endpoint in the sequence fails to show significance against corresponding null hypothesis regarding VE.

While achieving vaccine efficacy that rules out a lower bound of 30% against COVID-19 is the goal of the study, there is utility in formally assessing VEs that are less than 30%. Therefore, in the situation that the primary efficacy endpoint (VE against COVID-19) does not achieve statistical significance against a VE ≤ 30% null hypothesis at neither the interim analyses nor the primary analysis, at the primary analysis, sequential testing against a VE ≤ 20%, a VE ≤ 10%, and a VE ≤ 0% will be performed, halting when a rejection occurs. Figure 2 below demonstrates the testing strategy in this study:

Figure 2 Testing Strategy of Primary and Secondary Efficacy Endpoints

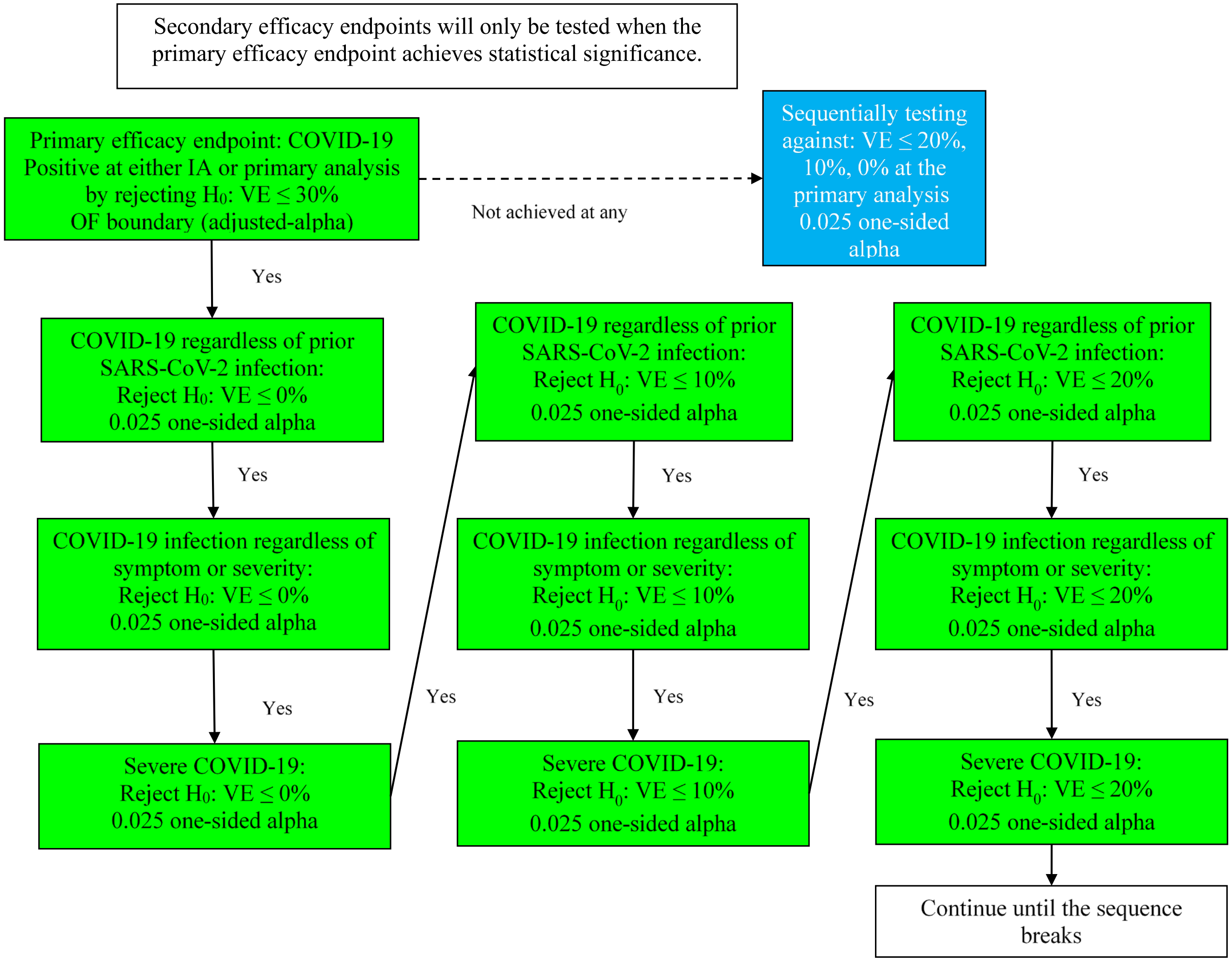
Secondary efficacy endpoints will only be tested when the primary efficacy endpoint achieves statistical significance.
Primary efficacy endpoint: COVID-19 Positive at either IA or primary analysis by rejecting H0: VE ≤ 30% OF boundary (adjusted-alpha)
Not achieved at any
Sequentially testing against: VE ≤ 20%, 10%, 0% at the primary analysis 0.025 one-sided alpha
Yes
COVID-19 regardless of prior SARS-CoV-2 infection: Reject H0: VE ≤ 0% 0.025 one-sided alpha
COVID-19 infection regardless of symptom or severity: Reject H0: VE ≤ 0% 0.025 one-sided alpha
Severe COVID-19: Reject H0: VE ≤ 0% 0.025 one-sided alpha
COVID-19 regardless of prior SARS-CoV-2 infection: Reject H0: VE ≤ 10% 0.025 one-sided alpha
COVID-19 infection regardless of symptom or severity: Reject H0: VE ≤ 10% 0.025 one-sided alpha
Severe COVID-19: Reject H0: VE ≤ 10% 0.025 one-sided alpha
COVID-19 regardless of prior SARS-CoV-2 infection: Reject H0: VE ≤ 20% 0.025 one-sided alpha
COVID-19 infection regardless of symptom or severity: Reject H0: VE ≤ 20% 0.025 one-sided alpha
Severe COVID-19: Reject H0: VE ≤ 20% 0.025 one-sided alpha
Continue until the sequence breaks

At the first interim analysis where vaccine efficacy for COVID-19 has been demonstrated at the pre-specified statistical criterion, there were 11 total severe COVID-19 cases based on adjudication committee starting 14 days after the 2nd injection.
At the time of IA1 and the primary analysis of efficacy, data for secondary efficacy endpoint infection regardless of symptomatology or severity were not available and were not included in these analyses.

6.6. Safety Analysis

Safety and reactogenicity will be assessed by clinical review of all relevant parameters including solicited ARs (local and systemic), unsolicited AEs, SAEs, MAAEs, AEs leading to withdrawal from study vaccine and/or study participation, vital signs, and physical examination findings. Solicited ARs and unsolicited AEs will be coded by SOC and PT according to the MedDRA. The Toxicity Grading Scale for Healthy Adult and Adolescent Volunteers Enrolled in Preventative Vaccine Clinical Trials (DHHS 2007) is used in this study with modifications for solicited ARs as presented in Table 4 of the study protocol.

All safety analyses will be based on the Safety Set, except summaries of solicited ARs which will be based on the Solicited Safety Set. All safety analyses will be provided by treatment group, unless otherwise specified. Unless specified otherwise, the language in this section pertains to Part A.

6.6.1. Unsolicited Treatment-emergent Adverse Events

A treatment-emergent AE (TEAE) is defined as any event occurring during the study not present before exposure to the IP or any event already present that worsens after exposure to study vaccine. Worsening of a pre-existing condition after vaccination will be reported as a new AE.

Adverse events will also be evaluated by the investigator for the coexistence of MAAE which is defined as an AE that leads to an unscheduled visit to a healthcare practitioner.

Unsolicited AEs will be coded by PT and SOC using MedDRA and summarized by vaccination group.

Unsolicited AEs will be collected for up to 28 days after each IP dose; SAEs, MAAEs, AEs leading to withdrawal will be collected throughout the study. Analyses of unsolicited AE will be provided for up to 28 days after any vaccination unless otherwise specified; and select analyses of unsolicited AE will be provided for Surveillance stage, and overall stage (Section 6.1).

All summary tables (except for the overall summary of AEs) for unsolicited AEs will be presented by SOC and PT or by PT only for TEAEs with counts of subjects included. SOC will be displayed in internationally agreed order. PT will be displayed in descending order of frequency of mRNA-1273 and then alphabetically within SOC. When summarizing the number and percentage of subjects with an event, subjects with multiple occurrences of the same AE or a continuing AE will be counted once. Only the maximum severity level will be presented in the severity summaries, and the strongest relationship level will be presented in the relationship summaries.

Unsolicited TEAE will be summarized up to 28 days after any injection. Unsolicited TEAEs may be summarized throughout the study and will be referred to as Overall stage (Part A), defined as from the first dose of study IP to the earlier date of (study discontinuation, study completion, death, data cutoff for safety, PDV/unblinding) for those who did not receive study IP in Part B; or from the first dose of study IP to the earlier date of (study discontinuation, study completion, death, data cutoff for safety, or 1st dose of study IP in Part B) for those who received study IP in Part B.

For AE events (Preferred Terms) with at least 7 participants in any treatment group who report the event, rate ratio and its 95% CI will be provided as a helpful descriptive measure to be used in review, not a formal method for assessing the statistical significance of the between-group differences in adverse experiences. The rate ratio is calculated as the ratio of percentage of participants reporting the event in mRNA-1273 divided by that in Placebo. The 95% CI for rate ratio will be provided using the Miettinen and Nurminen

method (1985). The threshold of at least 7 participants was chosen because the 95% CI for rate ratio will always include one when groups of equal size each have <7 participants reporting the event, and thus would add little for review purpose.

Percentages will be based upon the number of subjects in the Safety Set within each treatment group.

6.6.1.1. Overview of Unsolicited TEAEs

An overall summary of unsolicited TEAEs including the number and percentage of subjects by treatment group who experience the following will be presented:

- Any unsolicited TEAEs
- Any serious AEs
- Any unsolicited AEs that are medically-attended
- Any unsolicited TEAEs leading to discontinuation from participation in the study
- Any unsolicited severe TEAEs (severity of AEs is assessed as mild, moderate and severe)
- Any unsolicited TEAEs that are fatal

The table will also include number and percentage of subjects with unsolicited TEAEs that are treatment-related in each of the above categories.

The overall summary will be provided for unsolicited TEAE up to 28 days after any injection, and for the Overall Stage throughout Part A respectively.

In addition, separate listings containing individual subject adverse event data for unsolicited TEAEs leading to discontinuation from study vaccine, unsolicited TEAEs leading to discontinuation from participation in the study, serious AEs, serious treatment-related AEs, and unsolicited treatment-related medically-attended AEs may be provided. Listing of deaths including cause of death, and listing of TEAEs in subjects who died will be provided.

6.6.1.2. TEAEs by System Organ Class and Preferred Term

The following summary tables of TEAEs will be provided by SOC and PT using frequency counts and percentages (i.e. number and percentage of subjects with an event):

- All unsolicited TEAEs
- All unsolicited TEAEs that are treatment-related
- All serious AEs
- All serious AEs that are treatment-related
- All unsolicited TEAEs leading to discontinuation from study vaccine
- All unsolicited TEAEs leading to discontinuation from participation in the study
- All unsolicited severe TEAEs
- All unsolicited severe TEAEs that are treatment-related
- All unsolicited AEs that are medically-attended

Summary tables of all unsolicited TEAEs, Serious AEs, treatment-related SAEs, MAAEs, and TEAE leading to discontinuation from participation in the study will be also be provided by SOC and PT for the Overall Stage

6.6.1.3. TEAEs by Preferred Term

The following summary tables of TEAEs will be provided by PT sorting by frequency on the mRNA-1273 group:

- All unsolicited TEAEs

6.6.1.4. TEAEs by Toxicity Grade

The following summary tables of TEAEs will be provided by the maximum Toxicity Grade using frequency counts and percentages:

- All unsolicited TEAEs

- All unsolicited TEAEs that are treatment-related

6.6.1.5. Subgroup Analysis of TEAEs

An overview of TEAE, TEAE summaries presented by SOC and PT will be provided for the following subgroups:

- Age group (<65, and ≥ 65 years)
- Sex (male, female)
- Baseline SARS-CoV-2 status

These summaries may be provided for subgroups of select baseline characteristics.

6.6.1.6. Summary of AEs in Part B at the Final Analysis of Blinded Data

In Part B, for participants who were randomized to Placebo and requested mRNA-1273, SAE, MAAEs, and AE leading to withdrawal are collected throughout Part B.

At the time of the final analysis of blinded data (from the blinded phase of the study) (Section 6.4.3), in addition to the planned analysis of safety for the blinded phase (Part A), summary of SAE, MAAEs, and AE leading to withdrawal in Part B may be provided. The summary will be provided using the Part B groups during the Part B analysis period for safety as described in the table below.

Table 5 Part B groups and analysis period for safety

**Part B Cohort**	**Description**	**Part B Analysis Period for Safety**
mRNA-1273	Participants received at least one dose of mRNA-1273 in the Blinded Phase	From PDV/unblinding to earlier (data cutoff date for safety, last observed in Study).<br>If a participant received mRNA-1273 in Part B, from 1st date of study IP in Part B to earlier date of (data cutoff date for safety, last observed in Study).
Placebo	Participants only received Placebo in the Blinded Phase and did not receive mRNA-1273 in Part B	From PDV/unblinding to earlier date of (data cutoff date for safety, last observed in Study)

Placebo-mRNA-1273	Participants only received Placebo in the Blinded phase and received mRNA-1273 in Part B	From first dose of mRNA-1273 in Part B to earlier date of (data cutoff date for safety, last observed in Study)

6.6.2. Solicited Adverse Reactions

An AR is any AE for which there is a reasonable possibility that the test product caused the AE. The term "Solicited Adverse Reactions" refers to selected signs and symptoms occurring after injection administration during a specified post-injection follow-up period (day of injection and 6 subsequent days). The solicited ARs are recorded by the subject in eDiary. The occurrence and intensity of selected signs and symptoms is actively solicited from the subject during a specified post-injection follow-up period (day of injection and 6 subsequent days), using a pre-defined checklist in the eDiary (i.e. solicited ARs).

The following local ARs will be solicited by the eDiary: pain at injection site, erythema (redness) at injection site, swelling/induration (hardness) at injection site, and localized axillary swelling or tenderness ipsilateral to the injection arm.

The following systemic ARs will be solicited by the eDiary: headache, fatigue, myalgia (muscle aches all over the body), arthralgia (aching in several joints), nausea/vomiting, body temperature (potentially fever), and chills.

The solicited ARs will be graded based on the grading scales presented in Table 4 in the protocol, modified from the Toxicity Grading Scale for Healthy Adult and Adolescent Volunteers Enrolled in Preventative Vaccine Clinical Trials (DHHS 2007). Investigator will assess the Grading for Grade 4 events (with exception of fever).

If a solicited local or systemic AR continues beyond 7 days post injection, the subject will be prompted to capture solicited local or systemic AR in the eDiary until resolution.

All solicited ARs (local and systemic) will be considered causally related to injection.

Analyses of solicited ARs will be provided by treatment group for each injection (first or second) based on the associated subset of Solicited Safety Set, i.e. First (Second) Injection

Solicited Safety Set; and for any injection based on the Solicited Safety Set, unless otherwise specified.

The number and percentage of subjects who reported each individual solicited local AR (has a severity grade of Grade 1 or greater) and solicited systemic AR (has a severity grade of Grade 1 or greater) during the 7-day follow-up period after each injection will be provided by severity grade. The number and percentage of subjects who reported each individual solicited AR will also be summarized by severity grade, days of reporting and injection.

The number and percentage of subjects experiencing fever (a temperature greater than or equal to 38.0°C/100.4°F by the oral, axillary, or tympanic route) by severity grade and the number and percentage of subjects experiencing a fever of Grade 3 or higher temperature (a temperature greater than or equal to 39.0°C/102.1°F by the oral, axillary, or tympanic route) will be provided.

A two-sided 95% exact CI using the Clopper-Pearson method will be provided for the percentage of subjects who reported any solicited local AR, solicited systemic AR, or any solicited AR.

The onset of individual solicited AR is defined as the time point after each injection at which the respective solicited AR first occurred. The number and percentage of subjects with onset of individual solicited AR will be summarized by study day relative to the corresponding injection (Day 1 through Day 7).

The number of days will be calculated as the days of the solicited AR is reported within the 7 days of injection including the day of injection, no matter it is intermittent or continued. If the solicited AR continues beyond 7 days, the consecutive days a solicited AR is reported after 7 days will be included (e.g. an event that lasted 5 days in the first 7 days post injection and 3 consecutive days beyond 7 days post injection, the duration will be reported as 8 (5+3) days.)

All solicited ARs that continue beyond 7 days post injection will be summarized.

The above analyses of solicited ARs will be provided for the following subgroups:

- Age group (<65, and ≥ 65 years)
- Sex (male, female)
- Baseline SARS-CoV-2 status

These summaries may be provided for subgroups of select baseline characteristics.

6.6.3. Vital Sign Measurements

Vital sign measurements, including systolic and diastolic blood pressures, heart rate, respiratory rate, and body temperature, will be presented in a data listing. The values meeting the toxicity grading criteria will be flagged in the data listing. The abnormalities meeting the toxicity grading criteria (Grade 2 or higher) in any vital sign measurement will be listed separately. If a subject has a vital sign result with Grade 2 or higher abnormality at any post injection visit, then all results of vital sign measurement for that subject will be presented in the listing.

Observed values and changes from baseline for all vital sign measurements will be summarized at each visit by baseline SARS-CoV-2 serostatus and treatment group as defined in Section 6.1. Shift from baseline in the toxicity grades may be summarized at each visit by treatment group.

In addition, summary of pregnancies and outcome will be provided by treatment group. A listing of pregnancies including outcome will be provided.

6.7. Immunogenicity Analysis

There are two objectives on immunogenicity in this study: to characterize the immunogenicity of mRNA-1273 (secondary objective) and to assess correlates of risk and protection (exploratory objective).

This SAP includes analyses of immunogenicity data for the secondary immunogenicity objective: To evaluate the immunogenicity of 2 doses of mRNA-1273 given 28 days apart.

The analysis of assessing correlates of risk and protection will be included in a separate Immune Correlates SAP.

6.7.1. Sampling of the Immunogenicity Subset (Case-cohort Sampling Design)

For characterizing immunogenicity of the vaccine, and for assessing correlates of risk and protection (CoR/CoP), a case-cohort sampling design will be used for measuring bAb and nAb data from a randomly sampled subset of trial participants [the case-cohort Immunogenicity Analysis Set (ccIAS)], or Immunogenicity Subset. The ccIAS cohort consists of a stratified random sample of trial participants (Random SubCohort), augmented with a subset of all primary endpoint cases and SARS-CoV-2 infection endpoint cases. The immunogenicity samples of the ccIAS cohort will be processed and the immunogenicity data will be available for the ccIAS cohort.

A case-cohort sampling design (Prentice (1986)) is used for measuring antibody markers in a random sample of study participants and from all or a subset of cases of COVID-19 primary endpoint (COV-DIS) and infection endpoint (COV-INF) (Figure 3). The random sample is stratified by the key baseline characteristics: assigned treatment arm, baseline SARS-CoV-2 status (defined in Section 6.1), stratification factor used for randomization, and minority (defined as Race being Blacks or African Americans, American Indians or Alaska Natives, Native Hawaiians, and other Pacific Islanders; or Ethnicity being Hispanics or Latinos).

This design uses a stratified random sample instead of the simple random sample proposed by Prentice (1986), the design may also be referred to as a two-phase sampling design, where “phase one” refers to variables measured in all participants and “phase two" refers to variables only measured in a subset (thus the “case-cohort sample" constitutes the phase-two data, e.g. antibodies).

Figure 3 Case-cohort sampling design: participants selected into the stratified random sample (Random Subcohort) and in all or a subset of COVID-19 and COV-INF cases occurring outside of the Random Subcohort.

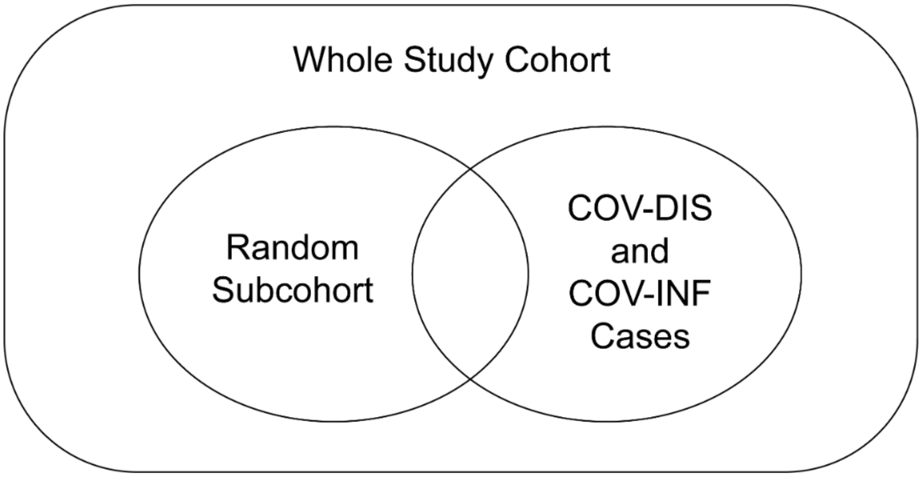

The Immunogenicity Subset will be used to characterize immunogenicity of mRNA-1273, and to assess correlates of risk and protection. The analysis of characterizing immunogenicity of mRNA-1273 is included in Section 6.7 of this SAP; the analysis of assessing correlates of risk and protection will be included in a separate Immune Correlates SAP.

Eligibility criteria for Immunogenicity SubSet

For stratified random sample:

Only participants in the FAS Set with non-missing key baseline characteristics for the strata, and with serum samples available at both Day 1 (baseline) and Day 57 are eligible for sampling into the stratified random sample (Random Subcohort).

Adjudicated COVID-19 cases and COV-INF cases:

Correlates of Risk (CoR) and Correlates of Protection (CoP) analysis will be documented in a separate Immune Correlates SAP. For CoR/CoP analysis, adjudicated COVID-19 primary endpoint cases (section 6.3.1.1) regardless of baseline SARS-CoV-2 status, infection cases on mRNA-1273 regardless of baseline SARS-CoV-2 status, and adjudicated COVID-19 cases and infection cases on Placebo in participants with baseline

positive SARS-CoV-2 status are eligible to be included regardless of the time of their event date.

<u>*Sample size* for the stratified random sample (Random Subcohort)</u>

Table 6 describes the numbers of participants sampled into the stratified random sample (Random Subcohort), for each of the 24 sampling strata defined by randomization arm, baseline SARS-CoV-2 status, stratification factor used for randomization (Age ⩾ 18 and < 65 years and not at risk, ⩾ 18 and < 65 years and at risk 18-64 not 'at risk', Age ⩾ 65), and minority status. Participants are randomly sampled into the Random Subcohort within each of the strata. This sampling design enables characterization immune response (bAb and nAb readouts) in all relevant subgroups. The sampling is implemented without replacement. A sampling list for the Random Subcohort was planned to be generated after all baseline characteristics for the strata were available and most of the participants have completed the Day 57 visit or discontinued the study.

Table 6 summarizes the size of the Random Subcohort, by baseline factors used to stratify the random sampling. For each of the mRNA-1273 and placebo arms, if any of the 6 baseline SARS-CoV-2 positive strata have fewer than 60 participants enrolled such that it is not possible to sample 60 participants, then all participants in the stratum will be sampled.

Table 6 Random Subcohort Sample Sizes by Baseline Strata

	**Baseline SARS-CoV-2 Negative (Total N=1224)**						**Baseline SARS-CoV-2 Positive (Total N=720)**					
**Baseline Covariate Strata[1]**	**1**	**2**	**3**	**4**	**5**	**6**	**1**	**2**	**3**	**4**	**5**	**6**
**Planned Evaluable participants (Total N=1620)**												
**mRNA-1273**	150	150	150	150	150	150	50	50	50	50	50	50
**Placebo**	20	20	20	20	20	20	50	50	50	50	50	50

**Adjusting for approximately 15% participants who may be excluded from the Per-protocol Subcohort for immunogenicity (Total N=1944)**												
**mRNA-1273**	**180**	**180**	**180**	**180**	**180**	**180**	**60**	**60**	**60**	**60**	**60**	**60**
**Placebo**	**24**	**24**	**24**	**24**	**24**	**24**	**60**	**60**	**60**	**60**	**60**	**60**

1 = ⩾ 18 and < 65 years and not at risk, Minority; 2 = ⩾ 18 and < 65 years and at risk, Minority; 3 = Age ⩾ 65, Minority; 4 = ⩾ 18 and < 65 years and not at risk, non-Minority; 5 = ⩾ 18 and < 65 years and at risk, non-Minority; 6 = Age ⩾ 65, non-Minority

6.7.2. Per-Protocol Random Subcohort for Immunogenicity

The Per-Protocol Random Subcohort for Immunogenicity (PPRSI) will be used as the analysis population to characterize immunogenicity of mRNA-1273. The Per-Protocol Subcohort for immunogenicity consists of participants in the FAS who are sampled into the Random Subcohort and
a). received both planned doses (i.e. received the treatment as the participant was randomized to) with dose 2 received within [21, 42] days after dose 1, and
b). no major protocol deviation that impact critical or key data.
A subset of the planned Random Subcohort that is representative of the Random Subcohort may be used to expedite analyses to characterize immunogenicity of mRNA-1273.

6.7.3. Immunogenicity Data

The following immunogenicity assessments (assays) may be used in this study:

- Serum bAb level against SARS-CoV-2 as measured by enzyme-linked immunosorbent assay (ELISA) specific to the SARS-CoV-2 S protein (vaccine antigen), tests may include VAC65 Spike IgG Antibody.
- The MSD-ECL Multiplex Assay (MSD-ECL = meso scale discovery – electrochemiluminescence assay) measures binding antibody to antigens corresponding to: Spike; the Receptor Binding Domain (RBD) of the Spike protein; and Nucleocapsid protein (NP), which is not contained in mRNA-1273.

- Pseudovirus nAb assay for measuring neutralizing antibodies against SARS-CoV-2 Spike-pseudotyped viruses: Pseudovirus neutralizing antibody ID50 and ID80 titers (PsVNT50 and PsVNT80)
- Wild Type Live virus-nAb assays measuring antibody-mediated neutralization of live wild-type SARS-CoV-2 (Wild Type Live virus-nAb MN [microneutralization] 50 titers)

6.7.4. Summary of Antibody-Mediated Immunogenicity Endpoints

Data from quantitative immunogenicity assays will be summarized for each treatment group using positive response rates and geometric means with 95% CI at each timepoint when an assessment is performed. Data from qualitative (i.e., yielding a positive or negative result) assays will be summarized by tabulating the frequency of positive responses for each assay by treatment group and by baseline status based on bAb specific to SARS-CoV-2 NP as measured by *Roche Elecsys* SARS-CoV-2 assay, if applicable, at each timepoint that an assessment is performed.

At the time of the final blinded efficacy analysis, immunogenicity analysis to characterize immunogenicity of mRNA-1273 may be performed. At the time of immunogenicity analysis, data for bAb to Spike (MSD, PPD-VAC65), RBD (MSD); and Pseudovirus neutralizing antibody ID50 and ID80 titers are expected. The assay by MSD to measure bAb against RBD may not have been validated yet at the time of the immunogenicity analysis, in that situation, bAb against RBD by MSD will not be summarized/analyzed. Immunogenicity data at the pre-specified timepoints: baseline (Day 1), Day 29 and Day 57 will be included.

- Geometric mean titer (GMT) or value with corresponding 95% CI will be provided at each time point. The 95% CIs will be calculated based on the t-distribution of the log-transformed values then back transformed to the original scale for presentation. GM level and corresponding 95% CI will be plotted at each timepoint. The

following descriptive statistics will be also provided at each time point: the number of subjects (n), median, minimum and maximum.

- Geometric mean fold rise (GMFR) with corresponding 95% CI will be provided at each post-baseline timepoint over pre-injection baseline at Day 1. The 95% CIs will be calculated based on the t-distribution of the log-transformed values then back transformed to the original scale for presentation. GMFR and corresponding 95% CI will be plotted at each timepoint. The following descriptive statistics will be also provided at each time point: the number of subjects (n), median, minimum and maximum.
- Reverse cumulative distribution function curve (RCDF) at each time point. The number and percentage of subjects with fold-rise ≥ 2, fold-rise ≥ 3, and fold-rise ≥ 4 from Baseline at each post injection time points will be tabulated with 2-sided 95% Clopper Pearson CIs. If assay-specific fold rise criteria are available, the number and percentage of subjects with fold-rise exceeding the criteria with 95% CI will be provided instead.
- Proportion of subjects with seroconversion due to vaccination will be tabulated with 2-sided 95% Clopper-Pearson CIs at each post-baseline timepoint. Seroconversion due to vaccination at a subject level may be defined as a change from below the LLOQ to equal to or above LLOQ, or 4-fold rise if baseline is equal to or above LLOQ. The definition of seroconversion may depend on assay-specific performance characteristics and the final definition of seroconversion due to vaccination will be documented in a memo.

The GMT and GM levels will be calculated using the following formula:

$$10^{\left\{\frac{\sum_{i=1}^{n}\log_{10}(t_i)}{n}\right\}}$$

where $t_1, t_2, \ldots, t_n$ $t_1, t_2, \ldots, t_n$ are n observed immunogenicity titers or levels.

The geometric mean fold rise (GMFR) measures the changes in immunogenicity titers within subjects. The GMFR will be calculated using the following formula:

$$10^{\left\{\frac{\sum_{i=1}^{n}\log_{10}\left(\frac{v_{ij}}{v_{ik}}\right)}{n}\right\}} = 10^{\left\{\frac{\sum_{i=1}^{n}\log_{10}(v_{ij})-\log_{10}(v_{ik})}{n}\right\}}$$

where, for *n* subjects, v_{ij} v_{ij} and v_{ik} v_{ik} are observed immunogenicity titers or levels for subject *i* at time points *j* and *k*, $j \neq k$.

6.7.5. Analysis of Antibody-Mediated Immunogenicity Endpoints

To assess the magnitudes of the differences between the two treatment groups (mRNA-1273 vs. Placebo) in SARS-CoV-2-specific nAb and S protein-specific bAb, a mixed model for repeated measures (MMRM) using SAS PROC MIXED will be used.

For each of the SARS-CoV-2-specific nAb and S protein-specific bAb, the analysis will be performed separately for subjects who are seropositive and seronegative (as measured by Roche Elecsys SARS-CoV-2 assay) at baseline respectively. The model will include all available log-transformed antibody titers at each post-baseline visit as the dependent variable, treatment groups, visit (as a class variable), and treatment-by-visit interaction visit as fixed effects, with adjustment for stratification factor used for randomization, baseline log-transformed anti-body titers if applicable, and subject as a random effect. The model may also adjust for minority group used to sample for the random subcohort. An unstructured covariance structure will be used to model the within-subject errors. A Kenward-Roger approximation will be used for the denominator degrees of freedom. If there is a convergence issue due to the unstructured covariance matrix, a compound symmetry covariance structure will be used to model the within-subject errors. No imputation of missing data will be done.

The geometric least squares mean (GLSM) and corresponding 2-sided 95% CI for the anti-body titers for each treatment group will be provided by visit. The GLSM, and corresponding 95% CI results in log-transformed scale estimated from the model will be

back-transformed to obtain these estimates in the original scale. Geometric mean ratio (GMR), estimated by the ratio of GLSM and the corresponding 2-sided 95% CI will be provided to assess the treatment difference between mRNA-1273 group vs. placebo group at each visit. With each treatment group, within-group comparison of timepoints may also be estimated using the same model.

In addition, an analysis of covariance (ANCOVA) model with the treatment group as explanatory variables, adjusting for the stratification factor at randomization, baseline value if applicable, will be used to assess the treatment effect at specific timepoints (scheduled visits) such as Day 57. GLSM with 95% CI for each treatment group, GMR with 95% CI for treatment difference will be estimated from the ANCOVA model. The model may also adjust for minority group used to sample for the random subcohort.

6.7.6. Summary of Immunogenicity in COVID-19 Cases on mRNA-1273 with negative SARS-CoV-2 status at baseline

To characterize immunogenicity of mRNA-1273, immunogenicity data (Section 6.7.3) will be analyzed using the Per-Protocol Random Subcohort for Immunogenicity.
It is of interest to examine the immune response in subjects on mRNA-1273 with baseline SARS-CoV-2 negative status who were COVID-19 or SARS-CoV-2 infection cases. Summary of immunogenicity may be provided in a subset of baseline SARS-CoV-2 negative participants on mRNA-1273 who were COVID-19 cases based on adjudication committee assessments.

6.8. Exploratory Analysis of Efficacy

Exploratory analyses of efficacy described in this section will not be performed at the interim analyses, and may be performed at the primary analysis, with the exception of exploratory analyses of Burden of Disease (BOD) and Burden of Infection (BOI) that will be performed at the interim analyses and provided to the DSMB.

6.8.1. Vaccine Efficacy Against Burden of Disease

Exploratory analysis of BOD due to COVID-19 will be performed. A BOD score is defined based on the post SARS-CoV-2 infection follow-up to reflect the worst severity of symptoms as shown in the table below.

Table 7 Burden of Disease Score

**Subject State**<br>**(Worst Category Following Disease Detection)**	**BOD Score**
Without COVID-19<br>(uninfected/asymptomatic infection)	0
COVID-19 without hospitalization<br>(symptomatic without hospitalization)	1
COVID-19 with hospitalization	2
Death	3

Summary of BOD score, number and percentage of subject with each level of BOD score, will be provided by treatment group using the PP Set. A t-test of the BOD scores may be performed to compare mRNA-1273 vs. placebo group.

To assess impact of baseline risk of severe disease on the vaccine effect regarding disease severity, summary of BOD may be provided by randomization strata (i.e. ≥ 65 years, < 65 years at risk, and < 65 years not at risk).

In order to assess the disease burden in subjects with COVID-19, the above analyses may also be performed in participants with COVID-19, i.e. subjects with BOD score of zero will be excluded from the analysis.

While transparent, the proportions in the summary table will change with increased follow-up time as more subjects inevitably acquire disease. This makes comparisons across

different periods of follow-up difficult. To augment this analysis, a proportional means model will be used to assess the treatment effect on BOD between mRNA-1273 and placebo in terms of ratio of mean severity score. The proportional means model allows for direct comparisons across different periods of follow-up, unaffected by differential follow-up. A proportional means model including vaccination group as fixed effect and stratified with stratification factor at randomization will be used to assess the vaccine effect on BOD. The VE for the BOD score will be estimated as 1 minus the ratio of means as estimated by the proportional means model and reported with 95% confidence intervals.

6.8.2. Vaccine Efficacy Against Burden of Infection

To fully understand the impact of vaccination on disease severity, asymptomatic infections should also be evaluated. Similarly, a burden of infection (BOI) score will be calculated as in the table below and used to understand the impact. Because asymptomatic infection is identified by seroconversion only at scheduled visits at which samples for RT-PCR test and/or serum samples will be taken (e.g. months 1, 2, 7, 13 and 25), summary of BOI will be provided for relevant periods (e.g. Baseline through M1, M1 through M2, M2 through M7, M7 through M13, and M13 through M25) only using subjects who have serological data for that period. With protocol amendment 6, at the unblinding/participant decision visit/Open-label-Day 1 (OL-D1) visit, NP swab for RT-PCR tests and serum samples will be taken. The relevant periods will be updated to Baseline through M1, M1 through M2, M2 through M7/OL-D1 whichever is earlier.

A t-test of the BOI scores may be performed to compare mRNA-1273 vs. placebo group. A proportional means model including vaccination group as fixed effect and stratified with stratification factor at randomization will be used to assess the vaccine effect on BOI. The VE for BOI will be estimated as 1 minus the ratio of mean BOI scores and reported with 95% confidence intervals.

Table 8 Burden of Infection Score

Patient State	BOI Score

**(Worst Category Following Disease Detection)**	
No infection	0
Asymptomatic infection	1/2
COVID-19 without hospitalization<br>(Symptomatic without hospitalization)	1
COVID-19 with Hospitalization	2
Death	3

6.8.3. Vaccine Efficacy on Duration and Presence/Severity of COVID-19 Symptoms

Subjects will report symptoms including fever (temperature ≥ 38ºC), chills, cough, shortness of breath, difficulty breathing, fatigue, muscle aches (myalgia), body aches, headache, new loss of taste, new loss of smell, sore throat, nasal congestion, runny nose (rhinorrhea), nausea, vomiting, and diarrhea. The severity scoring system of the symptoms is presented in the table below. A score endpoint aggregating duration and presence/severity of COVID-19 symptoms may be calculated as the sum of daily severity scores of all recorded symptoms across all days with symptoms. The VE of mRNA-1273 based on mean score of each treatment group will be calculated as below:

VE = [1 – Mean score of mRNA-1273 group / Mean score of placebo group] × 100%

Table 9 Grading of COVID-19 Symptoms

Grading	All Symptoms	For Nausea/Vomiting ONLY	For Sense of Smell/Taste ONLY	Score
None	No symptom			0

Mild	I had the symptom, but I could still do my normal activities	I was able to eat and drink normally	I had the symptom, but I retained some taste/smell	1
Moderate	The symptom really bothered me. It was hard to do my normal activities.	It bothered me enough that I did not eat or drink normally.	My taste/smell was significantly affected.	2
Severe	The symptom was very bad. I was not able to do activities that I usually do.	I could not eat or drink.	I have no taste or smell.	3

A proportional means model including vaccination group as fixed effect and stratified with stratification factor at randomization will be used to assess the VE (mRNA-1273 vs. placebo) on the score aggregating duration and presence/severity of COVID-19 symptoms based on the PP Set. The score will be summarized and categorized into quartiles (across the two groups) using 1, 2, 3, 4. The indication of the quartiles will be used as the dependent variable in the proportional mean model.

6.8.4. Vaccine Efficacy Against All-cause Mortality

To evaluate VE against all-cause mortality, the same Cox PH model described above based on the PP Set, mITT Set, and FAS will be used. Death, regardless of cause, from randomization will be included. Time to death will be calculated as date of death – date of randomization + 1. At the final analysis of blinded data, subjects with no documented death will be censored.

6.8.5. Vaccine Efficacy Against COVID-19 Over Time

For exploratory analysis of the VE on the COVID-19 primary endpoint over time, to assess if there is any time trend of the effect of the vaccine, e.g. if the effect of the vaccine is waning over time during the follow-up, an extended Cox model with an addition of a covariate incorporating interaction of time by treatment group may be carried out.

For exploratory analysis of the VE on the COVID-19 primary endpoint over time, the instantaneous hazard ratio (mRNA-1273 vs. placebo) of the endpoint may be estimated with pointwise and 95% confidence intervals, using nonparametric kernel smoothing estimation of each of the mRNA-1273 and placebo arm hazard functions over time, using the method of Gilbert *et al*. (Gilbert, 2002) with optimal bandwidths selected using the analytical formula in Andersen *et al*. (Andersen, 1993). At each interim analysis, a plot of the hazard ratio results, as well as plots of the point estimates and pointwise and simultaneous 95% confidence interval estimates for the treatment-arm specific hazard functions, may be provided.

6.8.6. Durability of Vaccine Efficacy Against COVID-19

Durability of vaccine efficacy will be assessed by the incidence rate of COVID-19 by time periods.

As an exploratory analysis to assess durability of VE against the primary endpoint, covariate adjustment method based on cumulative incidence may be used. Cumulative incidence VE may be assessed using a parameter $VE_{CI}(0-t)$, defined as one minus the ratio of cumulative incidences (mRNA-1273 vs. placebo) of the COVID-19 endpoint over follow-up through time t post enrollment. All times t ranging from 0 to $t*$ will be considered, where $t*$ is defined as the final time through which at least 1,000 subjects are at risk for the COVID-19 endpoint. The cumulative incidence for each vaccination group will be estimated using a covariate adjustment method based on Zeng (2004) that makes use of baseline subjection information. These covariates will include demographic information such as age, sex, and race/ethnicity, and stratification factor at randomization.

The most useful covariates to adjust for in producing potential efficiency gains are potential modifiers of VE.

The covariate adjustment method that will be used is more robust than unadjusted approaches (e.g., the Kaplan-Meier estimator), as it yields valid inferences even in cases in which loss to follow-up is informative, provided within each intervention arm the possible relationship between censoring and the COVID-19 endpoint can be explained by observed baseline covariates. Unadjusted methods, in contrast, can give biased answers in this setting, which manifests as 95% confidence intervals that contain the true VE less than 95% of the time, or as tests of the null H_0: VE ≤ 30% that reject with probability greater than the specified type 1 error level. Also, the covariate adjustment method often results in more precise estimates when available baseline covariates (e.g. stratification factor at randomization) are predictive of the COVID-19 endpoint, thereby yielding tighter confidence intervals for the cumulative VE and higher-powered tests of the null hypothesis. This efficiency gain may be especially pronounced in settings where the randomization is not stratified on factors such as age category or trial site.

The estimator for the survival function in arm v (mRNA-1273 or placebo) that adjusts for baseline covariates L is implemented using the following steps:

1. (a) Using only data from arm v, fit a main terms Cox regression to estimate the hazard of the COVID-19 endpoint conditionally on L. Refer to the fitted vector of coefficients as $\hat{\beta}$.

   (b) Repeat step (a), but estimate the hazard of *censoring* conditionally on L. Refer to the fitted vector of coefficients as $\hat{\gamma}$.

2. For given coefficient $\hat{\alpha}$ (= $\hat{\beta}$ or $\hat{\gamma}$) and a given covariate value ℓ, let $f_{\hat{\alpha}}(\ell)$ denote the $j \in 1, \ldots, 4$ for which $q_{\hat{\alpha},j-1} < \hat{\alpha}^\top \ell \leq q_{\hat{\alpha},j}$, where $q_{\hat{\alpha},1}, q_{\hat{\alpha},2}, q_{\hat{\alpha},3}$ denote the empirical quartiles of $\hat{\alpha}^\top L$ in arm v and $q_{\hat{\alpha},0} = -\infty$ and $q_{\hat{\alpha},4} = \infty$.

3. Using only data from arm v, fit a stratified Kaplan Meier using the COVID-19 endpoint as outcome within all non-empty strata of $(f_{\hat{\beta}}(L), f_{\hat{\gamma}}(L))$ – there will be at most 16 such strata. If any strata are empty, estimate the survival function within those strata as the constant function 1.
4. Estimate the arm-specific survival function by taking a weighted average across the 16 strata, where each stratum-specific survival function receives weight equal to the empirical probability of a randomly selected Subject (from either the vaccine or placebo arm) having baseline covariates belonging to that stratum.

Estimates of the arm-specific cumulative incidences and cumulative incidence VE are obtained by transforming the estimated arm-specific survival functions. Missing covariates will be accounted for using median imputation, which preserves the randomization of treatment even given imputed covariates.

The validity of this estimator relies on the condition that, within all strata of L, there must be a positive probability that a Subject will have follow-up through time t^*. As such, covariates such as trial site should not be included in if the enrollment timeline is such that this condition fails to hold.

Two-sided Wald-based 95% confidence intervals for a log-transformed cumulative incidence ratio estimate will be provided, and these intervals will be transformed to yield intervals for $VE_I(0-t)$, $t \in [0, t^*]$. Accompanying uniform confidence bands, constructed using the multiplier bootstrap with Rademacher weights (Kosorok 2007), will also be provided. The covariate-adjusted arm-specific cumulative incidence estimates will also be plotted over time, with confidence intervals and uniform confidence bands defined by transforming a Wald interval for a complementary log-log transformation of the corresponding survival functions.

6.9. Summary of Protocol Safety Review and Data Safety Monitoring Board

6.9.1. Protocol Safety Review Team

A Protocol Safety Review Team (PSRT) will be formed to review interim and cumulative blinded safety data on a regular basis with a remit to escalate concerns to the DSMB. The PSRT will be composed of the study's three coordinating investigators and representatives from Moderna, National Institute of Allergy and Infectious Diseases (NIAID), Biomedical Advanced Research and Development Authority (BARDA), and the CRO. The PSRT composition, its remit, and the routine and systematic procedures for safety review and oversight of blinded interval and cumulative data during the mRNA-1273 P301 study by the PSRT is documented in the Moderna mRNA-1273 P301 Protocol Safety Review Team Charter.

6.9.2. Data Safety Monitoring Board (DSMB)

The Coronavirus Disease 2019 (COVID-19) Vaccine Data and Safety Monitoring Board (DSMB) will monitor all randomized COVID-19 vaccine studies supported by the United States Government (USG). The independent DSMB will periodically review blinded and unblinded data of mRNA-1273-P301, including

- continuous monitoring for potential vaccine harm and non-efficacy based on imbalance between mRNA-1273 vs. placebo for both COVID-19 and severe COVID-19 case counts.
- regular review of safety data at scheduled data review meetings
- review the two planned interim analyses

The statistical considerations and methods for DSMB is documented in the Analysis Plan for DSMB (current version: Version 2.0 dated 07-August-2020).

6.9.2.1. Potential Vaccine Harm

The DSMB has continuously monitored potential vaccine harm in this study. Potential vaccine harm is assessed based on imbalance between mRNA-1273 vs. placebo for both COVID-19 and severe COVID-19 case counts using the Safety Set. For harm monitoring,

subjects are analyzed according to the IP that they actually received regardless of the group to which they were randomized. For harm monitoring, cases are counted starting after the first dose of study vaccination.

Cases of COVID-19 for the harm monitoring are defined as for the primary efficacy endpoint (eligible symptoms with positive RT-PCR result) but are based on the Safety Set rather than the PPS. For harm monitoring, cases of COVID-19 are derived based on RT-PCR results and symptoms collected in the clinical database to facilitate continuous monitoring.

Details of harm monitoring are provided in the analysis plan for DSMB. The monitoring for potential harm based on COVID-19 would continue to the first interim analysis, and the DSMB can decide how to continue; the harm monitoring for potential harm based on the number of severe COVID-19 cases would continue at least until the primary analysis.

DSMB recommended discontinuation of the harm monitoring reports to the DSMB after the DSMB review meeting on 29-Jan-2021. Thus, harm monitoring is considered completed and discontinued per DSMB recommendation effective immediately.

7. Changes from Planned Analyses in Protocol

Not applicable.

8. References

Andersen, P.K., Borgan, O., Gill, R.D., Keiding, N. Statistical Models Based on Counting Processes. Springer. 1993.

Estimands and Sensitivity Analysis in Clinical Trials. The Guideline on Statistical Principles for Clinical Trials. International Council for Harmonisation (ICH) E9(R1). November 2019.

Fine P.F., Gray R.J. A Proportional Hazards Model for the Subdistribution of a Competing Risk. Journal of the American Statistical Association. December 1999; Vol. 94, No. 446, 496-509.

Gilbert, P.B., Wei, L. J., Kosorok, M. R., Clemens, J. D. Simultaneous Inferences on the Contrast of Two Hazard Functions with Censored Observations. Biometrics. 2002 Dec; 58(4): 773-80.

Guidance for Industry: Toxicity Grading Scale for Healthy Adult and Adolescent Volunteers Enrolled in Preventive Vaccine Clinical Trials. U. S. Department of Health and Human Services (DHHS). Food and Drug Administration. Center for Biologics Evaluation and Research. September 2007.

Miettinen OS, Nurminen M. Comparative analysis of two rates. Statistics in Medicine 1985; 4:213-226.

Prentice, R. A case-cohort design for epidemiologic cohort studies and disease prevention trials. Biometrika. 1986, 73, 1-11.

Zeng D. Estimating marginal survival function by adjusting for dependent censoring using many covariates. The Annals of Statistics. 2004, 32(4), 1533-1555.

9. List of Appendices

9.1. Appendix A Standards for Variable Display in TFLs

Continuous Variables: The precision for continuous variables will be based on the precision of the data itself. The mean and median will be presented to one more significant figure than the original results; the SD will be presented to two more significant figures than the original results; the minimum and maximum will be presented to the same precision as the original results.

Categorical Variables: Percentages will be presented to 1 decimal place. If the count is 0, the percentage will not be displayed. If the count equals the denominator, the percentage will be displayed as 100.

9.2. Appendix B Analysis Visit Windows

Analysis will be summarized using the following analysis visit window for post injection assessments:

Step 1: If the assessments are collected at a scheduled visit, the collected data will be mapped to the nominal scheduled visit.

Step 2: If the assessments are collected at an unscheduled visit, the collected data will be mapped using the analysis visit windows described in Table 10 below. For subjects with confirmed COVID-19, unscheduled assessments will be preferably mapped to the visits with respect to the confirmation of COVID-19 (i.e. Illness Visit Day xx) over nominal scheduled visits (i.e. Day xx).

If a subject has multiple assessments within the same analysis visit, the following rule will be used:

- If multiple assessments occur within a given analysis visit, the assessment closest to the target study day will be used.
- If there are 2 or more assessments equal distance to the target study day, the last assessment will be used.

Table 10 Analysis Visit Windows

Visit	Target Study Day	Visit Window in Study Day
**Nasopharyngeal swab (or saliva)**		
Day 1	1 (Date of First Injection)	1, Pre-first-dose
Day 29	29 (Date of Second Injection)	
Illness Visit Day 1	X (Date of NP Swab test)	X
Illness Visit Day 3	X+2	[X+1, X+2]

Illness Visit Day 5	X+4	[X+3, X+4]
Illness Visit Day 7	X+6	[X+5, X+6]
Illness Visit Day 9	X+8	[X+7, X+10]
Illness Visit Day 14	X+13	[X+11, X+16]
Illness Visit Day 21	X+20	[X+17, X+23]
Illness Visit Day 28	X+27	[X+24, X+34]
**Vital Signs**		
Day 1	1 (Date of First Injection)	1, Pre-first-dose
Day 1	1 (Date of First Injection)	1, Post-first-dose
Day 29	29 (Date of Second Injection)	[2, 43] Pre-second-dose
Day 29	29 (Date of Second Injection)	[2, 43] Post-second-dose
Day 57	57	[44, 133]
Day 209	209	[134, 301]
Day 394	394	[302, 576]
Day 759	759	[577, 773]
Illness Visit Day 1	X (Date of COVID-19 Confirmation)	X
Illness Visit Day 28	X+27	[X+2, X+34]
**Immunogenicity**		
Day 1	1	1, Pre-first-dose
Day 29	29 (Date of Second Injection)	[2, 43] Pre-second-dose

Day 57	57	[44, 133]
Day 209	209	[134, 301]
Day 394	394	[302, 576]
Day 759	759	[577, 773]
Illness Visit Day 1	X (Date of NP Swab test)	X
Illness Visit Day 28	X+27	[X+2, X+34]

9.3. Appendix C Imputation Rules for Missing Dates of Prior/Concomitant Medications and Non-Study Vaccinations

Imputation rules for missing or partial start/stop dates of medication are defined below:

1. Missing or partial medication start date:
    - If only Day is missing, use the first day of the month, unless:
        - The medication end date is on/after the date of first injection or is missing/partial AND the start month and year of the medication coincide with the start month and year of the first injection. In this case, use the date of first injection.
    - If Day and Month are both missing, use the first day of the year, unless:
        - The medication end date is on/after the date of first injection or is missing/partial AND the start year of the medication coincide with the start year of the first injection. In this case, use the date of first injection.
    - If Day, Month, and Year are all missing, the date will not be imputed, but the medication will be treated as though it began prior to the first injection for purposes of determining if status as prior or concomitant.
2. Missing or partial medication stop date:

a. If only Day is missing, use the earliest date of (last day of the month, study completion, discontinuation from the study, or death).
b. If Day and Month are both missing, use the earliest date of (last day of the year, study completion, discontinuation from the study, or death).
c. If Day, Month, and Year are all missing, the date will not be imputed, but the medication will be flagged as a continuing medication.

In summary, the prior, concomitant or post categorization of medications and non-study vaccinations is described in the table below.

Table 11 Prior, Concomitant, and Post Categorization of Medications and Non-study Vaccinations

	Medication Stop Date		
**Medication Start Date**	**< First Injection Date of IP**	**≥ First Injection Date and ≤ 28 Days After Last Injection**	**> 28 Days After Last Injection [2]**
< First injection date of IP [1]	P	P, C	P, C, A
≥ First injection date and ≤ 28 days after last injection	-	C	C, A
> 28 days after last injection	-	-	A

A: Post; C: Concomitant; P: Prior

[1] includes medications with completely missing start date

[2] includes medications with completely missing end date

9.4. Appendix D Imputation Rules for Missing Dates of AEs

Imputation rules for missing or partial start dates and stop dates of AEs are defined below:

1. Missing or partial start date:

- If only Day is missing, use the first day of the month, unless:
    - The AE end date is on/after the date of first injection or is missing/partial AND the start month and year of the AE coincide with the start month and year of the first injection. In this case, use the date and time of first injection, even if AE time was collected.

- If Day and Month are both missing, use the first day of the year, unless:
    - The AE end date is on/after the date of first injection or is missing/partial AND the start year of the AE coincides with the start year of the first injection. In this case, use the date and time of first injection, when time is available.
- If Day, Month, and Year are all missing, the date will not be imputed. However, if the AE end date is prior to the date of first injection, then the AE will be considered a pre-treatment AE. Otherwise, the AE will be considered treatment-emergent.

2. Missing or partial end dates will not be imputed.

9.5. Appendix E Schedule of Events

Please refer to Table 16, 17, 18 and 19 in Appendix 1 Schedule of Events in the protocol.

9.6. Appendix F Estimands and Estimand Specifications

Table 12 Intercurrent Event Types

Label	Intercurrent Event Type	Comment
IcEv1 (early discontinuation or death without confirmation of cases, ie, unrelated death)	Early discontinuation from study preventing from confirmation of COVID-19, ie, unrelated death or withdrawal consent prior to documented confirmed COVID-19	Participants in PP Set who withdraw consent or die due to unrelated to COVID-19 without confirmation of being a case will all be included in primary efficacy analysis.
IcEv2 (early COVID-19)	COVID-19 starting up to 14 days after the second dose of IP	Participants in PP Set who experience an early COVID-19 up to 14 days after the second dose of IP will all be included in primary efficacy analysis.

10. Abbreviation: IcEv: intercurrent event, PP: per protocol.

Table 13 Primary Objective and Estimands with Rationale for Strategies to Address Intercurrent Events for Per Protocol Analysis

**Objective: To demonstrate the efficacy of mRNA-1273 to prevent COVID-19**	
**Estimand Description**	Vaccine efficacy will be measured using 1 – HR (mRNA-1273/Placebo) of COVID-19 from 14 days after second dose of IP in adults. A hypothetical strategy will be used for early discontinuation (eg, withdrawal consent, deaths unrelated to COVID-19) or early COVID-19 in participants in PP Set..
**Target Population**	Adults aged 18 years and older in circumstances at a high risk of SARS-CoV-2 infection but without medical conditions that pose additional risk of developing severe disease.<br>The population excludes those previously infected or vaccinated for SARS-CoV-2 or with a medical condition, on treatment that poses additional risks (including those requiring immunosuppressants or immune-modifying drugs), or pre-seropositive.
**Variable/Endpoint**	Time to COVID-19 disease, censoring at early discontinuation, early COVID-19, or last assessment for an event not being observed, whichever comes earlier.
**Treatment Condition(s)**	**Test:** mRNA-1273<br>**Reference:** Placebo
**Estimand Label**	**Estimand 1**
**Population-Level Summary**	Vaccine efficacy defined as 1 - HR of mRNA-1273/Placebo
**Intercurrent Event Strategy**	
**IcEv1 (Early discontinuation):**	Hypothetical
**IcEv2 (early COVID-19):**	Hypothetical

Objective: To demonstrate the efficacy of mRNA-1273 to prevent COVID-19

**Rationale for Strategy(s)**	Hypothetical: early discontinuation (including unrelated death) censored at time of discontinuation (or at time of death), and early case will be censored at the time at case onset, handled with independent censoring.
