# Supplementary material for: Durability of Protection Against Symptomatic COVID-19 Among Participants of the mRNA-1273 SARS-CoV-2 Vaccine Trial
Source: JAMA Netw Open. 2022 Jun 8;5(6):e2215984. doi: 10.1001/jamanetworkopen.2022.15984 (PMC9178430; doi:10.1001/jamanetworkopen.2022.15984)
Supplement: Supplement 2. — Nonauthor Collaborators. The COVE Study Group [file jamanetwopen-e2215984-s002.pdf]

Supplemental Online Content: Nonauthor Collaborators
*First name, last name, and suffix (if applicable) are required and will appear in PubMed.

*Group Name(s): The COVE Study Group							
***First Name and Middle Initial(s)**	***Last Name**	***Suffix (eg, Jr, III)**	Academic Degrees	Institution	Location (city, state/province, country)	Role or Contribution, eg, chair, principal investigator	Group (if more than 1 Group listed in the byline) and/or Subgroup (eg, Steering Committee)
Moni B	Neradilek		MS	Fred Hutchinson Cancer Research	Seattle, WA, USA	Data analysis	
Holly	Janes		PhD	Fred Hutchinson Cancer Research Center	Seattle, WA, USA	Conception of study, Data analysis	
Weiping	Deng		PhD	Moderna	Cambridge, MA, USA	Study conduct	
Honghong	Zhou		PhD	Moderna	Cambridge, MA, USA	Study conduct	
